# Diverse Combinatorial Biosynthesis Strategies for C–H Functionalization of Anthracyclinones

**DOI:** 10.1021/acssynbio.4c00043

**Published:** 2024-04-25

**Authors:** Rongbin Wang, Benjamin Nji Wandi, Nora Schwartz, Jacob Hecht, Larissa Ponomareva, Kendall Paige, Alexis West, Kathryn Desanti, Jennifer Nguyen, Jarmo Niemi, Jon S. Thorson, Khaled A. Shaaban, Mikko Metsä-Ketelä, S. Eric Nybo

**Affiliations:** † Department of Life Technologies, University of Turku, FIN-20014 Turku, Finland; ‡ Department of Pharmaceutical Sciences, College of Pharmacy, Ferris State University, Big Rapids, Michigan 49307, United States; ^§^Center for Pharmaceutical Research and Innovation, ^∥^Department of Pharmaceutical Sciences, College of Pharmacy, University of Kentucky, Lexington, Kentucky 40536, United States

**Keywords:** BioBricks, synthetic biology, natural product biosynthesis, anthracyclinones, Streptomyces coelicolor, oxygenase, anticancer

## Abstract

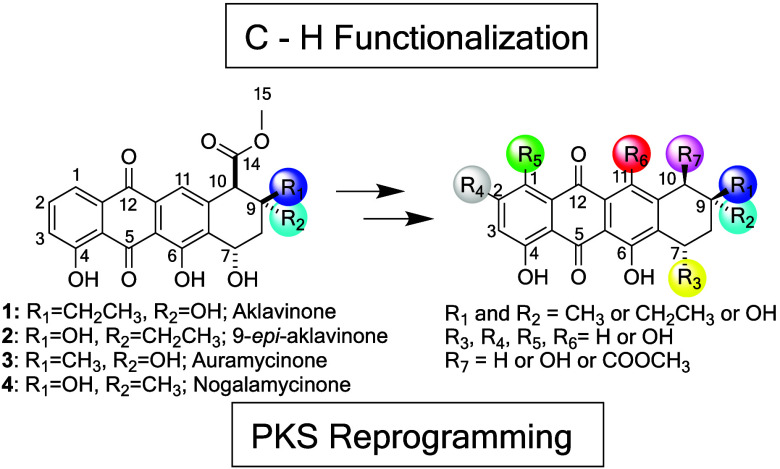

*Streptomyces* spp. are “nature’s antibiotic factories” that produce valuable bioactive metabolites, such as the cytotoxic anthracycline polyketides. While the anthracyclines have hundreds of natural and chemically synthesized analogues, much of the chemical diversity stems from enzymatic modifications to the saccharide chains and, to a lesser extent, from alterations to the core scaffold. Previous work has resulted in the generation of a BioBricks synthetic biology toolbox in *Streptomyces coelicolor* M1152Δ*matAB* that could produce aklavinone, 9-*epi*-aklavinone, auramycinone, and nogalamycinone. In this work, we extended the platform to generate oxidatively modified analogues *via* two crucial strategies. (i) We swapped the ketoreductase and first-ring cyclase enzymes for the aromatase cyclase from the mithramycin biosynthetic pathway in our polyketide synthase (PKS) cassettes to generate 2-hydroxylated analogues. (ii) Next, we engineered several multioxygenase cassettes to catalyze 11-hydroxylation, 1-hydroxylation, 10-hydroxylation, 10-decarboxylation, and 4-hydroxyl regioisomerization. We also developed improved plasmid vectors and *S. coelicolor* M1152Δ*matAB* expression hosts to produce anthracyclinones. This work sets the stage for the combinatorial biosynthesis of bespoke anthracyclines using recombinant *Streptomyces spp.* hosts.

## Introduction

Anthracyclines are glycosylated aromatic polyketides produced by various soil bacteria in the actinomycete family. Doxorubicin and aclarubicin are utilized as anticancer agents for treating various human cancers, making them some of the broadest spectrum antineoplastic agents used in the clinic.^1^ Anthracyclines inhibit the proliferation of cancer cells through two distinctive mechanisms: histone eviction and inhibition of topoisomerase II, leading to the scission of DNA strands.^1,2^ However, anthracyclines have limitations that diminish their clinical utility. Cancer cells can develop drug resistance to the anthracyclines, for example, by overexpressing the *p*-glycoprotein ATP binding cassette.^3^ Additionally, the long-term use of anthracyclines is associated with cardiotoxicity.^4^ These observations have motivated the systematic biosynthetic modification of anthracyclines to achieve new analogues with advantageous properties over currently used medications, including increased potency, decreased drug resistance, and reduced cardiotoxicity.^5^

**Figure 1:**
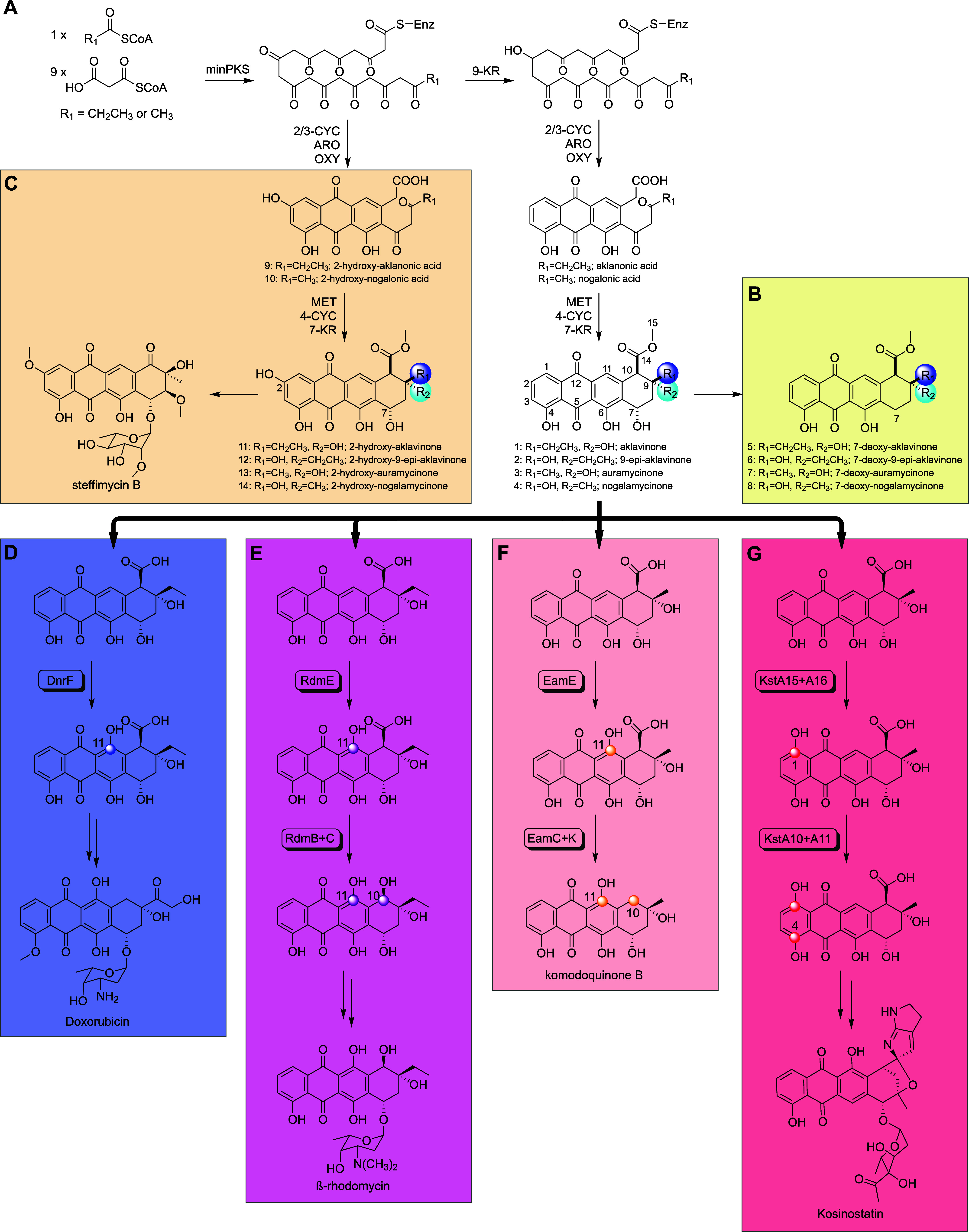
Metabolic engineering strategies for C–H functionalization of anthracyclinones. Biosynthesis of (A) the four anthracyclinone aglycones **1–4**, (B) degradation products **5–8** through the action of endogenous enzymatic activities of the host strain *S. coelicolor* and (C) 2-hydroxylated target compounds **9–14** accessible *via* PKS cassette engineering utilizing steffimycin B biosynthetic logic. Depiction of the diversity of post-PKS tailoring steps on (D) doxorubicin, (E) rhodomycin, (F) komodoquinone, and (G) kosinostatin pathways.

Anthracyclines are biosynthesized by polyketide synthase (PKS) complexes, which are composed of a minimal PKS (minPKS) consisting of a ketoacyl synthase (KSα), chain length factor (CLF or KSβ), and acyl carrier protein (ACP) that catalyzes the Claisen condensation of one molecule of acetyl-CoA or propionyl-CoA to nine molecules of malonyl-CoA (Figure 1A). The resulting poly β-keto thioester decaketide undergoes controlled folding by 9-ketoreductase (9-KR), aromatase (ARO), second-/third-ring cyclase (2/3-CYC), and oxygenase (OXY) enzymes to generate the first stable intermediates aklanonic acid and nogalonic acid. Further reactions by methyltransferase (MET), fourth-ring cyclase (4-CYC), and ketoreductase (7-KR) enzymes furnish the core tetracyclic aromatic carbon skeletons (Figure 1A).^3^ Previously, we developed a BioBricks platform for the improved biosynthesis of anthracyclinones.^6^ We developed vectors to produce four anthracyclinone scaffolds: aklavinone (**1**), 9-*epi*-aklavinone (**2**), auramycinone (**3**), and nogalamycinone (**4**) (Figure 1A). In addition, endogenous activity from the host strain *Streptomyces coelicolor* M1152Δ*matAB* led to the production of 7-deoxygenated versions of these compounds, including 7-deoxy-aklavinone (**5**), 7-deoxy-9*-epi*-aklavinone (**6**), 7-deoxy-auramycinone (**7**), and 7-deoxy-nogalamycinone (**8**) (Figure 1B).

Anthracycline biosynthetic gene clusters harbor extensive gene sets to modify the core anthracyclinone structures further. Particularly, gene products catalyzing redox chemistry for C–H functionalization, which is an important component in the chemodiversification of anthracyclines, are abundant. The daunorubicin pathway harbors the FAD-dependent 11-monooxygenase DnrF (Figure 1C).^7−9^ RdmE also catalyzes the same reaction on the rhodomycin pathway, containing the 15-methylesterase RdmC and the methyltransferase-like RdmB for C-10 hydroxylation (Figure 1D).^7,9,10^ The komodoquinone pathway includes enzymes for 10-decarboxylation by the C-15 esterase EamC and the methyltransferase-like EamK (Figure 1E).^11,12^ Finally, the kosinostatin biosynthetic gene cluster encodes the short-chain aldol reductase KstA16 and the cyclase-like KstA15 that jointly catalyze C-1 hydroxylation (Figure 1F).^13^ The reaction cascade is further extended to 4-hydroxyl regioisomerization by the NmrA-like short-chain dehydrogenase/reductase enzymes KstA11 and KstA10 (Figure 1F).^13^

In this work, we were interested in combinatorial biosynthesis and C–H functionalization of anthracyclinones using two distinct approaches. The first strategy included reprogramming the PKS cassettes to afford 2-hydroxylated analogues, similar to steffimycin biosynthesis.^14^ This could plausibly be achieved by excluding the KR gene and exchanging ARO/CYC genes with those residing on aureolic acid biosynthetic pathways (Figure 1C).^14−16^ The second strategy included diverse post-PKS tailoring genes from the kosinostatin,^13^ rhodomycin,^9,10^ doxorubicin,^8^ and komodoquinone B pathways.^11^ One significant goal of these studies was to systematically evaluate the substrate promiscuity of post-PKS tailoring enzymes toward alternative substrates **1**–**4**.

The metabolic engineering presented here resulted in the generation of 26 anthracyclinones, including nine novel analogues with regiospecific C–H oxygenation. Chemical characterization and bioactivity profiling revealed the importance of 1-, 10-, and 11-hydroxylation in the cytotoxicity of the anthracyclinones. Installing ketone, aldehyde, and alcohol functional groups provides chemical handles for group-transfer enzymes, such as methyltransferases, aminotransferases, and glycosyltransferases.^12^ This opens the door for rational metabolic engineering to generate diverse glycosylated anthracycline analogues in the future.

## Results and Discussion

### Development of Improved Strains and Vectors

**Figure 2:**
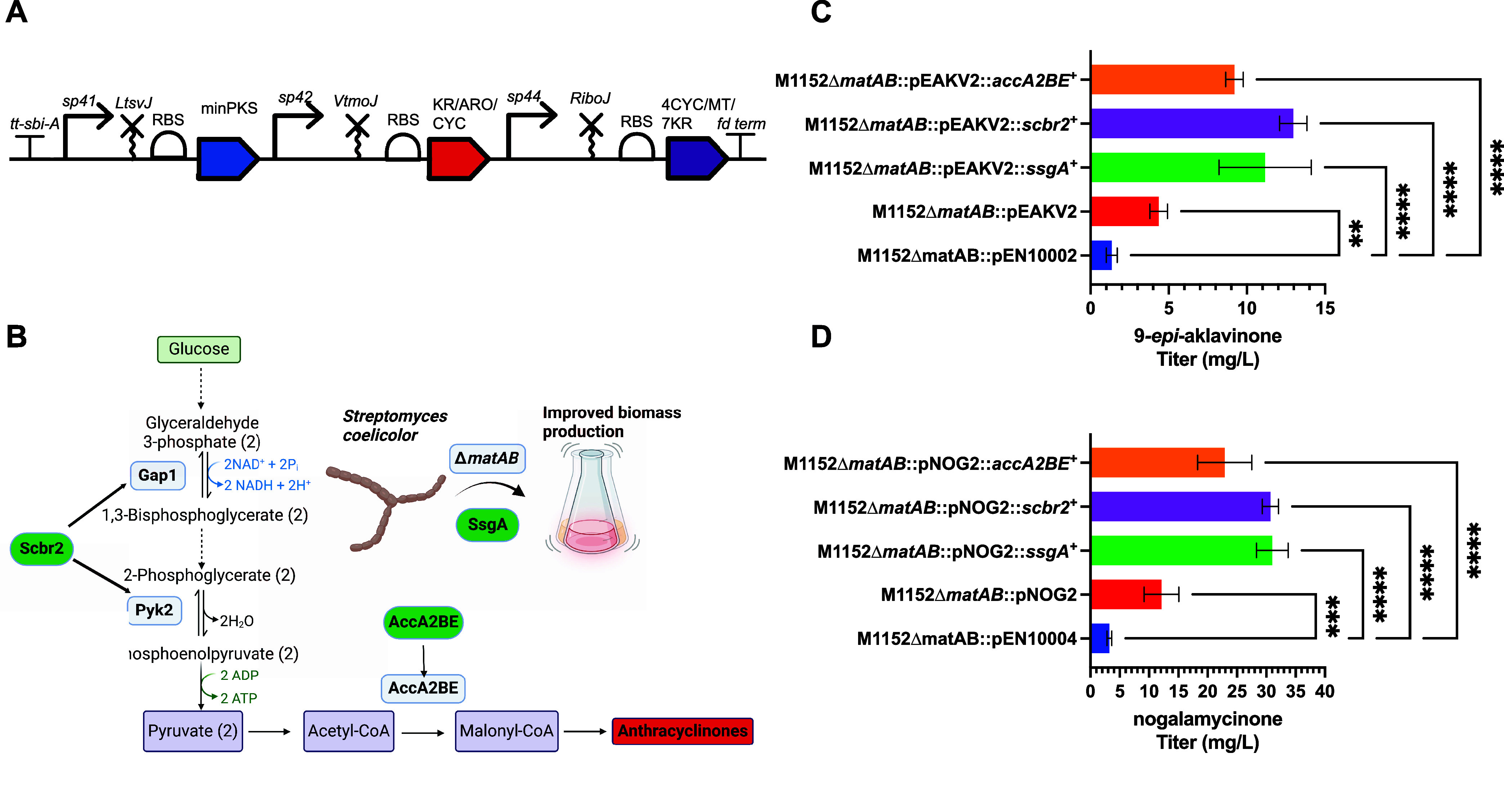
Four metabolic engineering strategies to increase the yields of anthracyclinones. (A) SBOL diagram of redesigned PKS cassettes. Constructs encoded the simultaneous expression of eight to ten genes (depending on the anthracyclinone) under the expression of strong *sp*41*, sp*42, and *sp*44 promoters. Constructs were insulated from external genomic promoter expression by incorporating *tt-sbi-A* and *fd-term* transcriptional terminators. Promoters were fused to ribozyme-insulator parts to stabilize the expression of the three operons within the construct. (B) Overexpression of *ssgA*, *scbr*2, and *accA*2*BE* for anthracyclinone enhancement. SsgA triggers sporulation and cell division, which works with the Δ*matAB* mutation to enhance biomass accumulation. Scbr2 is a pseudo-γ-butyrolactone response regulator that regulates glycolytic flux. AccA2BE enhances the supply of malonyl-CoA for anthracyclinone biosynthesis. (C) Production titers of 9-*epi*-aklavinone in strains expressing pEN10002 and/or coexpressing pEAKV2 with *scbr*2, *accA*2*BE*, and/or *ssgA*. (D) Production titers of nogalamycinone in strains expressing pEN10004 and/or coexpressing pNOG2 with *scbr*2*, accA*2*BE*, and/or *ssgA*.

The yields of **1**–**4** from the previous PKS cassettes ranged between 1 and 5 mg/L. We applied four distinct strategies to improve production (Figure 2A,B).^17^ First, we incorporated stronger synthetic *sp*41*, sp*42, and *sp*44 promoters (Table S1)^18,19^ together with ribozyme-based insulator parts (e.g., *sp*41*-*vtmoJ, *sp*42*-ltsvJ*, and *sp*44*-riboJ*) to eliminate interference between the promoters and ribosome initiation sites (Figure 2A).^6,20^ Ribozyme-based insulators function by cleaving the 5′ untranslated region (5′-UTR) of the mRNA to form a hairpin loop to stabilize the transcript. Furthermore, the cassettes were bracketed with transcriptional terminators (e.g., fd phage and *ttsbi-A* terminators)^21,22^ The new PKS cassettes were cloned into the pOSV802 vector, allowing single-copy chromosomal expression in *Streptomyces* (Table S1).^23^ Two vectors, pEAKV2 encoding the production of 9-*epi*-aklavinone and pNOG2 encoding the output of nogalamycinone, were expressed in *S. coelicolor* M1152Δ*matAB* (Table S2). The improved strains were fermented in E1 liquid media and produced 4 mg/L of **2** and 12 mg/L of **4**, which represented 1- and 6-fold improvement over the previous vectors pEN10002 and pEN10004, respectively (*p* < 0.001) (Figure 2C,D). The enhanced transcriptional stability of the constructs appeared to contribute to improved translation of the PKS machinery and metabolic flux to the target molecules.

Second, we increased substrate availability *via* acetyl-CoA carboxylase (e.g., *accA*2*BE*) overexpression (Figure 2B), previously employed with tetracenomycin engineering to achieve 3-fold yield enhancement.^24,25^ The acetyl-CoA carboxylase converts acetyl-CoA to malonyl-CoA, an essential precursor for the biosynthesis of polyketides.^26^ Integration of the pOSV808-*accA*2*BE* expression cassette into the *S. coelicolor* lines resulted in a 2-fold improvement in **2** (4.4 to 9.2 mg/L, *p* < 0.0001) and **4** (12.1 to 23 mg/L, *p* < 0.0001) (Figure 2C,D).

Third, we sought to increase yields by overexpressing *ssgA* from *Streptomyces griseus* (Figure 2B), which has been shown to suppress sporulation and enhance the fragmented growth of mycelia, thus resulting in faster growth kinetics and increased production of biomass and cell products.^27,28^ The overexpression of *ssgA* using the pENSV3 expression vector resulted in a nearly 3-fold increase in **2** (4.4 to 11.2 mg/L, *p* < 0.0001) and **4** (12.1 to 31.0 mg/L, *p* < 0.0001) production titers (Figure 2C,D). This result demonstrated that *ssgA* could improve anthracyclinone production titers.

Fourth, we overexpressed a pseudo-γ butyrolactone (GBL) receptor *scbr*2 in *S. coelicolor* M1152Δ*matAB*::cos16F4iE (Figure 2B). Scbr2 does not bind γ-butyrolactones but has been shown to interact with numerous endogenous and exogenous natural products.^29,30^ The regulatory effects of Scbr2 are mediated by promoting glycolysis *via* upregulation of glyceraldehyde-3-phosphate dehydrogenase (*gap*1) and pyruvate kinase (*pyk*2), which we reasoned could be important for carbon flow and growth kinetics.^31^ The *scbr*2 gene has been deleted in *S. coelicolor* M1152 as part of the *cpk* cluster,^32^ which has led to increased oxidative metabolism and oxidative stress (i.e., flux through the tricarboxylic acid cycle) based on a genome-scale metabolic model.^33^ To test this hypothesis, we overexpressed *scbr*2 in *S. coelicolor*, which resulted in a 3-fold improvement in **2** (4.4 to 13.0 mg/L, *p* < 0.0001) and **4** (12.1 to 30.0 mg/L, *p* < 0.0001) titers (Figure 2C,D). This result demonstrated that overexpression of *scbr*2 may have balanced glycolytic flux and relieved oxidative stress for improved product formation, but the exact mechanism requires further study.

### Metabolic Engineering of PKS Cassettes for 2-Hydroxylated Anthracyclinones

**Figure 3:**
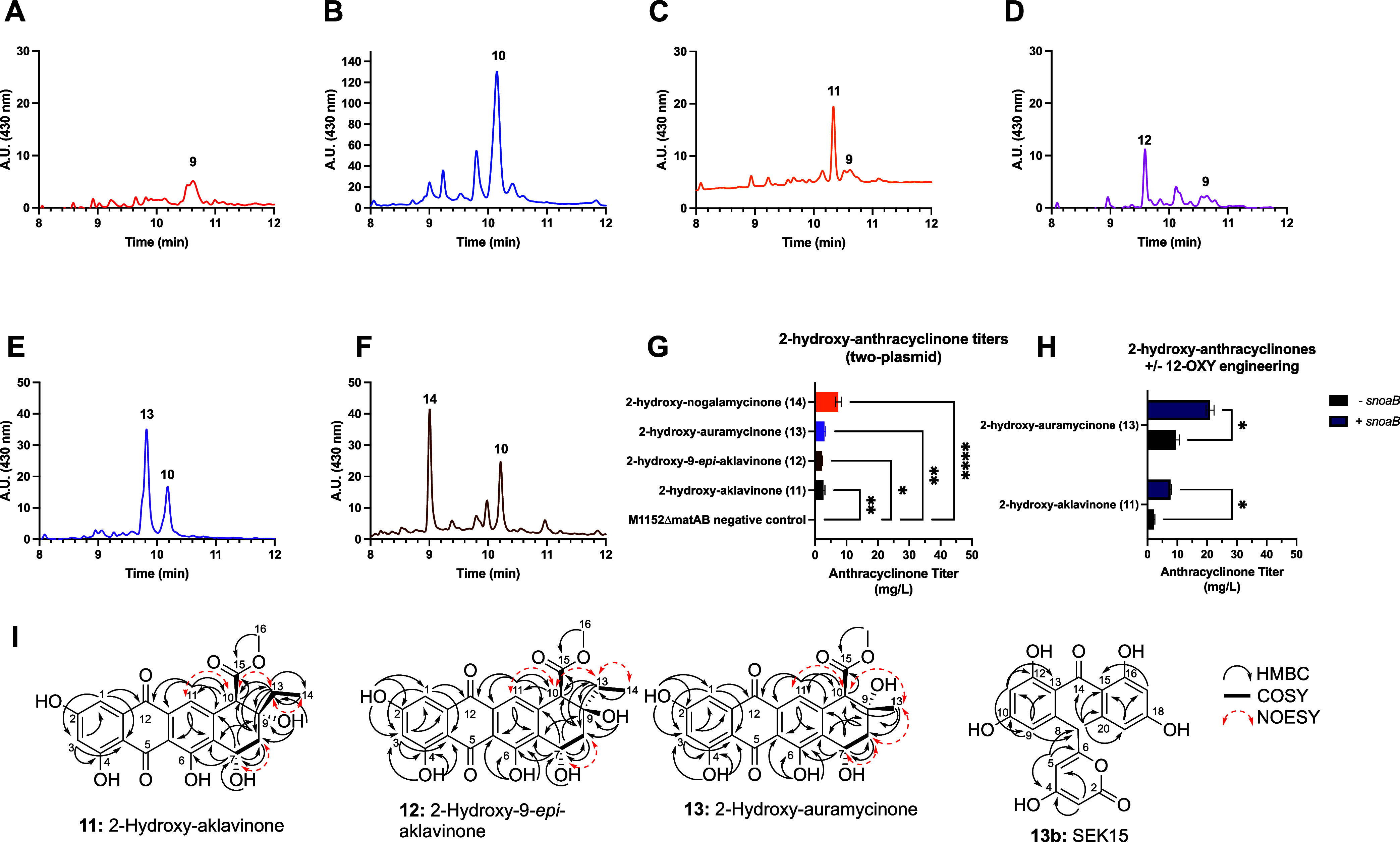
Engineering of aromatase/cyclase enzymes to biosynthesize 2-hydroxylated anthracyclinones. (A–F) HPLC-UV/vis chromatograms of different strains monitored at 430 nm engineered with two plasmids producing metabolites indicated with the numbered compound. (A) *S. coelicolor* M1152::*acc*::A2C1 (2-hydroxy-aklanonic acid, **9**); (B) *S. coelicolor* M1152::*acc*::S2C1 (2-hydroxy-nogalonic acid, **10**); (C) *S. coelicolor* M1152::*acc*::A2C1::A6 (2-hydroxy-aklavinone, **11**); (D) *S. coelicolor* M1152::*acc*::A2C1::S6 (2-hydroxy-*9-epi*-aklavinone, **12**); (E) *S. coelicolor* M1152::*acc*::S2C1::A6 (2-hydroxy-auramycinone, **13**); (F) *S. coelicolor* M1152::*acc*::S2C1::S6 (2-hydroxy-nogalamycinone, **14**). (G) Production titers of 2-hydroxylated anthracyclinones from strains engineered with two plasmids. (H) The production titers of strains engineered with and without expression of the C-12 oxygenase (*snoaB*). (I) The ^1^H,^13^C-HMBC, ^1^H,^1^H–COSY, and ^1^H,^1^H-NOESY two-dimensional NMR correlations for compounds **11, 12, 13**, and **13b**.

We sought to develop a means for producing 2-hydroxylated analogues of **1–4** by reprogramming the PKS cassettes.^6^ The polyketide-derived 2-hydroxyl group is removed during anthracycline biosynthesis by KR enzymes (Figure 1A) and, consistently, pathways lacking these redox enzymes have resulted in the production of 2-hydroxy-aklavinone^34^ and 2-hydroxy-nogalonic acid.^15,35^ Here, we reasoned that coexpression of anthracycline minPKS genes *aknBCDE*2*F* or *snoa*123 together with ARO and 2/3-CYC genes from nonreducing pathways,^36^ such as *mtmQY* involved in mithramycin biosynthesis, would result in the production of 2-hydroxy-aklanonic acid (**9**) or 2-hydroxy-nogalonic acid (**10**), respectively (Figure 1C). Cloning and transformation of gene cassettes pA2C1 (*aknBCDE*2*F* + *mtmQY*) and pS2C1 (*snoa*123 + *mtmQY*) resulted in the production of **9** and **10**, respectively (Figures 3A,B and S1–S2).

To extend the pathway, we cloned additional cassettes encoding MET, 4-CYC, and 7-KR (e.g., *aknGHU*) and pTG1-S6 (e.g., *snoaCLF*) to the host strain, which resulted in the 2-hydroxy-aklavinone (**11**), 2-hydroxy-9-*epi*-aklavinone (**12**), 2-hydroxy-auramycinone (**13**), and 2-hydroxy-nogalamycinone (**14**) based on high-performance liquid chromatography-mass spectrometry (HPLC-MS) (Figures 1C and 3C–F and Table S3). Although product yields were reasonable (3–10 mg/L) (Figure 3G), we hypothesized that additional coexpression of an OXY gene could enhance the folding and shaping of the unnatural 2-hydroxylated polyketides. Indeed, the coexpression of *snoaB* on a separate multicopy expression vector (pUWL201PW) under the control of the ermE*p resulted in a 50% increase in production of **11** and **13** (Figure 3H). Based on these findings, we refactored our constructs to generate improved versions of pHAKV2, pHEAKV2, pHAURA2, and pHNOG2 (e.g., encoding the production of **11, 12, 13**, and **14**, respectively, Table S1). pHAKV2, pHEAKV2, and pHAURA2 were used in subsequent scale-up fermentation experiments (see the Methods Section).

To confirm the structures of **11**, **12**, and **13**, the fermentations were scaled up in 5 L of E1 media, the metabolites were extracted and purified using various chromatographic techniques, and structurally characterized based on high-resolution electrospray ionization-MS (HRESI-MS) and NMR spectroscopy. The chemical characterization revealed that the early pathway intermediate SEK15 (**13b**) was accumulated as a side product in the scale-up fermentation of pHAURA2. In addition, three new 2-hydroxy-anthracyclinones (**11**–**13**) were isolated, based on one-dimensional (1D) (^1^H and ^13^C NMR) and two-dimensional (2D) (COSY, HSQC, HMBC, TOCSY, and NOESY) NMR spectroscopic measurements (Tables S4–S5 and Figures 3I and S3–S46). The molecular formulas of **11**–**13** were established as C_22_H_20_O_9_, C_22_H_20_O_9_, and C_21_H_18_O_9_ based on (+)-HRESIMS, with Δ*m*/*z* = 16 higher than those of aklavinone (**1**), 9-*epi*-aklavinone (**2**), and auramycinone (**3**), respectively, indicative of the presence of an extra oxygen atom in **11**–**13** (Figures S4, S17, and S27). Comparison of the NMR data (^1^H and ^13^C NMR) of the new compounds **11**-**13** with previously reported compounds **1**-**3** revealed that the main differences were observed in the aromatic ring A, where the trisubstituted aromatic rings in compounds **1**-**3** were converted to tetra-substituted aromatic rings in compounds **11**-**13** (with two *m*-coupled protons, H-1/H-3; Tables S4 and S5). The position of the hydroxy groups in compounds **11**-**13** was established to be at 2-position based on the observed HMBC correlations from H-1 to C-12/C-4a/CH-3; 2-OH to CH-1/C-2/CH-3 and H-3 to CH-1/C-4a (Figure 3I). All of the remaining 2D-NMR (^1^H, ^1^H–COSY, HMBC, TOCSY, and NOESY) correlations fully agree with structures **11**–**13** (Figure 3I and Supporting Information). As new natural products and are closely related to **1**–**3**, compounds **11**-**13** were designated as 2-hydroxy-aklavinone (**11**), 2-hydroxy-9-*epi*-aklavinone (**12**), and 2-hydroxy-auramycinone (2-hydroxy-9-*epi*-nogalamycinone; 2-hydroxy-9-*epi*-nogalavinone; **13**), respectively. The structure of **14** is suggested to be 2-hydroxy-nogalamycinone.

### Anthracyclinone 11-Hydroxylation by DnrF

To probe the substrate promiscuity of post-PKS tailoring enzymes for **1–4**, we carried out parallel investigations with purified enzymes and gene expression studies. We first cloned the *dnrF* gene^37^ from the doxorubicin pathway into the pBAD/His B vector for expression in *Escherichia coli* TOP10. After the production and purification of recombinant 11-hydroxylase DnrF, we assayed the conversion of **1–4** to the 11-hydroxylated species **15, 16, 17**, and **18** (Figures 1D and 4A). The incubation of **1** and **3** resulted in quantitative conversion to **15** and **17**, whereas **2** and **4** were converted to **16** and **18** with poor efficiency <5% (Figures 4B and S47–S75).

**Figure 4:**
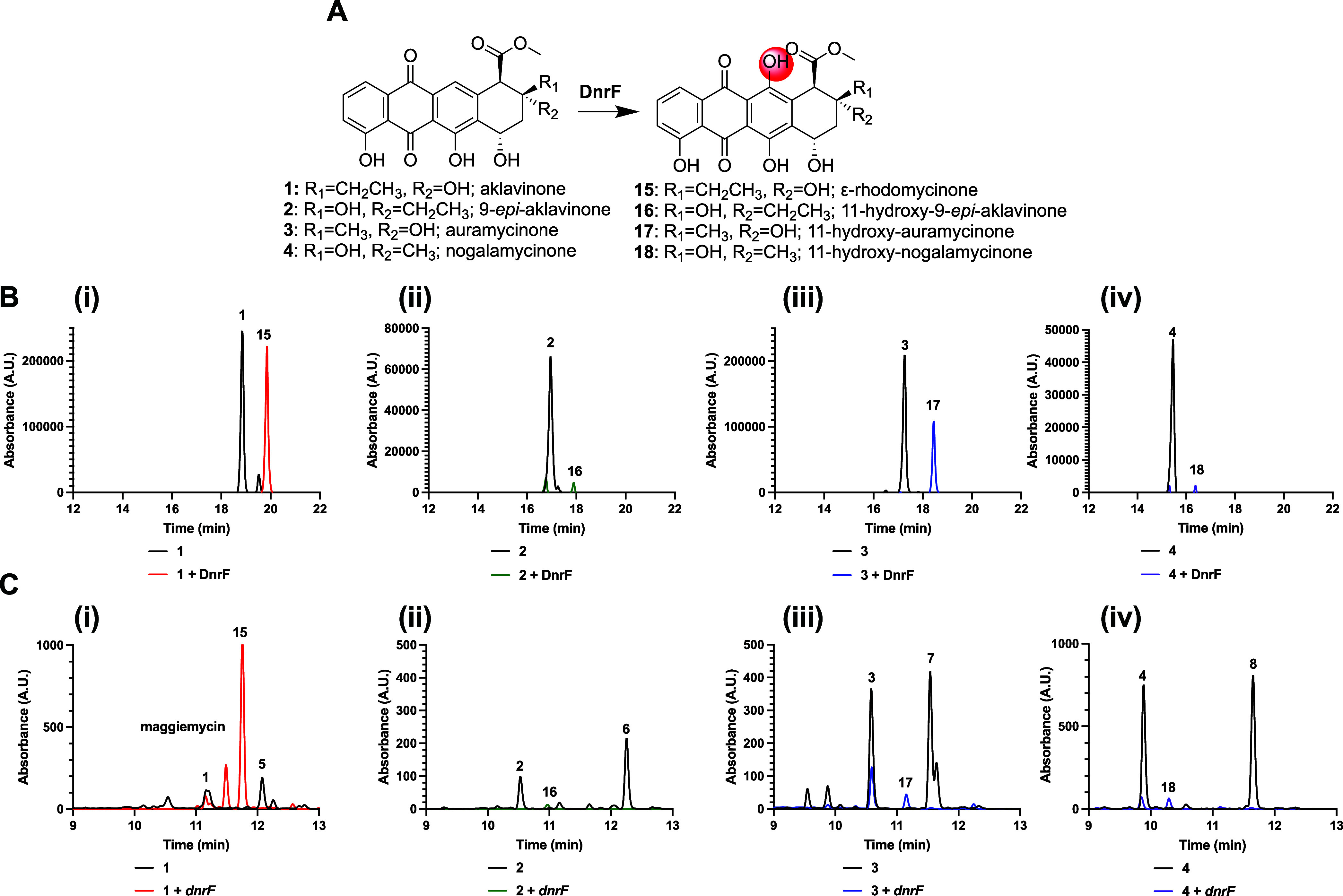
Enzymatic assays and metabolic engineering of 11-hydroxylated anthracyclinones. (A) DnrF catalyzes 11-hydroxylation of **1**–**4** to afford **15**–**18.** (B) HPLC-UV/vis traces at 490 nm of enzymatic reactions of **1–4** incubated with purified DnrF and no-enzyme controls. (C) HPLC-UV/vis traces at 490 nm of *S. coelicolor* lines engineered with expression constructs encoding **1–4** and *dnrF* or control lines producing only **1–4**.

For the *in vivo* expression experiments, *dnrF* was fused to the strong *gapdhpEL* promoter, cloned into expression vector pENSV3, and transformed into cell lines producing **1–4**. Analysis of culture extracts demonstrated that **1** was converted with >90% efficiency to **15** and maggiemycin, which is a shunt product derived from 11-hydroxylation of aklaviketone.^38^ Compound **3** was also converted in >90% efficiency to **17**, previously isolated from fermentations of *Streptomyces coeruleorubidis* ATCC 31276.^39^ However, similarly to the *in vitro* analyses, **2** and **4** were converted to **16** and **18** with poor 5–10% efficiency (Figures 4C and S47–S75), respectively. These results demonstrated that DnrF is more selective toward 9(*R*)-configured than 9(*S*)-configured anthracyclines.

### Anthracyclinone 10-Hydroxylation by RdmB and 10-Decarboxylation EamK

Both anthracycline 10-hydroxylation and 10-decarboxylation by the SAM-dependent hydroxylase RdmB and decarboxylase EamK, respectively, require initial 15-methylesterase activity (Figures 1E,F and 5A). Since the rhodomycin enzymes have a preference for glycosylated substrates,^11,40^ which is in contrast to the komodoquinone enzymes that convert aglycone substrates,^11^ we utilized the 15-methylesterase EamC in all experiments. Our *in vitro* (Figure 5B) and *in vivo* (Figure 5C) data were highly convergent and demonstrated that **1**–**4** were quantitatively converted by EamC and EamK to 10-decarboxylated products **19**–**22** (Figures S76–S91). In contrast, only **1** and **3** were converted to 10-hydroxylated species **23** and **24**, respectively (Figures 5B,C and S92–S97), which demonstrates that RdmB exhibits preference toward 9(*R*)-configured metabolites both *in vitro* and *in vivo*.

**Figure 5:**
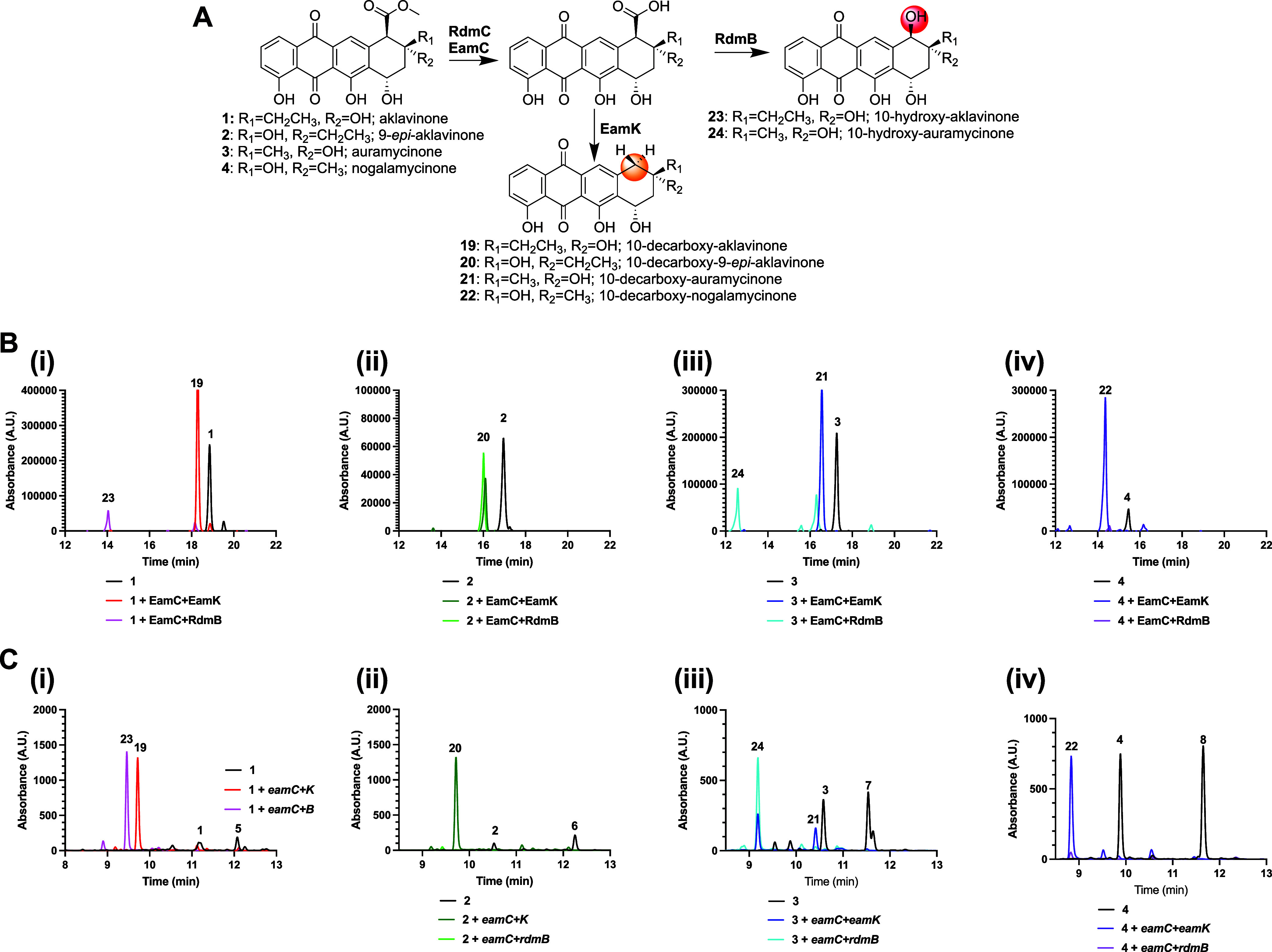
Enzymatic assays and metabolic engineering of 10-hydroxylated and 10-decarboxylated anthracyclinones. (A) EamC and EamK catalyze 10-decarboxylation of **1** – **4** to afford **19–22.** EamC and RdmB catalyze 10-hydroxylation of **1** and **3** to produce **23** and **24.** (B) HPLC-UV/vis traces at 430 nm of enzymatic reaction of **1–4** incubated with purified EamC + RdmB, EamC + EamK, and no-enzyme controls. (C) HPLC-UV/vis traces at 430 nm of *S. coelicolor* lines engineered with expression constructs encoding **1–4** and either *eamC* + *eamK* or *eamC* + *rdmB*.

### Anthracyclinone 10- and 11-Hydroxylation by RdmE, RdmC, and RdmB

**Figure 6:**
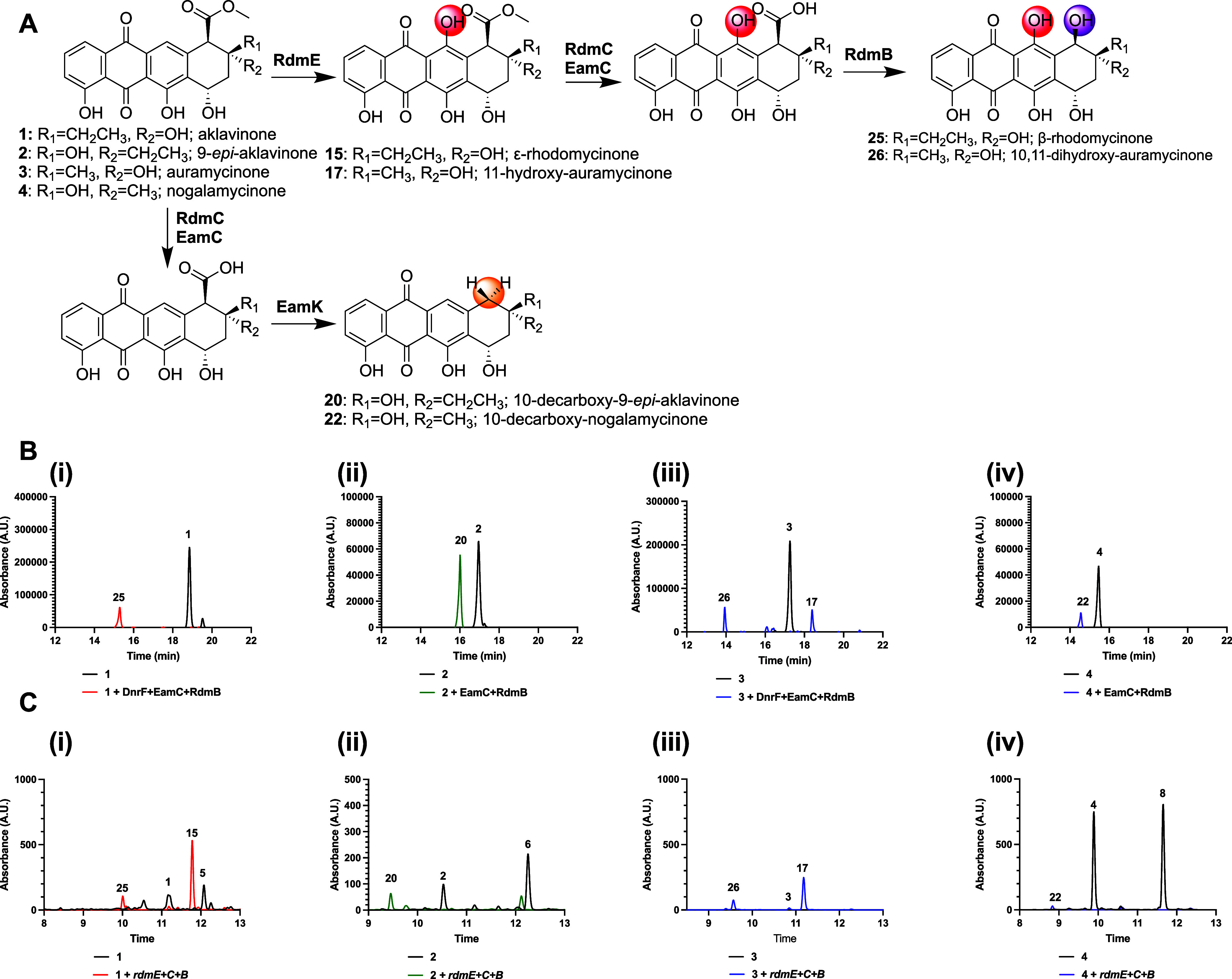
Enzymatic assays and metabolic engineering of 10-hydroxylated and 11-hydroxylated anthracyclinones. (A) RdmE, EamC/RdmC, and RdmB catalyze 11-hydroxylation and 10-hydroxylation of **1** and **4** to generate **25** and **26**, respectively. (B) HPLC-UV/vis traces at 490 nm of enzymatic reaction of **1** and **3** incubated with purified DnrF, EamC, RdmB, and no-enzyme controls. (C) HPLC-UV/vis traces at 490 nm of *S. coelicolor* lines engineered with expression constructs encoding **1** and **3** and *rdmE* + *rdmC* + *rdmB* and control lines producing **1** and **3**.

We next sought to incorporate the tailoring steps for concomitant 10 and 11-hydroxylation by the entire RdmE, RdmC, and RdmB reaction cascade (Figures 1E and 6A). We incubated **1**–**4** with purified DnrF, EamC, and RdmB, which resulted in the conversion of substrates **1** and **3** to **25** and **26** (Figures 6B and S98–S104). Similarly, **1** and **3** were converted to **25** and **26** in *Streptomyces* strains coexpressing the appropriate PKS cassettes and a cassette expressing *rdmE, rdmC*, and *rdmB* (Figures 6C and S98–S104). Both *in vitro* and *in vivo*, the substrates **2** and **4** underwent 10-decarboxylation toward **20** and **22** as the primary metabolic route and were not processed by the hydroxylating enzymes. The production titers of **25** and **26** from the engineered strains in SG-TES media were 30.3 ± 10 and 9.3 ± 4 mg/L, respectively (Figure S105). Altogether, this indicates robust production of the β-rhodomycinone analogues **25** and **26** in the engineered microorganisms.

### Anthracyclinone 1-Hydroxylation by KstA15 and KstA16

**Figure 7:**
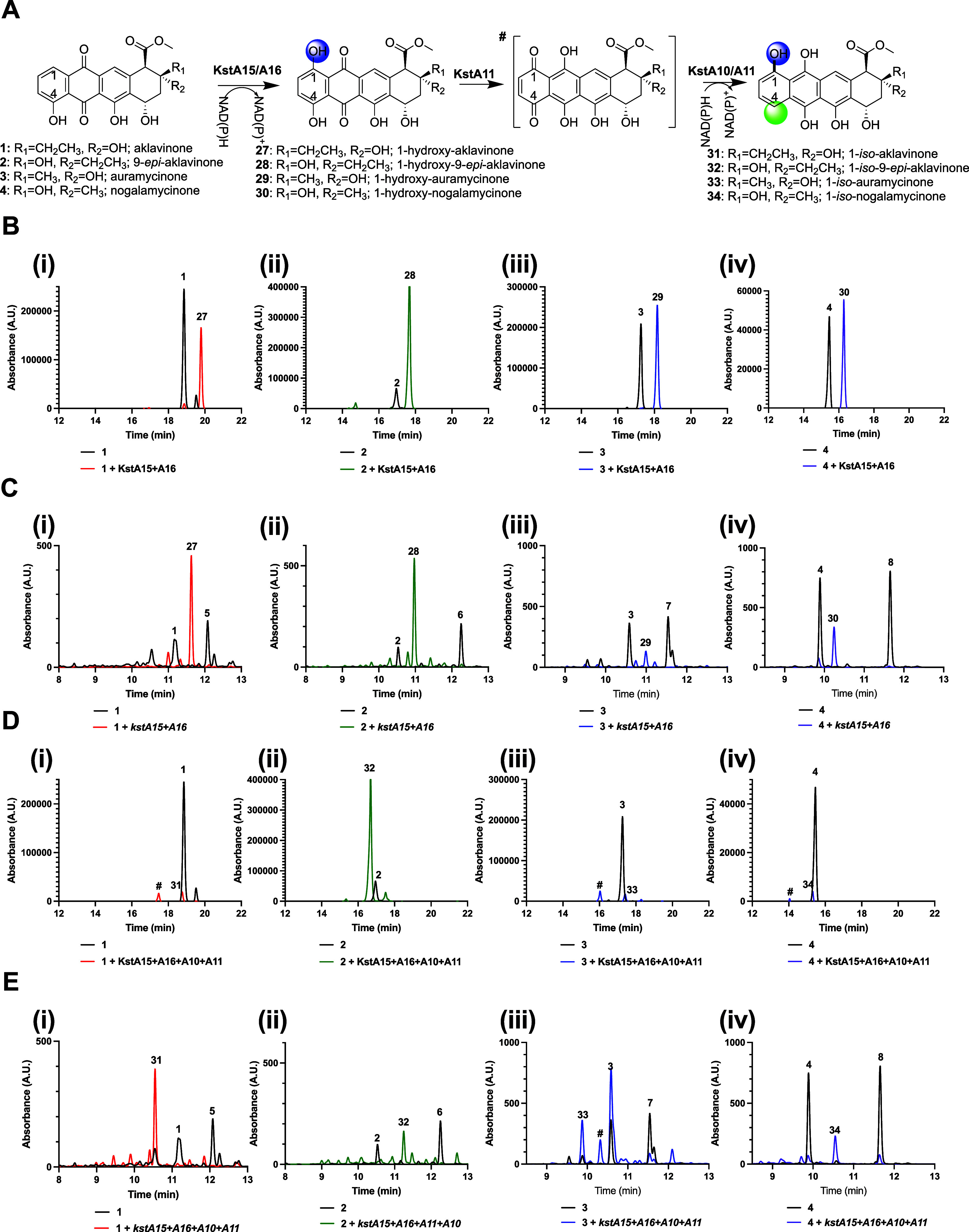
Enzymatic assays and metabolic engineering of 1-hydroxylated and 4-regioisomerized anthracyclinones. (A) KstA15 and KstA16 catalyze 1-hydroxylation of **1–4** yielding **27–30**, KstA10 and KstA11 carry out asymmetric reduction and dearomatization, followed by a region-specific reduction and dehydration yielding **31–34**. (B) HPLC-UV/vis traces of enzymatic reaction of **1–4** incubated with purified KstA15 and KstA16 and no-enzyme controls. (C) HPLC-UV/vis traces of *S. coelicolor* lines engineered with expression constructs encoding **1–4** and *kstA*15 and *kstA*16 and control lines producing **1–4**. (D) HPLC-UV/vis traces of enzymatic reaction of **1–4** incubated with purified KstA15, KstA16, KstA11, and KstA10 and no-enzyme controls. (E) HPLC-UV/vis traces of *S. coelicolor* lines engineered with expression constructs encoding **1–4** and *kstA*15*, kstA*16*, kstA*10, and *kstA*11 and control lines producing **1–4**.

We next utilized the two-component monooxygenase system, KstA15 and KstA16, from the kosinostatin pathway for 1-hydroxylation (Figures 1F and 7A).^13^ KstA15 is a polyketide cyclase-like enzyme,^41^ while KstA16 belongs to short-chain alcohol dehydrogenases.^42^ The enzyme assays with substrates **1–4** resulted in the quantitative conversion to 1-hydroxylated anthracyclinones **27**–**30** (Figures 7B and S106–S120). Analogously, the heterologous expression of a cassette encoding *kstA*15 and *kstA*16 resulted in the production of 1-hydroxylated species **27–30** (Figures 7C and S106–S120). The compounds **27**, **29**, and **30** have been generated in previous studies,^41−43^ but the anthracyclinone **28** has not been reported in the literature. The 1-hydroxylation mechanism is a necessary modification for 1-*O-*glycosylation of anthracyclines, such as that occurs with the nogalamycin family of compounds.^44^

### Anthracyclinone 4-Regioisomerization by KstA15, KstA16, KstA11, and KstA10

KstA15 and KstA16 are part of a four-enzyme cascade to generate a hydroxy regioisomerized anthracyclinone. Following the successful 1-hydroxylation by KstA15 and KstA16, we reconstituted the entire 4-regioisomerization pathway by incorporating KstA11 and KstA10 (Figures 1F and 7A).^13^ The *in vitro* assays with the four enzymes converted **1–4** to *iso*-anthracyclinones **31–34** (Figures 7D and S121–S132). Several putative intermediates were detected in the enzymatic reactions, including the dearomatized 1,4-diketone species (indicated with #) (Figure 7D). Similarly, coexpression of the four *kst* genes in appropriate *S. coelicolor* strains producing **1–4** led to accumulation of **31–34** (Figures 7E and S121–S132) in yields ranging from 8 to 30 mg/L (Figure S133). Within the ESI-MS single ion monitoring traces, the parental substrates, unknown intermediates (marked *), and products **31–34** were detected with the expected mass ions (Table S3). These results indicate that the kosinostatin enzymes are remarkably flexible toward 9(*R*) and 9(*S*)-configured anthracyclinones. Previously, **31** has been isolated from strains engineered for isoanthracycline production^45^ and **33** is the native substrate of the kosinostatin pathway,^13^ but compounds **32** and **34** have not been reported to date.

## Human Cancer Cell Viability Assays

To investigate the potential anticancer activity of the anthracyclinone extracts, the crude extracts were normalized to 40 μg/mL concentration and were tested in a panel of human cancer cells: A549 (nonsmall cell lung), PC3 (prostate), TC32 (Ewing sarcoma), and HCT116 (colorectal) human cancer cell lines (Table 1 and Figure S134). As previously reported, the anthracyclinone aglycones **3** and **4** are inactive at IC_50_ values >30 μM in A549, PC3, MKL1, and MCC26 cancer cell lines, though **1** is slightly more active in PC3 cells at 7 μM IC_50_ and A549 cells at 17 μM, respectively.^6^ Most of the extracts were inactive against these cancer cell lines; however, extracts from five engineered lines containing compounds **15**, **17**, **18**, **22**, **25**, **26**, and **30** exhibited substantive cytotoxic activity in the cell lines tested (<20% cell viability T/C) (Figures S134). Compounds **11**, **12**, and **13** were inactive at concentrations of 3 μM (Figure S135), which indicated that 2-hydroxylation does not enhance the cytotoxicity of these aglycones.

**Table 1:**
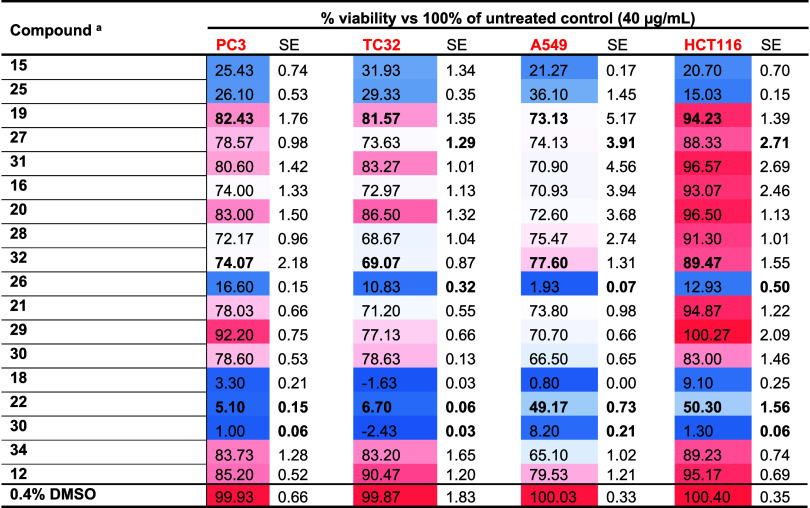
Cytotoxic Activities of the Generated Strain Extracts in PC3, TC32, A549, and HCT116 Cell Lines^b^^,^^c^

a The samples were culture extracts from the strains producing the numbered compound and contain other impurities. Actinomycin D and H_2_O_2_ were used as positive controls at 20 μM and 1 mM concentration, respectively (0% viable cells, *n* = 3. SE = standard error).

b The cell viability of each line was determined as the percentage of cell viability relative to untreated controls.

c % Viability values were obtained after 72 h incubation.

Anthracyclines are considered to require the glycoside moiety for binding to DNA and inhibition of DNA topoisomerases.^5^ Therefore, the significant cytotoxicity against the human cancer cell lines in selected extracts can be considered to be surprising (Table 1). The data provides guidance regarding the rational engineering of anthracyclines toward 11-hydroxylated, 10,11-dihydroxylated, or 1-hydroxylated nogalamycinone derivatives. Indeed, anthracyclines with C-1 or C-11 hydroxyl groups were shown to have increased potency in L1210 leukemia cells,^46^ while 11-hydroxylation of aclacinomycins resulted in markedly improved cytotoxicity in the NCI 60-cell line assay.^47^ Rhodomycin A, which is a 10,11-dihydroxylated anthracycline, was recently identified as an efficient antagonist for knocking down Src-associated oncogenes.^48^

## Conclusions

Anthracyclines have been a cornerstone of anticancer chemotherapy for several decades. Despite the success of these molecules, severe side effects have made the continued exploration of the chemical space around anthracyclines necessary for discovering improved congeners. For example, developing the semisynthetic idarubicin (4-demethoxy daunorubicin) for acute myeloid leukemia has demonstrated the importance of C–H functionalization of anthracyclines.^49^ However, the regiospecific modification of the polyaromatic anthracycline core has remained challenging using organic synthesis. This is in contrast to the biosynthesis of anthracyclines in *Streptomyces* bacteria, where several enzymatic systems have evolved for C–H functionalization in the various natural metabolic pathways.

**Figure 8:**
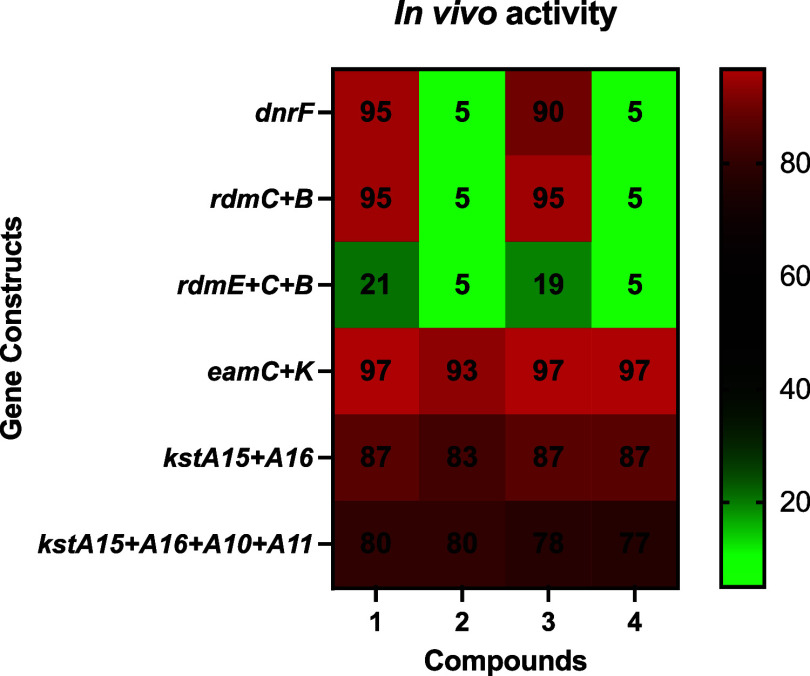
Heat map of the *in vivo* activity of the tailoring gene constructs utilized in this study when coexpressed with anthracyclinone scaffolds **1–4.**

We have used our BioBricks metabolic engineering platform to systematically probe 1-, 2-, 10-, and 11-hydroxylations, 10-decarboxylation, and 4-hydroxyl regioisomerization. For the first time, we analyzed the functionality of 10 tailoring genes with the four possible anthracyclinone core structures **1**–**4** comparatively. The results demonstrate that the *in vivo* activity remained consistently high in 10-decarboxylation, 1-hydroxylation, and 4-hydroxyl regioisomerization (Figure 8). In contrast, 10-hydroxylation and 11-hydroxylation were dependent on the 9*R*-stereochemistry but were tolerant toward both methyl and ethyl side chains at the same location. The dual 10- and 11-hydroxylations proved the most challenging, which may be because the 10-hydroxylase RdmB generally prefers glycosylated substrates (Figure 8).

Our work demonstrates that tailoring steps in anthracycline biosynthesis are well suited for combinatorial biosynthesis for increasing the chemical diversity of natural products. We utilized genetic material from five distinct pathways and their use in combination with the four aglycone possibilities led to the generation of nine novel anthracyclinones (**11, 12, 13, 14, 16, 18, 24, 26, 28**). It is noteworthy that even though all of the tailoring enzymes have been extensively studied in the past, including by structural analysis at atomic resolution, it was not possible to predict *a priori* which gene combinations resulted in new functional metabolic pathways. Therefore, the ability to use multiplasmid expression systems in a robust heterologous host in combination with an expanding modular BioBricks library is essential to allow high-throughput combinatorial work to compensate for the limitations of an unpredictable design space. In addition, the highly concurrent *in vitro* and *in vivo* data indicate that combinatorial enzymatic synthesis may be an efficient preliminary tool to guide metabolic engineering projects. Notably, the human cancer cell line viability assays (Table 1) provide further direction for the rational engineering of anthracyclines based on the **15**, **17, 25**, and **26** scaffolds. These strains will serve as valuable chassis for combinatorial biosynthesis of TDP-deoxysugar pathways to develop “new to nature” anthracyclines, which could be developed into potent antitumoral metabolites.

## Methods

### Bacterial Strains and Growth Conditions

*E. coli* TOP10 and *E. coli* ET12567 were grown in LB broth or LB agar at 37 °C as previously described.^50^*E. coli* TOP10 was used for plasmid propagation, subcloning, and enzyme expression. Enzymes were cloned into pBAD/His B vector (Invitrogen) and enzymes were expressed using the arabinose-inducible promoter.^51^*E. coli* ET12567/pUZ8002 was used as the conjugation donor host for mobilizing expression vectors into *S. coelicolor* as previously described.^52^ When appropriate, ampicillin (100 μg mL^–1^), kanamycin (25 μg mL^–1^), apramycin (25 μg mL^–1^), viomycin (25 μg mL^–1^), spectinomycin (100 μg mL^–1^), hygromycin (50 μg mL^–1^), and nalidixic acid (30 μg mL^–1^) were supplemented to media to select for recombinant microorganisms.

*S. coelicolor* derivative strains were routinely maintained on Soya-Mannitol Flour (SFM) agar supplemented with 10 mM MgCl2 and International Streptomyces Project medium #4 (ISP4) (BD Difco) at 30 °C as described previously.^52^ For liquid culturing, *S. coelicolor* derivative strains were grown in TSB media (3 mL) to ferment the seed culture and then grown in a modified 50 mL SG-TES liquid medium (soytone 10 g, glucose 20 g, yeast extract 5 g, TES free acid 5.73 g, CoCl_2_ 1 mg, per liter) or 50 mL E1 medium for production for four to 5 days.^53^ All media and reagents were purchased from Thermo Fisher Scientific.

### Molecular Biology Procedures

Routine genetic cloning and plasmid manipulation were carried out in *E. coli* DH10B cells (New England Biolabs). *E. coli* ET12567/pUZ8002 was used as the host for intergeneric conjugation with *S. coelicolor* as previously described.^52^*E. coli* chemically competent cells were prepared using the Mix and Go! *E. coli* Transformation Kit (Zymo Research). *E. coli* was transformed with plasmid DNA *via* chemically competent heat-shock transformation as described previously. Plasmid DNA was isolated *via* the Wizard Plus SV Minipreps DNA Purification System by following the manufacturer’s protocols (Promega). All molecular biology reagents and enzymes used for plasmid construction were purchased from New England Biolabs. The conjugation donor host *E. coli* ET12567/pUZ8002 was transformed with constructs for mobilization into *S. coelicolor* strains, as previously described. For each transformation, 9–12 independent exconjugants were plated to DNA plates supplemented with antibiotics and grown for 4–5 days until the formation of vegetative mycelium.

### Preparation of *In Vitro* Samples and *In Vivo* Samples for HPLC-MS Analysis

The in vitro samples’ conditions for protein expression, purification, and enzyme assays are described in the Supporting Information (Supporting Methods 1, 2, and 3). All reactions were checked by UHPLC (Shimadzu Nexera LC-40 system with a diode array detector set to 430 and 490 nm wavelengths) using a Phenomenex Kinetex Phenyl-Hexyl column (2.6 μm, 100 Å, 4.6 mm × 100 mm). Method: Solvent A: 15% CH3CN/0.1% FA; solvent B: CH_3_CN; flow rate: 0.5 mL/min; 0–2 min, 0% B; 2–20 min, 0–40% B; 20–24 min, 100% B; 24–29 min, 0% B.

For each *in vivo* experiment, four to six biological replicates were grown in 50 mL of SG-TES liquid media in baffled Erlenmeyer flasks as previously described.^6,24,25^ The shake flask fermentations were grown in an orbital shaker for 5 days at 30 °C at 200 rpm. The entire cultures were extracted with 3 volumes of 0.1% formic acid in ethyl acetate and the extracts were dried down *in vacuo*. The extracts were resuspended in 4 mL of methanol, filtered in a 0.5 μm nylon syringe filter, and 10 μL was analyzed *via* HPLC-MS.

The analysis of anthracyclinones was carried out on an Agilent 1260 Infinity II LC/MSD iQ single quadrupole instrument. In brief, 10 μL of the sample was injected *via* an autosampler onto the sample loop, separated on a Poroshell 120 Phenyl-Hexyl Column (ID 2.7 μm, 4.6 mm × 100 mm), and analyzed in gradients of solvent A (0.1% formic acid in water) and solvent B (0.1% formic acid in acetonitrile). The HPLC program used a constant flow rate of 0.5 mL per minute and the following gradient steps: 0 min, 95% solvent A and 5% solvent B; 0–10 min, 95% solvent A and 5% solvent B to 5% solvent A and 95% solvent B; 10–13 min, held at 5% solvent A and 95% solvent B; 13.1 min, reequilibrate to 95% solvent A and 5% solvent B; 13.1–15.1 min, 95% solvent A and 5% solvent B. The diode array detector (DAD) was set to monitor UV/vis absorbance at 430 and 490 nm. The ESI-MS was set to scan from 200 *m*/*z*–500 *m*/*z* fragments in positive and negative ionization modes.

The yields were determined by comparing them to authenticated external standard curves of **1** and **4** analyzed *via* HPLC-MS. The conversion percentages were determined by measuring the area under the curve of the anthracyclinone metabolites at 430 and 490 nm wavelengths.

### General Experimental Procedures

Ultraviolet–visible (UV–vis) spectra were taken directly from analytical HPLC runs and show relative intensities. The NMR spectra were recorded on a Bruker Avance NEO 400 MHz (Bruker BioSpin Corporation, Billerica, MA) (^1^H, 400.13 MHz; ^13^C, 100.25 MHz), Varian 500 MHz (Agilent, Santa Clara, CA) (^1^H, 500 MHz; ^13^C, 125.7 MHz), and/or Bruker Avance NEO 600 MHz NMR (^1^H, 600.37 MHz; ^13^C, 150.96 MHz) spectrometer, equipped with triple-channel TCI 5 mm cryoprobe (all spectra were processed using Bruker Topspin 4.1.4 version, and 2D spectra were apodized with QSINE or SINE window functions and zero-filled to (2048 × 1024 points)). All of the spectra were analyzed and plotted using Mnova [where δ-values were referenced to respective solvent signals CD_3_OD, δ_H_ 3.31 ppm, δ_C_ 49.15 ppm; DMSO-*d*_6_, δ_H_ 2.50 ppm, δ_C_ 39.51 ppm]. High-resolution electrospray ionization (HRESI) mass spectra were recorded on the AB SCIEX Triple TOF 5600 system (AB Sciex, Framingham, MA). HPLC-UV/MS analyses were accomplished with an Agilent InfinityLab LC/MSD mass spectrometer (MS Model G6125B; Agilent Technologies, Santa Clara, CA) equipped with an Agilent 1260 Infinity II Series Quaternary LC system and a Phenomenex NX-C18 column (250 mm × 4.6 mm, 5 μm; Phenomenex, Torrance, CA) [**Method A:** solvent A: H_2_O/0.1% formic acid, solvent B: CH_3_CN; flow rate: 0.5 mL min^–1^; 0–30 min, 5–100% B (linear gradient); 30–35 min, 100% B; 35–36 min, 100%–5% B; 36–40 min, 5% B]. HPLC-UV analyses were carried out in an Agilent 1260 system equipped with a photodiode array detector (PDA) and a Phenomenex C_18_ column (250 mm × 4.6 mm, 5 μm; Phenomenex, Torrance, CA) [**Method B:** solvent A: H_2_O/0.1% TFA, solvent B: CH_3_CN; flow rate: 1.0 mL min^–1^; 0–30 min, 5–100% B; 30–35 min, 100% B; 35–36 min, 100–5% B; 36–40 min, 5% B]. *Semi*-preparative HPLC were carried out in a Agilent 1260 Infinity II (Prep HPLC) system equipped with a Diode Array Detector (DAD) and a Gemini 5 μm C_18_ 110 Å, LC column 250 mm × 10 mm (Phenomenex, Torrance, CA) [**Method C:** solvent A: H_2_O/0.025% TFA; solvent B: CH_3_CN; flow rate: 5.0 mL min^–1^; 0–3 min, 25% B; 3–10 min, 25–75% B; 10–16 min, 75–100% B; 16–18 min, 100% B; 18–19 min, 100–25% B; 19–20 min, 25% B]; [**Method D:** solvent A: H_2_O/0.025% TFA; solvent B: CH_3_CN; flow rate: 5.0 mL min^–1^; 0–3 min, 25% B; 3–17 min, 25–100% B; 17–18 min, 100% B; 18–19 min, 100–25% B; 19–20 min, 25% B]; [**Method E:** solvent A: H_2_O/0.025% TFA; solvent B: CH_3_CN; flow rate: 5.0 mL min^–1^; 0–3 min, 25% B; 3–17 min, 25–100% B; 17–22 min, 100% B; 22–23 min, 100–25% B; 23–27 min, 25% B]. All solvents used were of ACS grade and purchased from Pharmco-AAPER (Brookfield, CT). Size exclusion chromatography was performed on Sephadex LH-20 (25–100 μm; GE Healthcare, Piscataway, NJ). A549, PC3, and HCT116 cells were obtained from ATCC (Manassas, VA). All other reagents used were of reagent grade and purchased from Sigma-Aldrich (Saint Louis, MO), unless otherwise noted.

### Purification of Compounds **11**–**13** and **13b**

The reddish-brown oily crude extract (3.82 g) produced by **Strain 1** [*S. coelicolor* M1152ΔmatAB::pSET154BB-kasOp*-snoa123-kasOp*-mtmQY-sp44-aknGHU] was dissolved in MeOH (10 mL) followed by Sephadex LH-20 (MeOH; 2.5 cm × 50 cm) mentored by TLC to afford six fractions. LC-MS analysis indicates that target metabolite **13** was mainly detected in fractions F3 and F4. *Semi*-prep-HPLC purification (**Method C**) of F3 and F4 afforded compound **13** (2-hydroxy-auramycinone; 24.4 mg) in pure form as red solid, and compound **13b** (SEK15; 8.5 mg) in pure form as pale-yellow solid.

The reddish-brown oily crude extract (3.20 g) produced by **Strain 2** [*S. coelicolorM*1152Δ*matAB*::*pHEAKV*2] was fractionated using silica gel column (DCM/0–50% MeOH; 2.5 cm × 30 cm) to afford six fractions F1 (DCM; 0.5 L), F2 (DCM/2% MeOH; 0.5 L), F3 (DCM/4% MeOH; 0.5 L), F4 (DCM/10% MeOH; 0.5 L), F5 (DCM/20% MeOH; 0.5 L), and F6 (DCM/50% MeOH; 0.5 L), followed by LC-MS analysis. The target compound was detected in F3 and F4. Fractions F3–F4 were combined followed by *semi*-prep-HPLC purification (**Method D**) to afford compound **12** (2-hydroxy-9-*epi*-aklavinone; 4.2 mg) in pure form as red solid.

In the same manner, the crude extract (2.22 g, reddish-brown oily) produced by **Strain 3** [*S. coelicolorM*1152Δ*matAB::*pSET-A2M1A6] was fractionated using silica gel column (DCM/0–50% MeOH; 2.0 cm × 25 cm) to yielded six fractions F1 (DCM; 0.5 L), F2 (DCM/2% MeOH; 0.5 L), F3 (DCM/4% MeOH; 0.5 L), F4 (DCM/10% MeOH; 0.5 L), F5 (DCM/20% MeOH; 0.5 L), and F6 (DCM/50% MeOH; 0.5 L). LC-MS analysis of the obtained fractions indicates that the target compound was detected in fractions F2–4, however, with low yield. Fractions F2–4 have been combined, dissolved in MeOH (2 mL) followed by Sephadex LH-20 (1 cm × 40 cm; MeOH) and *semi*-prep-HPLC purification (**Method E**) to give compound **11** (2-hydroxy-aklavinone; 1.56 mg) in pure form as red solid.

### Statistical Analyses

The statistical significance of the impact of genetic manipulations and combinatorially assessed variables on production was assessed *via* post hoc analysis. One-way ANOVA, two-way ANOVA, and Student’s *t* test analyses were performed using GraphPad Prism version 10.2.1 for Mac OS X, GraphPad Software, San Diego, CA, www.graphpad.com.

### Cancer Cell Line Viability Assay

Mammalian cell line cytotoxicity [A549 (nonsmall cell lung) and PC3 (prostate), TC32 (Ewing sarcoma), and HCT116 (colorectal) human cancer cell lines] assays were accomplished in triplicate following our previously reported protocols.^24,54−57^ Actinomycin D (A549 and PC3) was used as positive controls.

### Physicochemical Properties of Compounds **11**–**13**

#### 2-Hydroxy-aklavinone (**11)**

C_22_H_20_O_9_ (428); red solid; HPLC-*R*_t_ = 24.72 min (Supporting Information, Figures S9–S21); UV/vis λ_max_ 228, 250 (sh), 268, 290 (sh), 445 nm; ^1^H NMR (DMSO-*d*_6_, 600 MHz) and ^13^C NMR (DMSO-*d*_6_, 150 MHz), see Tables S1 and S2; (−)**-**ESI-MS: *m*/*z* 427 [M – H]^−^; (+)-ESI-MS: *m*/*z* 411 [(M-H_2_O) + H]^+^, 393 [(M-H_2_O) + H]^+^; (+)**-**HRESI-MS: *m*/*z* 393.0942 [(M-2H_2_O) + H]^+^ (calcd for C_22_H_17_O_7_, 393.0969), 879.1973 [2M + Na]^+^ (calcd for C_44_H_40_O_18_Na, 879.2106).

##### 2-Hydroxy-9-*epi*-aklavinone (**12**)

C_22_H_20_O_9_ (428); red solid; HPLC-*R*_t_ = 22.47 min (Supporting Information, Figures S22–S31); UV/vis λ_max_ 228, 268, 290 (sh), 445 nm; ^1^H NMR (DMSO-*d*_6_, 600 MHz) and ^13^C NMR (DMSO-*d*_6_, 150 MHz), see Tables S1 and S2; (−)**-**ESI-MS: *m*/*z* 427 [M – H]^−^; (+)-ESI-MS: *m*/*z* 393 [(M-2H_2_O) + H]^+^, 879 [2M + Na]^+^; (+)**-**HRESI-MS: *m*/*z* 393.0958 [(M-2H_2_O) + H]^+^ (calcd for C_22_H_17_O_7_, 393.0969), 451.0986 [M + Na]^+^ (calcd for C_22_H_20_O_9_Na, 451.0999), 879.2020 [2M + Na]^+^ (calcd for C_44_H_40_O_18_Na, 879.2106).

##### 2-Hydroxy-auramycinone (2-Hydroxy-9-*epi*-nogalamycinone; 2-Hydroxy-9-*epi*-nogalavinone; **13**)

C_21_H_18_O_9_ (414); red solid; HPLC-*R*_t_ = 30.39 min (Supporting Information, Figures S32–S40); UV/vis λ_max_ 228, 258, 270, 290, 430 nm; ^1^H NMR (DMSO-*d*_6_, 600 MHz) and ^13^C NMR (DMSO-*d*_6_, 150 MHz), see Tables S1 and S2; (−)**-**ESI-MS: *m*/*z* 413 [M – H]^−^; (+)-ESI-MS: *m*/*z* 397 [(M-H_2_O) + H]^+^, 379 [(M-2H_2_O) + H]^+^, 851 [2M + Na]^+^; (+)**-**HRESI-MS: *m*/*z* 379.0806 [(M-2H_2_O) + H]^+^ (calcd for C_21_H_15_O_7_, 379.0812), 851.1746 [2M + Na]^+^ (calcd for C_42_H_36_O_18_Na, 851.1793).

##### SEK15 (**13b**)

C_20_H_16_O_8_ (384); pale-yellow solid; HPLC-*R*_t_ = 26.81 min (Supporting Information, Figures S42–S51); UV/vis λ_max_ 210, 290, 320 (sh) nm; ^1^H NMR (CD_3_OD, 600 MHz) and ^13^C NMR (CD_3_OD, 150 MHz), see Tables S1 and S2; (−)**-**ESI-MS: *m*/*z* 383 [M – H]^−^, 767 [2M – H]^−^; (+)-ESI-MS: *m*/*z* 385 [M + H]^+^; (+)**-**HRESI-MS: *m*/*z* 385.0893 [M + H]^+^ (calcd for C_20_H_17_O_8_, 385.0918), 407.0679 [M + Na]^+^ (calcd for C_20_H_16_O_8_Na, 407.0737), 769.1791 [2M + H]^+^ (calcd for C_40_H_32_O_16_Na, 767.1763).

## Supporting Information Available

Plasmid and strain information; chemical schemas; NMR data for compounds **11**–**13** and SEK15; HPLC chromatograph traces of the *in vitro* and *in vivo* comparisons; mass spectra; high-resolution mass spectra, and mammalian cell cytotoxicity data (PDF)
