# Supplementary material for: Diverse Combinatorial Biosynthesis Strategies for C–H Functionalization of Anthracyclinones
Source: ACS Synth Biol. 2024 Apr 25;13(5):1523–36. doi: 10.1021/acssynbio.4c00043 (PMC11101304; doi:10.1021/acssynbio.4c00043)
Supplement: Supplementary file 1 — sb4c00043_si_001.pdf [file sb4c00043_si_001.pdf]

Diverse combinatorial biosynthesis strategies for C – H functionalization of anthracyclinones

Rongbin Wang [†,1], Benjamin Nji Wandi [†,1], Nora Schwartz [†,2], Jacob Hecht [†,2], Larissa Ponomoreva [3,4], Kendall Paige [2], Alexis West [2], Kathryn Desanti [2], Jennifer Nguyen [2], Jarmo Niemi [1], Jon S. Thorson [3,4], Khaled A. Shaaban [*,3,4], Mikko Metsä-Ketelä [*,1], and S. Eric Nybo [*,2]

[1] Department of Life Technologies, University of Turku, FIN-20014 Turku, [2] Department of Pharmaceutical Sciences, College of Pharmacy, Ferris State University, Big Rapids, MI 49307, [3] Center for Pharmaceutical Research and Innovation, [4] Department of Pharmaceutical Sciences, College of Pharmacy, University of Kentucky, Lexington, Kentucky 40536, United States

† These authors contributed equally to this work.

* **Corresponding Authors –** Prof. Dr. Khaled A. Shaaban, University of Kentucky, Lexington, KY. Email: Khaled_shaaban@uky.edu; Prof. Dr. Mikko Metsä-Ketelä, University of Turku, Finland. Email: mianme@utu.fi; Prof. Dr. S. Eric Nybo, Ferris State University, Big Rapids, MI. Email: EricNybo@Ferris.edu.

ORCID

Benjamin Nji Wandi: https://orcid.org/0000-0003-1071-3111

Larissa V. Ponomareva: https://orcid.org/0000-0003-2182-8774

Jarmo Niemi: https://orcid.org/0000-0002-7447-8379

Jon S. Thorson: https://orcid.org/0000-0002-7148-0721

Khaled A. Shaaban: https://orcid.org/0000-0001-7638-4942

Mikko Metsä-Ketelä: https://orcid.org/0000-0003-3176-2908

S. Eric Nybo: https://orcid.org/0000-0001-7884-7787

Table of Contents

Table S1. List of plasmids constructed and/or used in this study. 11

Table S2. List of strains constructed and/or used in this study. 14

Table S3. Summary of compounds 1-34 and HRMS data. 18

Table S4. ^{13}C (150 MHz) NMR Spectroscopic Data of Compounds 11 – 13 and SEK15 (δ in ppm). 19

Table S5. ^{1}H (600 MHz) NMR Spectroscopic Data of Compounds 11 – 13 and SEK15 (δ in ppm). 19

Method 1. Gene synthesis and plasmid construction for protein expression. 20

Method 2. Protein production and purification. 23

Method 3. Substrate purification for enzymatic assays. 25

Method 4. Enzymatic reactions with purified enzymes and substrates. 26

Figure S1. Mass spectrum of 2-hydroxy-aklanonic acid (9). 27

Figure S2. Mass spectrum of 2-hydroxy-nogalonic acid (10). 28

Figure S3: HPLC-MS analysis of 2-hydroxy-aklavinone (11). 29

Figure S4. (+)-HRESI-MS spectrum of 2-hydroxy-aklavinone (11). 30

Figure S5. ^{1}H NMR spectrum (DMSO-*d6*, 600 MHz) of 2-hydroxy-aklavinone (11). 31

Figure S6. ^{1}H NMR spectrum (DMSO-*d6*, 500 MHz) of 2-hydroxy-aklavinone (11). 32

Figure S7. ^{13}C NMR spectrum (DMSO-*d6*, 150 MHz) of 2-hydroxy-aklavinone (11). 33

Figure S8. ^{13}C NMR spectrum (DMSO-*d6*, 100 MHz) of 2-hydroxy-aklavinone (11). 34

Figure S9. ^{1}H (DMSO-*d6*, 600 MHz) and ^{13}C (DMSO-*d6*, 150 MHz) NMR spectra of 2-hydroxy-aklavinone (11). 35

Figure S10. ^{1}H (DMSO-*d6*, 500 MHz) and ^{13}C (DMSO-*d6*, 100 MHz) NMR spectra of 2-hydroxy-aklavinone (11). 36

Figure S11. ^{1}H,^{1}H-COSY spectrum (DMSO-*d6*, 500 MHz) of 2-hydroxy-aklavinone (11). 37

Figure S12. HSQC spectrum (DMSO-*d6*, 500 MHz) of 2-hydroxy-aklavinone (11). 38

Figure S13. HMBC spectrum (DMSO-*d6*, 600 MHz) of 2-hydroxy-aklavinone (11). 39

Figure S14. TOCSY spectrum (DMSO-*d6*, 500 MHz) of 2-hydroxy-aklavinone (11). 40

Figure S15. NOESY spectrum (DMSO-d_6, 600 MHz) of 2-hydroxy-aklavinone (11). 41

Figure S16: HPLC-MS analysis of 2-hydroxy-9-*epi*-aklavinone (12). 42

Figure S17. (+)-HRESI-MS spectrum of 2-hydroxy-9-*epi*-aklavinone (12). 43

Figure S18. ^{1}H NMR spectrum (DMSO-d_6, 600 MHz) of 2-hydroxy-9-*epi*-aklavinone (12). 44

Figure S19. ^{13}C NMR spectrum (DMSO-d_6, 150 MHz) of 2-hydroxy-9-*epi*-aklavinone (12). 45

Figure S20. ^{1}H (DMSO-d_6, 600 MHz) and ^{13}C (DMSO-d_6, 150 MHz) NMR spectra of 2-hydroxy-9-*epi*-aklavinone (12). 46

Figure S21. ^{1}H,^{1}H-COSY spectrum (DMSO-d_6, 600 MHz) of 2-hydroxy-9-*epi*-aklavinone (12). 47

Figure S22. HSQC spectrum (DMSO-d_6, 600 MHz) of 2-hydroxy-9-*epi*-aklavinone (12). 48

Figure S23. HMBC spectrum (DMSO-d_6, 600 MHz) of 2-hydroxy-9-*epi*-aklavinone (12). 49

Figure S24. TOCSY spectrum (DMSO-d_6, 600 MHz) of 2-hydroxy-9-*epi*-aklavinone (12). 50

Figure S25. NOESY spectrum (DMSO-d_6, 600 MHz) of 2-hydroxy-9-*epi*-aklavinone (12). 51

Figure S26: HPLC-MS analysis of 2-hydroxy-auramycinone (13). 52

Figure S27. (+)-HRESI-MS spectrum of 2-Hydroxy-auramycinone (13). 53

Figure S28. ^{1}H NMR spectrum (DMSO-d_6, 600 MHz) of 2-hydroxy-auramycinone (13). 54

Figure S29. ^{13}C NMR spectrum (DMSO-d_6, 150 MHz) of 2-hydroxy-auramycinone (13). 55

Figure S30. ^{1}H (DMSO-d_6, 600 MHz) and ^{13}C (DMSO-d_6, 150 MHz) NMR spectra of 2-hydroxy-auramycinone (13). 56

Figure S31. ^{1}H,^{1}H-COSY spectrum (DMSO-d_6, 600 MHz) of 2-hydroxy-auramycinone (13). 57

Figure S32. HSQC spectrum (DMSO-d_6, 600 MHz) of 2-hydroxy-auramycinone (13). 58

Figure S33. HMBC spectrum (DMSO-d_6, 600 MHz) of 2-hydroxy-auramycinone (13). 59

Figure S34. TOCSY spectrum (DMSO-d_6, 600 MHz) of 2-hydroxy-auramycinone (13). 60

Figure S35. NOESY spectrum (DMSO-d_6, 600 MHz) of 2-hydroxy-auramycinone (13). 61

Figure S36. Mass spectrum of 2-hydroxy-nogalamycinone (14). 62

Figure S37: HPLC-MS analysis of SEK15. 63

Figure S38. (+)-HRESI-MS spectrum of SEK15. 64

Figure S39. ^{1}H NMR spectrum (CD_3OD, 600 MHz) of SEK15. 65

Figure S40. ^{13}C NMR spectrum (CD_3OD, 150 MHz) of SEK15. 66

Figure S41. ^{1}H (CD_3OD, 600 MHz) and ^{13}C (CD_3OD, 150 MHz) NMR spectra of SEK15. 67

Figure S42. ^{1}H,^{1}H-COSY spectrum (CD_3OD, 600 MHz) of SEK15. 68

Figure S43. HSQC spectrum (CD_3OD, 600 MHz) of SEK15. 69

Figure S44. HMBC spectrum (CD_3OD, 600 MHz) of SEK15. 70

Figure S45. TOCSY spectrum (CD_3OD, 600 MHz) of SEK15. 71

Figure S46. NOESY spectrum (CD_3OD, 600 MHz) of SEK15. 72

Figure S47. Mass spectrum of aklavinone (1) standard used for *in vitro* reactions. 73

Figure S48. Mass spectrum of aklavinone (1) and 7-deoxy-aklavinone (5) identified from *in vivo* extracts. 74

Figure S49. (-)-HRESI-MS spectrum of aklavinone (1). 75

Figure S50. Mass spectrum of 9-*epi*-aklavinone (2) standard used for *in vitro* reactions 76

Figure S51. Mass spectrum of 9-*epi*-aklavinone (2) and 7-deoxy-9-*epi*-aklavinone (6) identified from *in vivo* extracts. 77

Figure S52. (-)-HRESI-MS spectrum of 9-*epi*-aklavinone (2). 78

Figure S53. Mass spectrum of auramycinone (3) standard used in *in vitro* reactions. 79

Figure S54. Mass spectrum of auramycinone (3) and 7-deoxy-auramycinone (7) identified from *in vivo* extracts. 80

Figure S55. (-)-HRESI-MS spectrum of auramycinone (3). 81

Figure S56. Mass spectrum of nogalamycinone (4) used in *in vitro* reactions. 82

Figure S57. Mass spectrum of nogalamycinone (4) and 7-deoxy-nogalamycinone (8) identified in *in vivo* extracts. 83

Figure S58. (-)-HRESI-MS spectrum of nogalamycinone (4). 84

Figure S59. (-)-HRESI-MS spectrum of 7-deoxy-aklavinone (5). 85

Figure S60. (-)-HRESI-MS spectrum of 7-deoxy-9-*epi*-aklavinone (6). 86

Figure S61. (-)-HRESI-MS spectrum of 7-deoxy-auramycinone (7). 87

Figure S62. (-)-HRESI-MS spectrum of 7-deoxy-nogalamycinone (8). 88

Figure S63. Mass spectrum of epsilon-rhodomycinone (15) standard. 89

Figure S64. Mass spectrum of maggiemycin and epsilon-rhodomycinone (15) identified from strain co-expressing the aklavinone pathway and *dnrF*. 90

Figure S65. (-)-HRESI-MS spectrum of epsilon-rhodomycinone (15). 91

Figure S66. Alignment of epsilon-rhodomycinone (15) produced both *in vivo* and *in vitro*. 92

Figure S67. Mass spectrum of 11-hydroxy-9-*epi*-aklavinone (16) identified from strain co-expressing the 9-*epi*-aklavinone pathway and *dnrF*. 93

Figure S68. (-)-HRESI-MS spectrum of 11-hydroxy-9-*epi*-aklavinone (16). 94

Figure S69. Alignment of 11-hydroxy-9-*epi*-aklavinone (16) produced both *in vivo* and *in vitro*. 95

Figure S70. Mass spectrum of 11-hydroxy-auramycinone (17) identified from strain co-expressing the auramycinone pathway and *rdmE*. 96

Figure S71. (-)-HRESI-MS spectrum of 11-hydroxy-auramycinone (17). 97

Figure S72. Alignment of 11-hydroxy-auramycinone (17) produced both *in vivo* and *in vitro*. 98

Figure S73. Mass spectrum of 11-hydroxy-nogalamycinone (18) identified from strain co-expressing the nogalamycinone pathway and *dnrF*. 99

Figure S74. (-)-HRESI-MS spectrum of 11-hydroxy-nogalamycinone (18). 100

Figure S75. Alignment of 11-hydroxy-nogalamycinone (18) produced both *in vivo* and *in vitro*. 101

Figure S76. Mass spectrum of 10-decarboxy-aklavinone (19) produced in in vitro assay with purified aklavinone and EamC+K 102

Figure S77. Mass spectrum of 10-decarboxy aklavinone (19) identified from strain co-expressing the aklavinone pathway and *eamC+K*. 103

Figure S78. (-)-HRESI-MS spectrum of 10-decarboxy-aklavinone (19). 104

Figure S79. Alignment of 10-decarboxy-aklavinone (19) produced both *in vitro* and *in vivo*. 105

Figure S80. Mass spectrum of 10-decarboxy-9-*epi*-aklavinone (20) produced in *in vitro* assay with purified 9-*epi*-aklavinone and EamC+K. 106

Figure S81. Mass spectrum of 10-decarboxy-9-*epi*-aklavinone (20) identified from strain co-expressing the 9-*epi*-aklavinone pathway and *eamC+K*. 107

Figure S82. (-)-HRESI-MS spectrum of 10-decarboxy-9-*epi*-aklavinone (20). 108

Figure S83. Alignment of 10-decarboxy-9-*epi*-aklavinone (20) produced both *in vitro* and *in vivo*. 109

Figure S84. Mass spectrum of 10-decarboxy-auramycinone (21) produced in *in vitro* assay with purified auramycinone and EamC+K. 110

Figure S85. Mass spectrum of 10-decarboxy-auramycinone (21) identified from strain co-expressing the auramycinone pathway and *eamC+K*. 111

Figure S86. (-)-HRESI-MS spectrum of 10-decarboxy-auramycinone (21). 112

Figure S87. Alignment of 10-decarboxy-auramycinone (21) produced *in vitro* and *in vivo*. 113

Figure S88. Mass spectrum of 10-decarboxy-nogalamycinone (22) produced in *in vitro* assay with purified nogalamycinone and EamC+K. 114

Figure S89. Mass spectrum of 10-decarboxy-nogalamycinone (22) identified from strain co-expressing the nogalamycinone pathway and *eamC+K*. 115

Figure S90. (-)-HRESI-MS spectrum of 10-decarboxy-nogalamycinone (22). 116

Figure S91. Alignment of 10-decarboxy nogalamycinone (22) produced both *in vitro* and *in vivo*. 117

Figure S92. Mass spectrum of 10-hydroxy-aklavinone (23) produced in *in vitro* assay with purified aklavinone and EamC+RdmB. 118

Figure S93. Mass spectrum of 10-hydroxy-aklavinone (23) identified from strain co-expressing the aklavinone pathway and *rdmC+B*. 119

Figure S94. (-)-HRESI-MS spectrum of 10-hydroxy-aklavinone (23). 120

Figure S95. Mass spectrum of 10-hydroxy-auramycinone (24) produced in *in vitro* assay from purified auramycinone and EamC+RdmB. 121

Figure S96. Mass spectrum of 10-hydroxy-auramycinone (24) identified from strain co-expressing the auramycinone pathway and *rdmC+B*. 122

Figure S97. (-)-HRESI-MS spectrum of 10-hydroxy-auramycinone (24). 123

Figure S98. Mass spectrum of β-rhodomycinone (25) produced in *in vitro* assay with purified aklavinone and DnrF+EamC+RdmB........124

Figure S99. Mass spectrum of β-rhodomycinone (25) identified from strain co-expressing the aklavinone pathway and *rdmE+C+B.* 125

Figure S100. (-)-HRESI-MS spectrum of beta-rhodomycinone (25). 126

Figure S101. Alignment of β-rhodomycinone (25) produced both *in vitro* and *in vivo.*...127

Figure S102. Mass spectrum of 10,11-dihydroxy-auramycinone (26) produced in *in vitro* assay with purified auramycinone and DnrF+EamC+RdmB. 128

Figure S103. Mass spectrum of 10,11-dihydroxy-auramycinone (26) identified from strain co-expressing the auramycinone pathway and *rdmE+C+B.*...129

Figure S104. Alignment of 10,11-dihydroxy-auramycinone (26) produced both *in vitro* and *in vivo.*..130

Figure S105. Production titers of compounds 25 and 26 from lines expressing the *rdmE+C+B* construct..131

Figure S106. Mass spectrum of 1-hydroxy-aklavinone (27) produced in *in vitro* assay with purified aklavinone and KstA15+A16. 132

Figure S107. Mass spectrum of 1-hydroxy-aklavinone (27) identified from a strain co-expressing the aklavinone pathway and KstA15+A16. 133

Figure S108. (-)-HRESI-MS spectrum of 1-hydroxy-aklavinone (27)...134

Figure S109. Alignment of 1-hydroxy-aklavinone (27) produced *in vitro* and *in vivo.*...135

Figure S110. Mass spectrum of 1-hydroxy-9-*epi*-aklavinone (28) identified from strain co-expressing the 9-*epi*-aklavinone pathway with *kstA15+A16.* 136

Figure S111. (-)-HRESI-MS spectrum of 1-hydroxy-9-*epi*-aklavinone (28). 137

Figure S112. Alignment of 1-hydroxy-9-*epi*-aklavinone (28) produced *in vitro* and *in vivo.* 138

Figure S113. Mass spectrum of 1-hydroxy-auramycinone (29) produced in *in vitro* assay with purified auramycinone and KstA15+A16. 139

Figure S114. Mass spectrum of 1-hydroxy-auramycinone (29) identified from a strain co-expressing the auramycinone pathway and *kstA15+A16.* 140

Figure S115. (-)-HRESI-MS spectrum of 1-hydroxy-auramycinone (29). 141

Figure S116. Alignment of 1-hydroxy-auramycinone (29) produced both *in vitro* and *in vivo.* 142

Figure S117. Mass spectrum of 1-hydroxy-nogalamycinone (30) produced in *in vitro* assay with purified nogalamycinone and KstA15+A16. 143

Figure S118. Mass spectrum of 1-hydroxy-nogalamycinone (30) identified from strain co-expressing the nogalamycinone pathway and *kstA15+A16.* 144

Figure S119. (-)-HRESI-MS spectrum of 1-hydroxy-nogalamycinone (30). 145

Figure S120. Alignment of 1-hydroxy-nogalamycinone (30) produced *in vitro* and *in vivo*. 146

Figure S121. Mass spectrum of aklavinone (1) and *iso*-aklavinone (31) identified from a strain co-expressing the aklavinone pathway and *kstA15+A16+A10+A11.* 147

Figure S122. (-)-HRESI-MS spectrum of 1-iso-aklavinone (31) 148

Figure S123. Alignment of *iso*-aklavinone (31) produced both *in vitro* and *in vivo.* 149

Figure S124. Mass spectrum of 9-*epi*-aklavinone (2) and *iso*-9-*epi*-aklavinone (32) produced from a strain co-expressing the 9-*epi*-aklavinone pathway and *kstA15+A16+A10+A11.* 150

Figure S125. (-)-HRESI-MS spectrum of 1-*iso*-9-*epi*-aklavinone (32). 151

Figure S126. Alignment of *iso*-9-*epi*-aklavinone (32) produced both *in vitro* and *in vivo.* 152

Figure S127. Mass spectrum of auramycinone (3) and *iso*-auramycinone (33) produced from a strain co-expressing the 9-*epi*-aklavinone pathway and *kstA15+A16+A10+A11.* 153

Figure S128. (-)-HRESI-MS spectrum of 1-iso-auramycinone (33). 154

Figure S129. Alignment of *iso*-auramycinone (33) produced *in vitro* and *in vivo*. 155

Figure S130. Mass spectrum of nogalamycinone (4) and *iso*-nogalamycinone (34) produced from a strain co-expressing the 9-*epi*-aklavinone pathway and *kstA15+A16+A10+A11.* 156

Figure S131. Figure (-)-HRESI-MS spectrum of 1-iso-nogalamycinone (34). 157

Figure S132. Alignment of *iso*-nogalamycinone (34) produced *in vitro* and *in vivo*. 158

Figure S133. Production titers of 31-34 from strains expressing *kstA15+A16+A10+A11*. 159

Figure S134. % Viability vs 100% of untreated control of the generated strains extracts at 40 μg/mL concentration. 160

Figure S135. % Viability vs 100% of untreated control of compounds 11-13, 15 and SEK15, at 3 μM concentration. 161

Table S6. Cytotoxic activities of the generated strain extracts in PC3, TC32, A549, and HCT116 cell lines. 162

References 163

Table S1. List of plasmids constructed and/or used in this study.

Plasmid	Genotype and relevant characteristics	Reference
pSET152BB	BioBricks®-compatible vector pSET152; *aac3(IV)*^{R}, *oriT*, ϕC31int, *attP*, MCS	[1]
pSET154BB	pSET152 with BioBricks® [RFC 10] compatible restriction sites in the polylinker; *aac3(IV)*, *oriT*, *lacZ*α, ΦC31int, *attP*, MCS with strong 5′-*tt-sbi-A* terminator and 3′-*fd* phage terminator	[2]
pOSV802	BioBricks®-compatible vector; *aac(3)IV*^{R}*, oriT*, ϕC31int, *attP*, *amilCFP*, MCS	[3]
pOSV808	BioBricks®-compatible vector; *hph*^{R}*, oriT*, VWBint, *attP*, *amilCFP*, MCS	[3]
pOSV821	BioBricks®-compatible vector; *aac(3)IV*^{R}*, oriT*, ϕC31int, *attP*, *amilCFP*, MCS with strong 5′-*tt-sbi-A* terminator and 3′-*fd* phage terminator	This study
pENSV3	pENSV1 with strong 5′- ECK120010818-term and 3′-ECK120029600-spy-term terminators	[2]
pOSV808-*accA2BE*	*ermE**p-*accA2BE* fragment cloned into pOSV808	[1]
pOSV808-*scbr2*	*Scbr2* under the control of its native promoter cloned into pOSV808.	This study.
pOSV808-*ssgA*	*ssgA* under the control of synthetic promoter ScoSPL20 and the strong *tuf1* RBS	This study
pSET-S2C1	wild-type *snoa123* and codon-optimized *mtmQY* fused to the strong tandem *kasOp** promoters; cloned in pSET152BB vector	This study
pSET-A2C1	wild-type *aknBCDE2F* and codon-optimized *mtmQY* fused to the strong *kasOp** promoter; cloned in pSET152BB vector	This study
pSET-A2C1A6	wild-type *aknBCDE2F* and codon-optimized *mtmQY* fused to the strong *kasOp** promoter; codon-optimized *aknGHU* fused to the strong *sp44* promoter; cloned in pSET152BB vector	This study
pSET-A2C1S6	wild-type *aknBCDE2F* and codon-optimized *mtmQY* fused to the strong *kasOp** promoter; codon-optimized *snoaCFL* fused to the strong *sp44* promoter; cloned in pSET152BB vector	This study

pSET-S2C1A6	wild-type *snoa123* and codon-optimized *mtmQY* fused to the strong *kasOp** promoter; codon-optimized *aknGHU* fused to the strong *sp44* promoter; cloned in pSET152BB vector	This study
pSET-S2C1S6	wild-type *snoa123* and codon-optimized *mtmQY* fused to the strong *kasOp** promoter; codon-optimized *snoaCFL* fused to the strong *sp44* promoter; cloned in pSET152BB vector	This study
pAKV2	wild-type *aknBCDE2F* fused to the strong *sp41-ltsvJ* promoter insulator-ribozyme; wildtype *aknAE1WX* fused to the strong *sp42-vtmoJ* promoter insulator-ribozyme; wildtype *aknGHU* fused to the strong *sp44-riboJ* promoter insulator-ribozyme; cloned in vector pOSV821	[4,] This study
pEAKV2	wild-type *aknBCDE2F* fused to the strong *sp41-ltsvJ* promoter insulator-ribozyme; wildtype *aknAE1WX* fused to the strong *sp42-vtmoJ* promoter insulator-ribozyme; wildtype *snoaLFC* fused to the strong *sp44-riboJ* promoter insulator-ribozyme; cloned in vector pOSV821	[4,] This study
pAURA2	wild-type *snoa123* fused to the strong *sp41-ltsvJ* promoter insulator-ribozyme; wildtype *aknAE1WX* fused to the strong *sp42-vtmoJ* promoter insulator-ribozyme; wildtype *aknGHU* fused to the strong *sp44-riboJ* promoter insulator-ribozyme; cloned in vector pOSV821	[4,] This study
pNOG2	wild-type *snoa123* fused to the strong *sp41-ltsvJ* promoter insulator-ribozyme; wildtype *aknAE1WX* fused to the strong *sp42-vtmoJ* promoter insulator-ribozyme; wildtype *snoaLFC* fused to the strong *sp44-riboJ* promoter insulator-ribozyme; cloned in vector pOSV821	[4,] This study
pHAKV2	wild-type *aknBCDE2F* fused to the strong *sp41-ltsvJ* promoter insulator-ribozyme; wildtype *mtmQY* fused to the strong *sp42-vtmoJ* promoter insulator-ribozyme; wildtype *aknGHU* fused to the strong *sp44-riboJ* promoter insulator-ribozyme; cloned in vector pOSV821	[4,] This study
pHEAKV2	wild-type *aknBCDE2F* fused to the strong *sp41-ltsvJ* promoter insulator-ribozyme; wildtype *mtmQY* fused to the strong *sp42-vtmoJ* promoter insulator-ribozyme; wildtype *snoaLFC* fused to the strong *sp44-riboJ* promoter insulator-ribozyme; cloned in vector pOSV821	[4,] This study

pHAURA2	wild-type *snoa123* fused to the strong *sp41-ltsvJ* promoter insulator-ribozyme; wildtype *mtmQY* fused to the strong *sp42-vtmoJ* promoter insulator-ribozyme; wildtype *aknGHU* fused to the strong *sp44-riboJ* promoter insulator-ribozyme; cloned in vector pOSV821	[4,] This study
pHNOG2	wild-type *snoa123* fused to the strong *sp41-ltsvJ* promoter insulator-ribozyme; wildtype *mtmQY* fused to the strong *sp42-vtmoJ* promoter insulator-ribozyme; wildtype *snoaLFC* fused to the strong *sp44-riboJ* promoter insulator-ribozyme; cloned in vector pOSV821	[4,] This study
pSV-*dnrF*	*gapdhpEL-dnrF* cloned in pENSV3	This study
pSV-*eamCK*	*gapdhpEL-eamC+K* cloned in pENSV3	This study
pSV-*rdmCB*	*gapdhpEL-rdmCB* cloned in pENSV3	This study
pSV-*rdmECB*	*gapdhpEL-rdmECB* cloned in pENSV3	
pSV-*eamCK*	*gapdhpEL-eamC+K* cloned in pENSV3	This study
pSV-*kstA15-A16*	*gapdhpEL-kstA15-A16* cloned in pENSV3	This study
pSV-*kstA15-A16-A10-A11*	*gapdhpEL-kstA15-A16-A10-A11* cloned in pENSV3	This study
pBAD-*dnrF*	*dnrF* under the control of the pBAD promoter.	This study
pBAD-*kstA15*	*kstA15* under the control of the pBAD promoter.	This study
pBAD-*kstA16*	*kstA16* under the control of the pBAD promoter.	This study
pBAD-*kstA10*	*kstA10* under the control of the pBAD promoter.	This study
pBAD-*kstA11*	*kstA11* under the control of the pBAD promoter.	This study

Table S2. List of strains constructed and/or used in this study.

Strain	Genotype and relevant characteristics	Reference
E. coli JM109	endA1 glnV44 thi-1 relA1 gyrA96 recA1 mcrB+Δ(lac-proAB) e14-[F' traD36 proAB+ lacIq lacZ ΔM15] sdR17(rK-mK+); general cloning host	[5]
E. coli ET12567	*F- dam*13::Tn9 *dcm*6 *hsdM hsdR zjj*-202::Tn10 *recF*143 *galK2 galT22 ara*-14 *lac*Y1 *xyl*-5 *leuB6 thi*-1 *tonA*31 *rpsL*136 *his*G4 *tsx*-78 *mtl*-1 *glnV*44	[6]
E. coli ET12567*/* (pUZ8002*)*	*F- dam*13::Tn9 *dcm*6 *hsdM hsdR zjj*-202::Tn10 *recF*143 *galK2 galT22 ara*-14 *lac*Y1 *xyl*-5 *leuB6 thi*-1 *tonA*31 *rpsL*136 *his*G4 *tsx*-78 *mtl*-1 *glnV*44; *tra, neo^R, RP4*; *E. coli*-*Streptomyces* intergeneric conjugation host	[6]
Streptomyces coelicolor M1152Δ*matAB*	SCP1$^-$, SCP2$^-$, Δ*act* Δ*red* Δ*cpk* Δ*cda* Δ*sco2962-2963*	[2]
S. coelicolor::pA2C1	*S. coelicolor* M1152Δ*matAB* transformed with plasmid pA2C1. Produces **9**.	This study
S. coelicolor::pS2C1	*S. coelicolor* M1152Δ*matAB* transformed with plasmid pA2C1. Produces **10**.	This study
S. coelicolor::pA2C1A6	*S. coelicolor* M1152Δ*matAB* transformed with plasmid pA2C1A6. Produces **11**.	This study
S. coelicolor::pA2C1S6	*S. coelicolor* M1152Δ*matAB* transformed with plasmid pA2C1S6. Produces **12**.	This study
S. coelicolor::pS2C1A6	*S. coelicolor* M1152Δ*matAB* transformed with plasmid pS2C1A6. Produces **13**.	This study
S. coelicolor::pA2C1A6	*S. coelicolor* M1152Δ*matAB* transformed with plasmid pS2C1S6. Produces **14**.	This study
S. coelicolor::AKV2	*S. coelicolor* M1152Δ*matAB* transformed with plasmid AKV2. Produces **1** and **5**.	This study
S. coelicolor::EAKV2	*S. coelicolor* M1152Δ*matAB* transformed with plasmid EAKV2. Produces **2** and **6**.	This study
S. coelicolor::AURA2	*S. coelicolor* M1152Δ*matAB* transformed with plasmid AKV2. Produces **3** and **7**.	This study

S. coelicolor::NOG2	*S. coelicolor* M1152Δ*matAB* transformed with plasmid EAKV2. Produces **4** and **8**.	This study
S. coelicolor::HAKV2	*S. coelicolor* M1152Δ*matAB* transformed with plasmid AKV2. Produces **11**.	This study
S. coelicolor::HEAKV2	*S. coelicolor* M1152Δ*matAB* transformed with plasmid EAKV2. Produces **12**.	This study
S. coelicolor::HAURA2	*S. coelicolor* M1152Δ*matAB* transformed with plasmid AKV2. Produces **13**.	This study
S. coelicolor::HNOG2	*S. coelicolor* M1152Δ*matAB* transformed with plasmid EAKV2. Produces **14**.	This study
S.coelicolor::*accA2BE*::NOG2	*S. coelicolor* transformed with pOSV808-*accA2BE* and pNOG2	This study
*S.coelicolor::scbr2::*NOG2	*S. coelicolor* transformed with pOSV808-*scbr2* and pNOG2	This study
*S.coelicolor::ssgA::*NOG2	*S. coelicolor* transformed with pOSV808-*ssgA* and pNOG2	This study
S.coelicolor::*accA2BE*::EAKV2	*S. coelicolor* transformed with pOSV808-*accA2BE* and pEAKV2	This study
*S.coelicolor::scbr2::*EAKV2	*S. coelicolor* transformed with pOSV808-*scbr2* and pEAKV2	This study
*S.coelicolor::ssgA::*EAKV2	*S. coelicolor* transformed with pOSV808-*ssgA* and pEAKV2	This study
S. coelicolor::AKV2::*dnrF*	*S. coelicolor transformed with* pAKV2 and pSV-*dnrF*	This study
S. coelicolor::AKV2::*eamCK*	*S. coelicolor transformed with* pAKV2 and pSV-*eamCK*	This study
S. coelicolor::AKV2::*rdmCB*	*S. coelicolor transformed with* pAKV2 and pSV-*rdmCB*	This study
S. coelicolor::AKV2::*rdmECB*	*S. coelicolor transformed with* pAKV2 and pSV-*rdmECB*	This study
S. coelicolor::AKV2::*kstA15-A16*	*S. coelicolor transformed with* pAKV2 and pSV-*kstA15-A16*	This study
S. coelicolor::AKV2::*kstA15-A16-A10-A11*	*S. coelicolor transformed with* pAKV2 and pSV-*kstA15-A16-A10-A11*	This study
S. coelicolor::EAKV2::*dnrF*	*S. coelicolor transformed with* pEAKV2 and pSV-*dnrF*	This study

S. coelicolor::EAKV2::*eamCK*	*S. coelicolor transformed with* pEAKV2 and pSV-*eamCK*	This study
S. coelicolor::EAKV2::*rdmCB*	*S. coelicolor transformed with* pEAKV2 and pSV-*rdmCB*	This study
S. coelicolor::EAKV2::*rdmECB*	*S. coelicolor transformed with* pEAKV2 and pSV-*rdmECB*	This study
S. coelicolor::EAKV2::*kstA15-A16*	*S. coelicolor transformed with* pEAKV2 and pSV-*kstA15-A16*	This study
S. coelicolor::EAKV2::*kstA15-A16-A10-A11*	*S. coelicolor transformed with* pEAKV2 and pSV-*kstA15-A16-A10-A11*	This study
S. coelicolor::AURA2::*dnrF*	*S. coelicolor transformed with* pAURA2 and pSV-*dnrF*	This study
S. coelicolor::AURA2::*eamCK*	*S. coelicolor transformed with* pAURA2 and pSV-*eamCK*	This study
S. coelicolor::AURA2::*rdmCB*	*S. coelicolor transformed with* pAURA2 and pSV-*rdmCB*	This study
S. coelicolor::AURA2::*rdmECB*	*S. coelicolor transformed with* pAURA2 and pSV-*rdmECB*	This study
S. coelicolor::AURA2::*kstA15-A16*	*S. coelicolor transformed with* pAURA2 and pSV-*kstA15-A16*	This study
S. coelicolor::AURA2::*kstA15-A16-A10-A11*	*S. coelicolor transformed with* pAURA2 and pSV-*kstA15-A16-A10-A11*	This study
S. coelicolor::NOG2::*dnrF*	*S. coelicolor transformed with* pNOG2 and pSV-*dnrF*	This study
S. coelicolor::NOG2::*eamCK*	*S. coelicolor transformed with* pNOG2 and pSV-*eamCK*	This study
S. coelicolor::NOG2::*rdmCB*	*S. coelicolor transformed with* pNOG2 and pSV-*rdmCB*	This study
S. coelicolor::NOG2::*rdmECB*	*S. coelicolor transformed with* pNOG2 and pSV-*rdmECB*	This study
S. coelicolor::NOG2::*kstA15-A16*	*S. coelicolor transformed with* pNOG2 and pSV-*kstA15-A16*	This study
S. coelicolor::NOG2::*kstA15-A16-A10-A11*	*S. coelicolor transformed with* pNOG2 and pSV-*kstA15-A16-A10-A11*	This study
E. coli TOP10/pBAD-*dnrF*	Heterologous host for producing DnrF.	This study

E. coli TOP10/pJN054	Heterologous host for producing RdmE.	[7]
E. coli TOP10/pBAD-*rdmB*	Heterologous host for producing RdmB.	[8]
E. coli TOP10/pBAD-*rdmC*	Heterologous host for producing RdmC.	[8]
E. coli TOP10/pBAD-*eamC*	Heterologous host for producing EamC.	[8]
E. coli TOP10/pBAD-*eamK*	Heterologous host for producing EamK.	[8]
E. coli TOP10/pBAD-*kstA15*	Heterologous host for producing KstA15.	This study.
E. coli TOP10/pBAD-*kstA16*	Heterologous host for producing KstA16.	This study.
E. coli TOP10/pBAD-*kstA10*	Heterologous host for producing KstA10.	This study.
E. coli TOP10/pBAD-*kstA11*	Heterologous host for producing KstA11.	This study.

Table S3. Summary of compounds 1-34 and HRMS data.

Comp No.	Compound Name	Mass	MF	Calcd. [M-H]	$[M-H]^-$	Figure No.
**1**	aklavinone	412	C22H20O8	411.108541	411.1085	Figure S49
**2**	9-*epi*-aklavinone	412	C22H20O8	411.108541	411.1086	Figure S52
**3**	auramycinone	398	C21H18O8	397.092891	397.0929	Figure S55
**4**	nogalamycinone	398	C21H18O8	397.092891	397.0963	Figure S58
**5**	7-deoxy-aklavinone	396	C22H20O7	395.113627	395.1135	Figure S59
**6**	7-deoxy-9-*epi*-aklavinone	396	C22H20O7	395.113627	395.1138	Figure S60
**7**	7-deoxy-auramycinone	382	C21H18O7	381.097976	381.0974	Figure S61
**8**	7-deoxy-nogalamycinone	382	C21H18O7	381.097976	381.0972	Figure S62
**9**	2-hydroxy-aklanonic acid	412	C21H16O9	411.072156	NA	NA
**10**	2-hydroxy-nogalonic acid	398	C20H14O9	397.056506	NA	NA
**11**	2-hydroxy-aklavinone	428	C22H20O9	427.103456	Scaled-up	Scaled-up
**12**	2-hydroxy-9-*epi*-aklavinone	428	C22H20O9	427.103456	Scaled-up	Scaled-up
**13**	2-hydroxy-auramycinone	414	C21H18O9	413.087806	Scaled-up	Scaled-up
**14**	2-hydroxy-nogalamycinone	414	C21H18O9	413.087806	NA	NA
**15**	epsilon-rhodomycinone	428	C22H20O9	427.103456	427.1036	Figure S65
**16**	11-hydroxy-9-*epi*-aklavinone	428	C22H20O9	427.103456	427.1042	Figure S68
**17**	11-hydroxy-auramycinone	414	C21H18O9	413.087806	413.0886	Figure S71
**18**	11-hydroxy-nogalamycinone	414	C21H18O9	413.087806	413.0897	Figure S74
**19**	10-decarboxy-aklavinone	354	C20H18O6	353.103062	353.1026	Figure S78
**20**	10-decarboxy-9-*epi*-aklavinone	354	C20H18O6	353.103062	353.1034	Figure S82
**21**	10-decarboxy-auramycinone	340	C19H16O6	339.087412	339.0885	Figure S86
**22**	10-decarboxy-nogalamycinone	340	C19H16O6	339.087412	339.0880	Figure S90
**23**	10-hydroxy-aklavinone	370	C20H18O7	369.097976	369.0992	Figure S94
**24**	10-hydroxy-auramycinone	356	C19H16O7	355.082326	355.0831	Figure S97
**25**	beta-rhodomycinone	386	C20H18O8	385.092891	385.0928	Figure S100
**26**	10,11-dihydroxy-auramycinone	372	C19H16O8	371.077241	ND	ND
**27**	1-hydroxy-aklavinone	428	C22H20O9	427.103456	427.1035	Figure S108
**28**	1-hydroxy-9-*epi*-aklavinone	428	C22H20O9	427.103456	427.1037	Figure S111
**29**	1-hydroxy-auramycinone	414	C21H18O9	413.087806	413.0887	Figure S115
**30**	1-hydroxy-nogalamycinone	414	C21H18O9	413.087806	413.0892	Figure S119
**31**	1-*iso*-aklavinone	412	C22H20O8	411.108541	411.1078	Figure S122
**32**	1-*iso*-9-*epi*-aklavinone	412	C22H20O8	411.108541	411.1088	Figure S125
**33**	1-*iso*-auramycinone	398	C21H18O8	397.092891	397.0939	Figure S128
**34**	1-*iso*-nogalamycinone	398	C21H18O8	397.092891	397.0937	Figure S131

ND: Not detected; **NA**= Not Assigned; **Scale-up**= Compound was isolated and identified by full NMR and MS data analysis (from the scale-up fermentation followed by isolation and purification and structure elucidation)

Table S4. ^{13}C (150 MHz) NMR Spectroscopic Data of Compounds **11 – 13** and SEK15 (δ in ppm).

Position	**11** a)	**12** a)	**13** a)	Position	**SEK15** b)
	δ_C, type	δ_C, type	δ_C, type		δ_C, type
1	109.2, CH	108.9, CH	108.9, CH	2	168.2, C
2	166.2, C	165.7, C	165.7, C	3	90.0, CH
3	108.0, CH	108.0, CH	108.0, CH	4	173.2, C
4	164.6, C	164.5, C	164.5, C	5	103.0, CH
4a	108.9, C	109.1, C	109.1, C	6	165.9, C
5	189.6, C	190.0, C	189.9, C	7	38.6, CH_2
5a	114.3, C	114.1, C	114.1, C	8	136.5, C
6	160.8, C	161.0, C	160.8, C	9	111.6, CH
6a	133.6, C	133.9, C	133.6, C	10	162.2, C
7	60.6, CH	61.2, CH	60.8, CH	11	103.3, CH
8	35.4, CH_2	36.4, CH_2	38.3, CH_2	12	159.9, C
9	71.4, C	70.3, C	69.6, C	13	122.4, C
10	56.4, CH	57.0, CH	57.4, CH	14	202.8, C
10a	140.9, C	142.3, C	140.9, C	15	118.7, C
11	119.3, CH	119.4, CH	119.3, CH	16	165.1, C
11a	132.0, C	131.9, C	131.9, C	17	102.0, CH
12	181.2, C	181.1, C	181.0, C	18	164.4, C
12a	135.2, C	135.3, C	135.2, C	19	112.5, CH
13	31.9, CH_2	32.7, CH_2	27.3, CH_3	20	143,7, C
14	6.6, CH_3	7.5, CH_3		20-CH_3	21.8, CH_3
15	171.0, C	171.2, C	171.1, C		
16	52.4, CH_3	51.8, CH_3	52.3, CH_3		

a) DMSO-d_6; b) CD_3OD; See Supplementary Information for NMR spectra. Assignments supported by 2D HSQC and HMBC experiments.

Table S5. ^1H (600 MHz) NMR Spectroscopic Data of Compounds **11 – 13** and **SEK15** (δ in ppm).

**11** a)	**12** a)	**13** a)	**SEK15** b)

Position	δ_H (mult, J in [Hz])	δ_H (mult, J in [Hz])	δ_H (mult, J in [Hz])	Position	δ_H (mult, J in [Hz])
1	7.13 (d, 2.5)	7.14 (d, 2.4)	7.12 (d, 2.4)	3	5.25 (brs)
2-OH	11.46 (brs)	11.44 (brs)	11.45 (brs)	5	5.66 (brs)
3	6.60 (d, 2.5)	6.63 (d, 2.4)	6.62 (d, 2.3)	7	3.59 (brs)
4-OH	12.03 (s)	12.01 (s)	11.99 (s)	9	6.31 (d, 2.3)
6-OH	12.77 (s)	12.75 (s)	12.70 (s)	11	6.30 (d, 2.2)
7	5.09 (brd, 4.5)	5.11 (brs)	5.05 (brdd, 4.8, 2.3)	17	6.16 (d, 2.4)
7-OH	5.30 (brs)	5.08 (brs)	5.15 (brs)	19	6.10 (brd, 2.2)
8	2.25 (dd, 14.5, 4.7)<br>2.05 (brd, 14.5)	2.35 (brdd, 14.0, 5.4)<br>1.94 (brd, 14.0)	2.32 (dd, 14.3, 4.6)<br>2.00 (brd, 14.5)	20-CH_3	1.89 (s)
9-OH	5.45 (brs)	4.82 (brs)	5.69 (brs)		
10	4.06 (s)	3.98 (s)	4.04 (s)		
11	7.41 (s)	7.36 (s)	7.38 (s)		
13	1.59 (dq, 14.6, 7.3)<br>1.44 (dq, 14.3, 7.2)	1.70 (dq, 14.3, 7.3)<br>1.53 (dq, 14.3, 7.1)	1.28 (s)		
14	0.99 (t, 7.4)	0.90 (t, 7.2)			
16	3.62 (s)	3.58 (s)	3.64 (s)		

a) DMSO-d_6; b) CD_3OD; See Supplementary Information for NMR spectra. Assignments supported by 2D HSQC and HMBC experiments. *Atom numbering based on compound **1** for better comparison.

Method 1. Gene synthesis and plasmid construction for protein expression.

E. coli codon-optimized and synthesized *dnrF* sequence

<u>AGATCT</u>GCGCTGACCAAACCGGATGTGGATGTGCTTGTTGTAGGCGGCGGGCTTGGGGGTCTGAGCACGGCGCTGTTTCTAGC
ACGCCGCGGAGCTCGGGTGCTGCTGGTGGAACGCCATGCGAGCACGAGCGTGCTGCCGAAAGCGGCGGGTCAGAACCCGCG
CACCATGGAACTGTTTCGCTTTGGCGGCGTGGCGGATGAAATTCTGGCGACCGATGATATTCGCGGCGCGCAAGGCGATTTTA
CCATTAAAGTGGTGGAACGCGTGGGCGGCCGCGTGCTGCATAGCTTTGCGGAAAGCTTTGAAGAATTGGTTGGTGCTACCGAG
CAGTGCACCCCGATGCCTTGGGCGCTGGCTCCTCAAGATCGCGTGGAACCGGTGCTGGTGGCGCATGCGGCGAAACATGGCG
CGGAAATTCGCTTTGCGACCGAACTGACGAGCTTTCAAGCGGGCGACGATGGCGTTACCGCGCGCTTACGAGACTTAGGCACC
GGCGCGGAAAGCACCGTTTCTGCGCGCTATCTCGTGGCAGCGGACGGGCCGCGCAGCGCGATTCGCGAAAGCCTGGGCATTA
CCCGCCATGGCCATGGCACCCTGGCGCATTTTATGGGCGTGATTTTTGAAGCGGATCTGACCGCGGTGGTGCCGCCGGGCAG
CACCGGCTGGTATTATCTGCAGCATCCGGATTTTACCGGCACCTTTGGCCCGACCGATCGCCCGAACCGCCATACCTTTTATGT
GCGCTATGATCCTGAACGCGGAGAACGCCCGGAAGATTATACCCCGCAGCGCTGCACCGAACTGATTCGCCTGGCGGTGGATG

CGCCGGGCCTGGTGCCGGATATTCTGGATATTCAAGCGTGGGATATGGCGGCGTATATTGCGGATCGCTGGCGCGAAGGCCC
GGTCCTGCTGGTAGGGGATGCGGCGAAAGTGACACCCCCGACCGGCGGCATGGGCGGCAACACCGCGATTGGCGATGGCTTT
GATGTGGCGTGGAAACTGGCGGCGGTGCTGCGCGGCGAAGCGGGCGAACGCCTGCTGGATAGCTATGGCGCGGAACGCAGC
CTGGTGAGCCGCCTGGTGGTGGATGAAAGCTTAGCGATTTACGCCCAACGCATGGCGCCGCATCTCCTGGGCAGCGTACCGGA
AGAGCGCGGCACCGCGCAAGTGGTGCTGGGCTTTCGCTATCGCAGTACCGCGGTGGCGGCGGAGGATGACGATCCGGAACCA
ACTGAAGATCCGCGCCGCCCAAGCGGCCGCCCGGGCTTTCGCGCGCCGCATGTGTGGATTGAACAAGATGGCACCCGCCGCA
GCACCGTGGAACTATTCGGCGATTGCTGGGTGCTATTAGCAGCACCCGAGGGCGGGGCTTGGGGGCAAGCGGCCGCACGGG
CTGCGGCGGACTTAGGCGTGCGCCTGGACGTGCATTTGGTGGGTCGCGATGTTGCGGCGCCGTCGGGAGAGTTGACCCGCAC
ATATGGTATAGGTCGAGCCGGCGCGTCCTTGGTCCGCCCGGATGGCGTCGTGGCGTGGCGCACCGCGGTTGCTCCTGGTGCG
GAGGCCCAAGATCAGCTGAGCACCCTGCTGACCCGCCTGCTGGCGCGCTAAGAATTC

Synthesized gene sequence for *kstA15*
AGATCTGCGCTGACCAAACCGGATGTCCCTGACCGAGAACGCGGAACGGTCCCTGACCGACGACCGGACCCTGGCCGAGAAC
AAGGAGCGCTGCCTCCAGATGGTGGCCGCGTGGAACCGGTGGGAGCTGGACGGGATCATCAAGTACTGGGCCCCGGACGTCG
TCCACTACTCCGAGGACAAGGTGGTCGACACCGACGAGATGATCCGCCGGATGGAGGGCGGCATCCAGGCGTTCCCCGACCT
GCACCTCGACGTGAAGAGCATCATGGCCGAGGAGGACCGGGTCATCCTGCGGATCACCATCACGGCCACCCACAAGGGCCGG
TTCGGCGACCTCGCCCCGACCAACCGGAAGGTCGCCTGGCACATCGTCGAGGAGCTGCGCTTCGTCGACGGCAAGGTGGTCG
AGCACTGGGACGTCATGAACTACCTGCCGATGCTCAAGGAGCTCGGCAAGGTGCCGGCGGACGTCTGAGAATTC

Synthesized gene sequence for *kstA16*

AGATCTGCGCTGACCAAACCGGATGACGATCCTGGTGACGGGCGCGACCGGACACGTCGGCCGACACGTGGTCACGGAGCTG
CTGGCCGGGGGCCACGCGGTGCGGGCGATGACCCGCGACCCGCAGCGCGGCCGGTTCCCGGCCGGCGTCGAGGTGGTCCG
GGGCGACCTGACCGAGCCCGCGGGGCTGGCGGACGCCCTGCGCGGCGTGGAGAAGATGTACCTGTTCCCGGTGCCGGACAC
CGCCGTGGAGGTGGTGCGGGCCGCGGAGCGGGCCGGCGTCCGGCACGCGGTGGTGCTCTCCTCCACCTCCGCCGACGACG
CCACGAACTTCAGCGGCGTCTACCACCGCACCGTGGAGCGGGCGGTGGAGGAGAGCGGCCTGGACTGGACGTTCGTCCGGC
CGGACGAGTTCGCCACGAACCTGCTGTGGAAGTGGGGCCACTCCGTGCGTACGGAGGGGGTGGTGCGGGGGCCGTACCCGC
AGGCCCGGCGGGCGCTGATCCACGAGGTGGACATCGCGGCGGTGGTCGCGCTCGCGCTGACCGAGGCTGGCCACGCCGGCC
AGGTGTACGACCTGACCGGCCCGGAGGCGCTGGACCAGCGCACCCAGGTGGCGCAGCTCGCCGAGGCCACCGGACGTCAGA
TCCGCTTCGAGGAGGTCAGCCCGGCGGCGGCCCGCGCCGAGCTGACCGCGTACATGCCGGAGCCGGTGGTCGACATGGTGC
TCGGCTACCTGGCCGACTCCGTGGACCGCCCGCCGGTGGTGCTGCCGACGGTGGAGAAGCTGACCGGCCGACCGGGGACGC
CGTTCGCCCGCTGGGCGGCCGACCACGCCGACGAGTTCCGCGCGGCCTGAGAATTC

Synthesized gene sequence for *kstA10*

AGATCTGCGCTGACCAAACCGGATGATCCTCGTCACCGGCGCGACCGGAAAGGTGGGCAGGCACGTGGTGGCCCGCCTGCGC
GAGGCGGGCCACCGGGTCCGCGCCCTGAGCCGCGACCCCGCGCGGGCCGACCTGCCGCCCGACGTCGAGGTGGTGGCCGG
CAGCCCCGGCGACGCCGACGCGTCCGCCGCCGCGCTGGCCGGCGTGGACGCCGCGTTCGTCTGCCTGGTGGGCGACGTGGA
GGCCGGGGCGCGGCCCTTCGCCGAGGCGATCCGGCGGGCCGGCGGCGTGCGCCGGCTGGTGCTGCTGTCGTCGTCGGCCG
TGCTGCACCCCGTGCGGCACCGCATCGGCGACGAGCACCGCGCCGCCGAGGAGCTGATCGGCGCGGCGGCCCCGGACGCCA
CCCTGCTGCGGCCCGGCCCGTTCCACACCAACTCGCTGTGGTGGGCGAAGTCCATCCGGGAGCAGGGCCGGGTGCGTTGCCT
GGTGGGCAACAACCCCGGCGCCCCCGTCGACCCGGACGACCTGGCCGCGGTGGCCGTGGCGGCGCTGACCTCCGGCGAGCA
CGCCCGCCGCCGGTACGAGCTGACCGGCCCGGAGGTGCTCACCTCCGCCGAGCAGGTCCGCGTCATCTCCGACGTGCTCGG
CCGGGACCTCGACTTCGAGGTGGCCGCCCCCGAGGAGGTGGTGGCGACGTTCGCCTCGATCACCGGCGACCGACCGGCCGC
GGAGACCAACGTCGCGGCGCTGCACAGCCCACGGGTGCCCTGGGGGCGCACCAACGGCACCGTCGAGCGGCTGCTCGGCCG
CAAGCCGCGCACCTTCCGCGACTGGGCGGCCGAGCACGCCCACCTGTTCCGATGAGAATTC

Synthesized gene sequence for *kstA11*

AGATCTGCGCTGACCAAACCGGATGGCCGACAGCAACCGCACCATCCTCGTCACCGGGGCCACCGGCACCCAGGGCGGCGCG
ACCGTCCGGGCGCTGCTCGCCCGGGGCCGCCCGGTGCGCGCCCTGGTCCGCGACCCGGGCACCGACGCCGCGCGGGCGCT
CGCCGCGGCCGGCGTGTCGCTGGTGACCGGCGACCTCAACGACCAGGCCTCCCTGCGGGCGGCGATGGCCGACGTGCACGG
GGTGTTCAGCGTGCAGACCTTCATGACCCCCGGCGGGCTGGGCGCGGAGCTGCGCCAGGGCCGCGCGGTCGCCGACGCTGC
GGCGGCCACCGGCGTACGCCACGTCGTCTACAGCTCGGTCGGCGGTGCGGACCGCGCCAGCGGGGTGCCGCACTTCGAGAC
CAAGTGGACGATCGAGCGGCACCTGCGGTCGCTGGGCGTGCCCACCACGGTGCTGCGCCCCACCTTCTTCATGGACAACTTC
GCCGCCTGGGGCCCCCAGGCGGTCGACGGCACCCTGGTGGTGCGGCTGCCGCTGAAGCCGCAGACCCGGGTGCAGCTCATC
GCCGCCGAGGACATCGGGGTGTTCGCCGCGACCGCCTTCGACGACCCGGACACCTACGTCGGGGCGGCCCTCGAACTGGCC
GGCGACGAGCTGACCGGCCCGGAATTGGCCGCCCGCTTCGGCGAGCTGGCGGGGATGCCCGCCCGGTTCGAGGAGCGCAGC
CTCGACGAGGCGGCGGCCGACCCGTGGATCCCGTACAGCCACGAGATCGCGGTGATGTTCGAGTGGTTCCAGACCGACGGGT
ACGCCGCCGACATCGCGGCGCTGCGGGCCCGCCACCCGGGGCTGCGCACCTTCGCCGACTGGCTGCGCGCGATCGGCTGGC
GCGTCCCGGCGCCCGCCGCCGGGTGAGAATTC

Strain and Plasmid construction

Recombinant plasmids carrying the N-poly-His tagged genes for *dnrF, kstA15, kstA16, kstA10,* and *kstA11* were constructed as follows. The coding sequence for *dnrF* was codon optimized based on the codon usage in *E. coli* and synthesized by Genewiz (Suzhou, China). The genes for *kstA15, kstA16, kstA10,* and *kstA11* were synthesized using the native sequence. The synthesized genes were flanked by *Bgl*II/*Eco*RI sites and cloned into the pBAD/HisB backbone by using the same restriction sites.

Method 2. Protein production and purification.

The expression and purification of the enzymes used in this paper was carried out as follows. Briefly, protein was expressed in *Escherichia coli* TOP10 cells carrying the pBAD/HisB with the coding genes. Protein purification was carried out through a polyHis-tag affinity chromatography with TALON SuperFlow resin and PD-10 desalting columns. Enzymes were concentrated using Pierce™ Protein Concentrators PES (10K MWCO). The purity of all the enzymes was checked by SDS-PAGE.

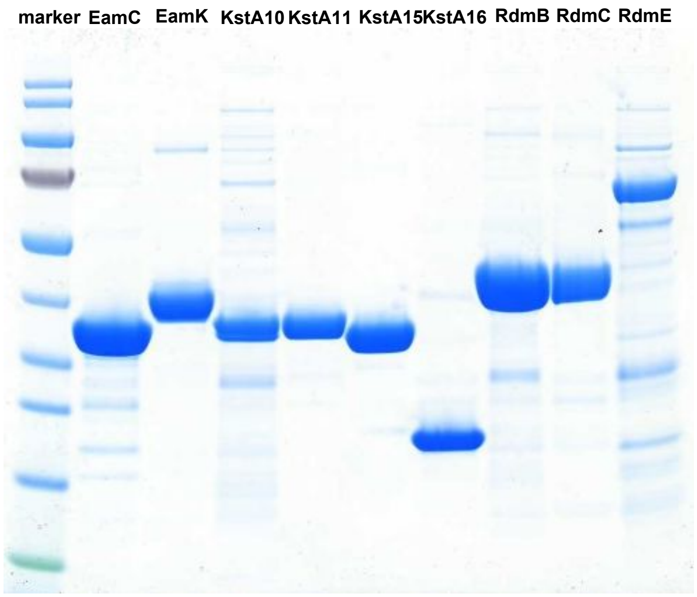

SDS-PAGE analysis of purified enzymes.

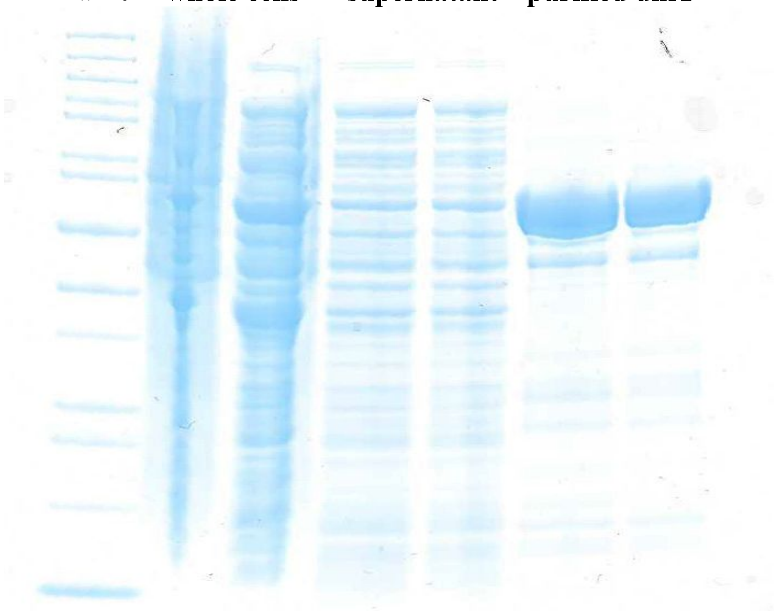

SDS-PAGE analysis of purified DnrF enzyme.

Method 3. Substrate purification for enzymatic assays.

To generate compound **1** as the substrate for enzymatic reactions, aclacinomycin A was hydrolyzed as described before [9]. The extract was dried with a rotary evaporator and resuspended in Methanol. The Shimadzu Propminence Preparative HPLC equipped with a Phenomenex Kinetex Phenyl-Hexyl column (5 μm, 100 Å, 250 × 21.2 mm) was used to further purify the extract to obtain **1** in >95% purity. Method: Solvent A: 15% CH3CN/0.1% FA; solvent B: CH3CN; flow rate: 20 mL/min; 0-2 min, 0% B; 2-3 min, 0-30% B; 3-13 min, 30-70% B; 13-15 min, 100% B; 15-20 min, 0% B.

Compounds **2**, **3**, and **4** were respectively produced and purified from *S. coelicolor* M1152matABΔ::pA2A5S6 [2], *S. coelicolor* M1152matABΔ::pS2S5S6 [2], and *S. albus* Δ*snogE* [10]. The strains were grown in 1 – 2 L of SG-TES media. The fermentation was carried out for 7 days in an orbital shaker at 30 degrees Celsius at 200 RPm. The fermentation was harvested after the cultivation of 7 days, centrifuged, and 20g/L LXA-1180 resin (SUNRESIN) was added to the culture supernatant to bind the compounds overnight with shaking at 100rpm. Resin was then collected and eluted by methanol to get the crude extracts. The extracts were dried, resuspended in a minimal volume of methanol, and purified by the preparative HPLC with the same method used for compound **1**.

Method 4. Enzymatic reactions with purified enzymes and substrates.

The reactions were performed in 200μL reaction buffer (50mM phosophate, 50mM NaCl, pH7.5). The substrates were added to the reaction buffer to get a final concentration between 10-50μM. The RdmE/DnrF reactions were performed with incubation of 10 μM RdmE/DnrF, 0.5 mM NADPH overnight at 30°C. For EamC+RdmB reactions, 5 μM EamC, 5 μM RdmB, 10 μM DTT and 400 μM SAM were reacted with the substrates at 30°C for 4 h. For the DnrF+EamC+RdmB reactions, the overnight products from DnrF reactions were extracted with chloroform and dried *in vacuo*, then 5 μM EamC, 5 μM RdmB, 10 μM DTT and 400 μM SAM were added and incubated in the new reaction buffer at 30°C for 4 h. KstA15+A16 reactions were achieved with a reaction at 30°C for 1 h, containing 10 μM KstA15, 10 μM KstA16, and 0.5 mM NADPH. To get obtain KstA15+A16+A10+A11 products, products from KstA15+A16 reactions were extracted with chloroform and dried *in vacuo* to get the substrate for next step. 10 μM KstA10, 10 μM KstA11, 0.5 mM NADPH and fresh reaction buffer were added and incubated at 30°C for 2 h to get the final product.

All reactions were checked by UHPLC (Shimadzu Nexera LC-40 system with a diode array detector) using a Phenomenex Kinetex Phenyl-Hexyl column (2.6 μm, 100 Å, 4.6 × 100 mm). Method: Solvent A: 15% CH3CN/0.1% FA; solvent B: CH3CN; flow rate: 0.5 mL/min; 0-2 min, 0% B; 2-20 min, 0-40% B; 20-24 min, 100% B; 24-29 min, 0% B.

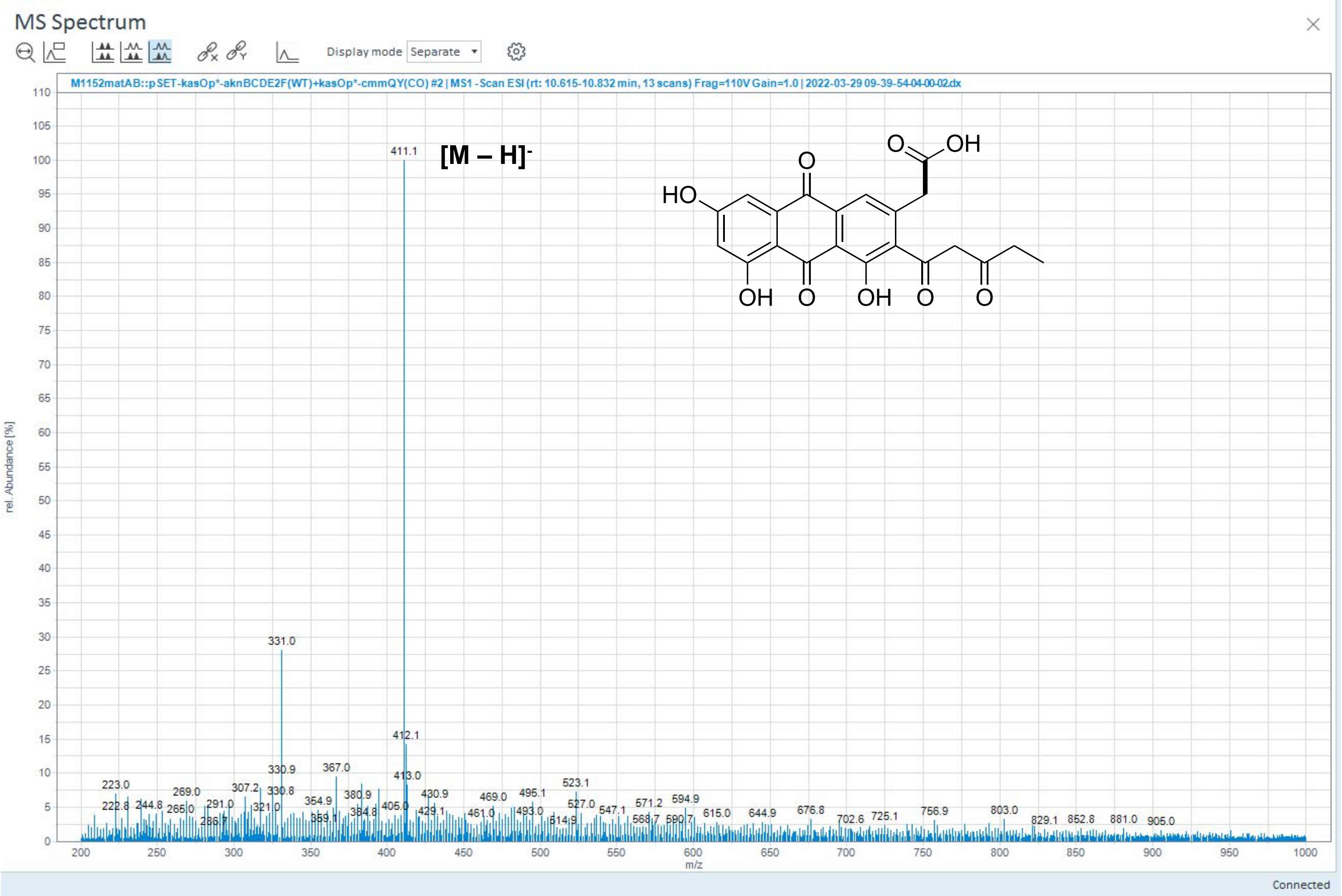

Figure S1. Mass spectrum of 2-hydroxy-aklanonic acid (**9**).

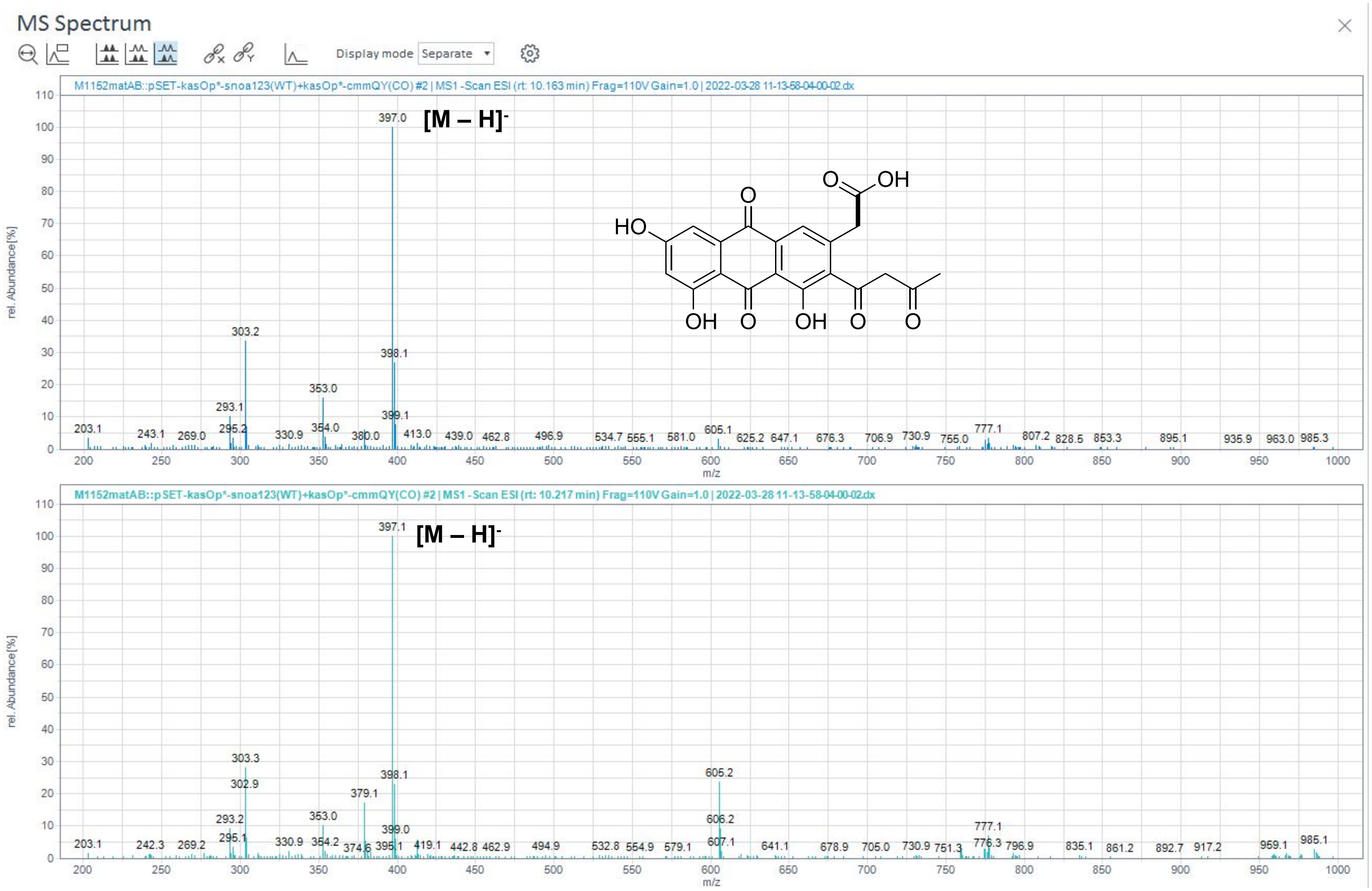

Figure S2. Mass spectrum of 2-hydroxy-nogalonic acid (**10**).

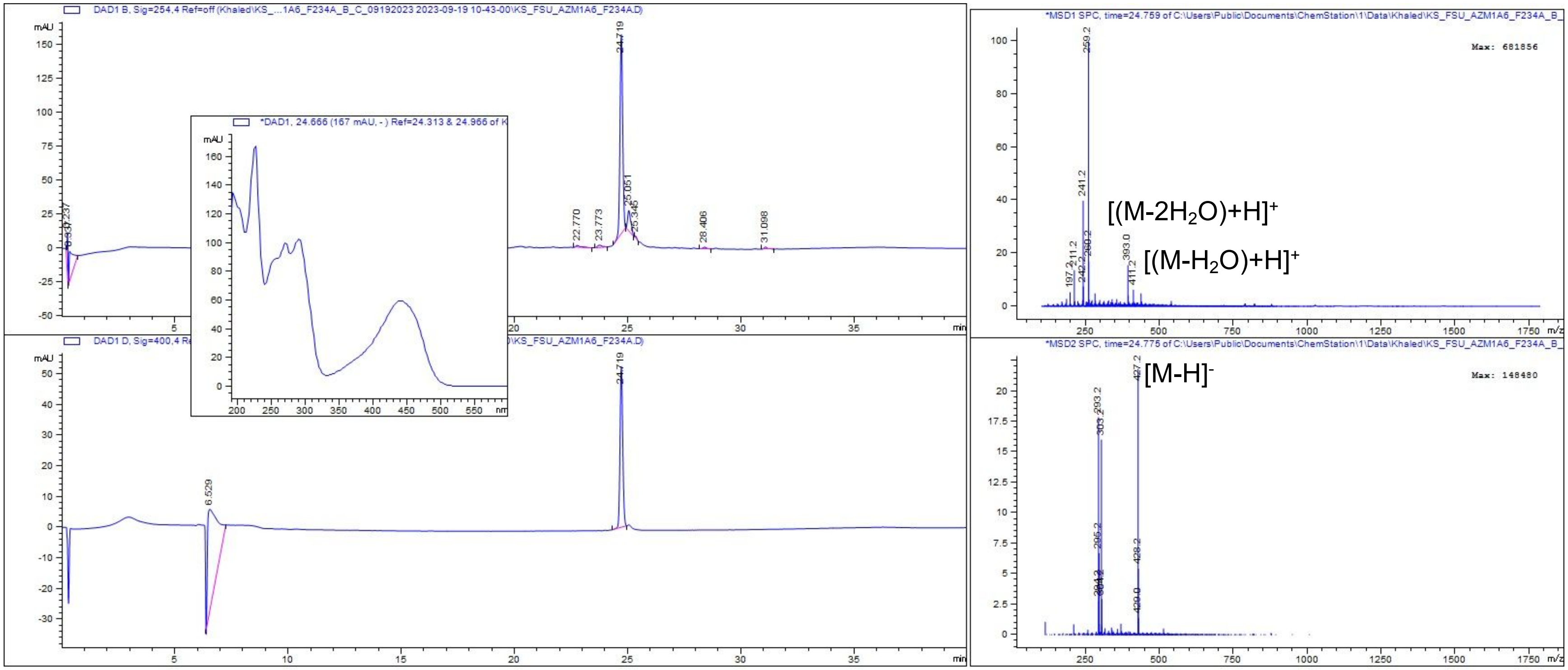

Figure S3: HPLC-MS analysis of 2-hydroxy-aklavinone (**11**). HPLC-conditions: solvent A: H_2O/0.1% FA; solvent B: CH_3CN; flow rate: 0.5 mL min^{-1}; 0-30 min, 5-100% B; 30-35 min, 100% B; 35-36 min, 100-5% B; 36-40 min, 5% B; Phenomenex NX-C18 column (250 × 4.6 mm, 5 μm); 254 nm, 280 nm, 400 nm. UV-vis inset of full wavelength scan (190-600 nm).

**Sample Name**	CN-579-A7M1A6-F234A1	**Position**	P1-C6	**Instrument Name**	Instrument 1
**User Name**		**Inj Vol**	10	**InjPosition**	
**Sample Type**	Sample	**IRM Calibration Status**	Success	**Data Filename**	CN-579-A7M1A6-F234A1.d
**ACQ Method**	Zheng_AQC ACC ND short_Pos.m	**Comment**		**Acquired Time**	10/10/2023 6:24:53 PM (UTC-04:00)

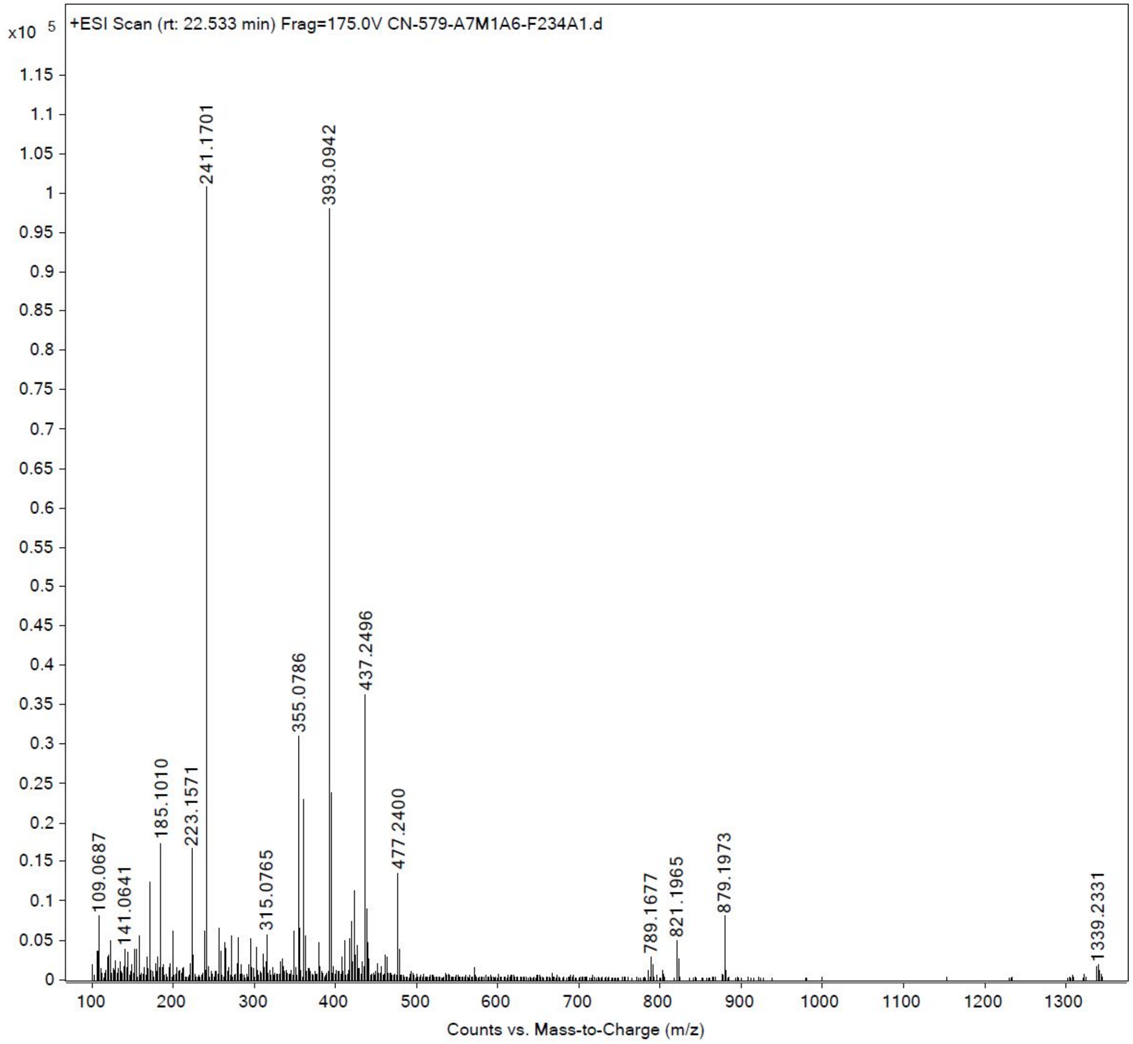

Figure S4. (+)-HRESI-MS spectrum of 2-hydroxy-aklavinone (**11**).

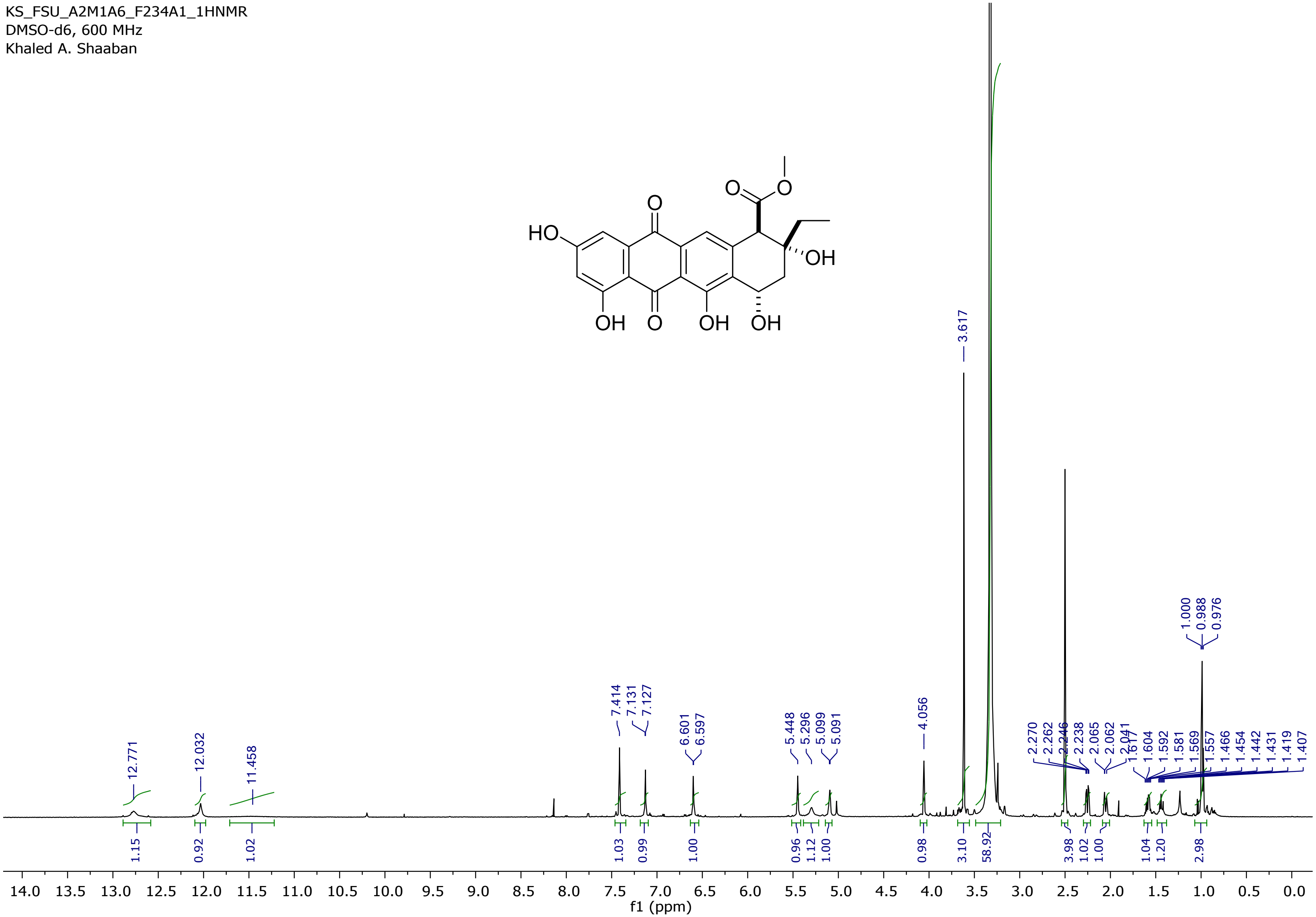

Figure S5. ^{1}H NMR spectrum (DMSO-*d6*, 600 MHz) of 2-hydroxy-aklavinone (**11**).

KS_FSU_A7M1A6_F234A1_1HNMR
DMSO-d6, 500 MHz
Khaled A. Shaaban

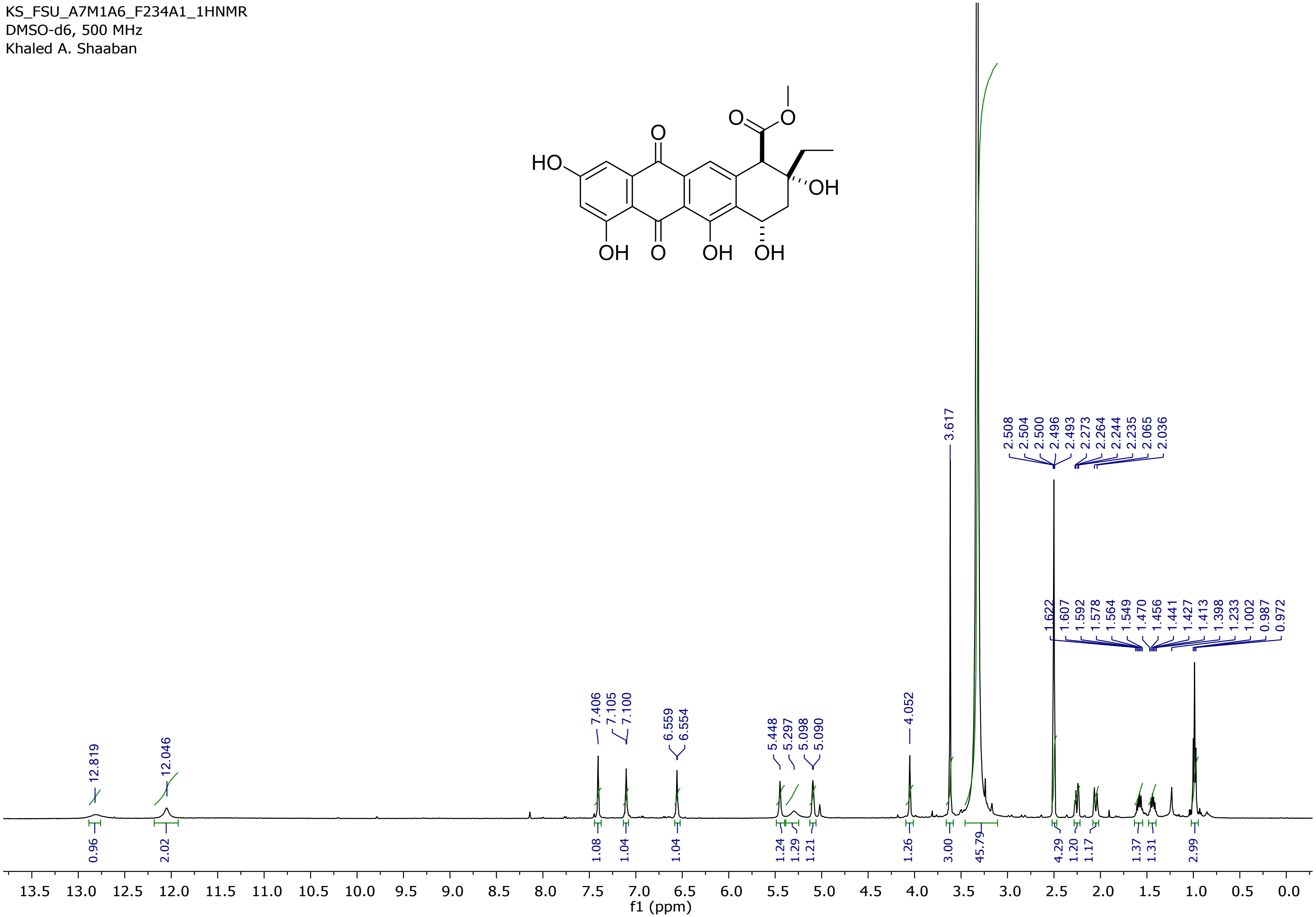

Figure S6. ^{1}H NMR spectrum (DMSO-*d6*, 500 MHz) of 2-hydroxy-aklavinone (**11**).

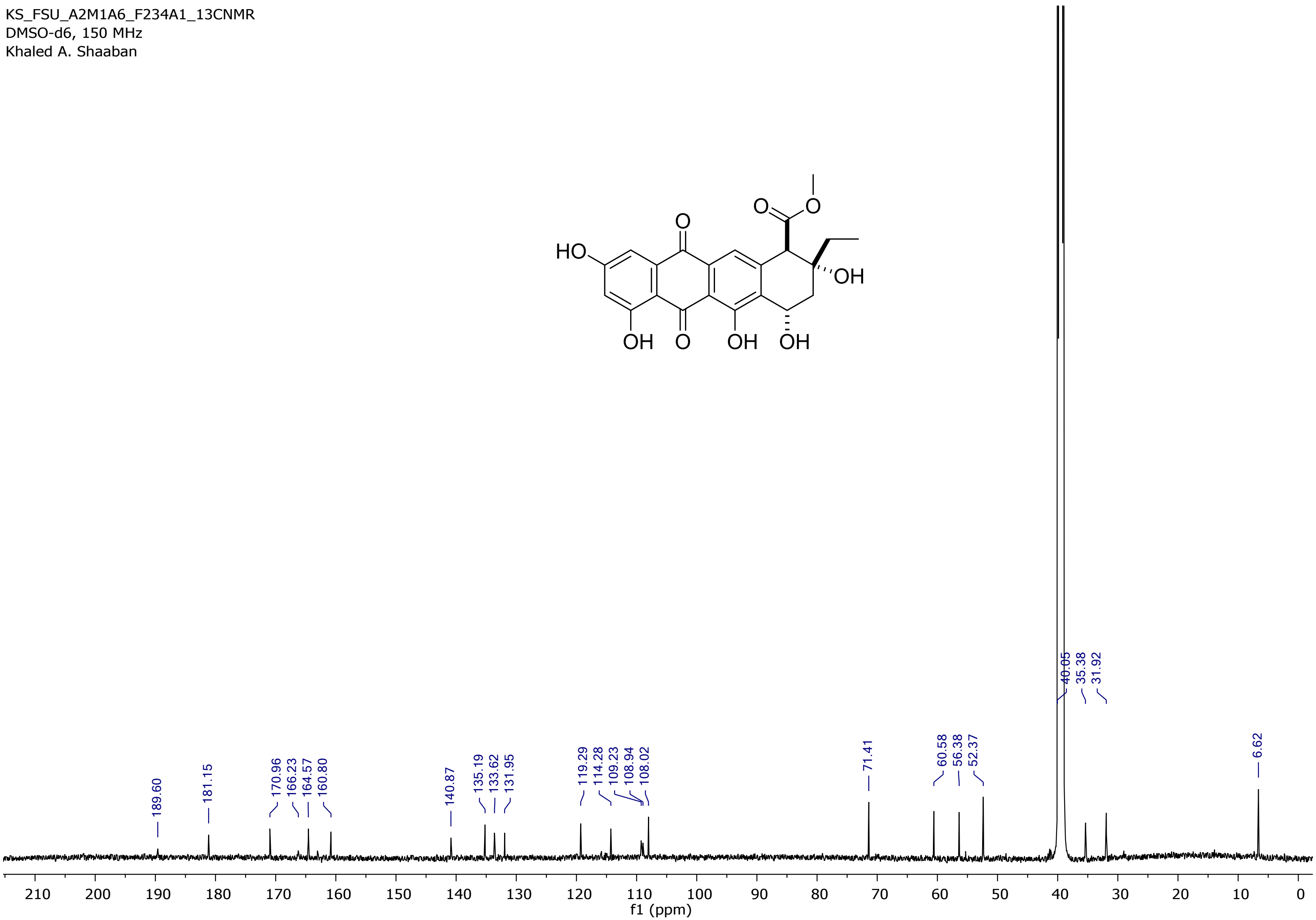

Figure S7. ^{13}C NMR spectrum (DMSO-*d6*, 150 MHz) of 2-hydroxy-aklavinone (**11**).

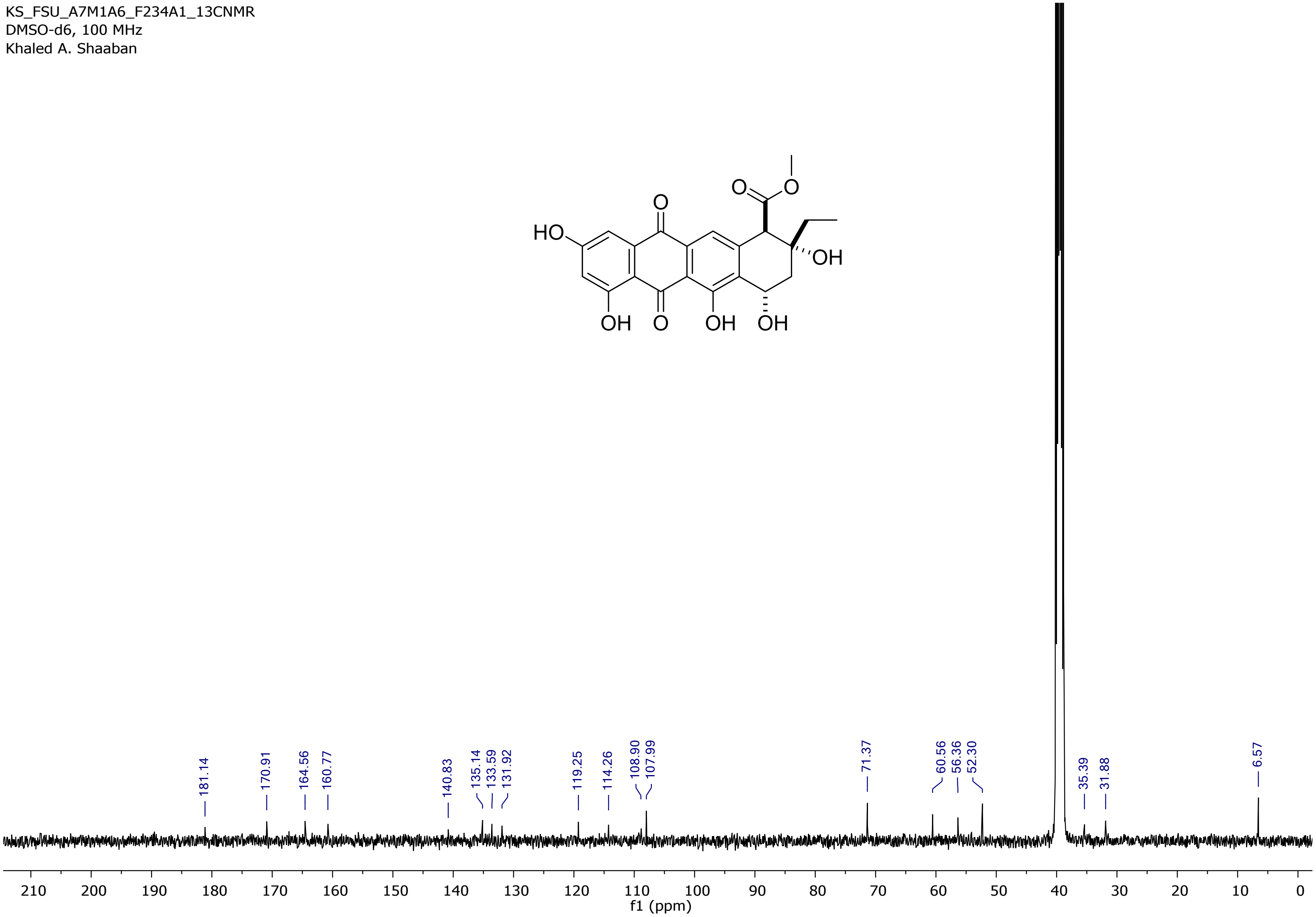

Figure S8. ^{13}C NMR spectrum (DMSO-*d6*, 100 MHz) of 2-hydroxy-aklavinone (**11**).

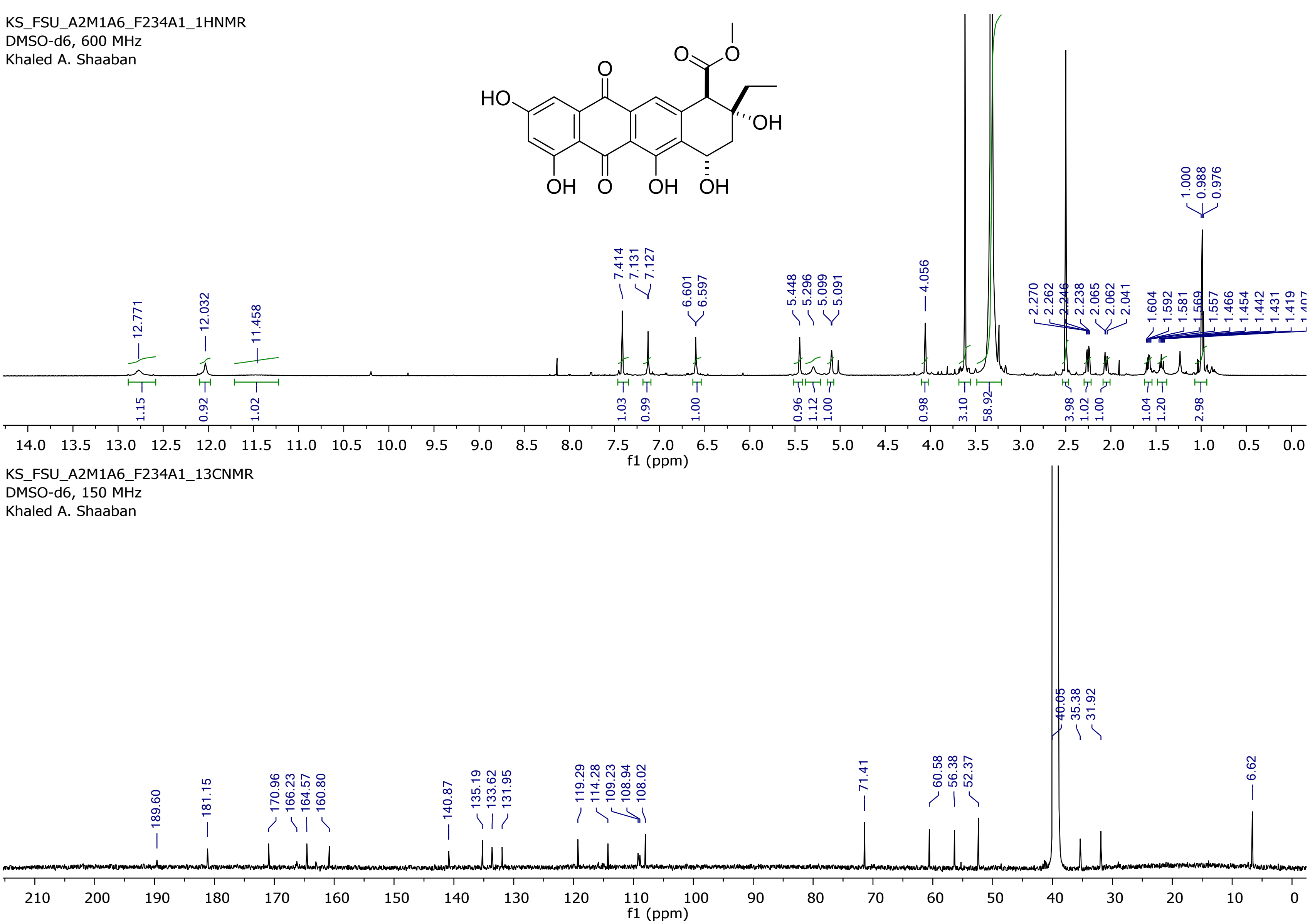

Figure S9. ^{1}H (DMSO-*d6*, 600 MHz) and ^{13}C (DMSO-*d6*, 150 MHz) NMR spectra of 2-hydroxy-aklavinone (**11**).

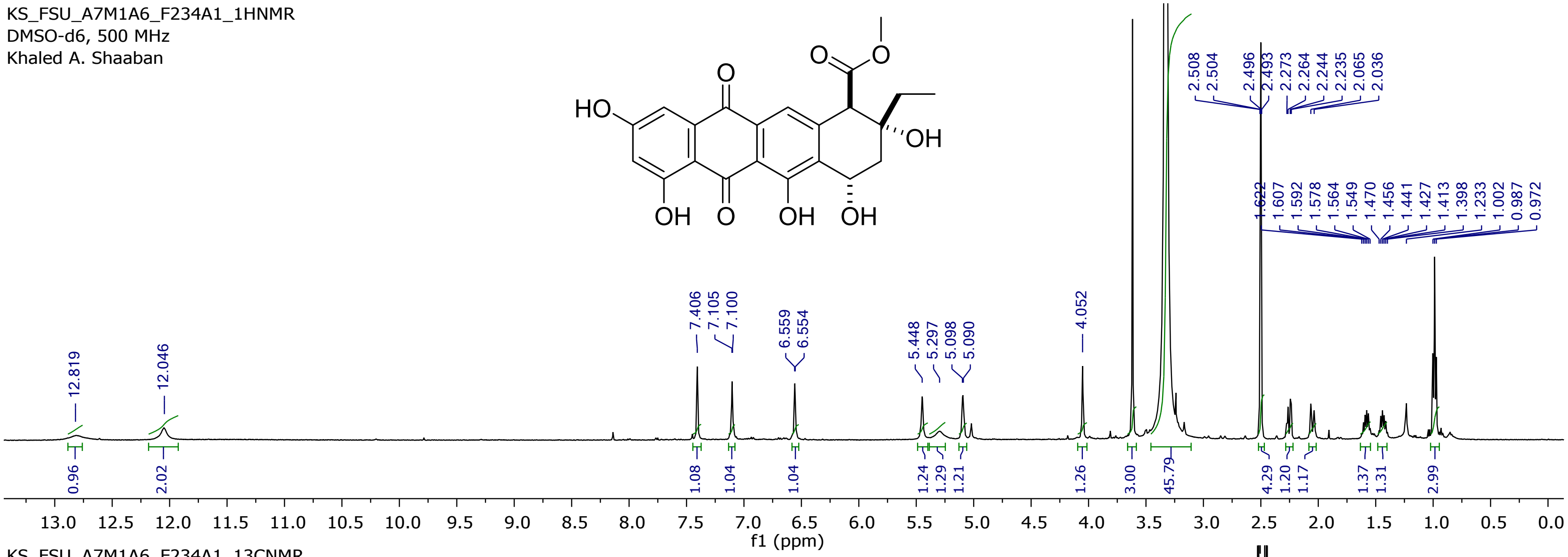

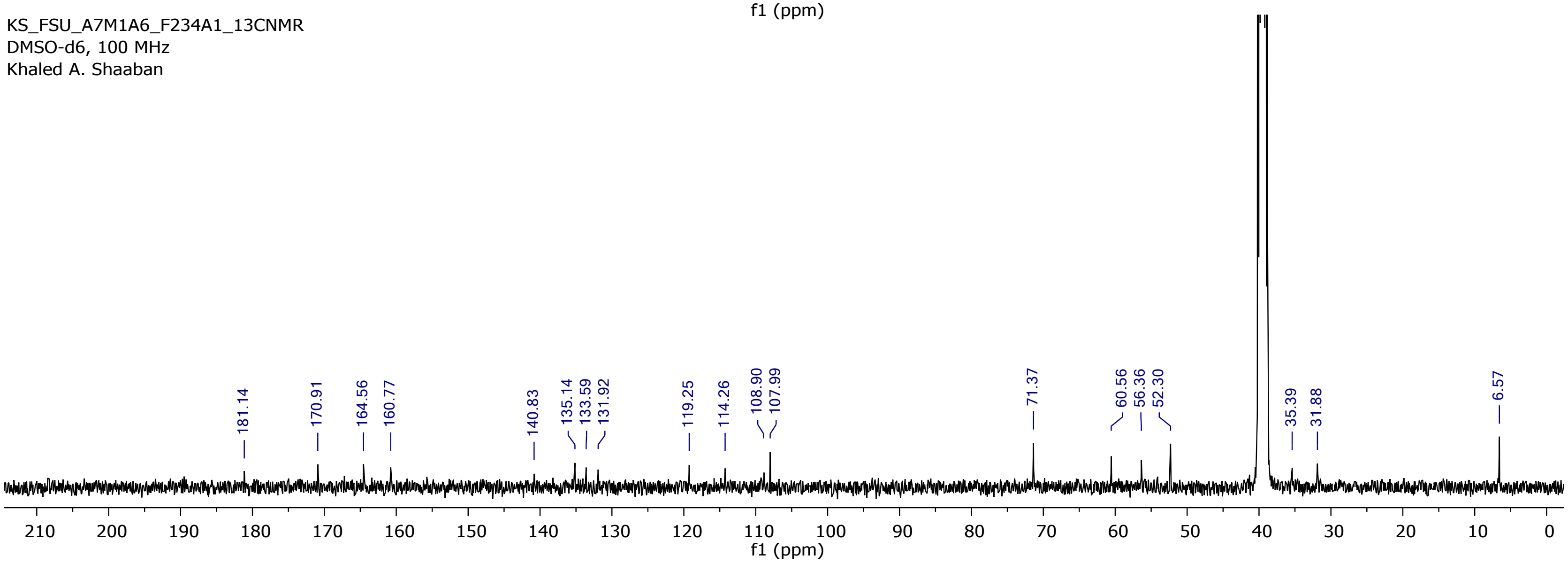

Figure S10. ${}^{1}H$ (DMSO-*d6*, 500 MHz) and ${}^{13}C$ (DMSO-*d6*, 100 MHz) NMR spectra of 2-hydroxy-aklavinone (11).

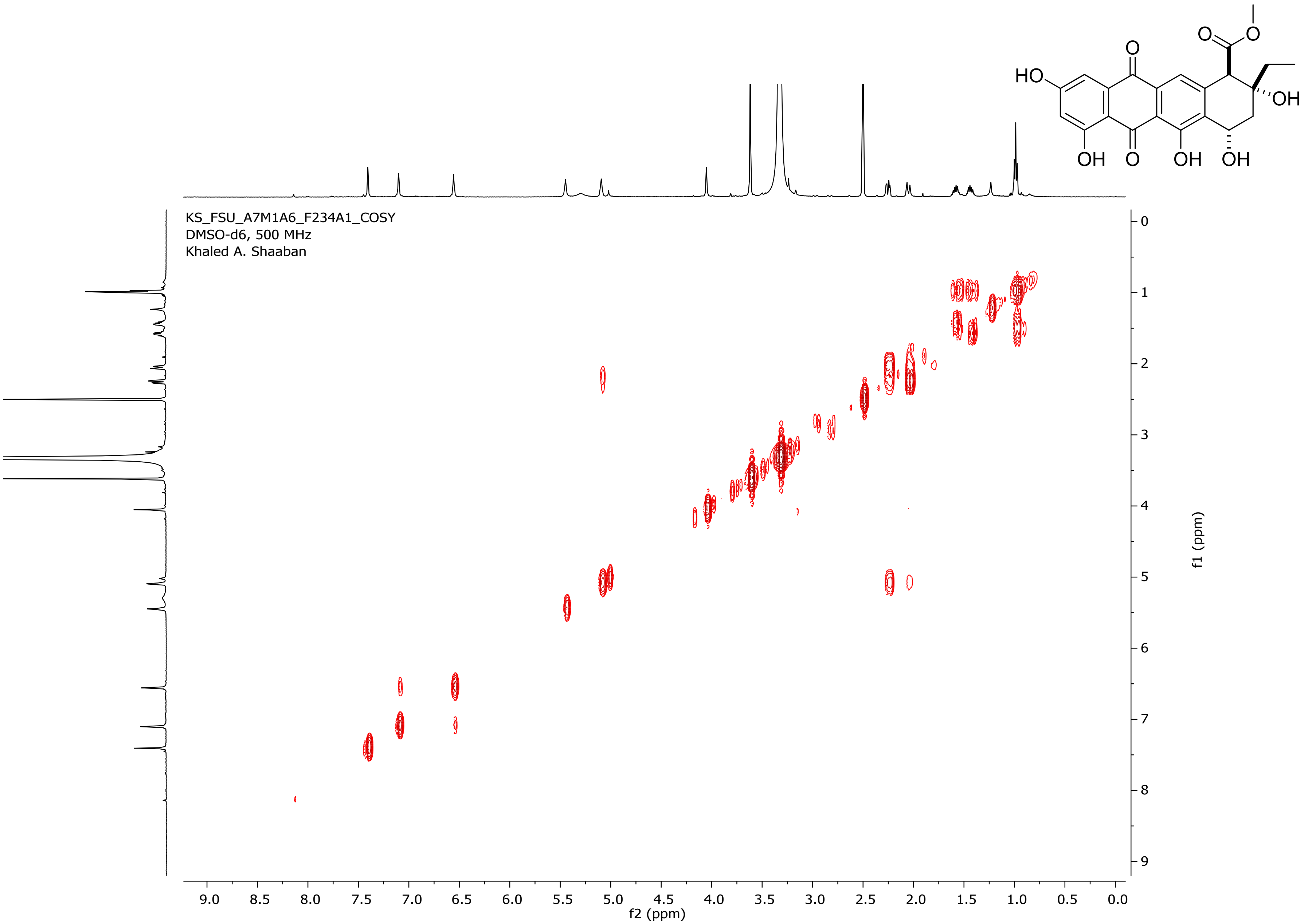

Figure S11. ^{1}H,^{1}H-COSY spectrum (DMSO-*d6*, 500 MHz) of 2-hydroxy-aklavinone (**11**).

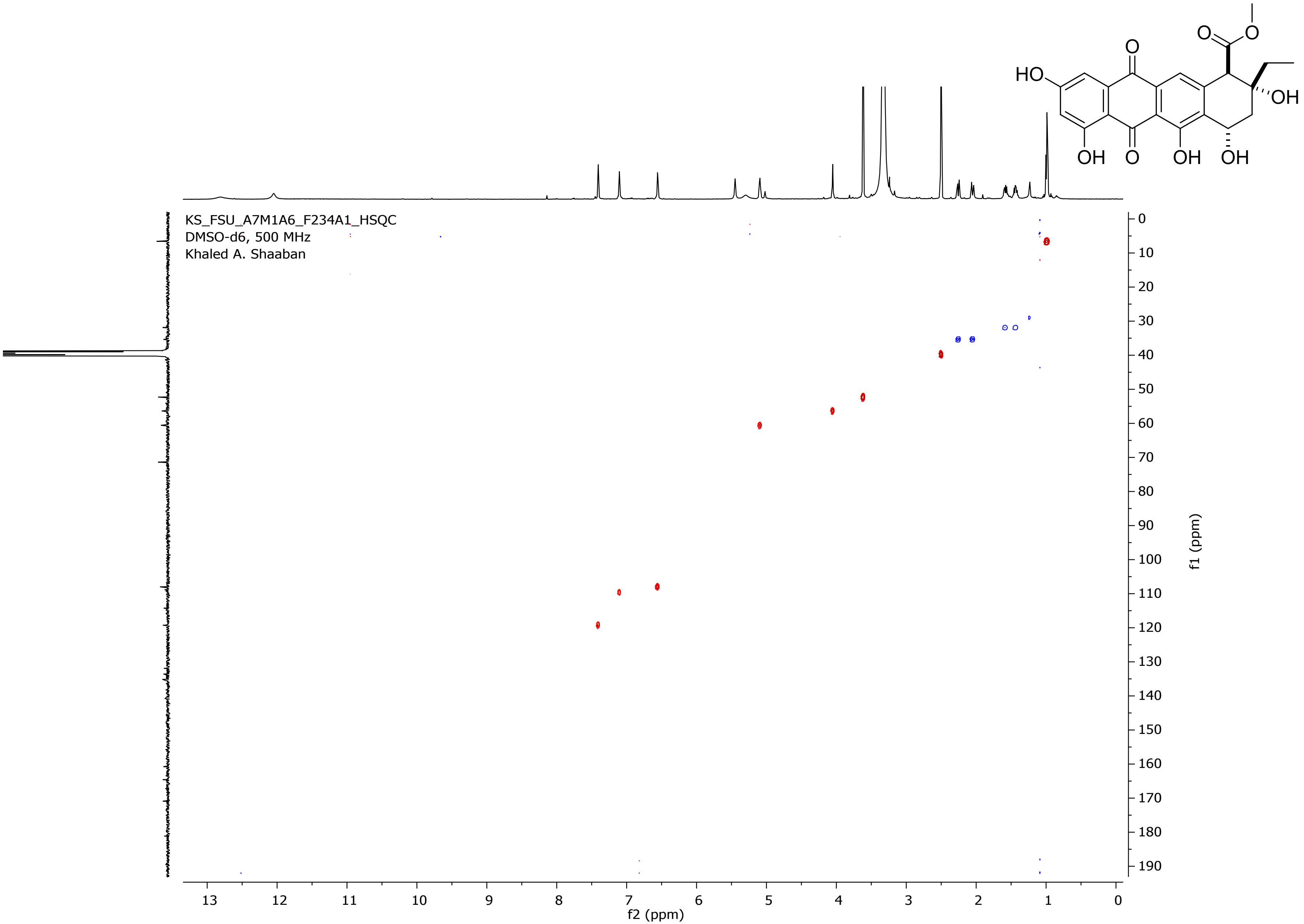

Figure S12. HSQC spectrum (DMSO-*d6*, 500 MHz) of 2-hydroxy-aklavinone (**11**).

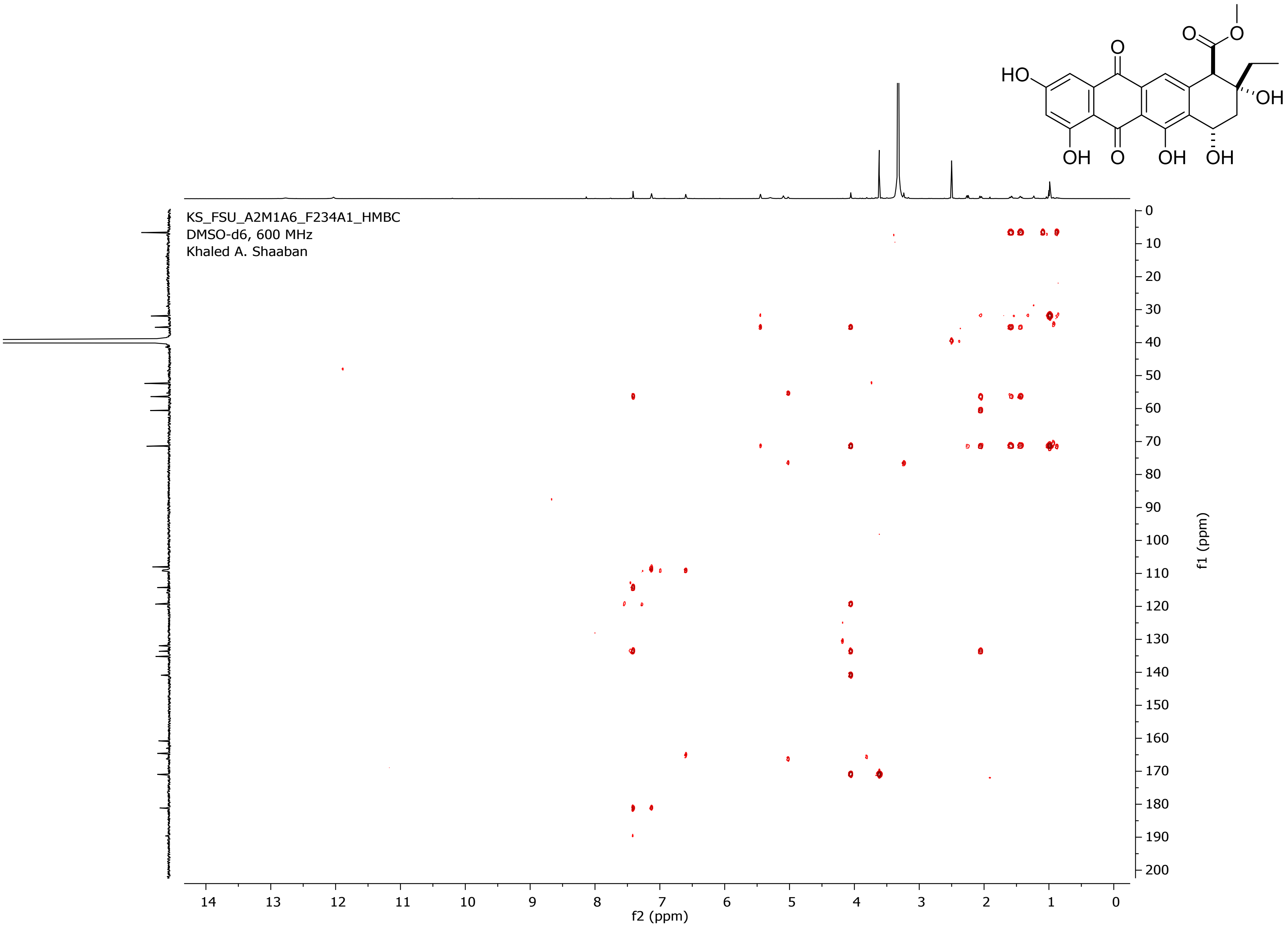

Figure S13. HMBC spectrum (DMSO-*d6*, 600 MHz) of 2-hydroxy-aklavinone (**11**).

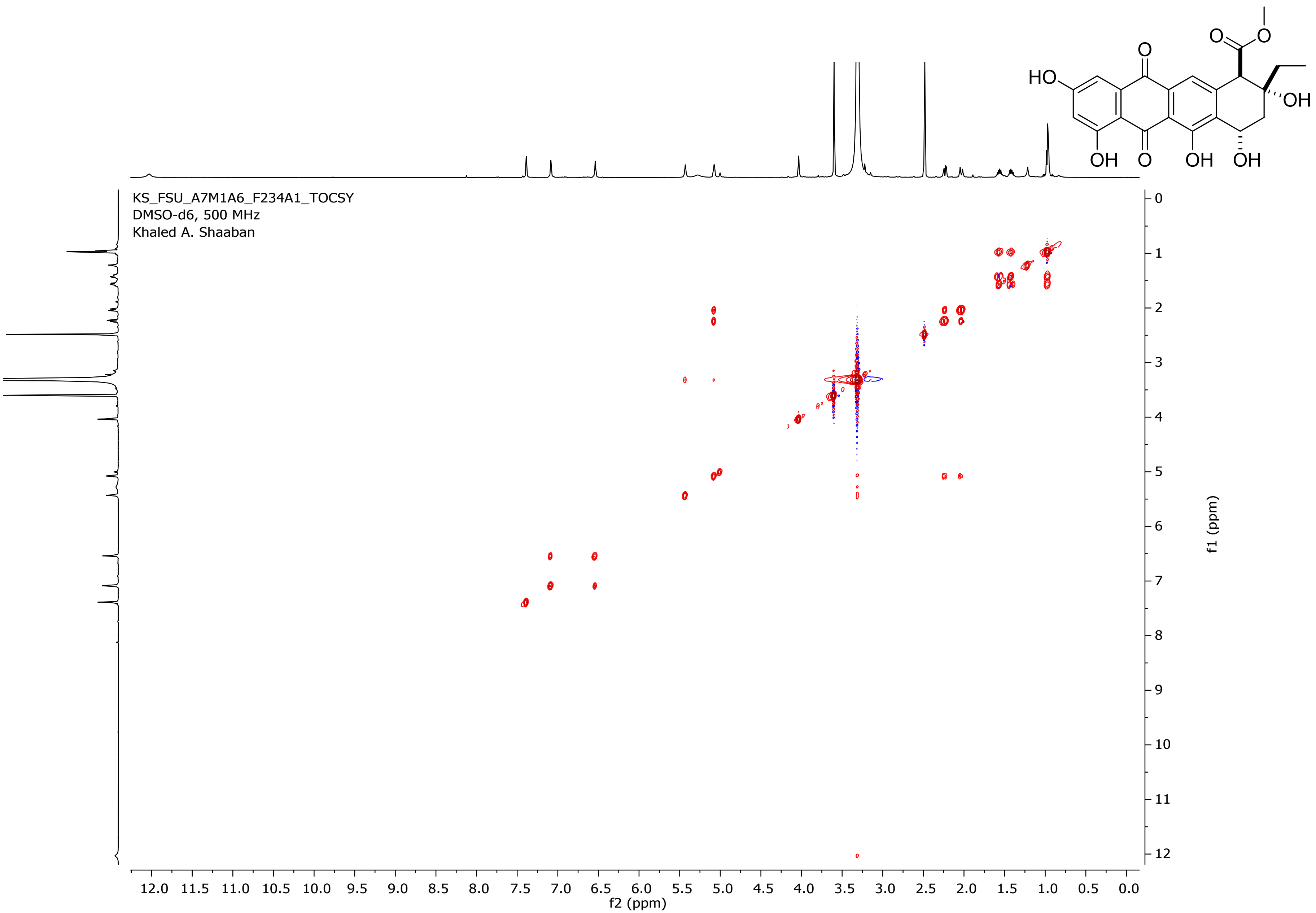

Figure S14. TOCSY spectrum (DMSO-*d6*, 500 MHz) of 2-hydroxy-aklavinone (**11**).

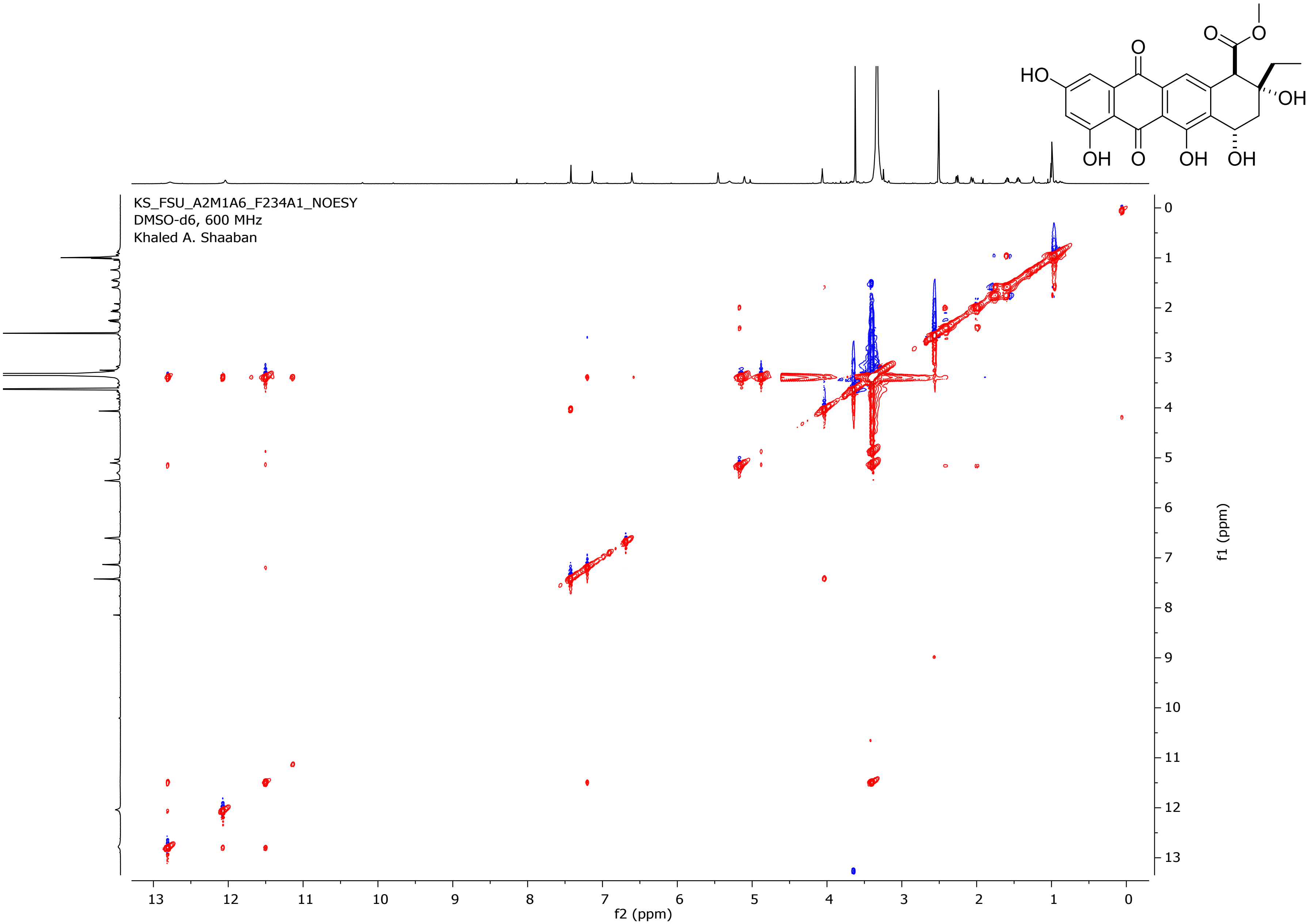

Figure S15. NOESY spectrum (DMSO-d_6, 600 MHz) of 2-hydroxy-aklavinone (**11**).

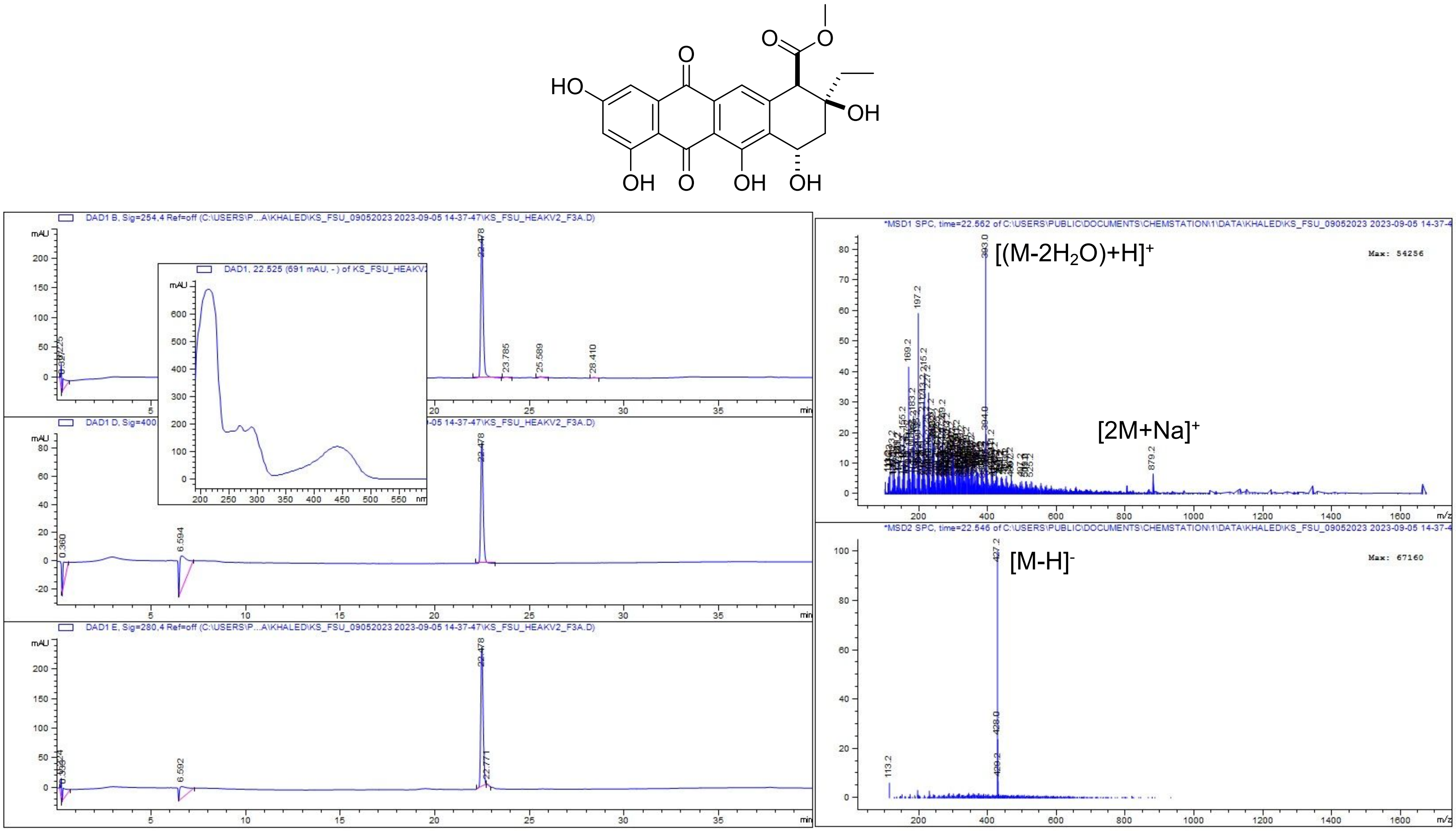

Figure S16: HPLC-MS analysis of 2-hydroxy-9-*epi*-aklavinone (**12**). HPLC-conditions: solvent A: H_2O/0.1% FA; solvent B: CH_3CN; flow rate: 0.5 mL min^{-1}; 0-30 min, 5-100% B; 30-35 min, 100% B; 35-36 min, 100-5% B; 36-40 min, 5% B; Phenomenex NX-C18 column (250 × 4.6 mm, 5 *µ*m); 254 nm, 280 nm, 400 nm. UV-vis inset of full wavelength scan (190-600 nm).

**Sample Name**	CN574-HEAKV2-F3A	**Position**	P1-C5	**Instrument Name**	Instrument 1
**User Name**		**Inj Vol**	10	**InjPosition**	
**Sample Type**	Sample	**IRM Calibration Status**	Success	**Data Filename**	CN574-HEAKV2-F3A.d
**ACQ Method**	Zheng_AQC ACC ND short_Pos.m	**Comment**		**Acquired Time**	10/10/2023 5:55:49 PM (UTC-04:00

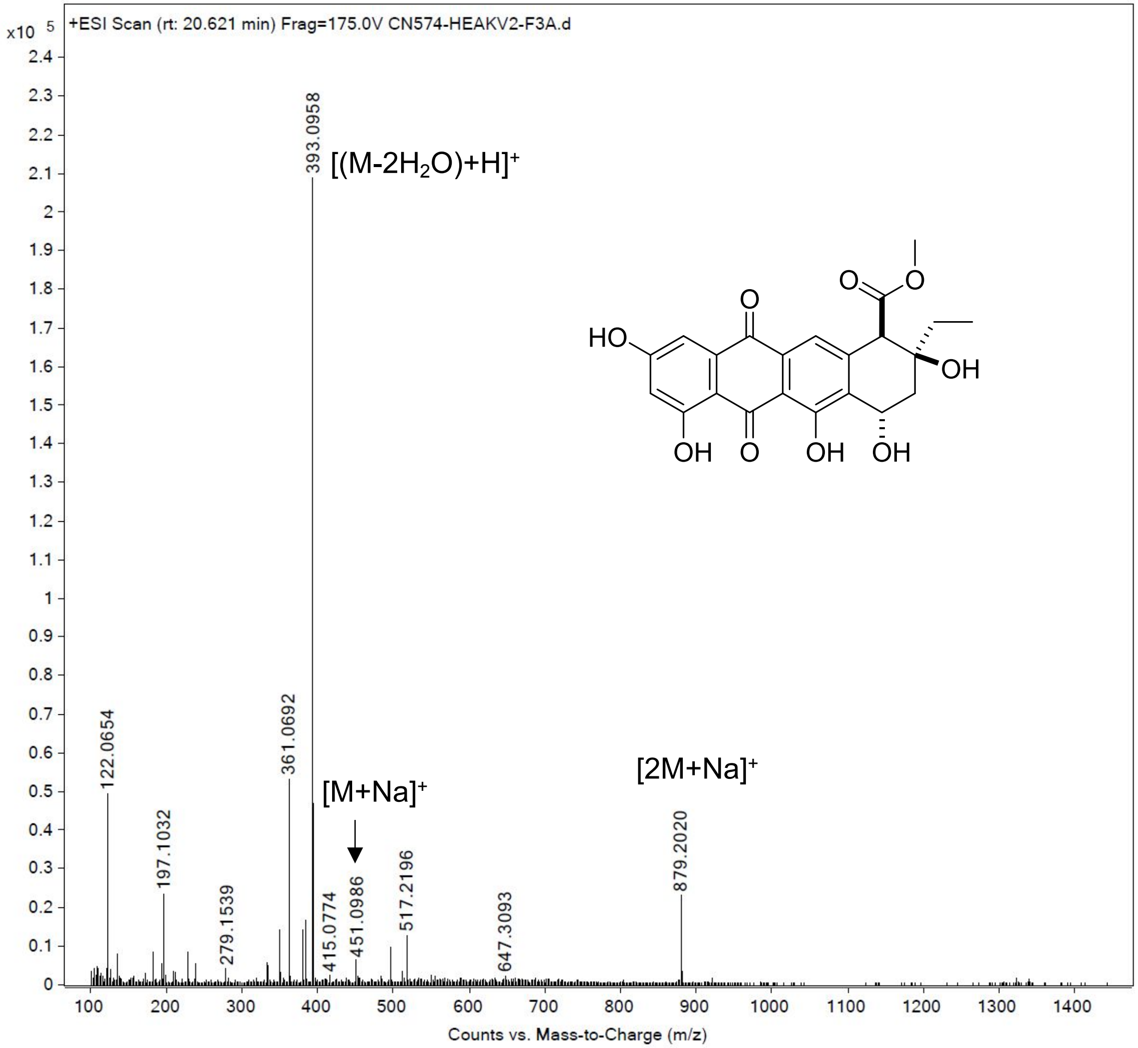

Figure S17. (+)-HRESI-MS spectrum of 2-hydroxy-9-*epi*-aklavinone (**12**).

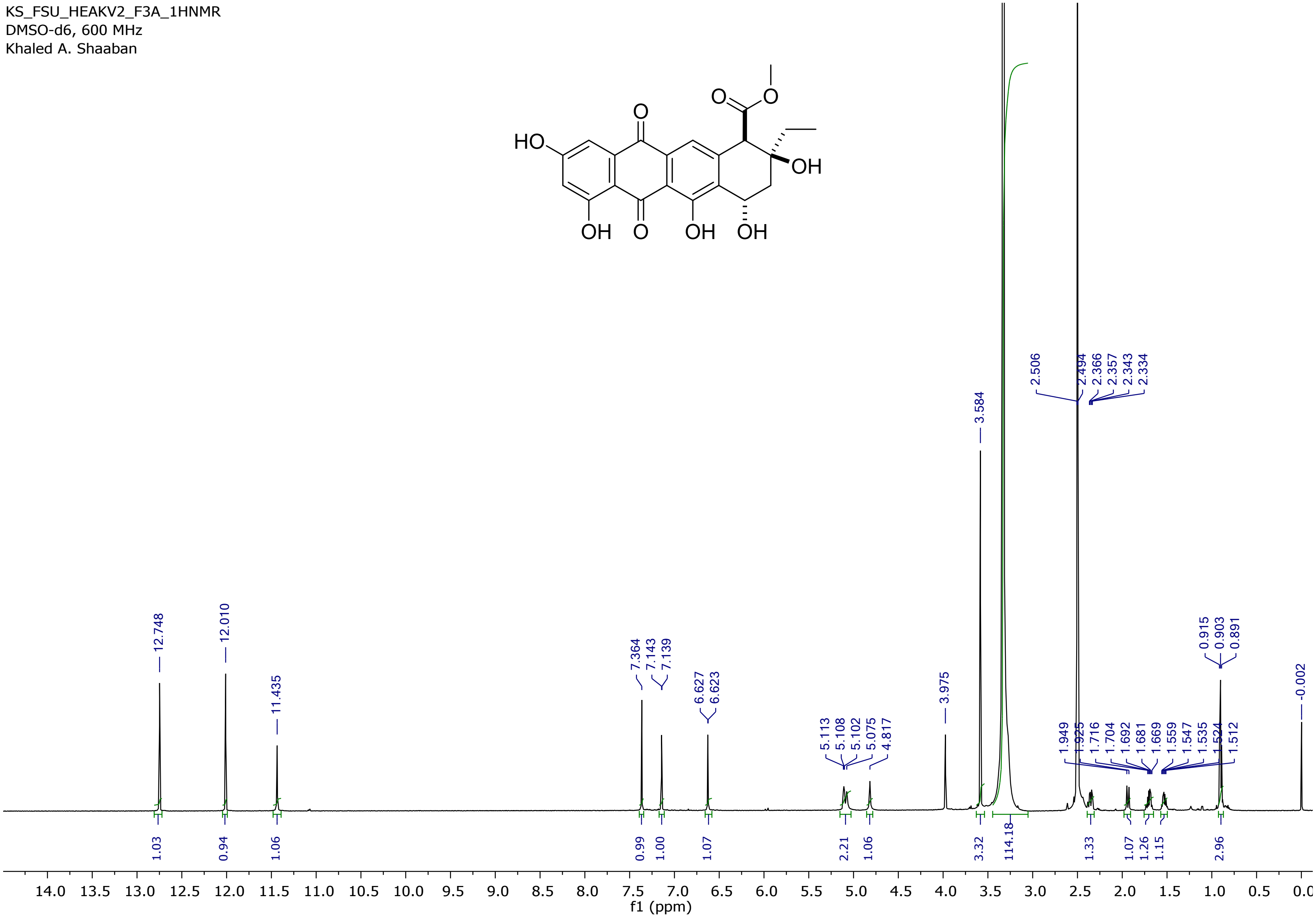

Figure S18. ^{1}H NMR spectrum (DMSO-d_6, 600 MHz) of 2-hydroxy-9-*epi*-aklavinone (**12**).

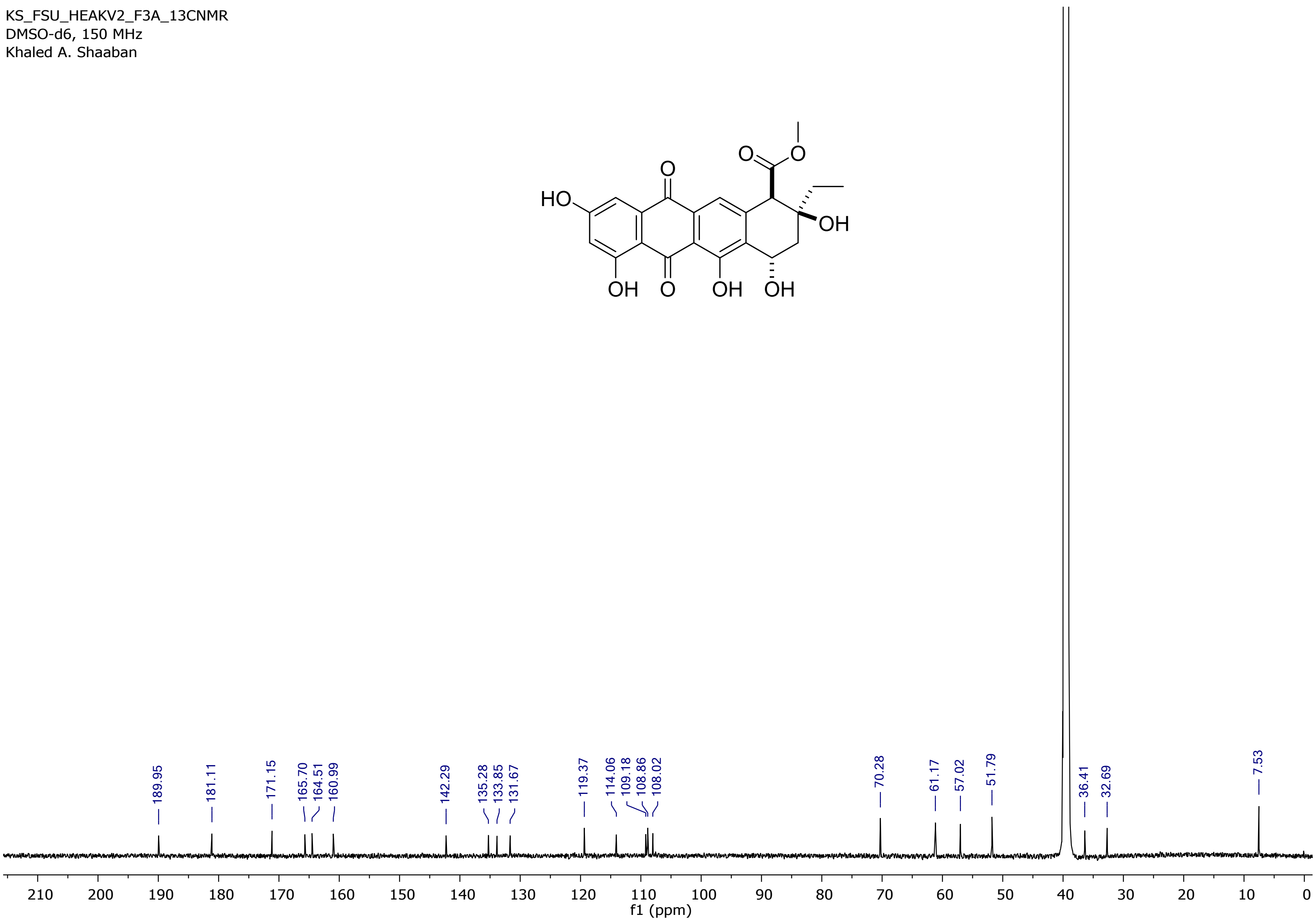

Figure S19. ^{13}C NMR spectrum (DMSO-d_6, 150 MHz) of 2-hydroxy-9-*epi*-aklavinone (**12**).

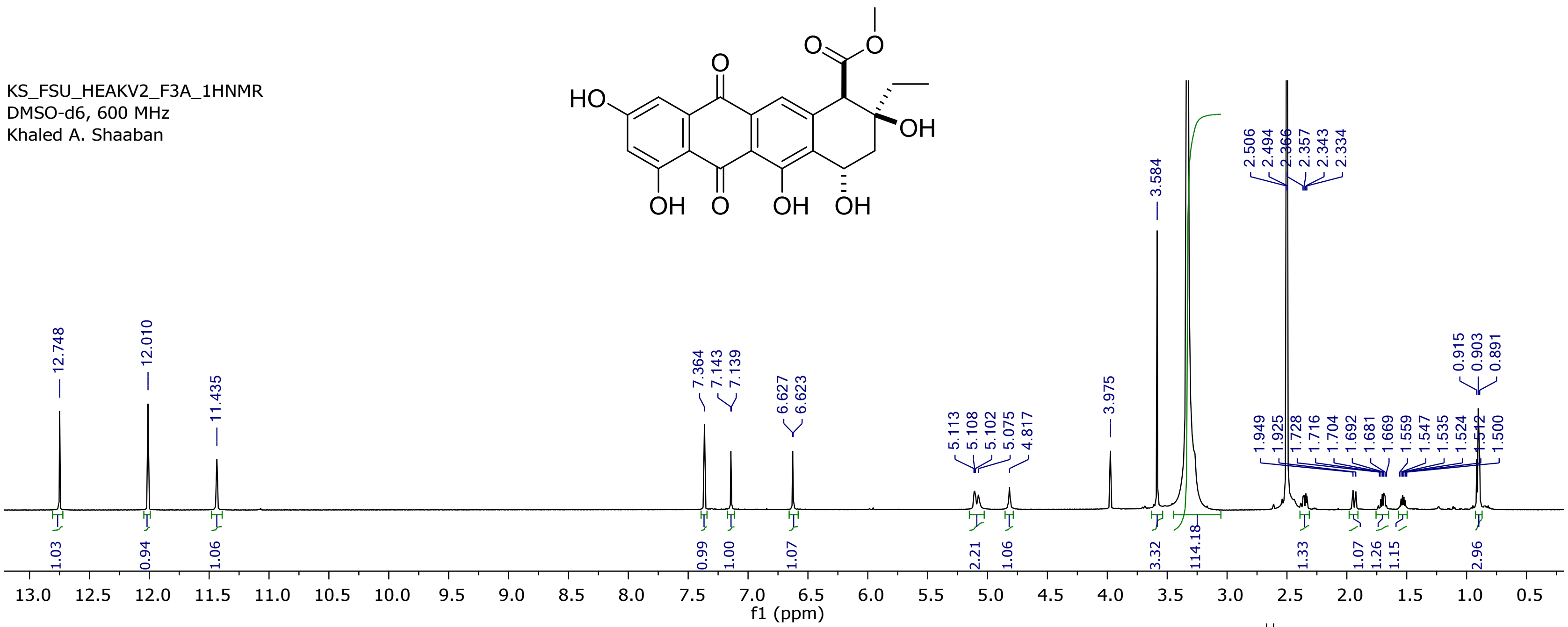

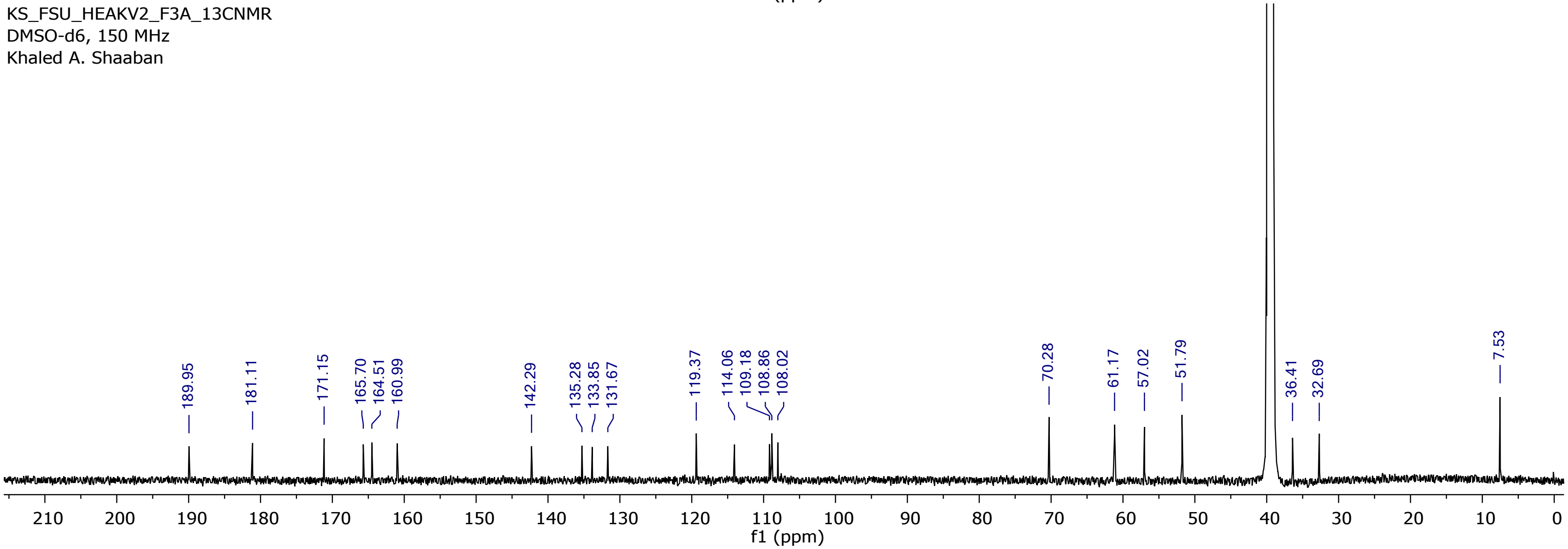

Figure S20. ^1H (DMSO-d_6, 600 MHz) and ^{13}C (DMSO-d_6, 150 MHz) NMR spectra of 2-hydroxy-9-*epi*-aklavinone (**12**).

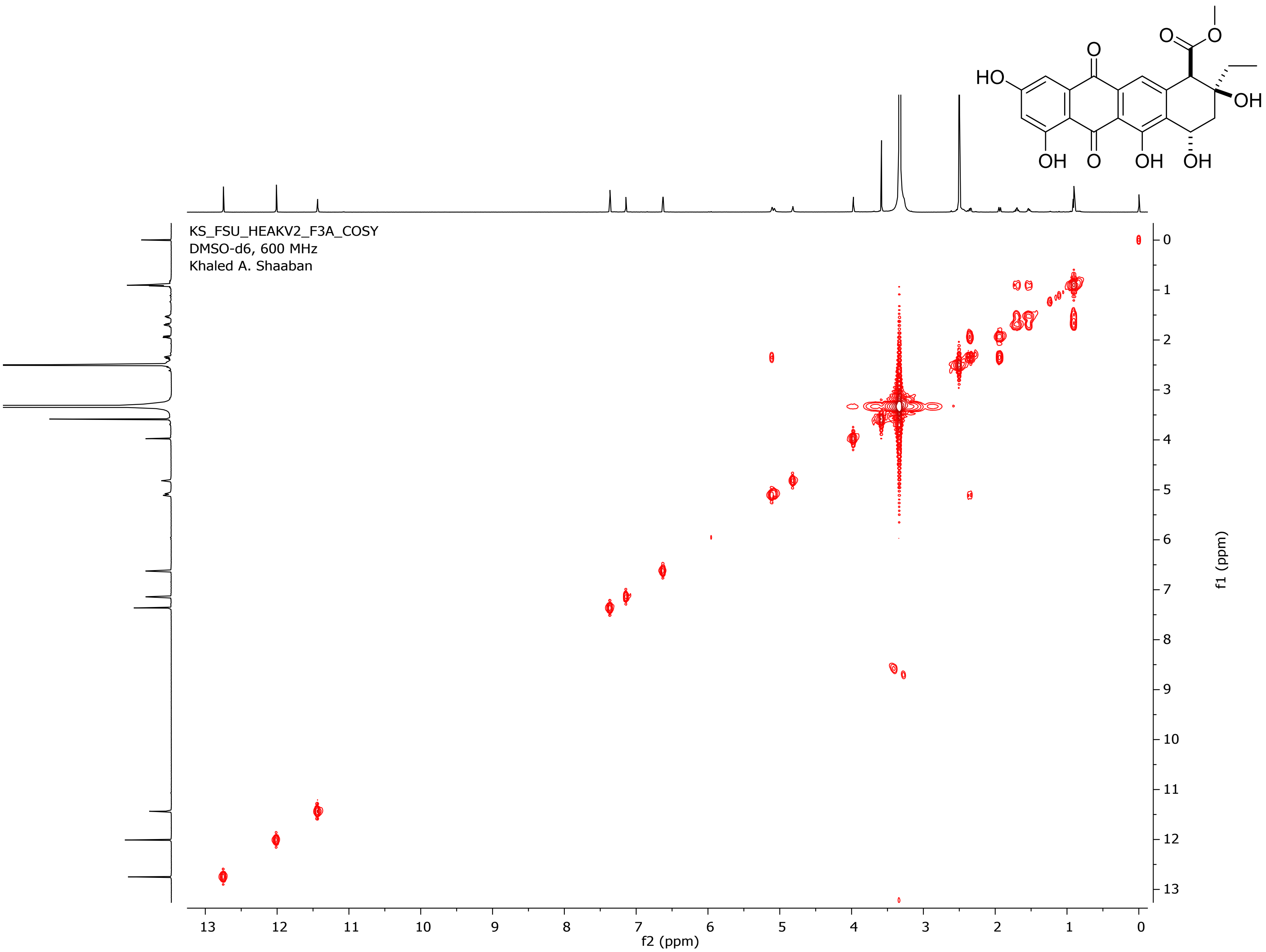

Figure S21. $^1H,^1H$-COSY spectrum (DMSO-d_6, 600 MHz) of 2-hydroxy-9-*epi*-aklavinone (**12**).

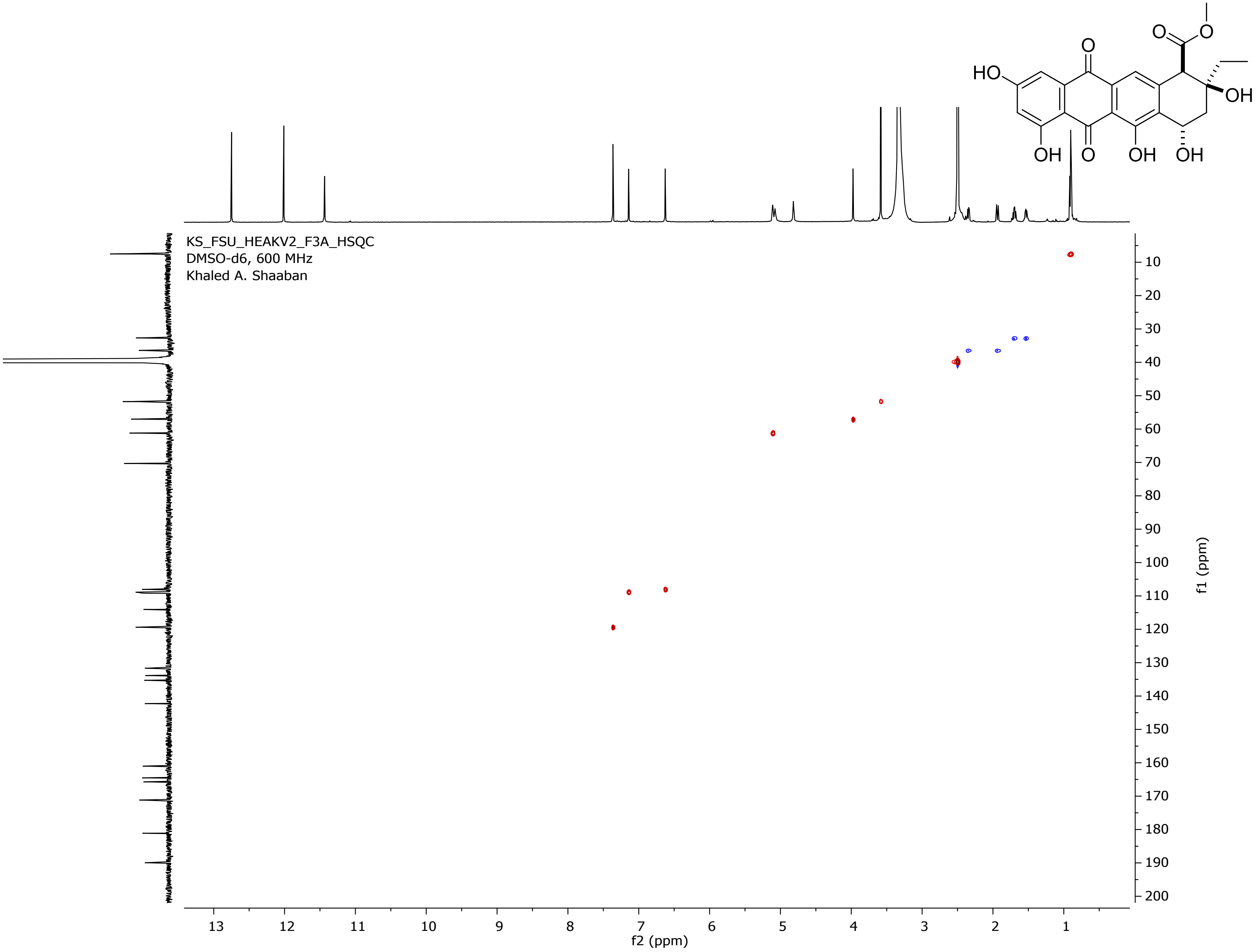

Figure S22. HSQC spectrum (DMSO-d_6, 600 MHz) of 2-hydroxy-9-*epi*-aklavinone (**12**).

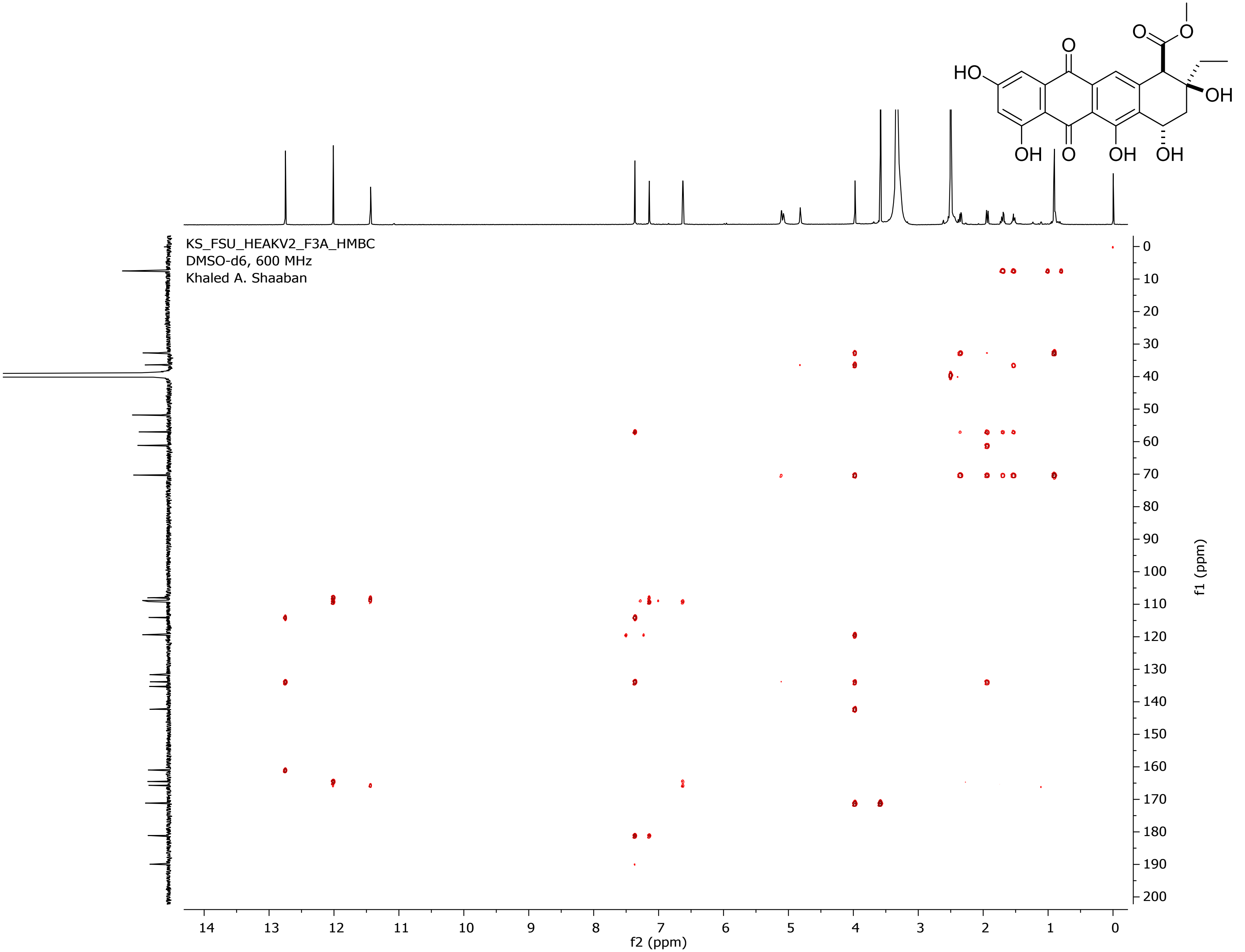

Figure S23. HMBC spectrum (DMSO-d_6, 600 MHz) of 2-hydroxy-9-*epi*-aklavinone (**12**).

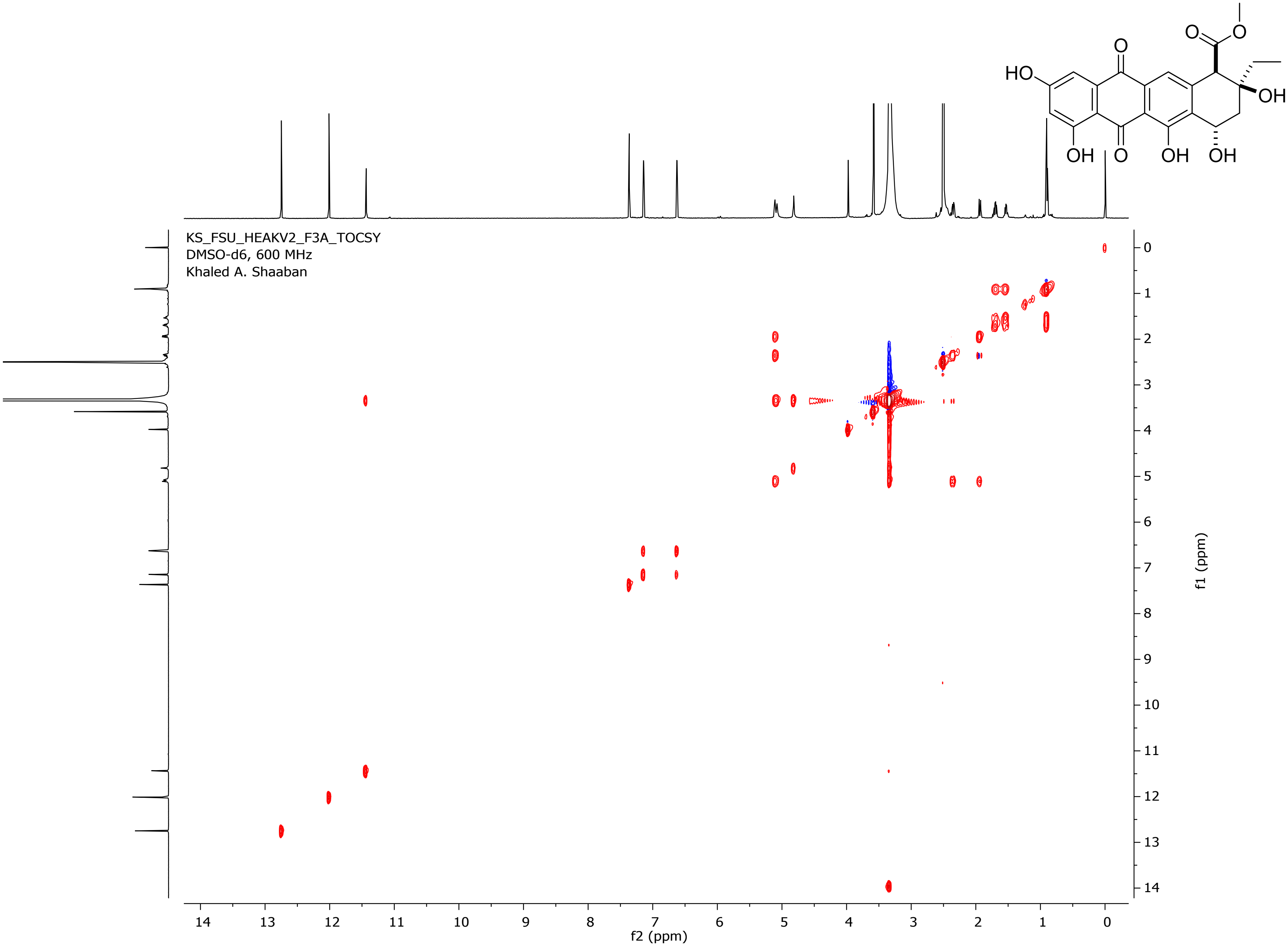

Figure S24. TOCSY spectrum (DMSO-d_6, 600 MHz) of 2-hydroxy-9-*epi*-aklavinone (**12**).

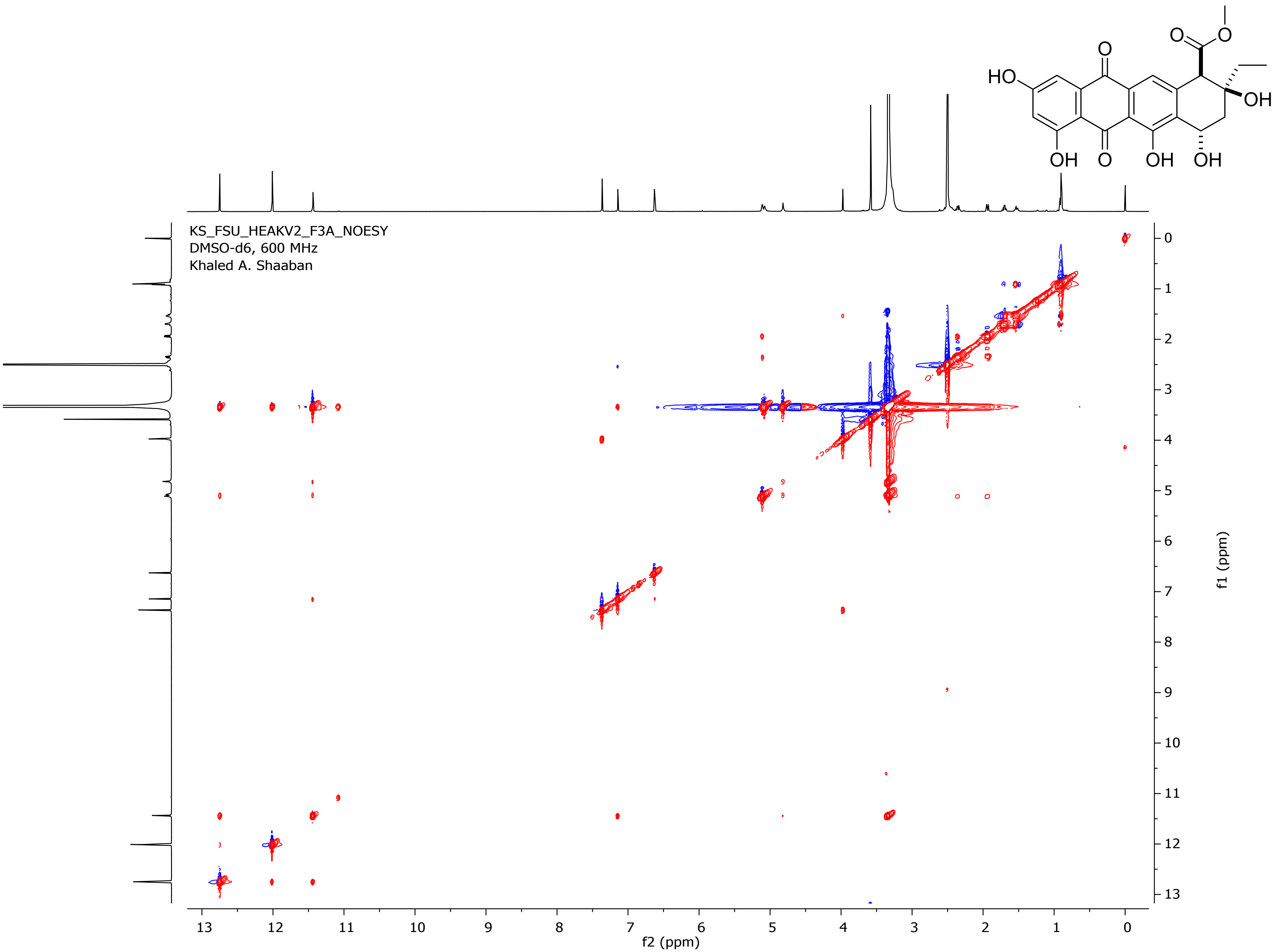

Figure S25. NOESY spectrum (DMSO-d_6, 600 MHz) of 2-hydroxy-9-*epi*-aklavinone (**12**).

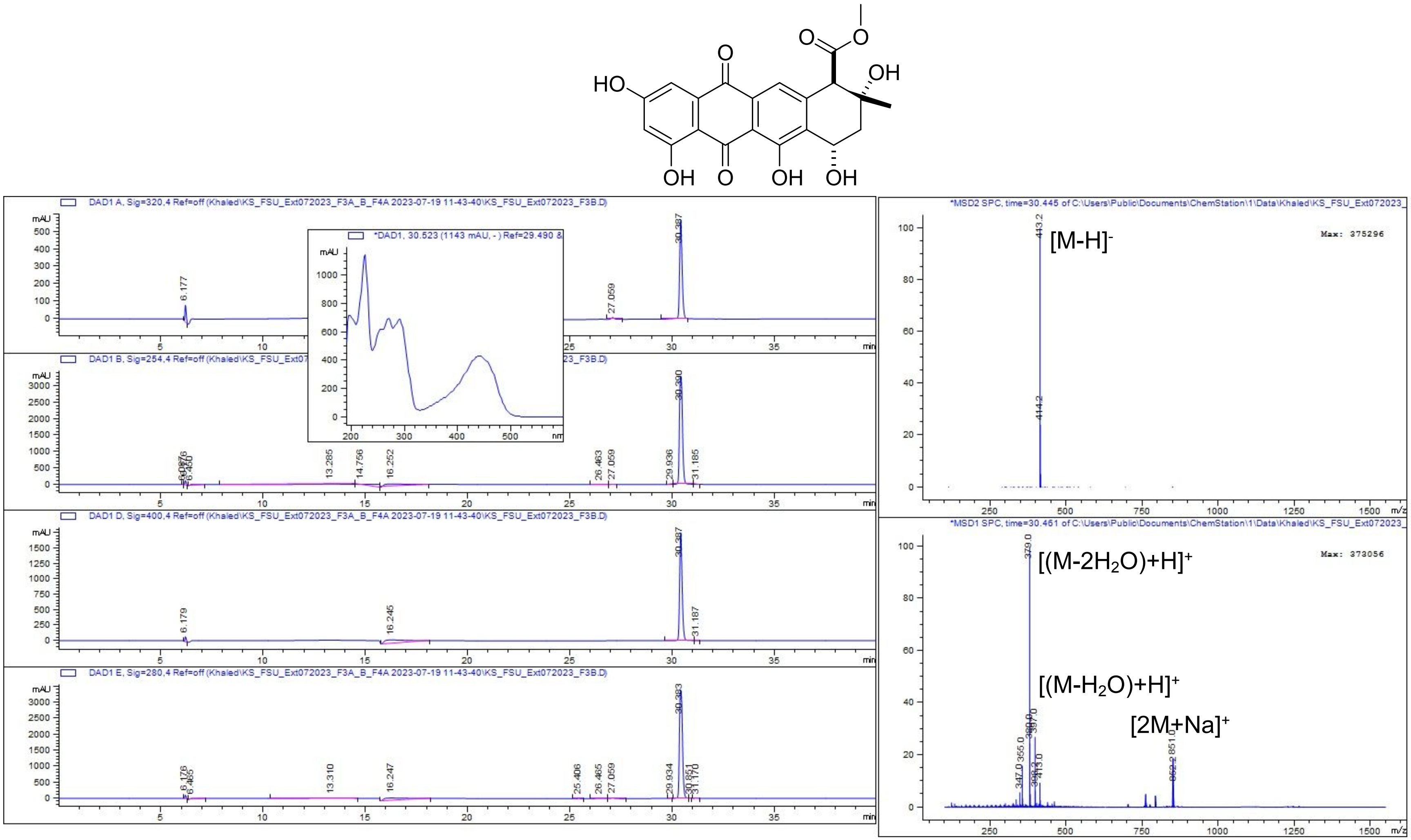

Figure S26: HPLC-MS analysis of 2-hydroxy-auramycinone (**13**). HPLC-conditions: solvent A: H_2O/0.1% FA; solvent B: CH_3CN; flow rate: 0.5 mL min^{-1}; 0-30 min, 5-100% B; 30-35 min, 100% B; 35-36 min, 100-5% B; 36-40 min, 5% B; Phenomenex NX-C18 column (250 × 4.6 mm, 5 μm); 254 nm, 280 nm, 320 nm, 400 nm. UV-vis inset of full wavelength scan (190-600 nm).

**Sample Name**	CN572-F3B	**Position**	P1-C2	**Instrument Name**	Instrument 1
**User Name**		**Inj Vol**	10	**InjPosition**	
**Sample Type**	Sample	**IRM Calibration Status**	Success	**Data Filename**	CN572-F3B.d
**ACQ Method**	Zheng_AQC ACC ND short_Pos.m	**Comment**		**Acquired Time**	10/10/2023 4:28:32 PM (UTC-04:00)

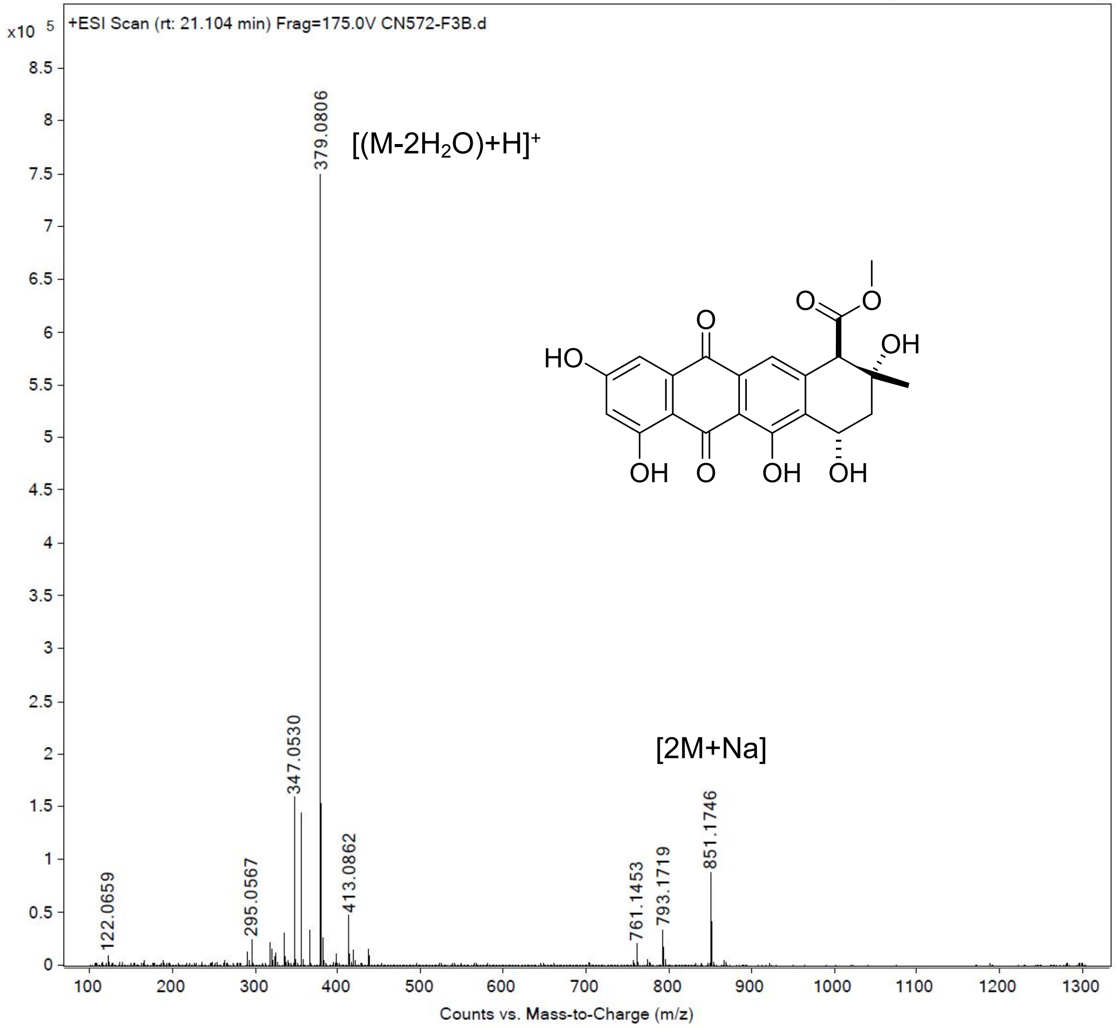

Figure S27. (+)-HRESI-MS spectrum of 2-Hydroxy-auramycinone (**13**).

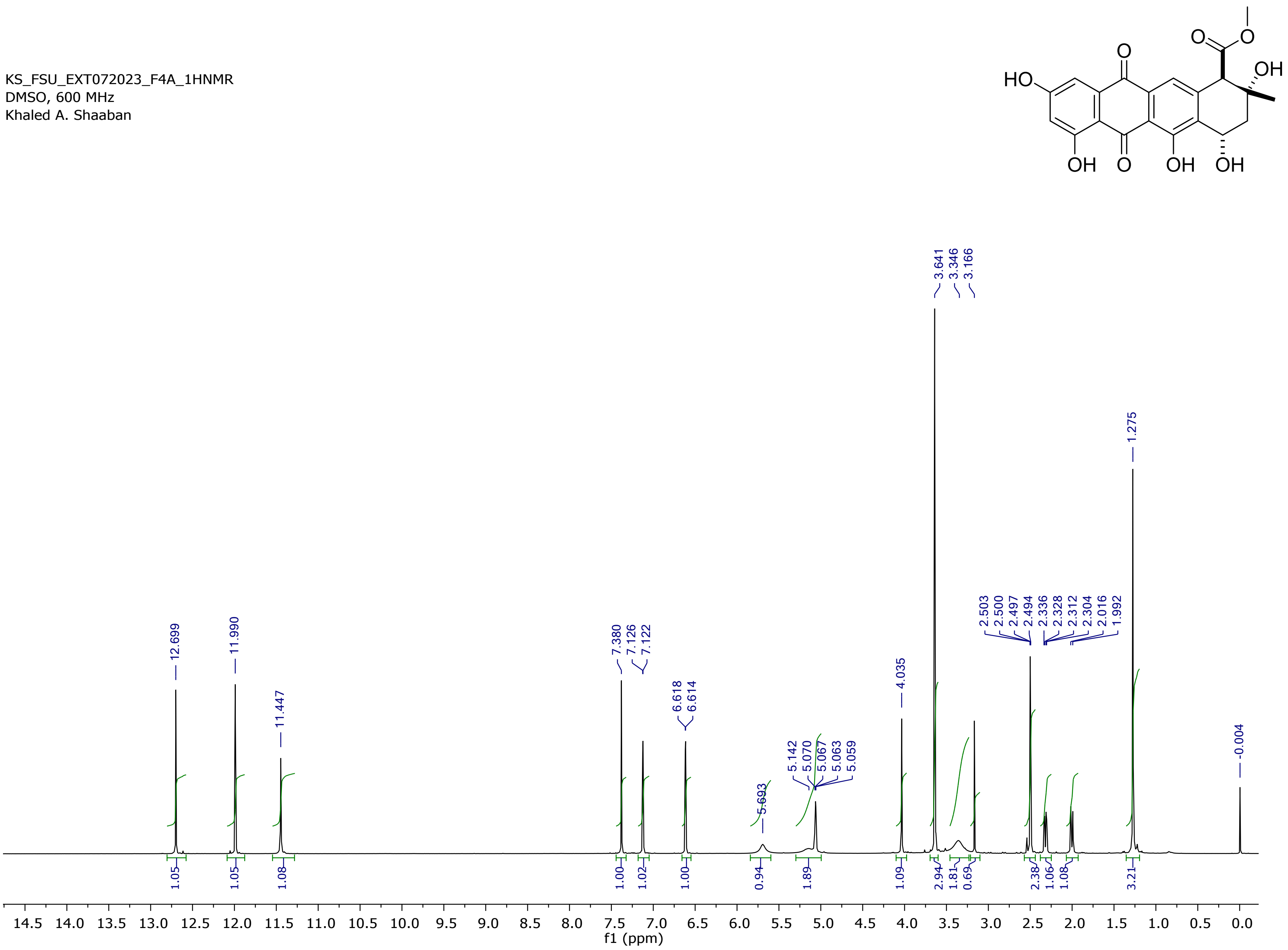

Figure S28. ^{1}H NMR spectrum (DMSO-d_6, 600 MHz) of 2-hydroxy-auramycinone (**13**).

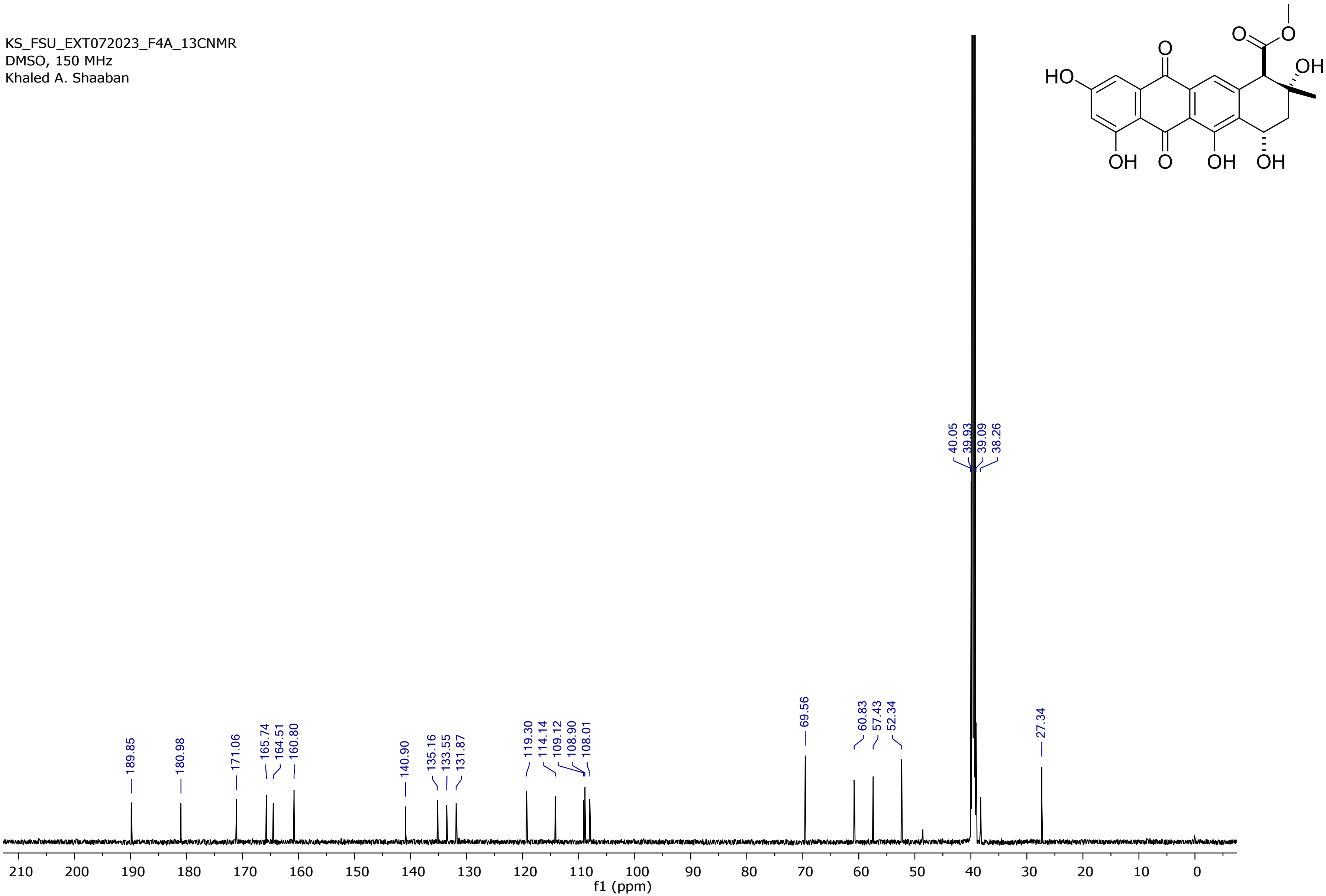

Figure S29. ^{13}C NMR spectrum (DMSO-d_6, 150 MHz) of 2-hydroxy-auramycinone (**13**).

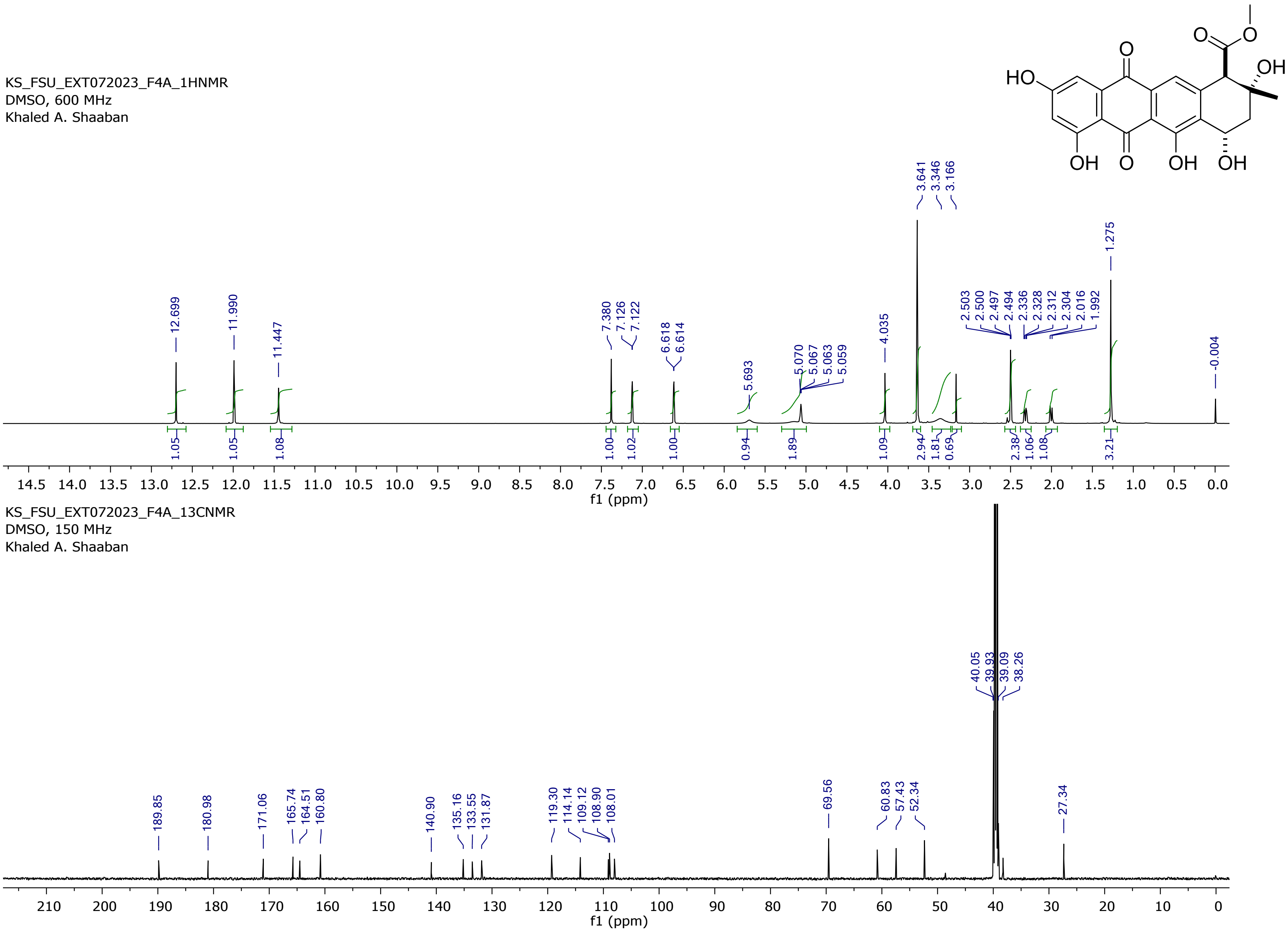

Figure S30. ^{1}H (DMSO-d_6, 600 MHz) and ^{13}C (DMSO-d_6, 150 MHz) NMR spectra of 2-hydroxy-auramycinone (**13**).

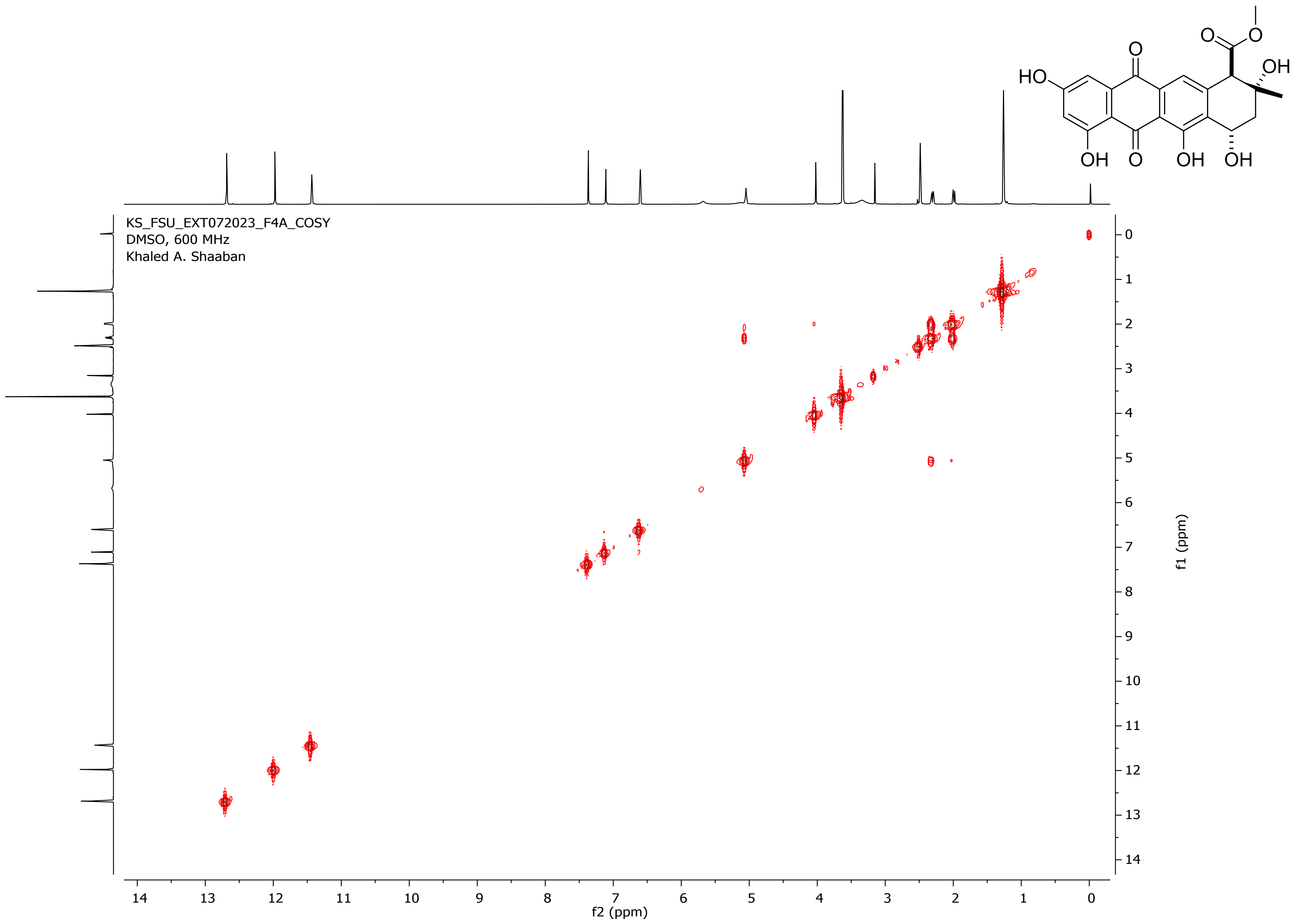

Figure S31. ^{1}H,^{1}H-COSY spectrum (DMSO-d_6, 600 MHz) of 2-hydroxy-auramycinone (**13**).

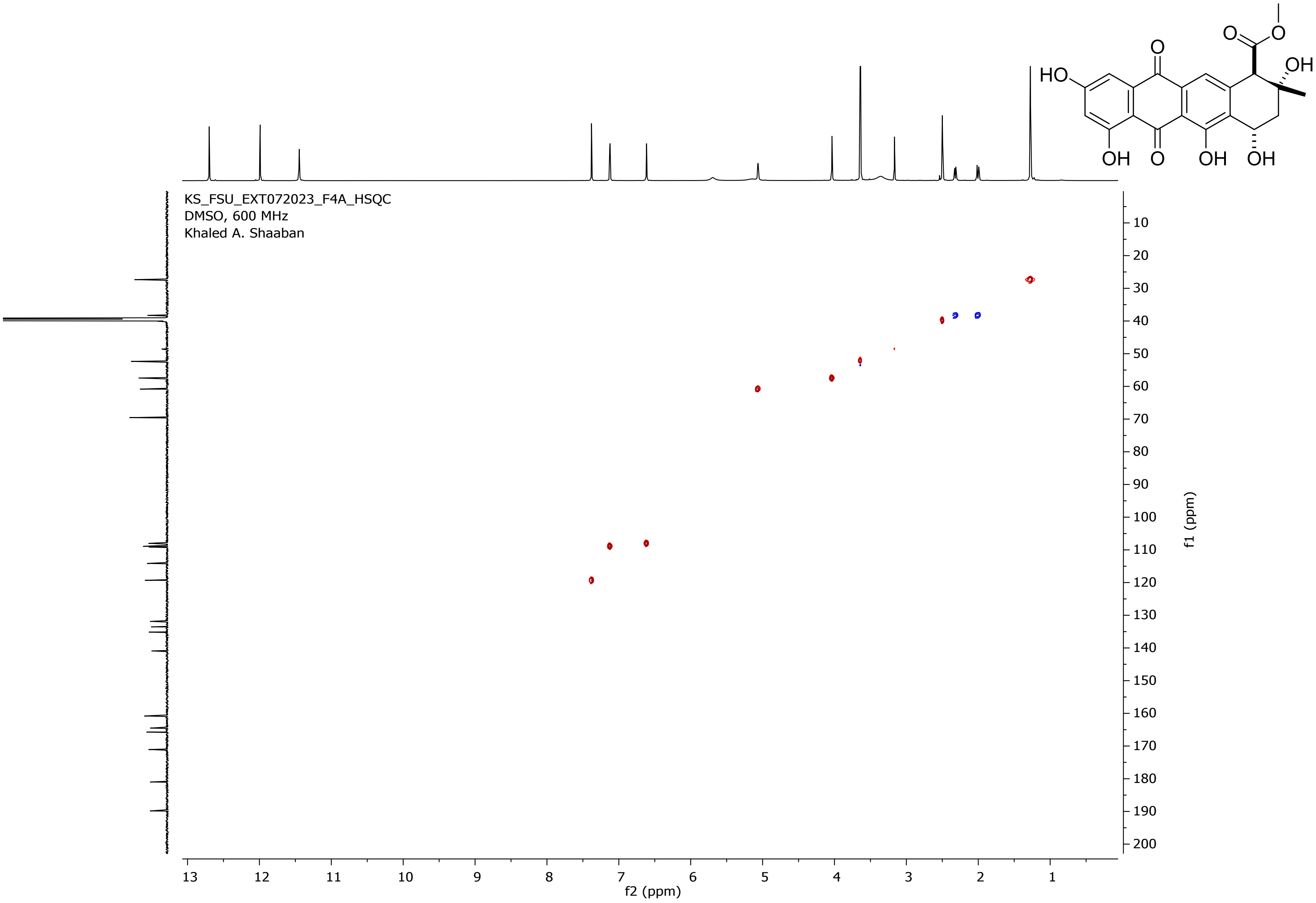

Figure S32. HSQC spectrum (DMSO-d_6, 600 MHz) of 2-hydroxy-auramycinone (**13**).

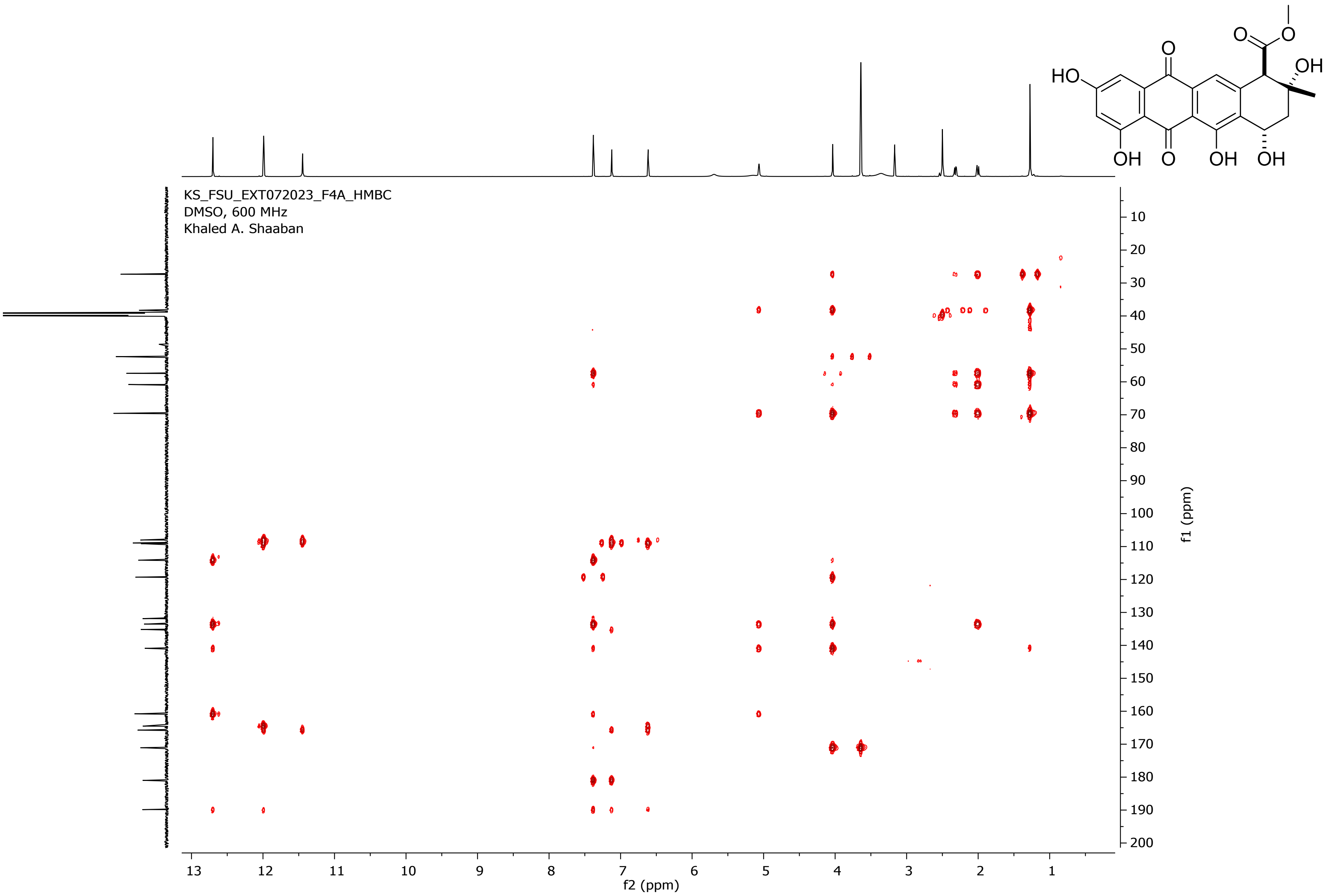

Figure S33. HMBC spectrum (DMSO-d_6, 600 MHz) of 2-hydroxy-auramycinone (**13**).

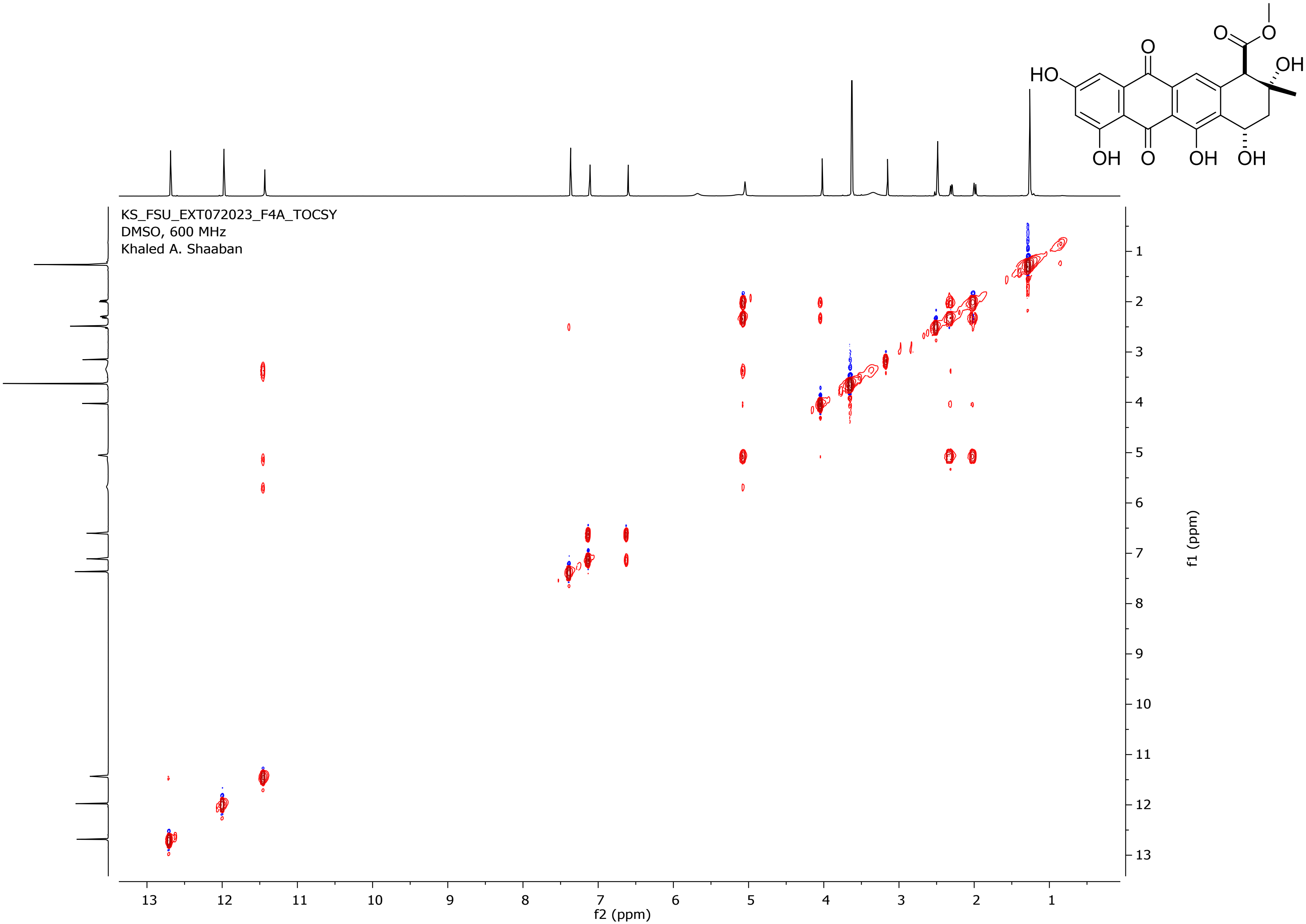

Figure S34. TOCSY spectrum (DMSO-d_6, 600 MHz) of 2-hydroxy-auramycinone (**13**).

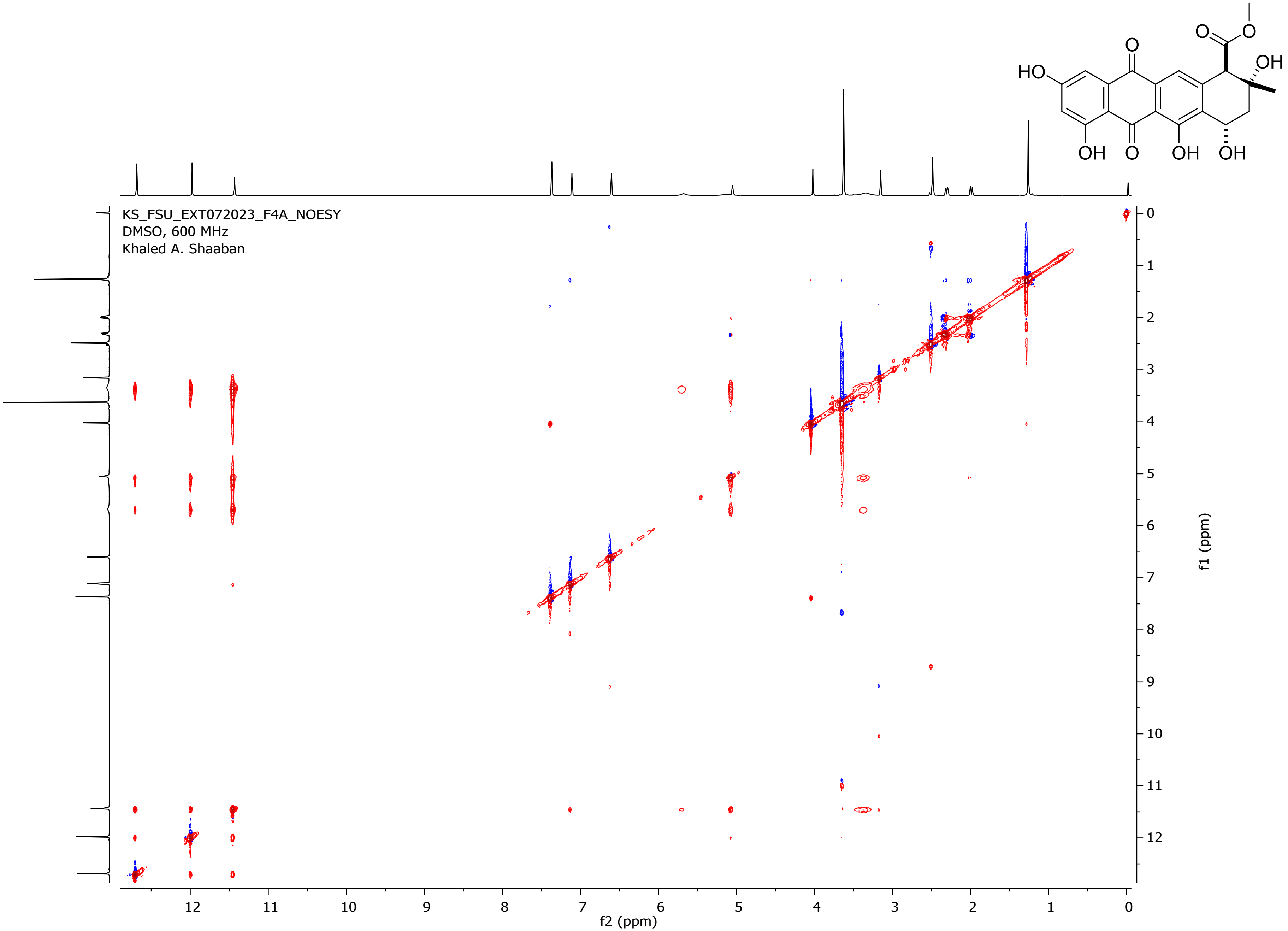

Figure S35. NOESY spectrum (DMSO-d_6, 600 MHz) of 2-hydroxy-auramycinone (**13**).

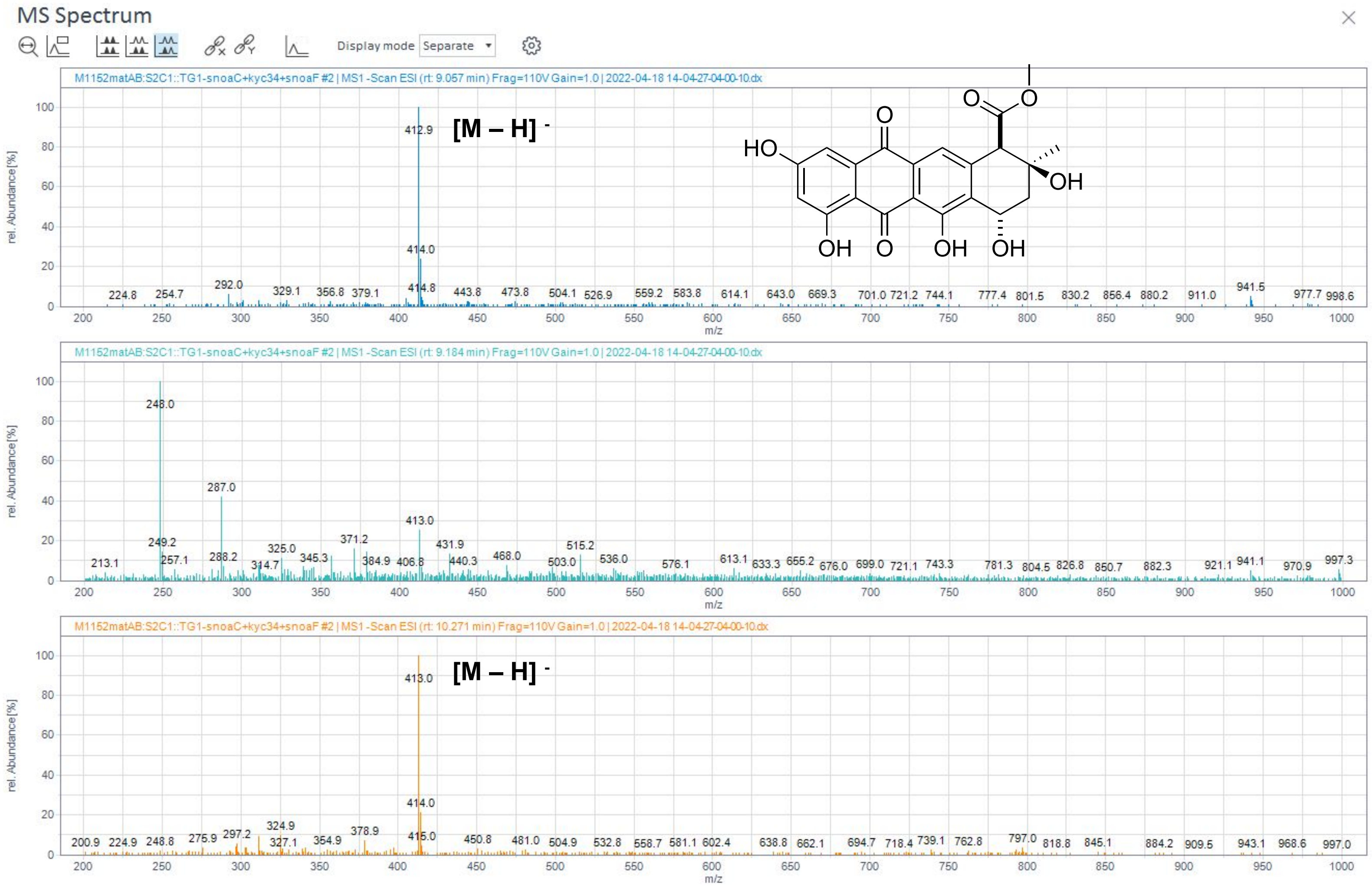

Figure S36. Mass spectrum of 2-hydroxy-nogalamycinone (**14**).

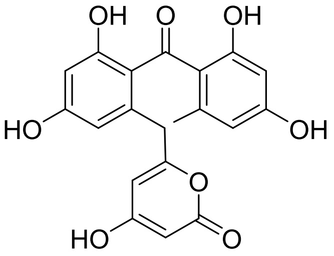

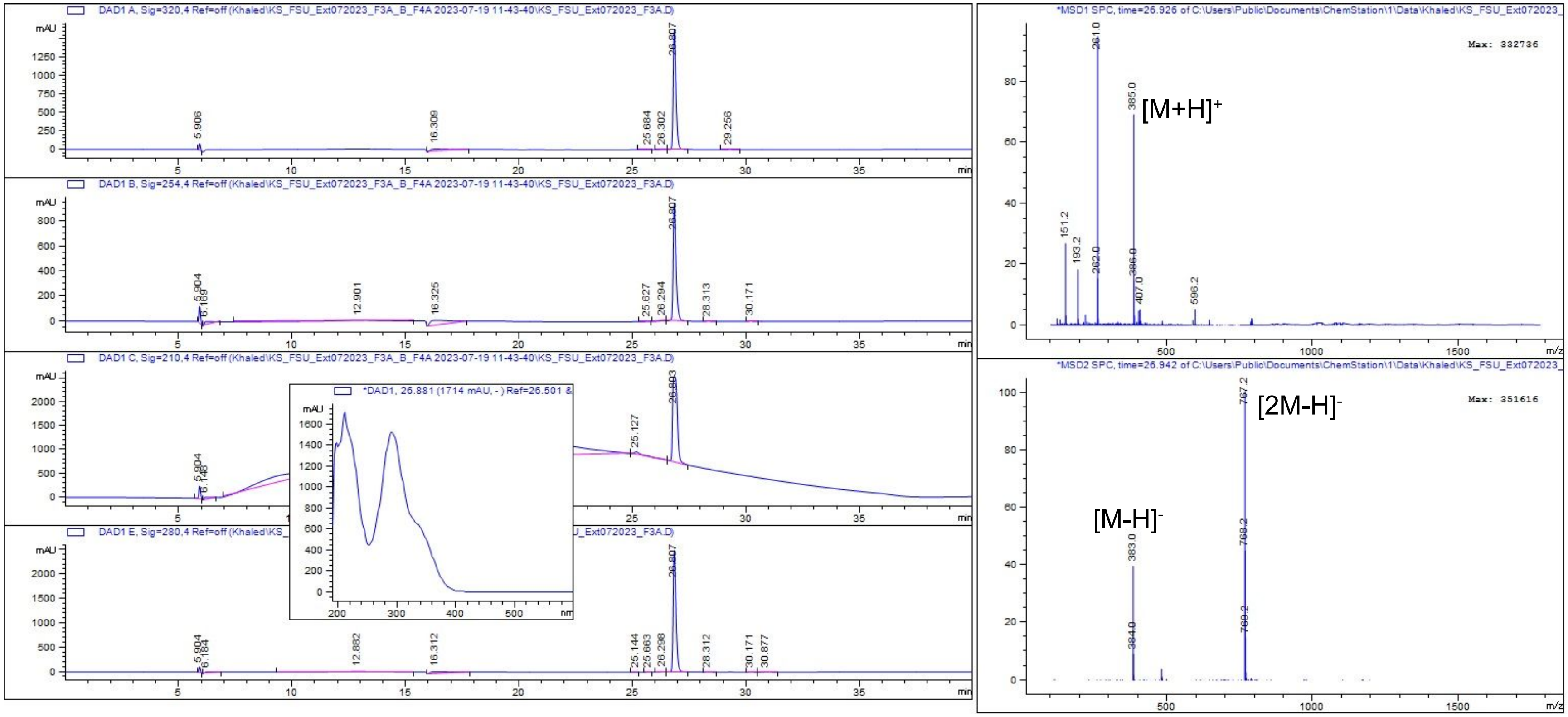

Figure S37: HPLC-MS analysis of SEK15. (**4**). HPLC-conditions: solvent A: H_2O/0.1% FA; solvent B: CH_3CN; flow rate: 0.5 mL min^{-1}; 0-30 min, 5-100% B; 30-35 min, 100% B; 35-36 min, 100-5% B; 36-40 min, 5% B; Phenomenex NX-C18 column (250 × 4.6 mm, 5 μm); 210 nm, 254 nm, 280 nm, 320 nm. UV-vis inset of full wavelength scan (190-600 nm).

**Sample Name**	CN573-F3A	**Position**	P1-C4	**Instrument Name**	Instrument 1
**User Name**		**Inj Vol**	10	**InjPosition**	
**Sample Type**	Sample	**IRM Calibration Status**	Success	**Data Filename**	CN573-F3A.d
**ACQ Method**	Zheng_AQC ACC ND short_Pos.m	**Comment**		**Acquired Time**	10/10/2023 5:26:42 PM (UTC-04:00

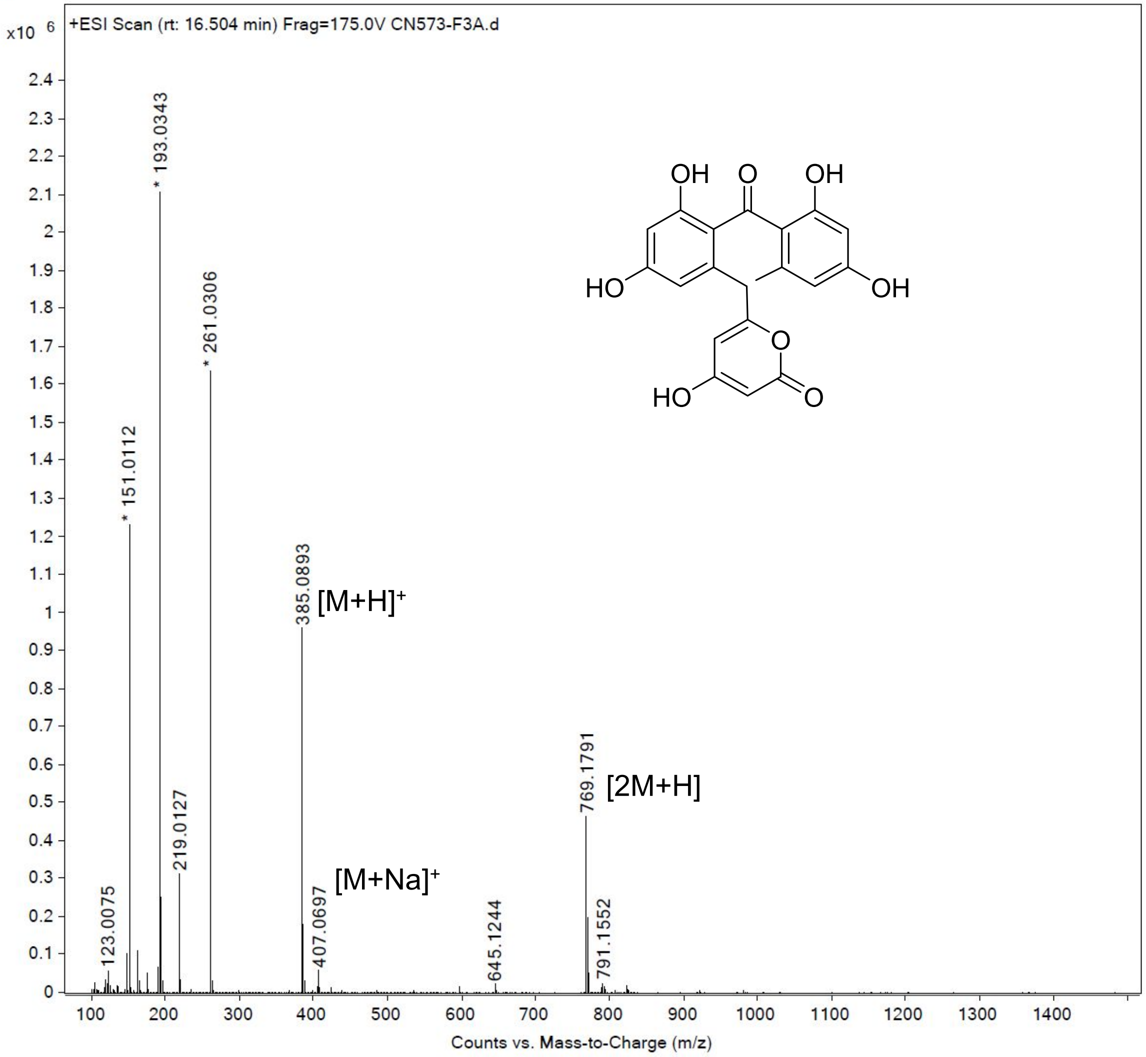

Figure S38. (+)-HRESI-MS spectrum of SEK15.

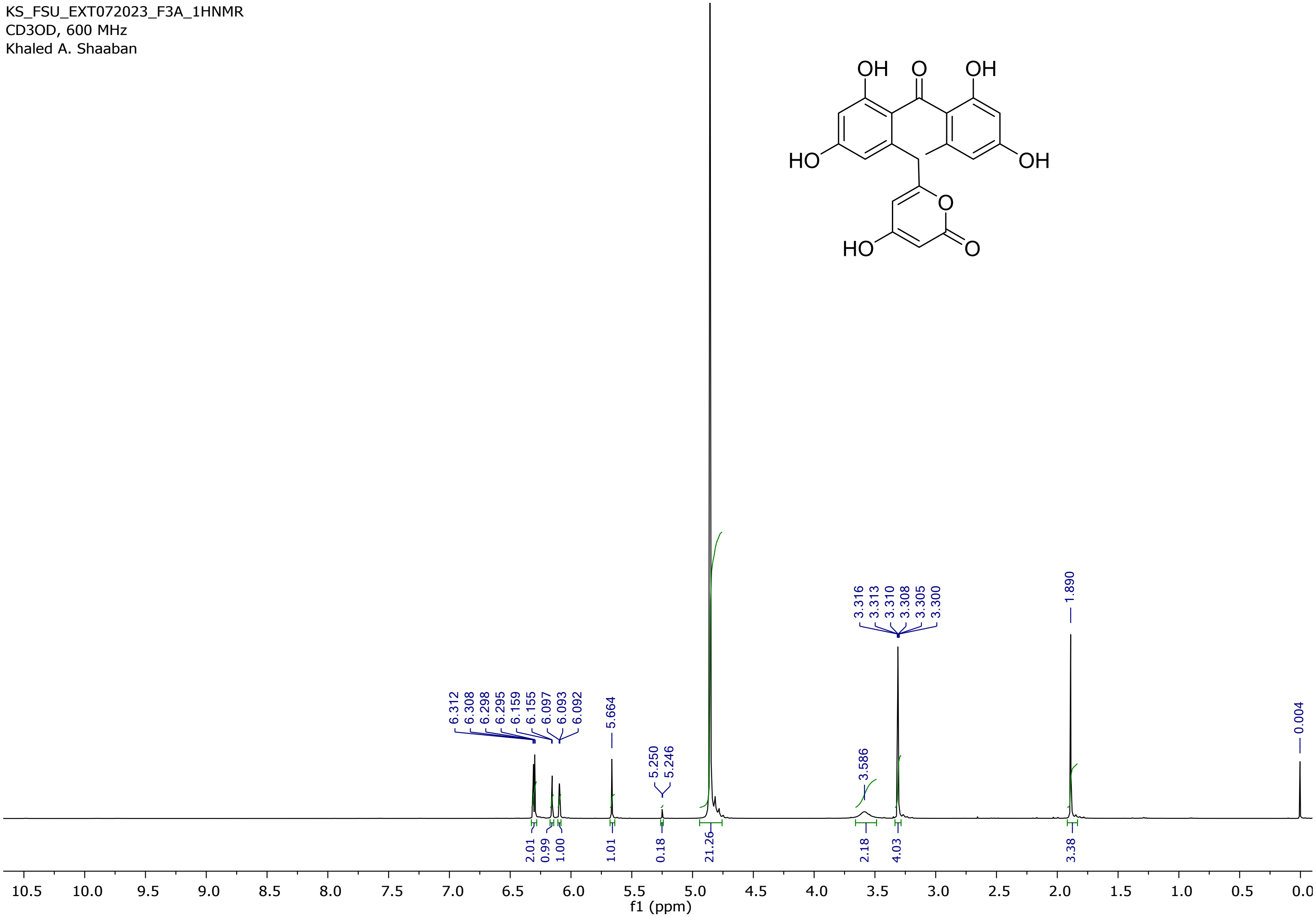

Figure S39. ^{1}H NMR spectrum (CD_3OD, 600 MHz) of SEK15.

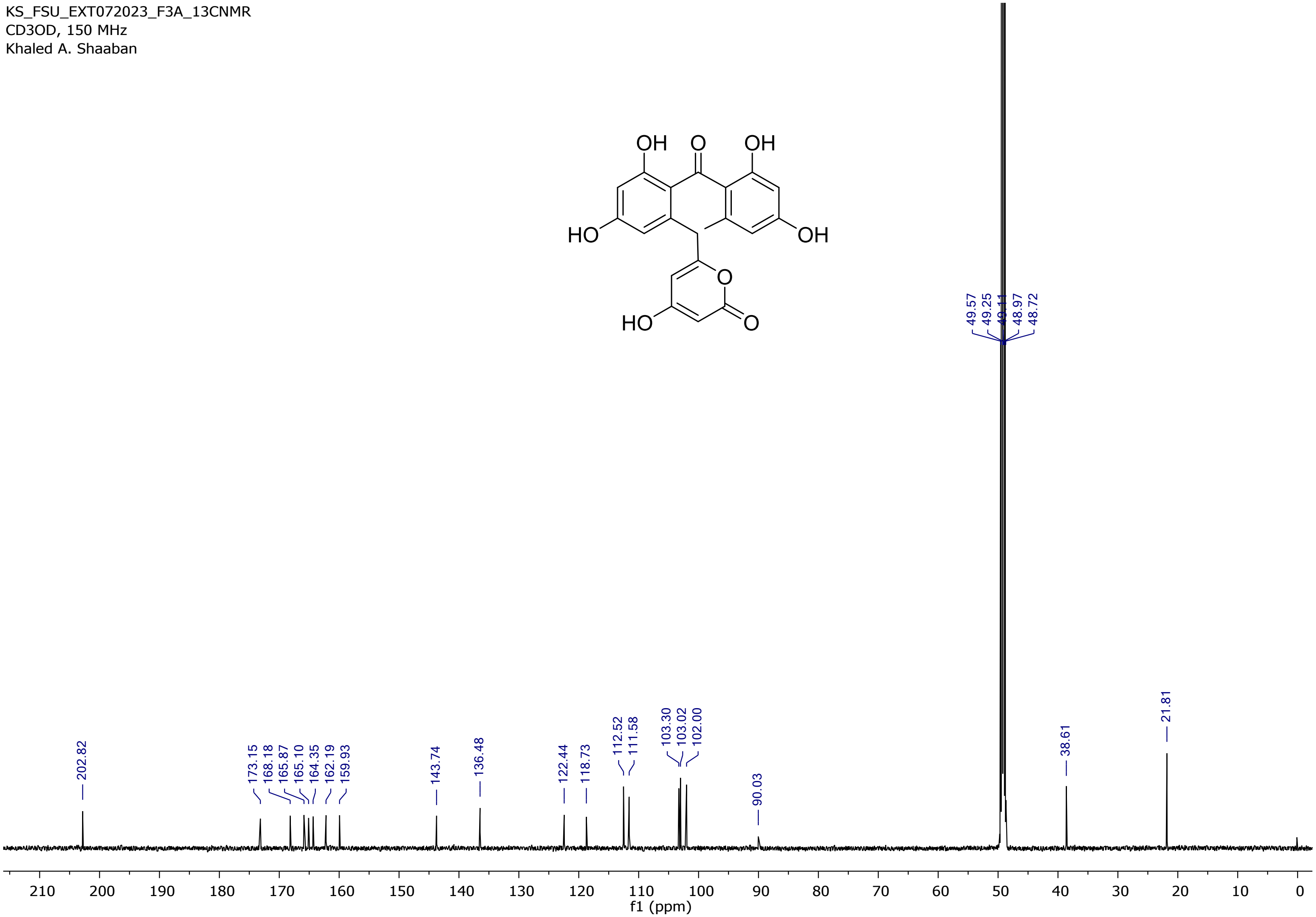

Figure S40. ^{13}C NMR spectrum (CD_3OD, 150 MHz) of SEK15.

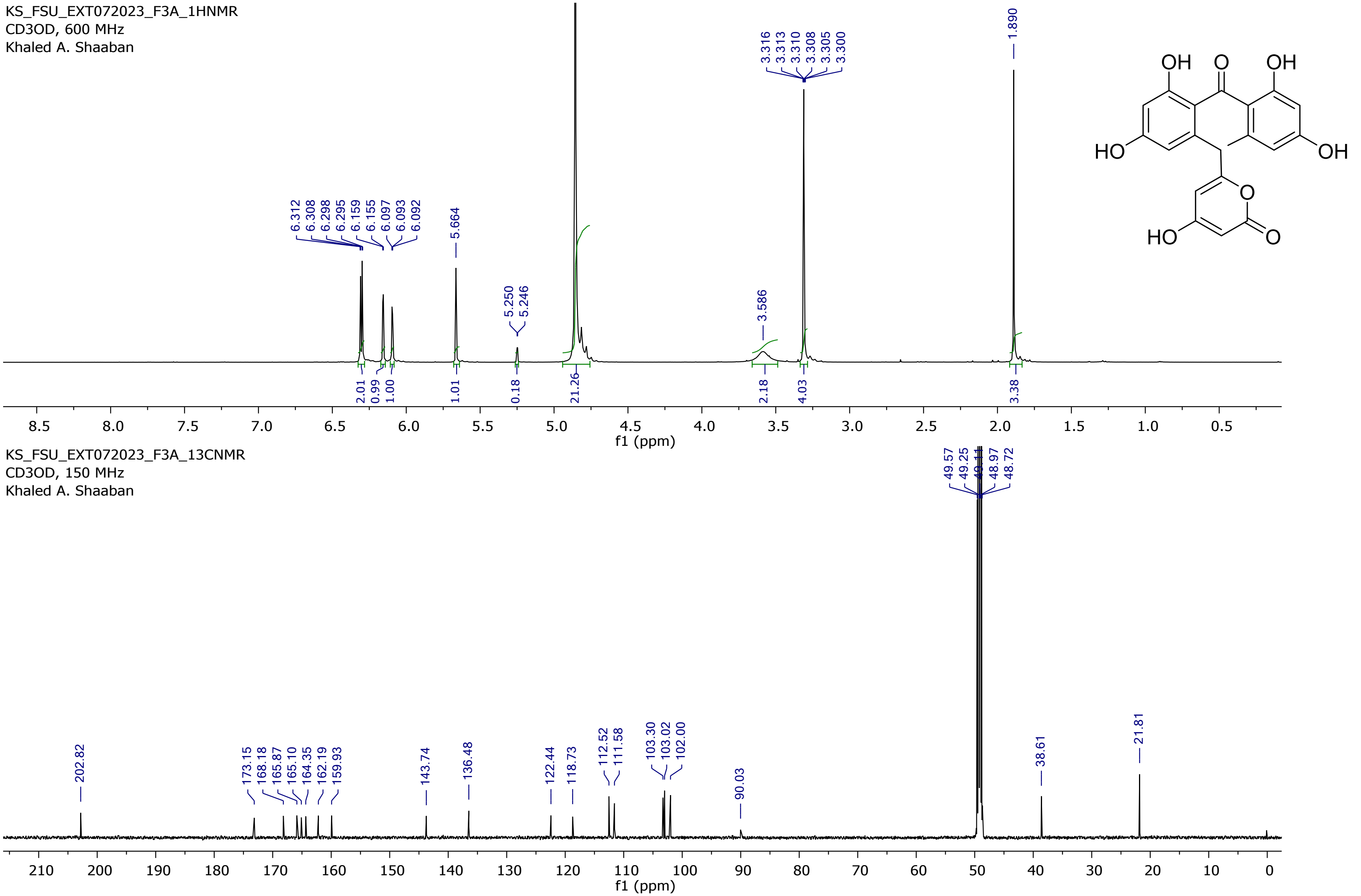

Figure S41. ^{1}H (CD_3OD, 600 MHz) and ^{13}C (CD_3OD, 150 MHz) NMR spectra of SEK15.

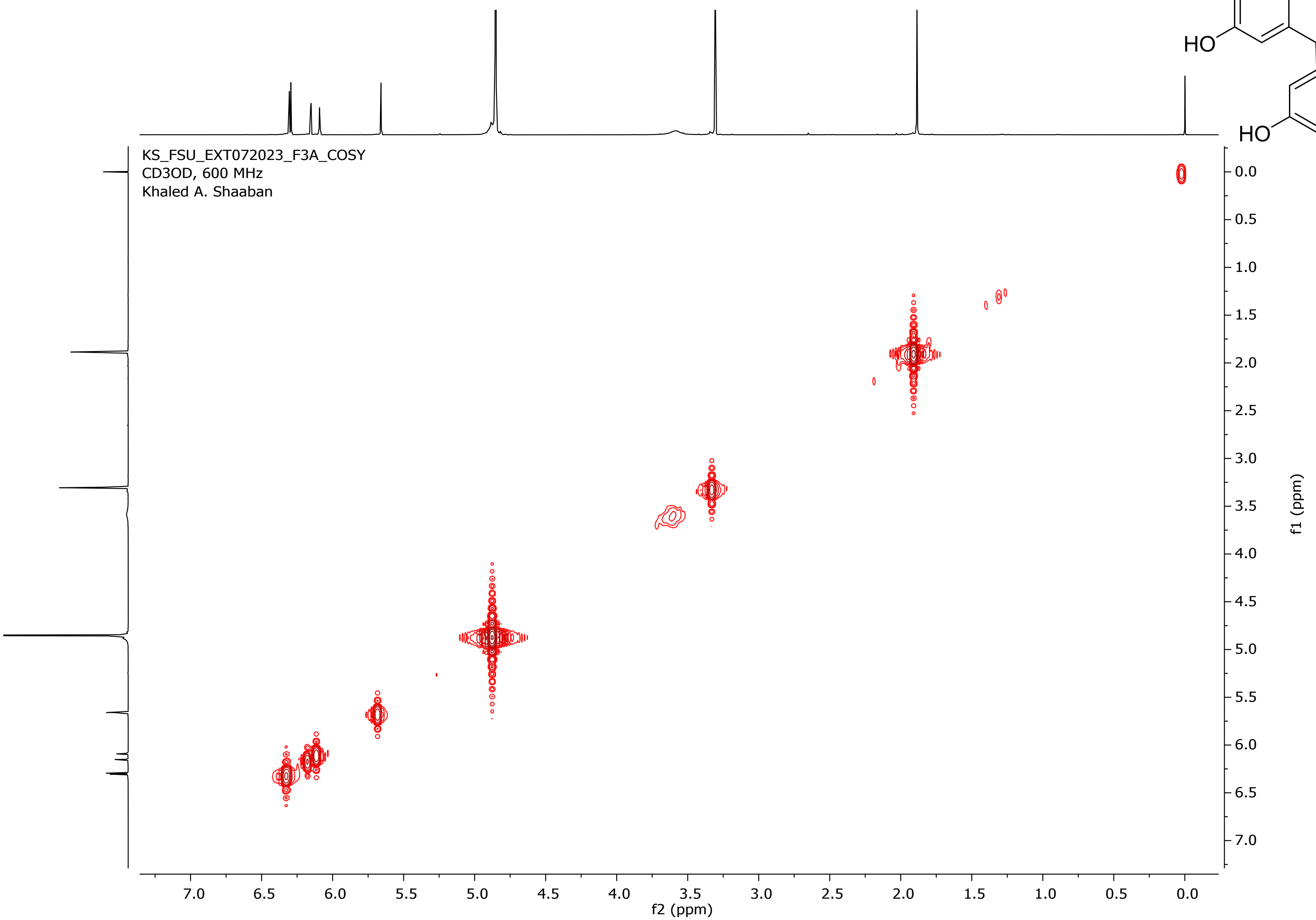

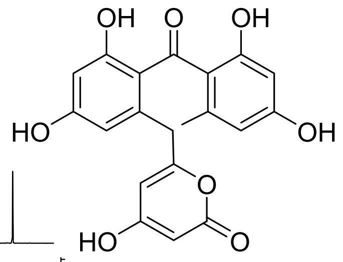

Figure S42. $^{1}H,^{1}H$-COSY spectrum (CD_3OD, 600 MHz) of SEK15.

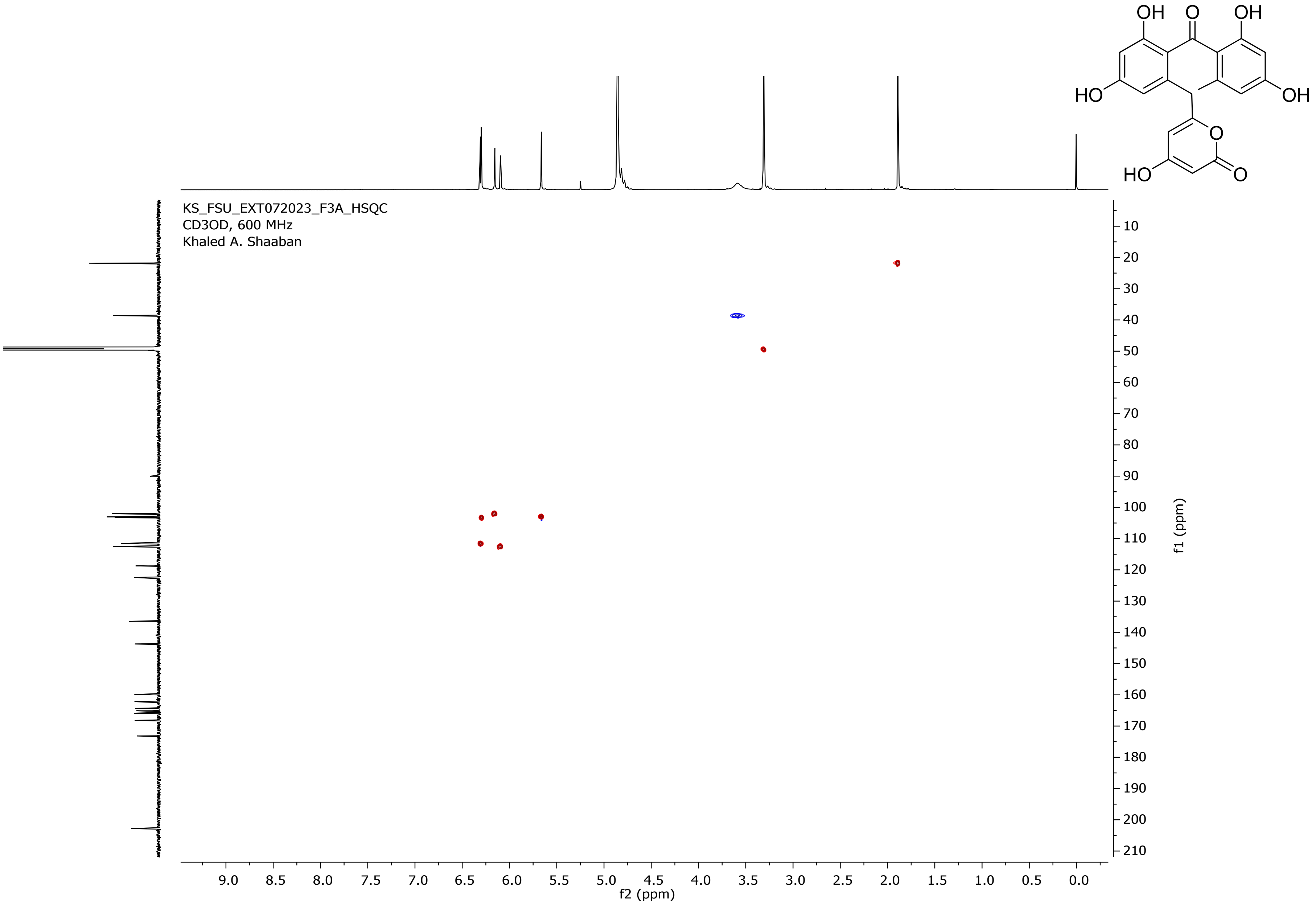

Figure S43. HSQC spectrum (CD_3OD, 600 MHz) of SEK15.

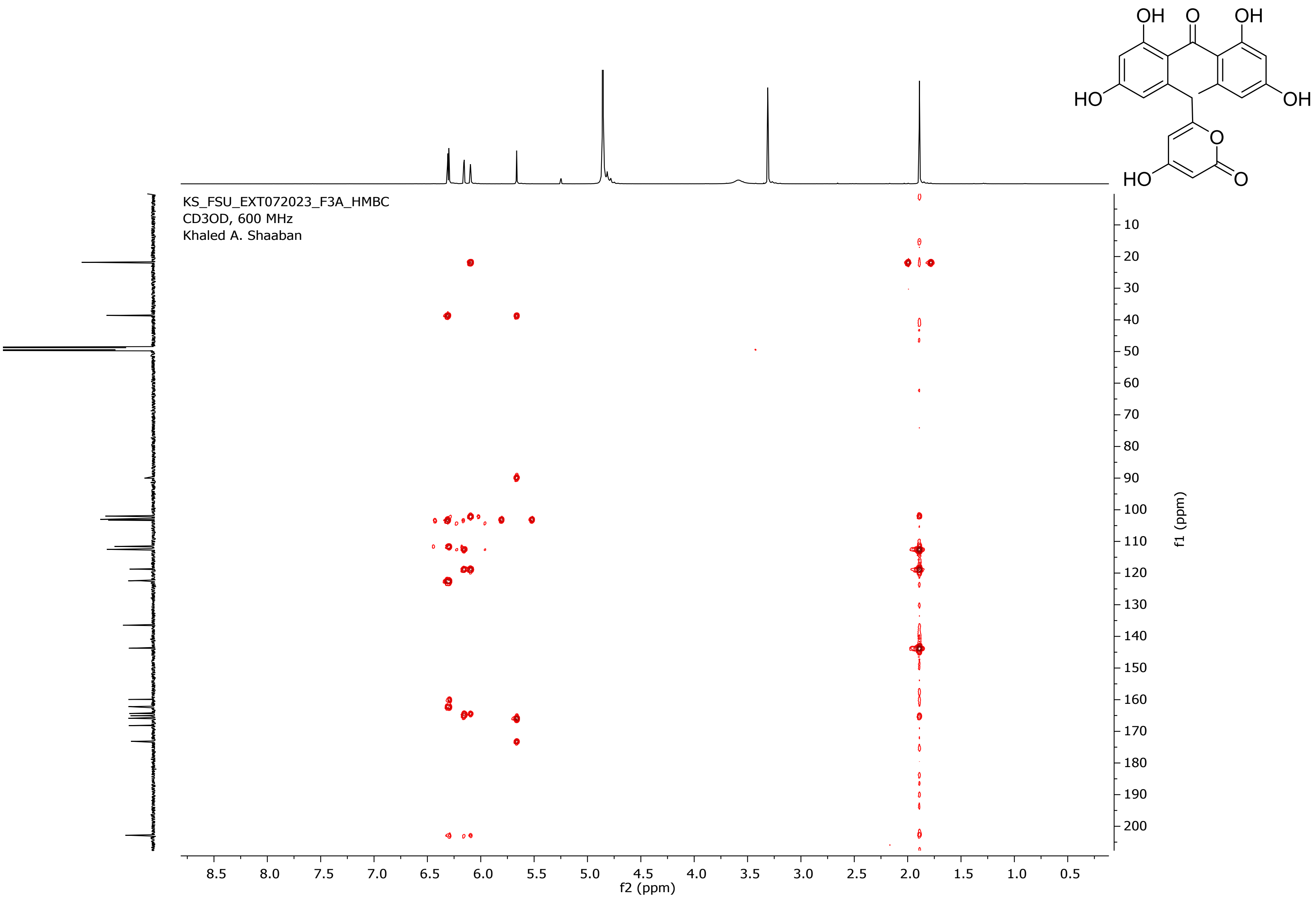

Figure S44. HMBC spectrum (CD_3OD, 600 MHz) of SEK15.

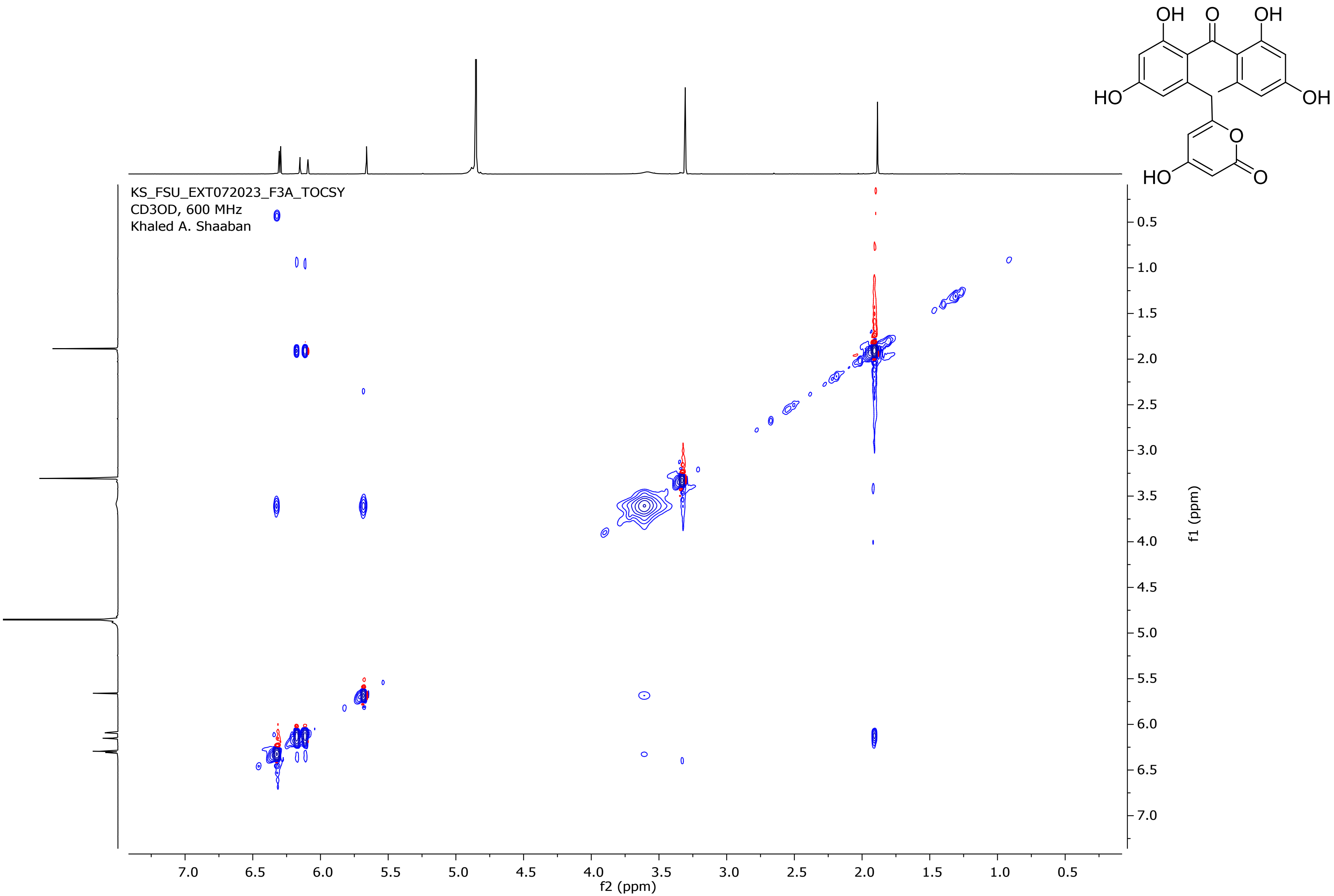

Figure S45. TOCSY spectrum (CD_3OD, 600 MHz) of SEK15.

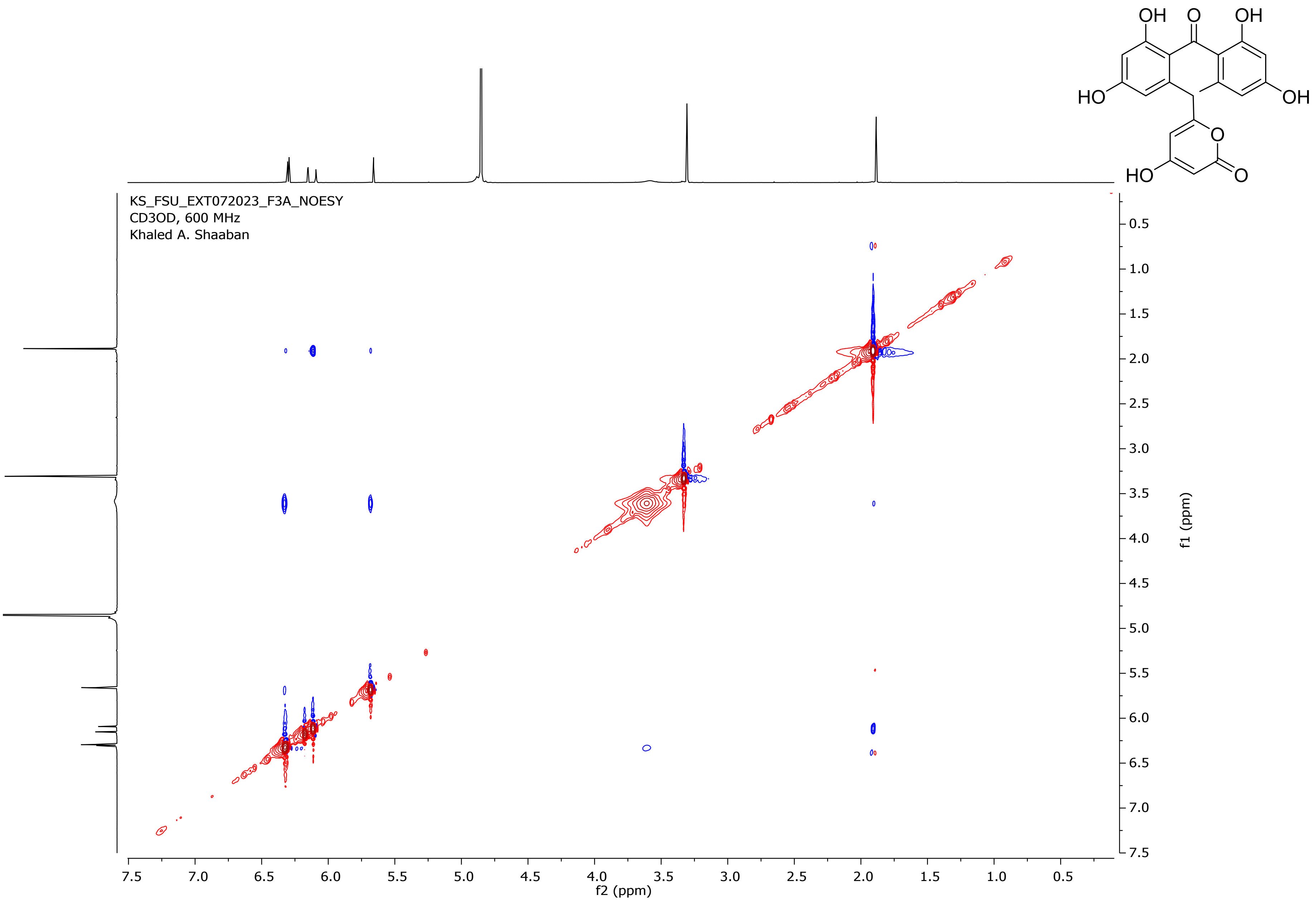

Figure S46. NOESY spectrum (CD_3OD, 600 MHz) of SEK15.

Print of window 80: MS Spectrum

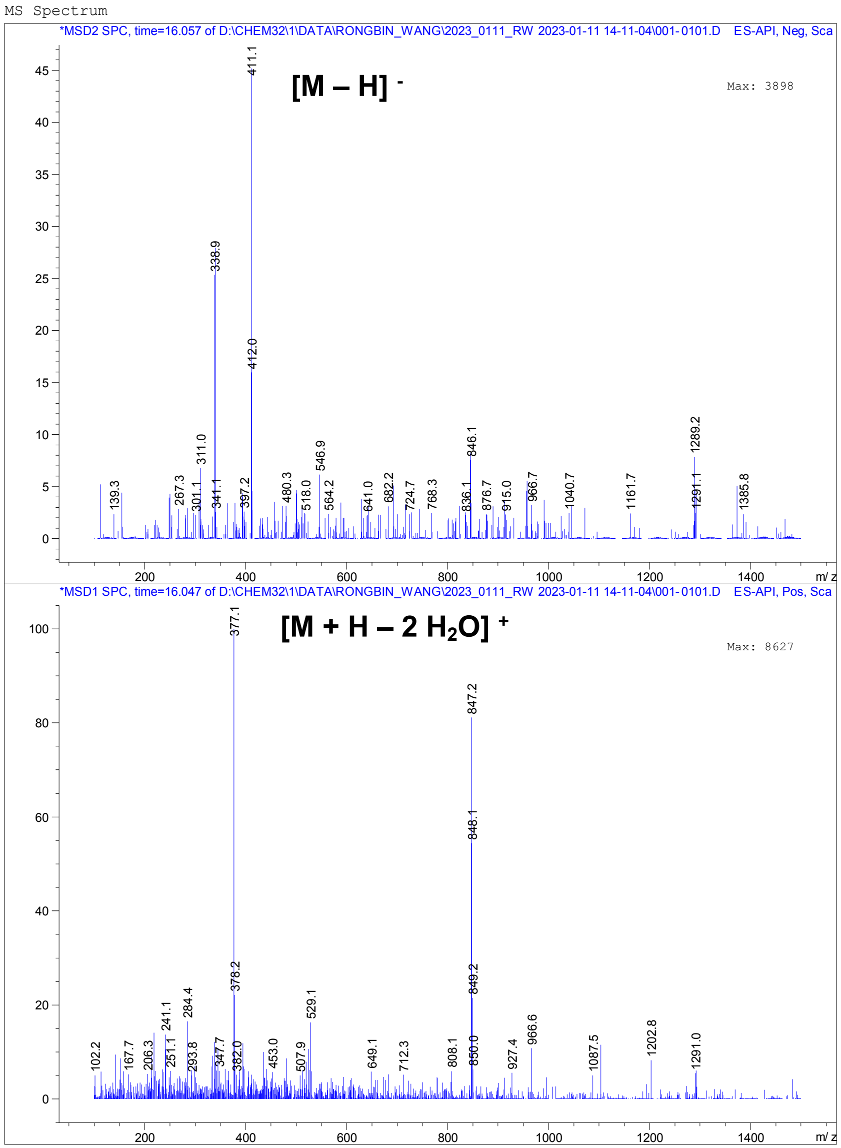

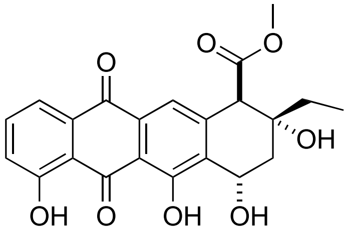

Agilent_LCMS 1/13/2023 3:23:56 PM SYSTEM Page 1 of 1

Figure S47. Mass spectrum of aklavinone (**1**) standard used for *in vitro* reactions.

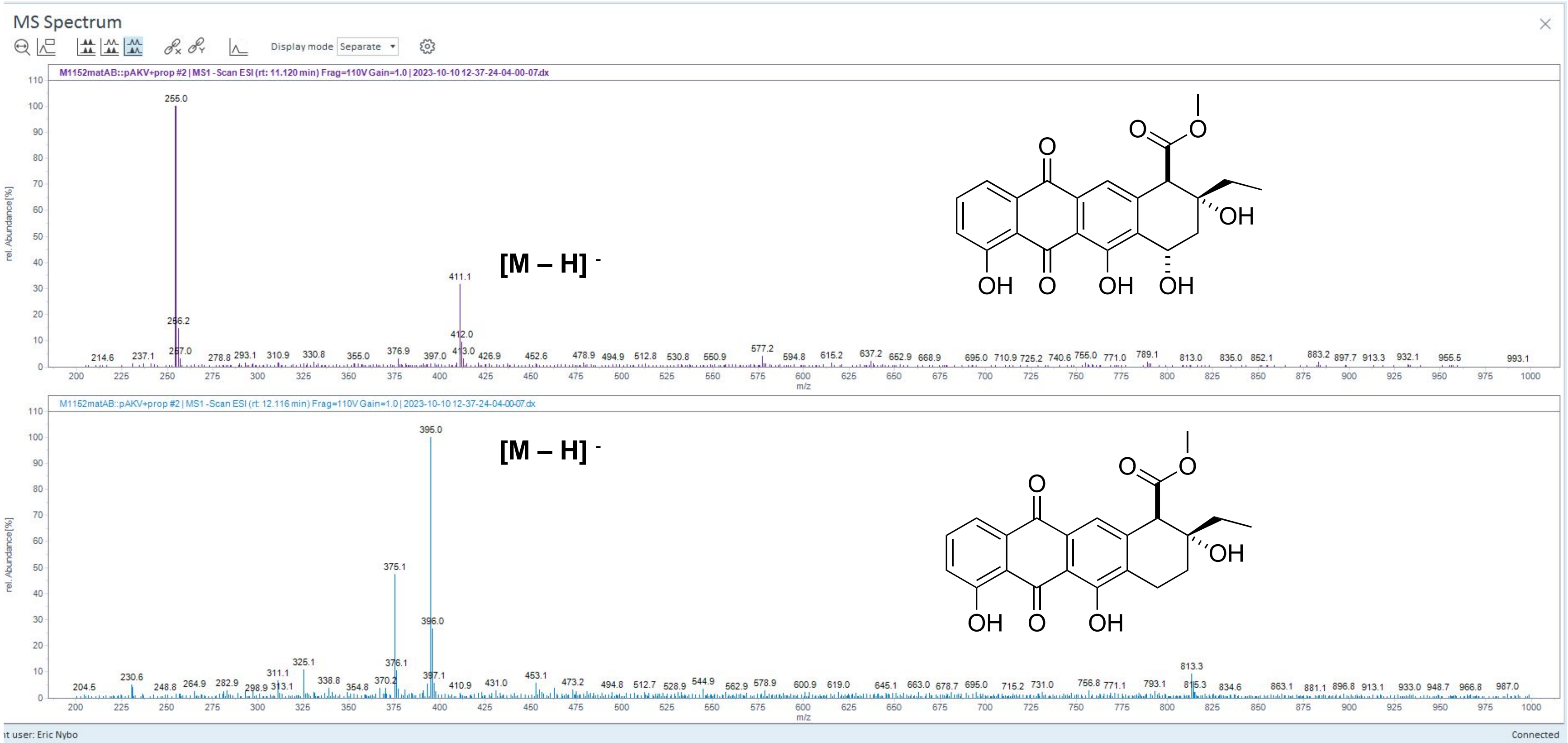

Figure S48. Mass spectrum of aklavinone (**1**) and 7-deoxy-aklavinone (**5**) identified from *in vivo* extracts.

**Sample Name**	KS_FSU_E1	**Position**	P1-B2	**Instrument Name**	Instrument 1
**User Name**		**Inj Vol**	5	**InjPosition**	
**Sample Type**	Sample	**IRM Calibration Status**	Success	**Data Filename**	KS_FSU_E1.d
**ACQ Method**	Zheng_AQC ACC short_Neg.m	**Comment**		**Acquired Time**	11/13/2023 4:42:07 PM (UTC-05:00)

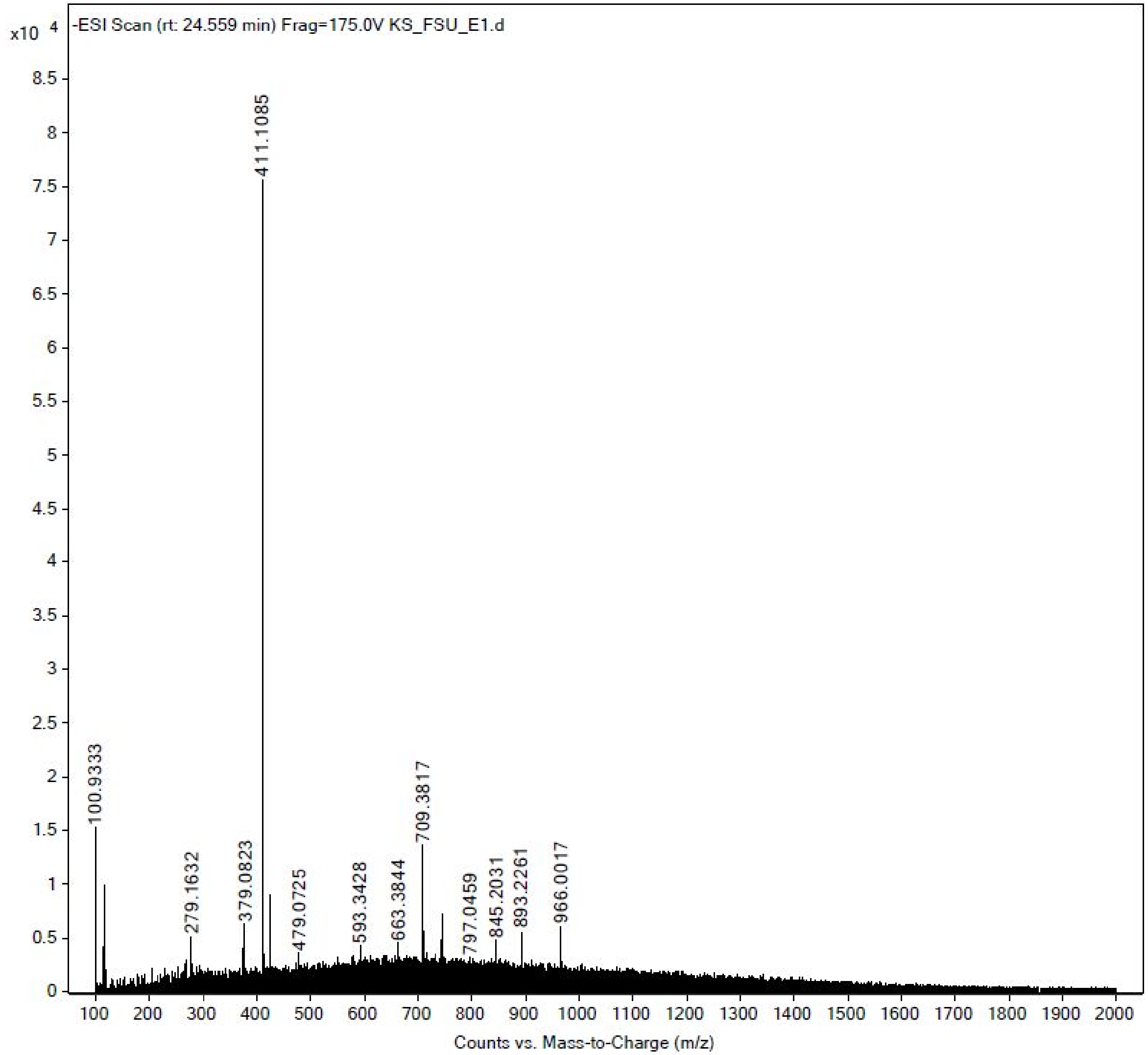

Figure S49. (-)-HRESI-MS spectrum of aklavinone (**1**).

Print of window 80: MS Spectrum

MS Spectrum

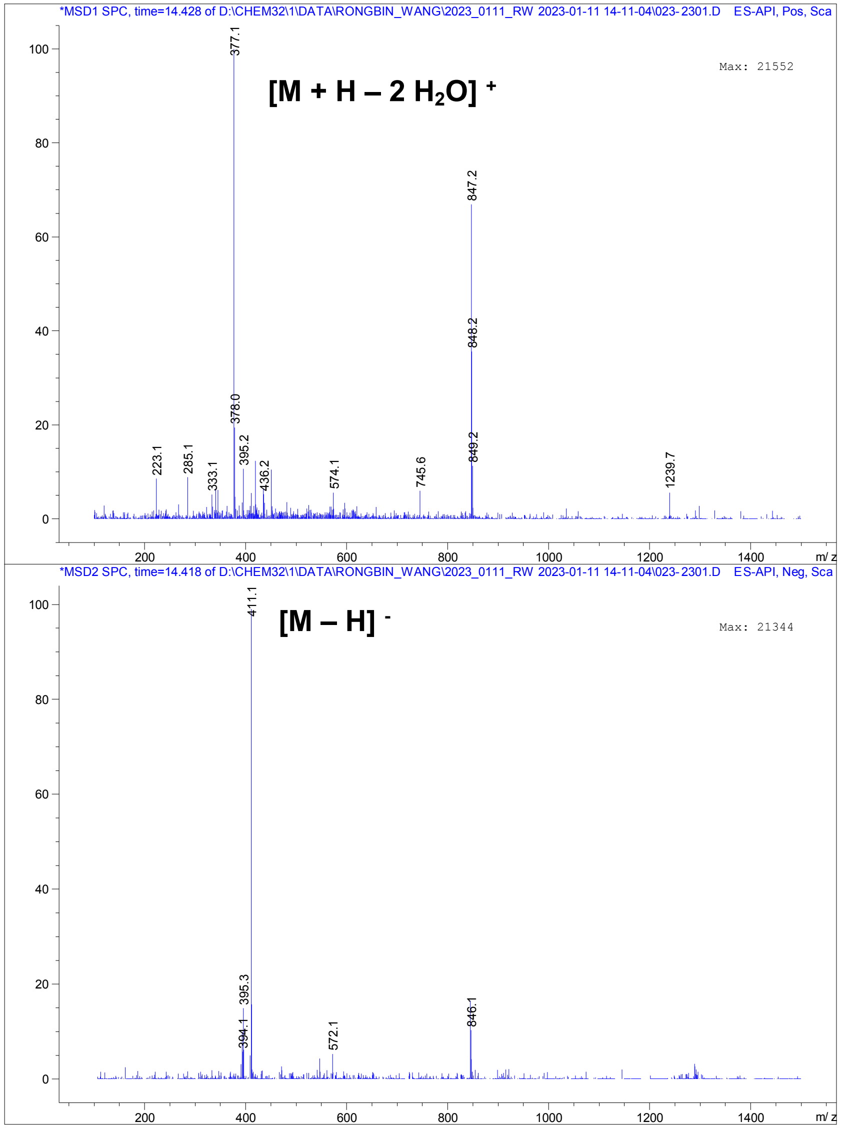

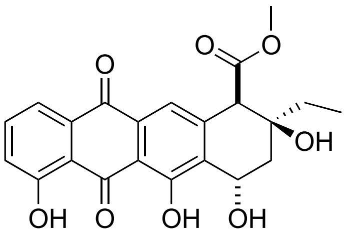

Agilent_LCMS 1/15/2023 11:40:34 PM SYSTEM Page 1 of 1

Figure S50. Mass spectrum of 9-*epi*-aklavinone (**2**) standard used for *in vitro* reactions.

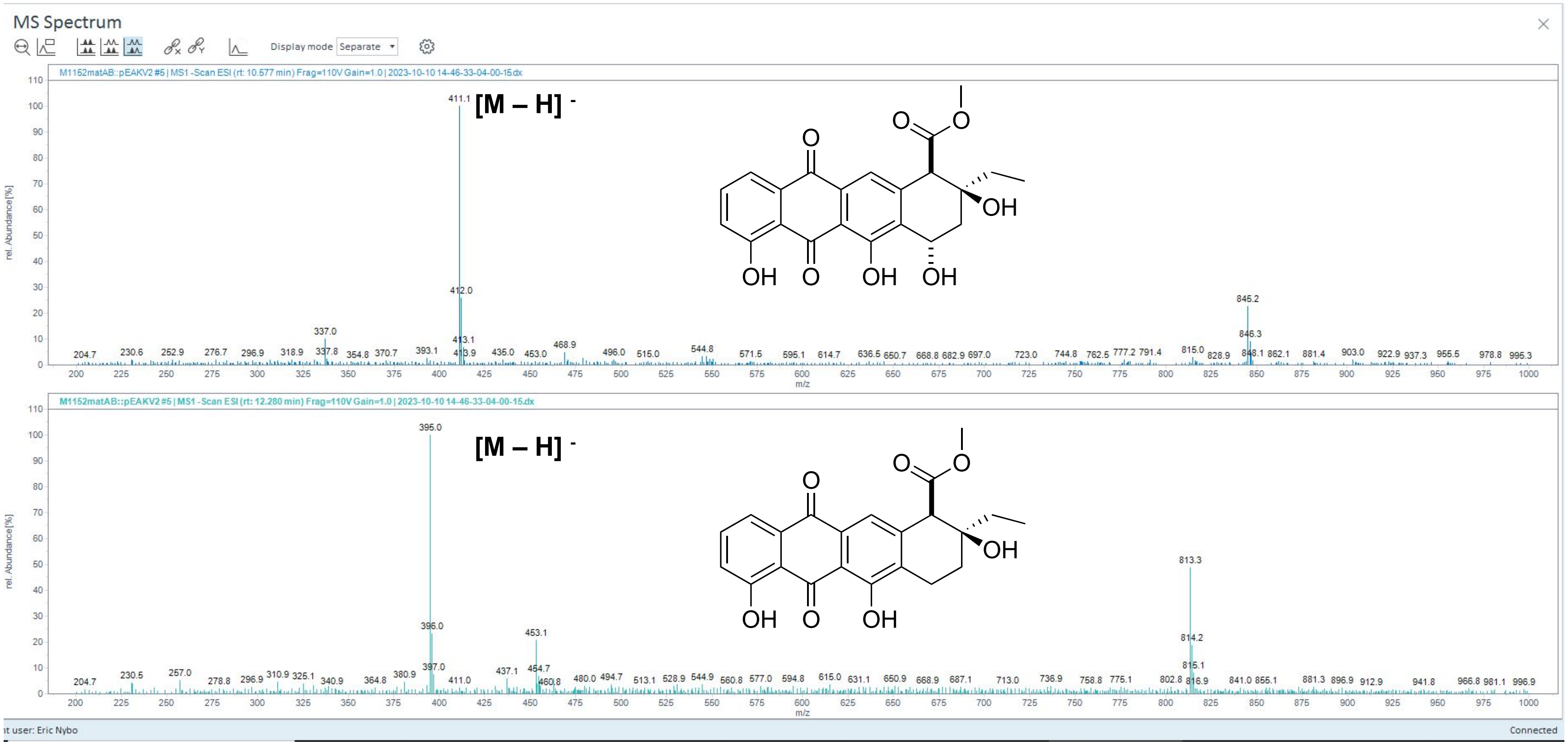

Figure S51. Mass spectrum of 9-*epi*-aklavinone (**2**) and 7-deoxy-9-*epi*-aklavinone (**6**) identified from *in vivo* extracts.

**Sample Name**	KS_FSU_E7	**Position**	P1-B8	**Instrument Name**	Instrument 1
**User Name**		**Inj Vol**	5	**InjPosition**	
**Sample Type**	Sample	**IRM Calibration Status**	Success	**Data Filename**	KS_FSU_E7.d
**ACQ Method**	Zheng_AQC ACC short_Neg.m	**Comment**		**Acquired Time**	11/13/2023 7:36:09 PM (UTC-05:00)

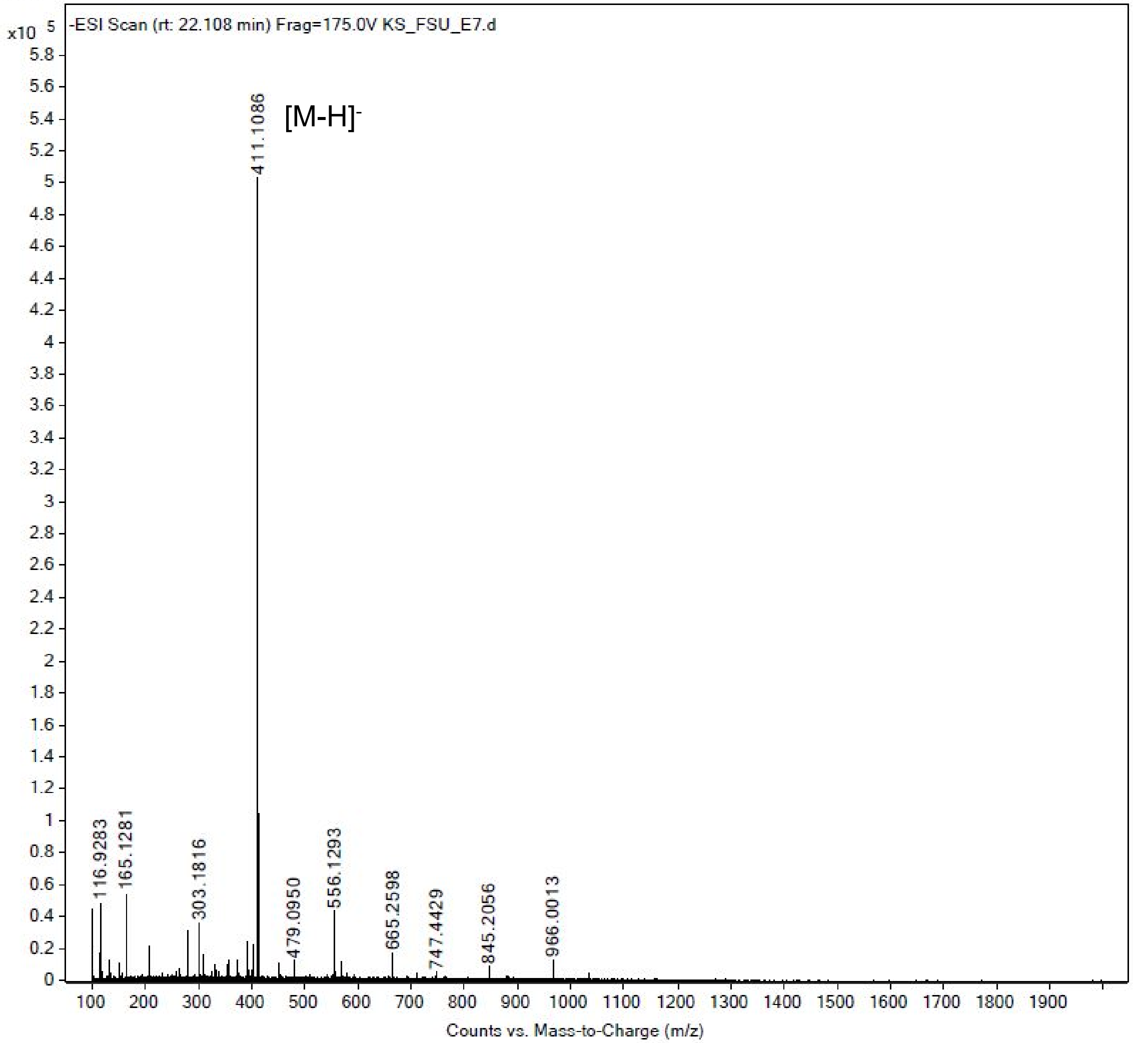

Figure S52. (-)-HRESI-MS spectrum of 9-*epi*-aklavinone (**2**).

Print of window 80: MS Spectrum

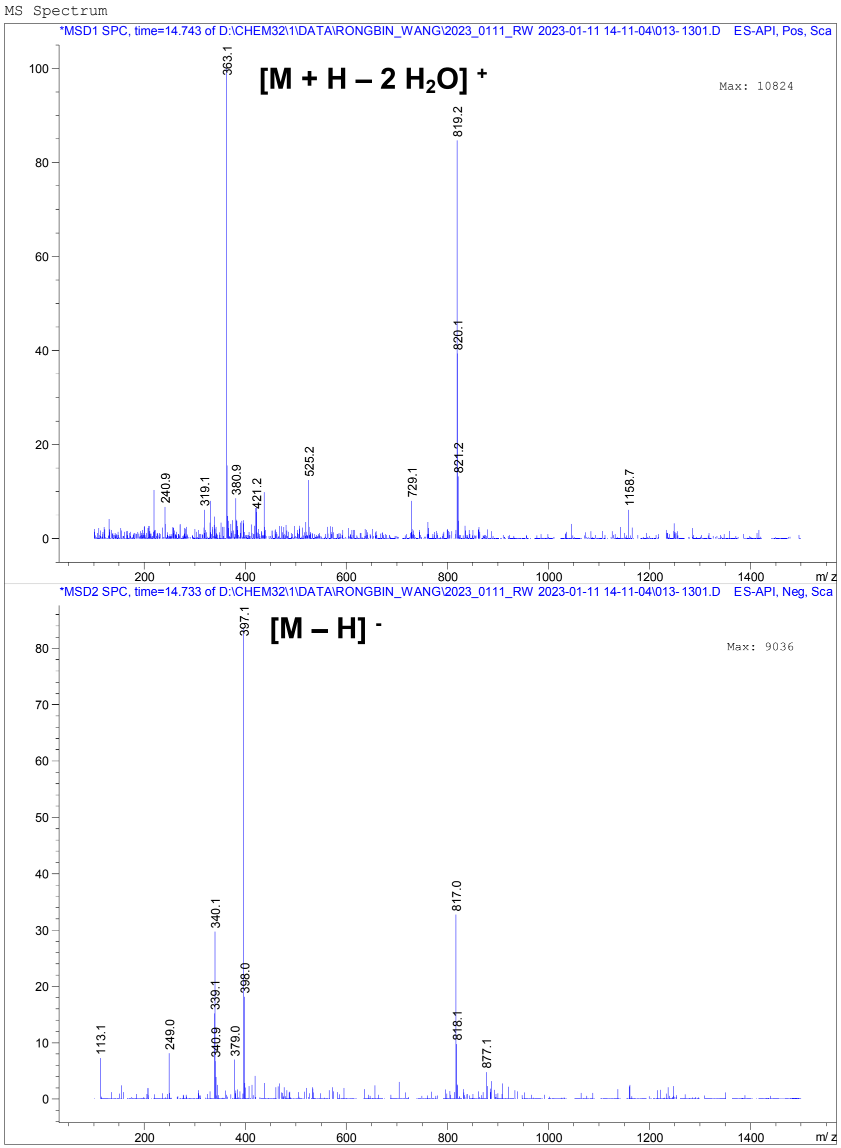

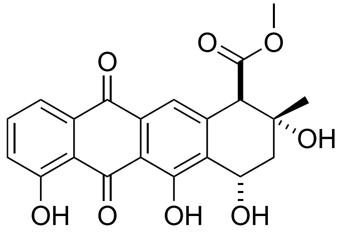

Agilent_LCMS 1/13/2023 3:31:50 PM SYSTEM Page 1 of 1

Figure S53. Mass spectrum of auramycinone (**3**) standard used in *in vitro* reactions.

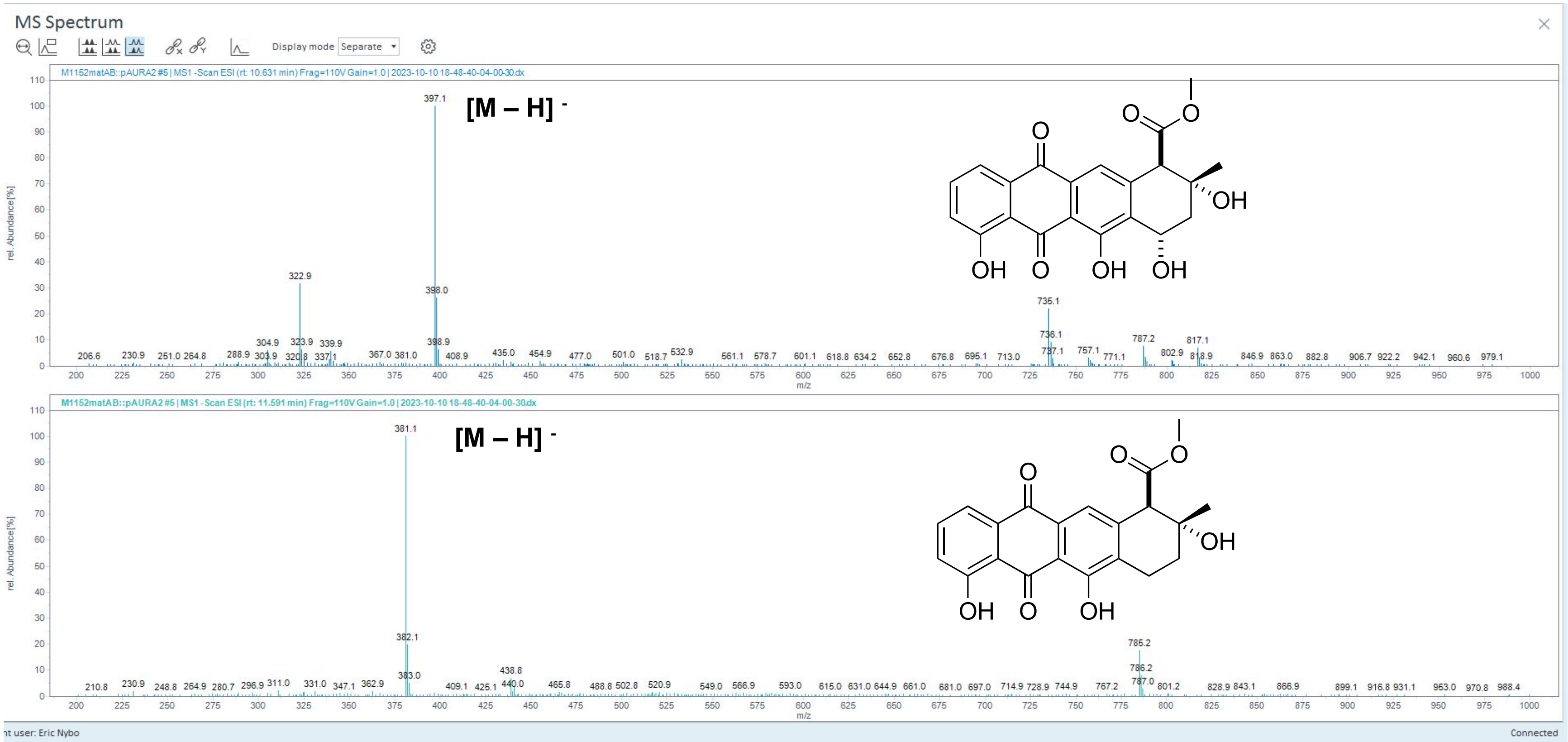

Figure S54. Mass spectrum of auramycinone (**3**) and 7-deoxy-auramycinone (**7**) identified from *in vivo* extracts.

**Sample Name**	KS_FSU_E14	**Position**	P1-C4	**Instrument Name**	Instrument 1
**User Name**		**Inj Vol**	5	**InjPosition**	
**Sample Type**	Sample	**IRM Calibration Status**	Success	**Data Filename**	KS_FSU_E14.d
**ACQ Method**	Zheng_AQC ACC short_Neg.m	**Comment**		**Acquired Time**	11/13/2023 10:59:14 PM (UTC-05:00)

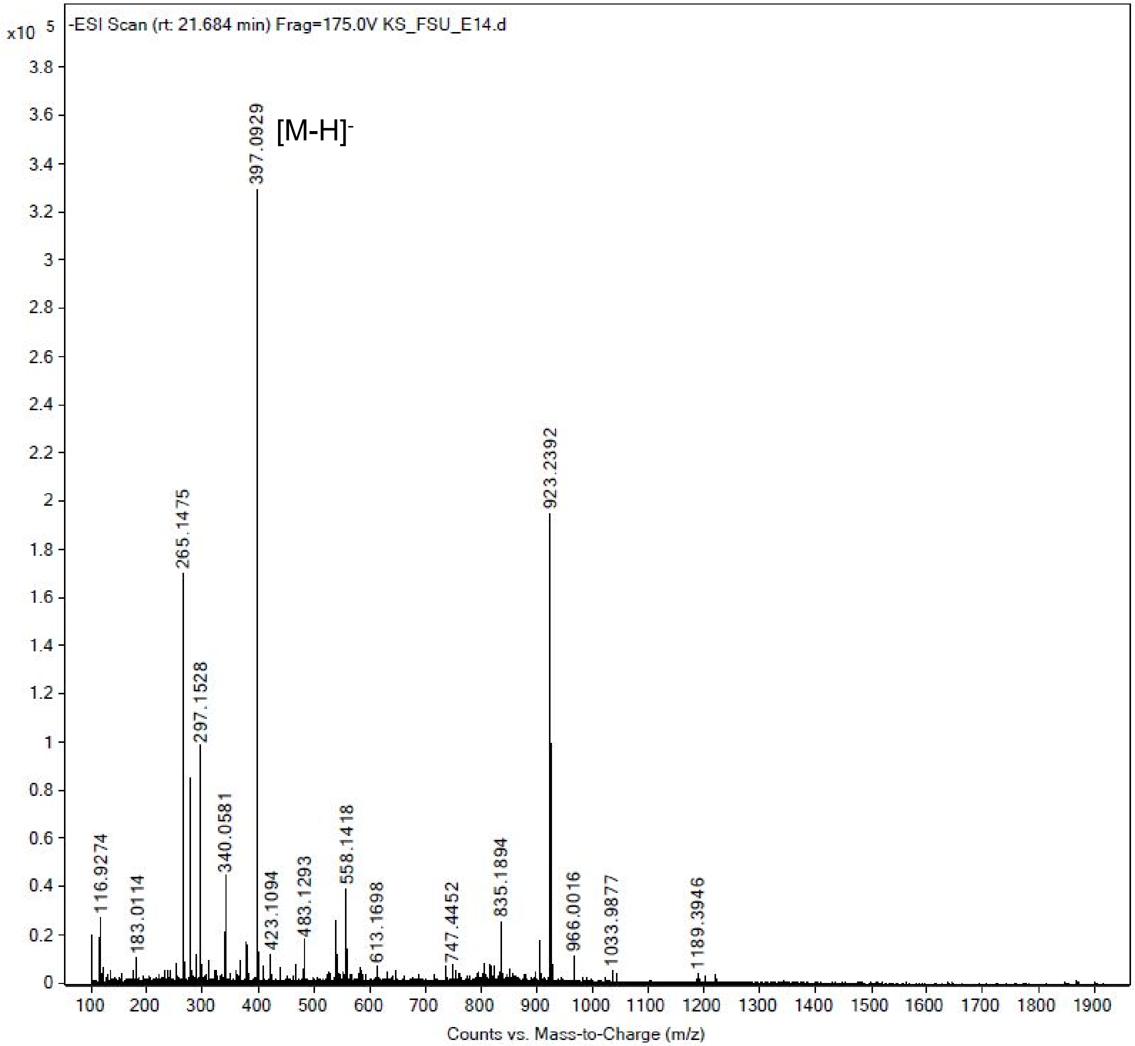

Figure S55. (-)-HRESI-MS spectrum of auramycinone (**3**).

Print of window 80: MS Spectrum

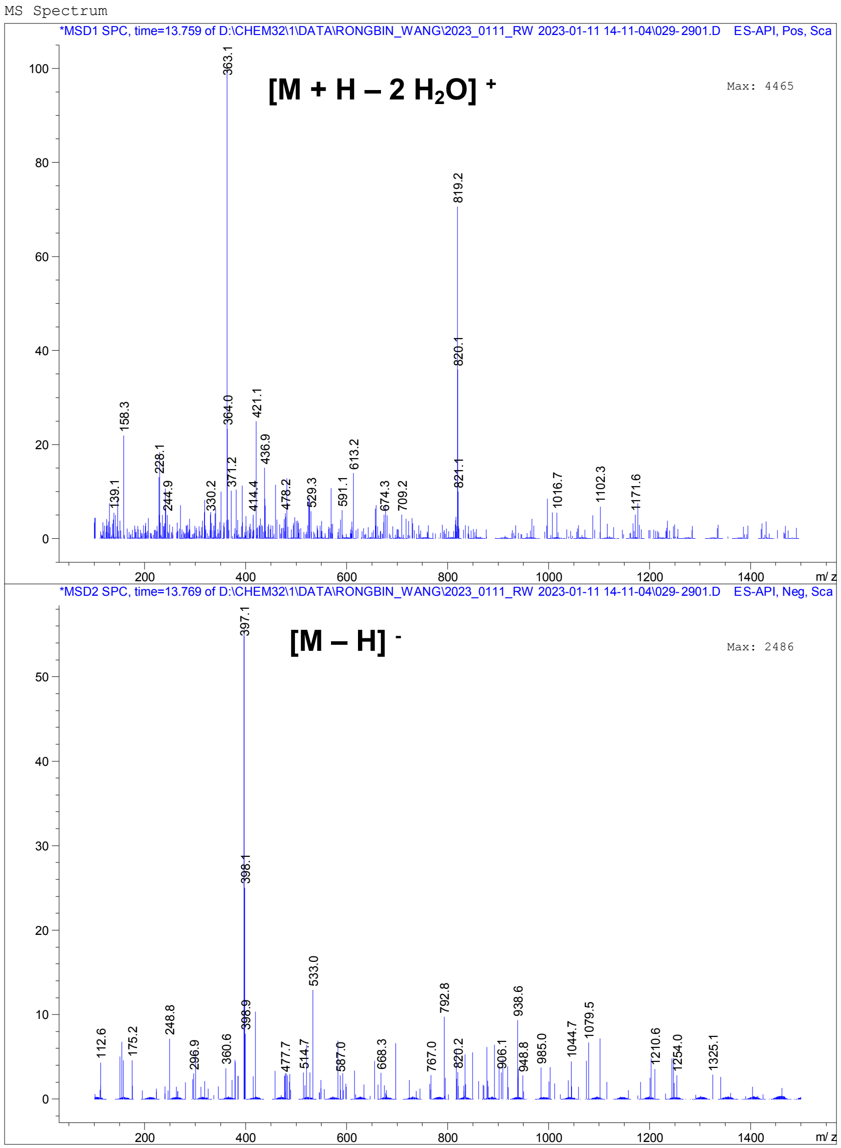

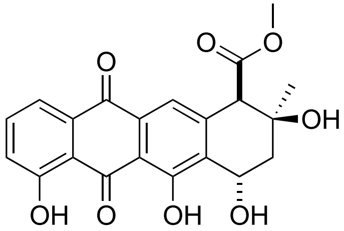

Agilent_LCMS 1/15/2023 11:48:01 PM SYSTEM Page 1 of 1

Figure S56. Mass spectrum of nogalamycinone (**4**) used in *in vitro* reactions.

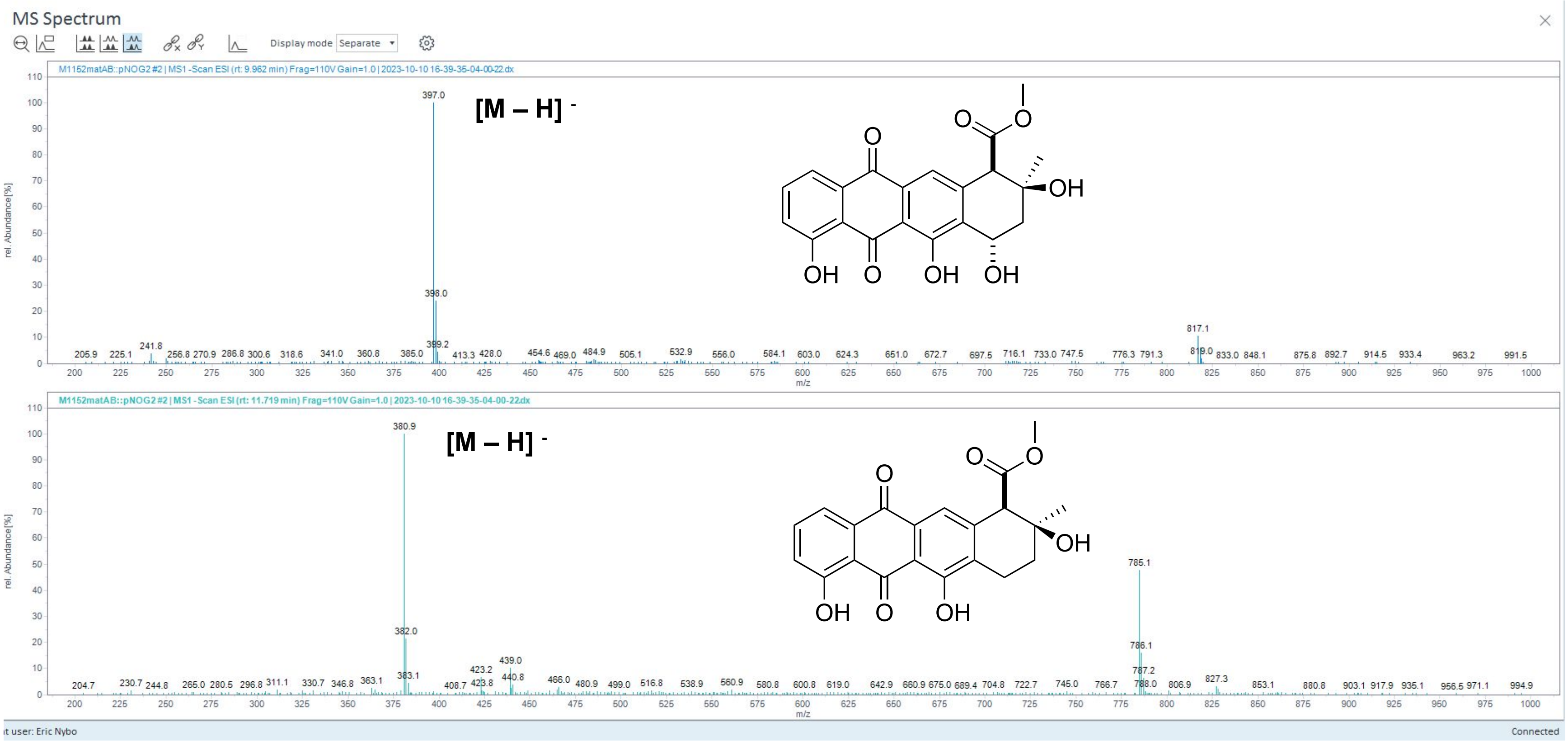

Figure S57. Mass spectrum of nogalamycinone (**4**) and 7-deoxy-nogalamycinone (**8**) identified in *in vivo* extracts.

**Sample Name**	KS_FSU_E18	**Position**	P1-C8	**Instrument Name**	Instrument 1
**User Name**		**Inj Vol**	5	**InjPosition**	
**Sample Type**	Sample	**IRM Calibration Status**	Success	**Data Filename**	KS_FSU_E18.d
**ACQ Method**	Zheng_AQC ACC short_Neg.m	**Comment**		**Acquired Time**	11/14/2023 12:55:17 AM (UTC-05:00)

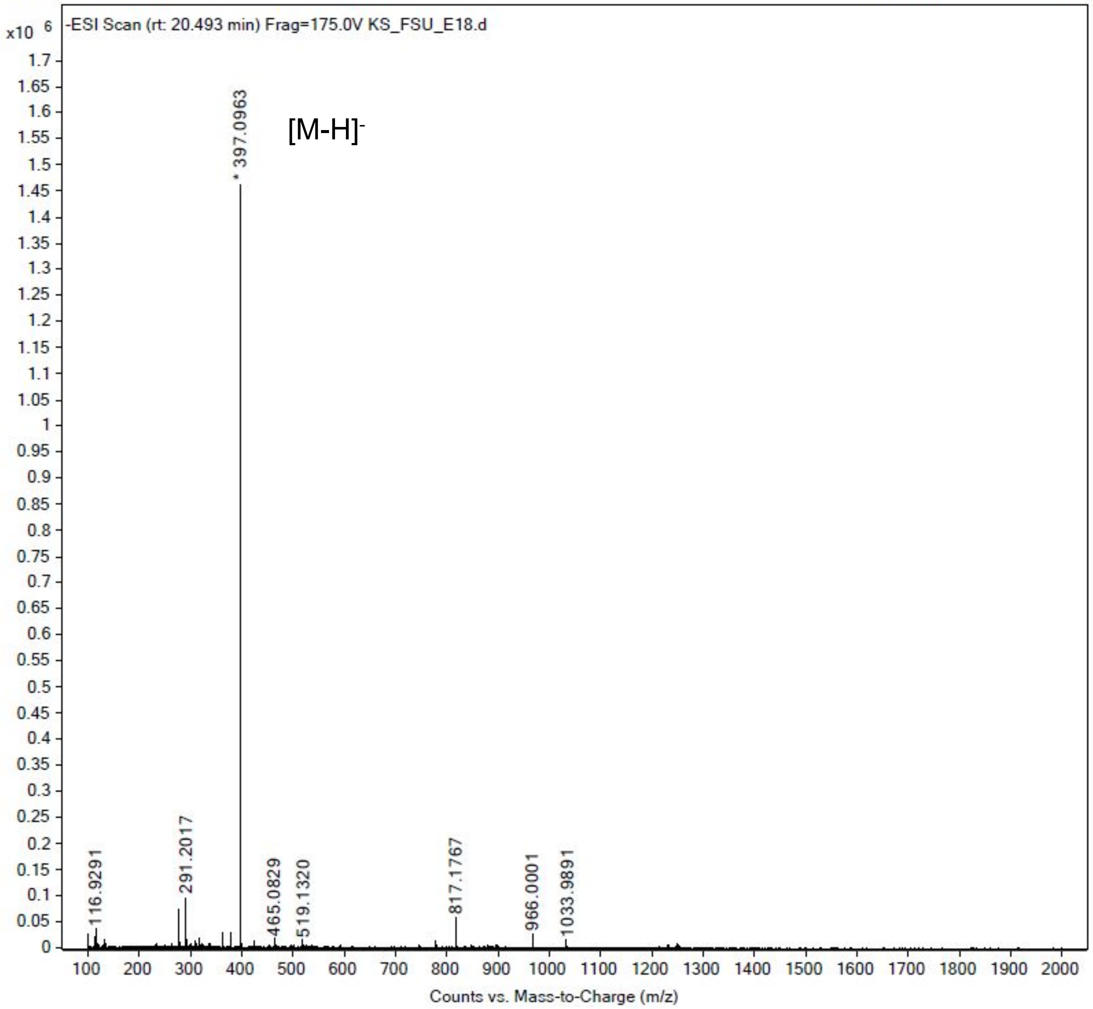

Figure S58. (-)-HRESI-MS spectrum of nogalamycinone (**4**).

**Sample Name**	KS_FSU_E3	**Position**	P1-B4	**Instrument Name**	Instrument 1
**User Name**		**Inj Vol**	5	**InjPosition**	
**Sample Type**	Sample	**IRM Calibration Status**	Success	**Data Filename**	KS_FSU_E3.d
**ACQ Method**	Zheng_AQC ACC short_Neg.m	**Comment**		**Acquired Time**	11/13/2023 5:40:07 PM (UTC-05:00)

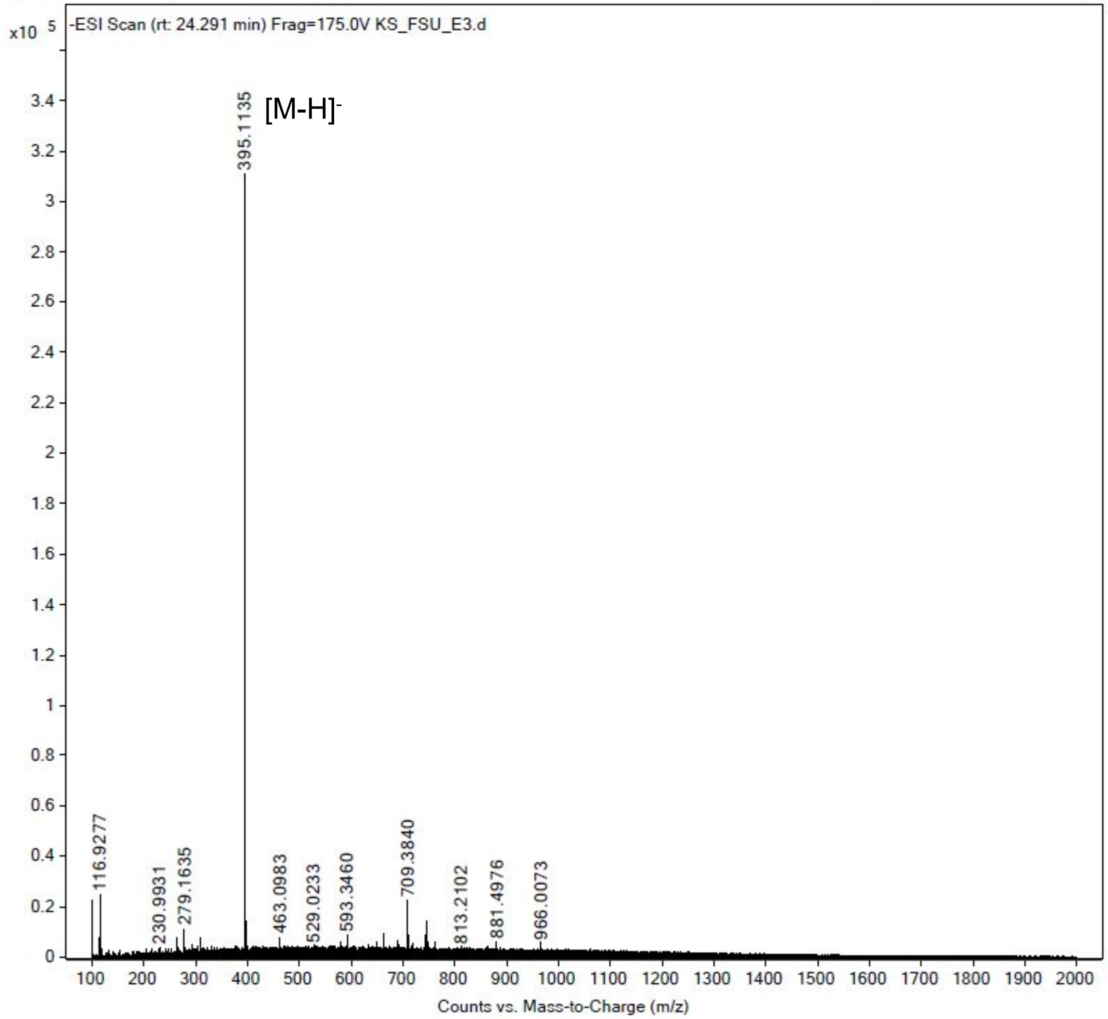

Figure S59. (-)-HRESI-MS spectrum of 7-deoxy-aklavinone (**5**).

**Sample Name**	KS_FSU_E8	**Position**	P1-B9	**Instrument Name**	Instrument 1
**User Name**		**Inj Vol**	5	**InjPosition**	
**Sample Type**	Sample	**IRM Calibration Status**	Success	**Data Filename**	KS_FSU_E8.d
**ACQ Method**	Zheng_AQC ACC short_Neg.m	**Comment**		**Acquired Time**	11/13/2023 8:05:10 PM (UTC-05:00)

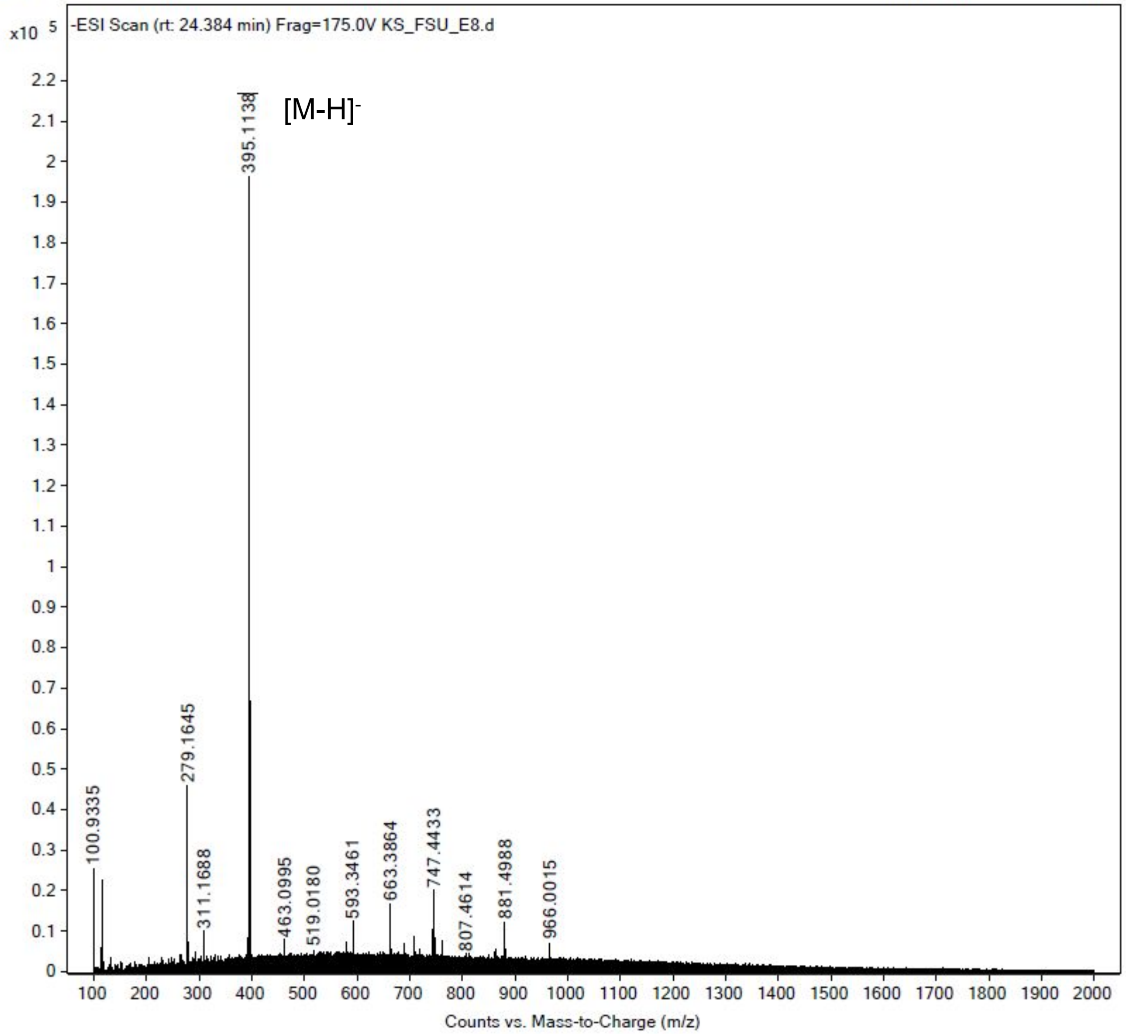

Figure S60. (-)-HRESI-MS spectrum of 7-deoxy-9-*epi*-aklavinone (**6**).

**Sample Name**	KS_FSU_E11	**Position**	P1-C1	**Instrument Name**	Instrument 1
**User Name**		**Inj Vol**	5	**InjPosition**	
**Sample Type**	Sample	**IRM Calibration Status**	Success	**Data Filename**	KS_FSU_E11.d
**ACQ Method**	Zheng_AQC ACC short_Neg.m	**Comment**		**Acquired Time**	11/13/2023 9:32:12 PM (UTC-05:00)

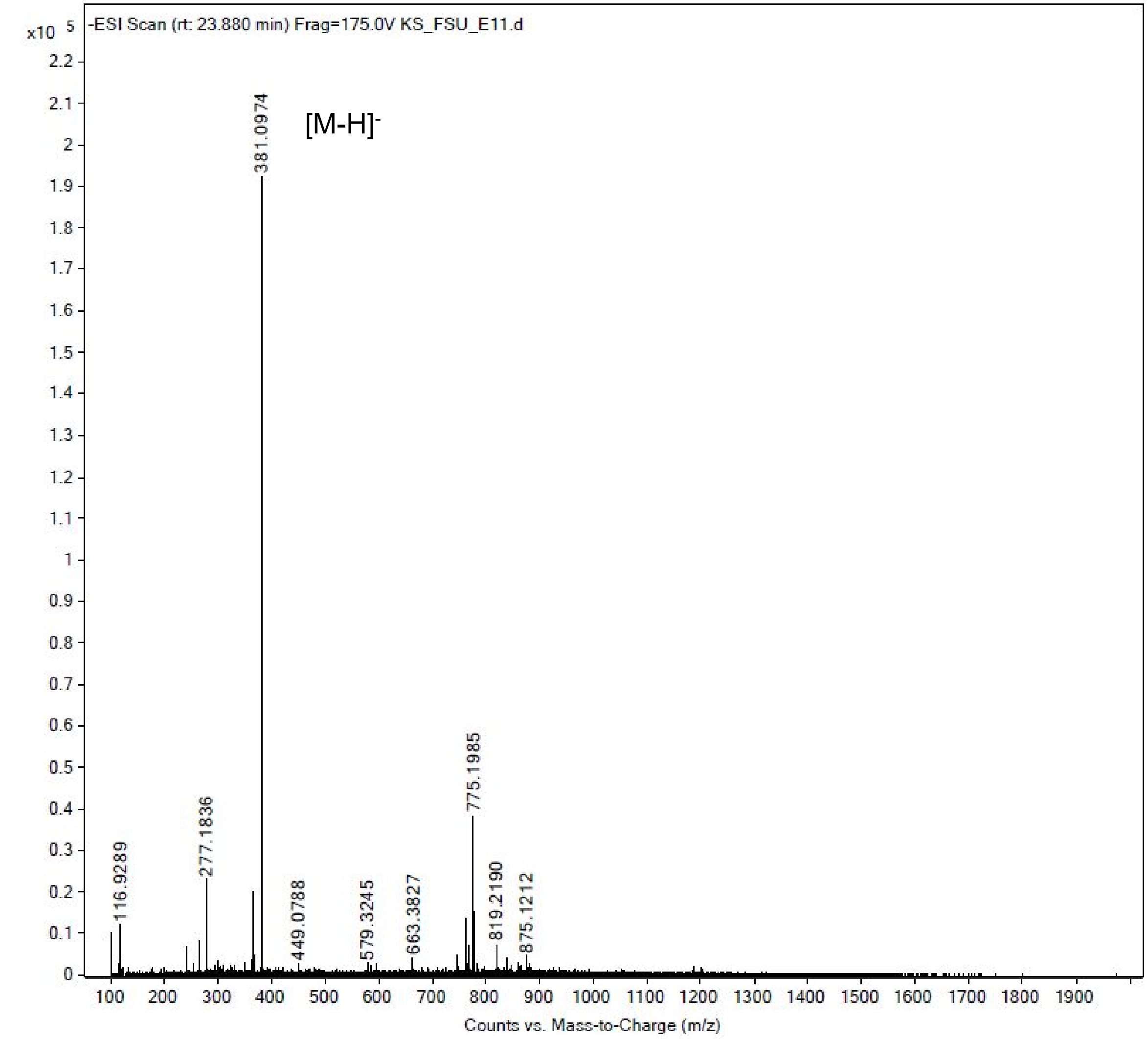

Figure S61. (-)-HRESI-MS spectrum of 7-deoxy-auramycinone (**7**).

**Sample Name**	KS_FSU_E15	**Position**	P1-C5	**Instrument Name**	Instrument 1
**User Name**		**Inj Vol**	5	**InjPosition**	
**Sample Type**	Sample	**IRM Calibration Status**	Success	**Data Filename**	KS_FSU_E15.d
**ACQ Method**	Zheng_AQC ACC short_Neg.m	**Comment**		**Acquired Time**	11/13/2023 11:28:15 PM (UTC-05:00)

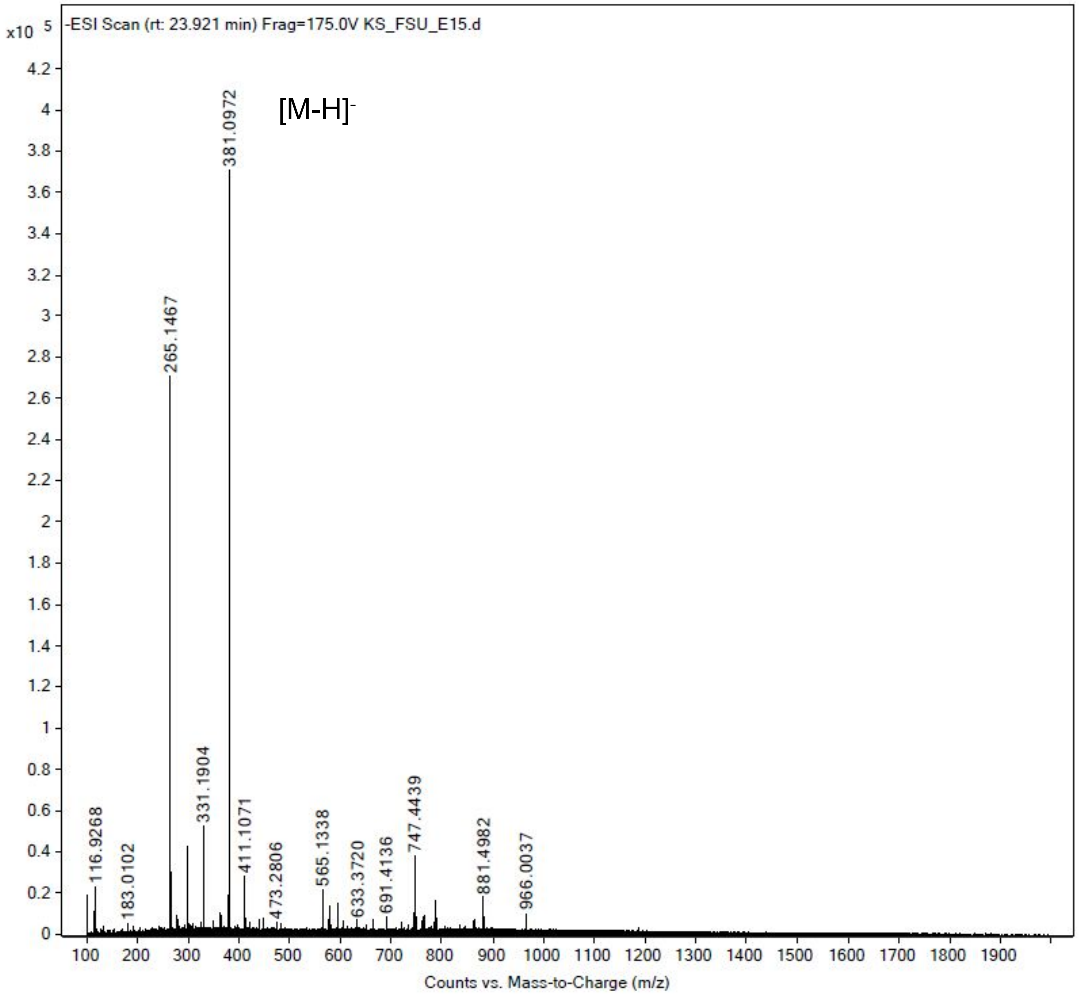

Figure S62. (-)-HRESI-MS spectrum of 7-deoxy-nogalamycinone (**8**).

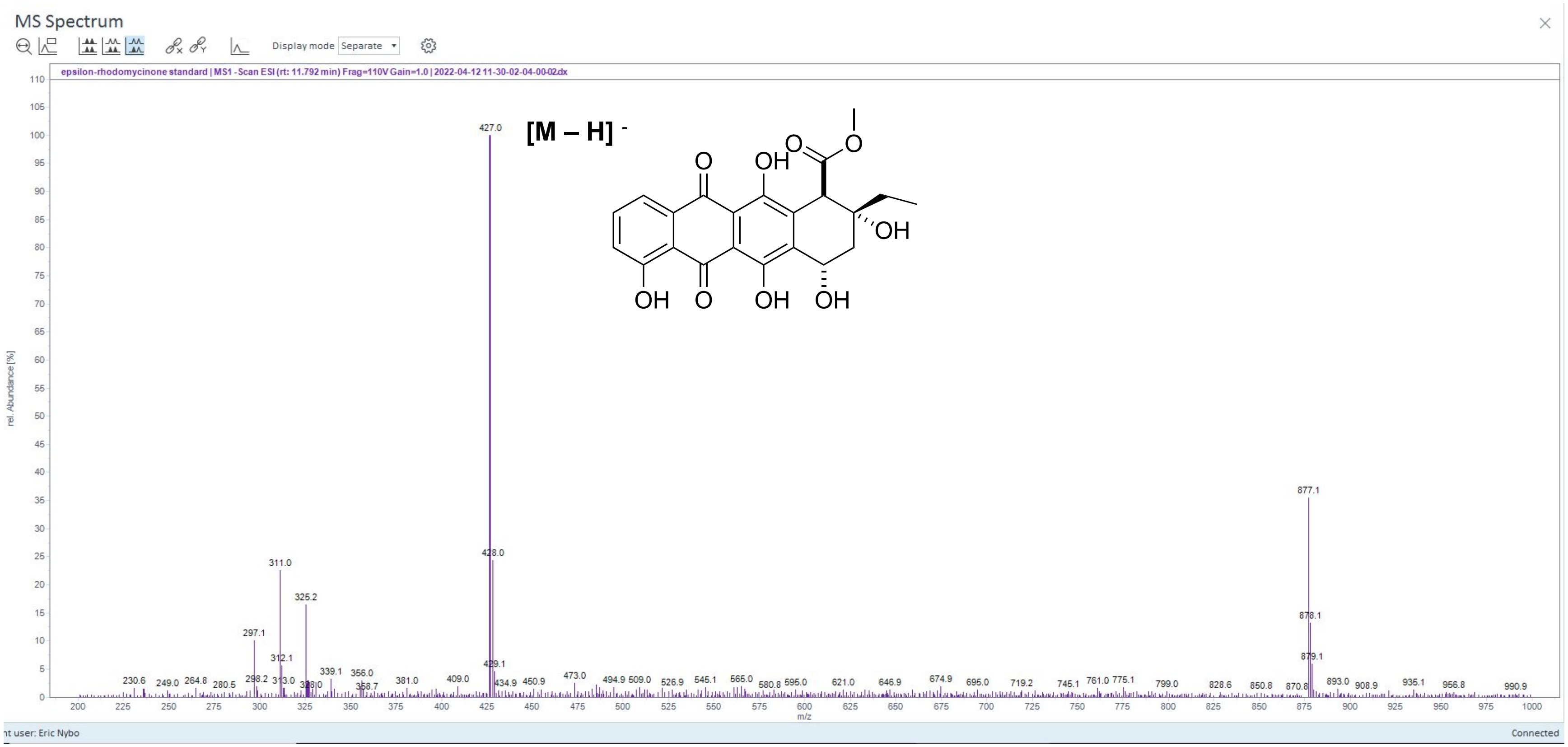

Figure S63. Mass spectrum of epsilon-rhodomycinone (**15**) standard.

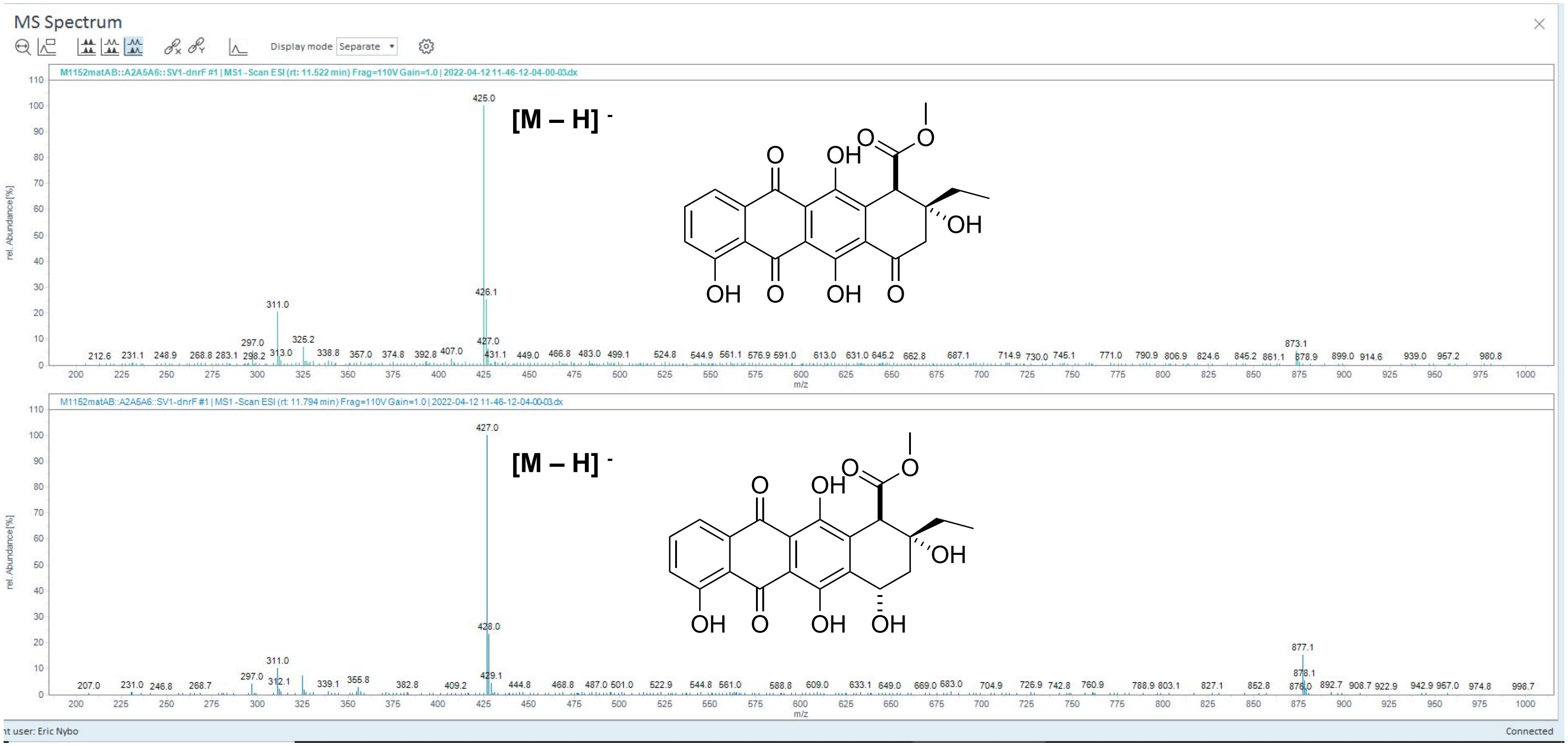

Figure S64. Mass spectrum of maggiemycin and epsilon-rhodomycinone (**15**) identified from strain co-expressing the aklavinone pathway and *dnrF.*

**Sample Name**	KS_FSU_E1	**Position**	P1-B2	**Instrument Name**	Instrument 1
**User Name**		**Inj Vol**	5	**InjPosition**	
**Sample Type**	Sample	**IRM Calibration Status**	Success	**Data Filename**	KS_FSU_E1.d
**ACQ Method**	Zheng_AQC ACC short_Neg.m	**Comment**		**Acquired Time**	11/13/2023 4:42:07 PM (UTC-05:00)

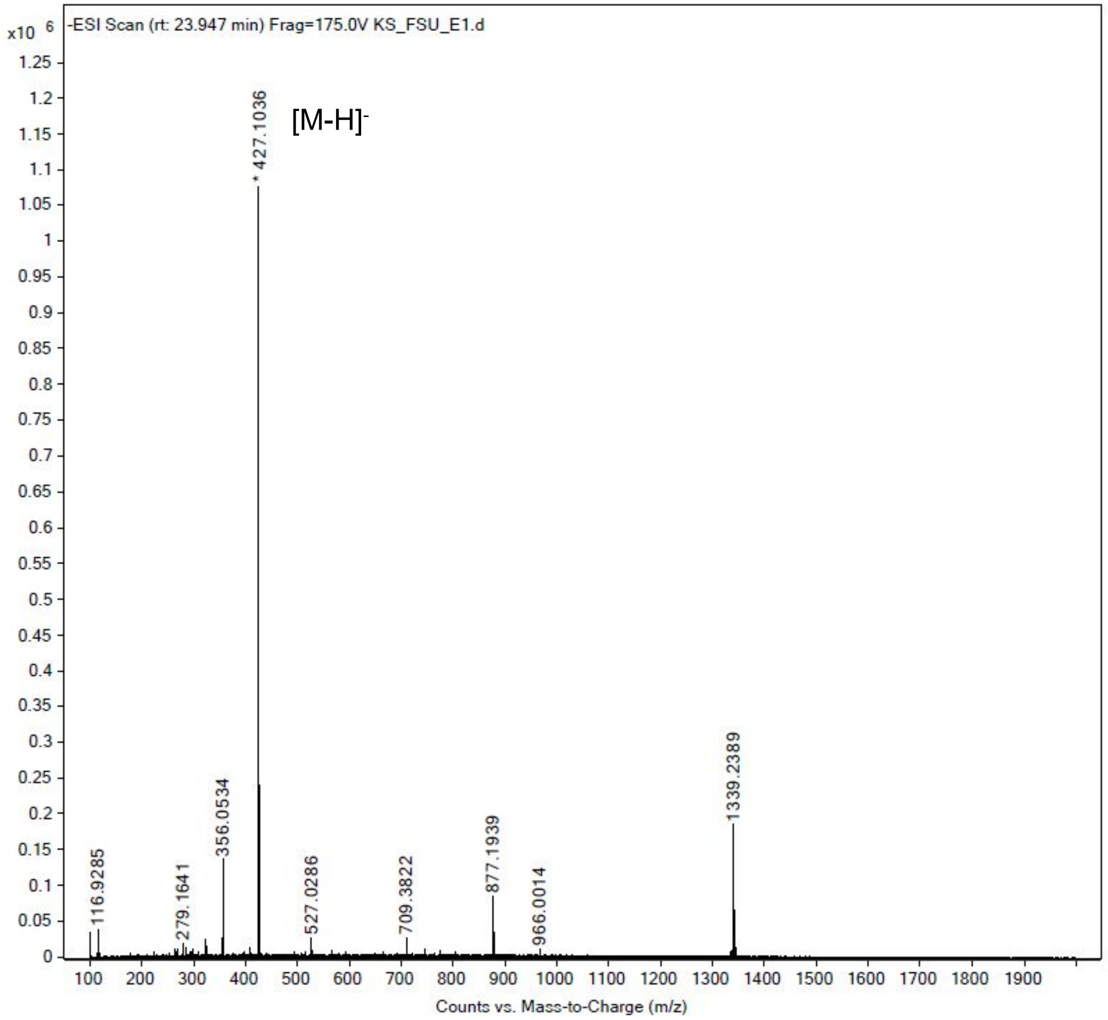

Figure S65. (-)-HRESI-MS spectrum of epsilon-rhodomycinone (**15**).

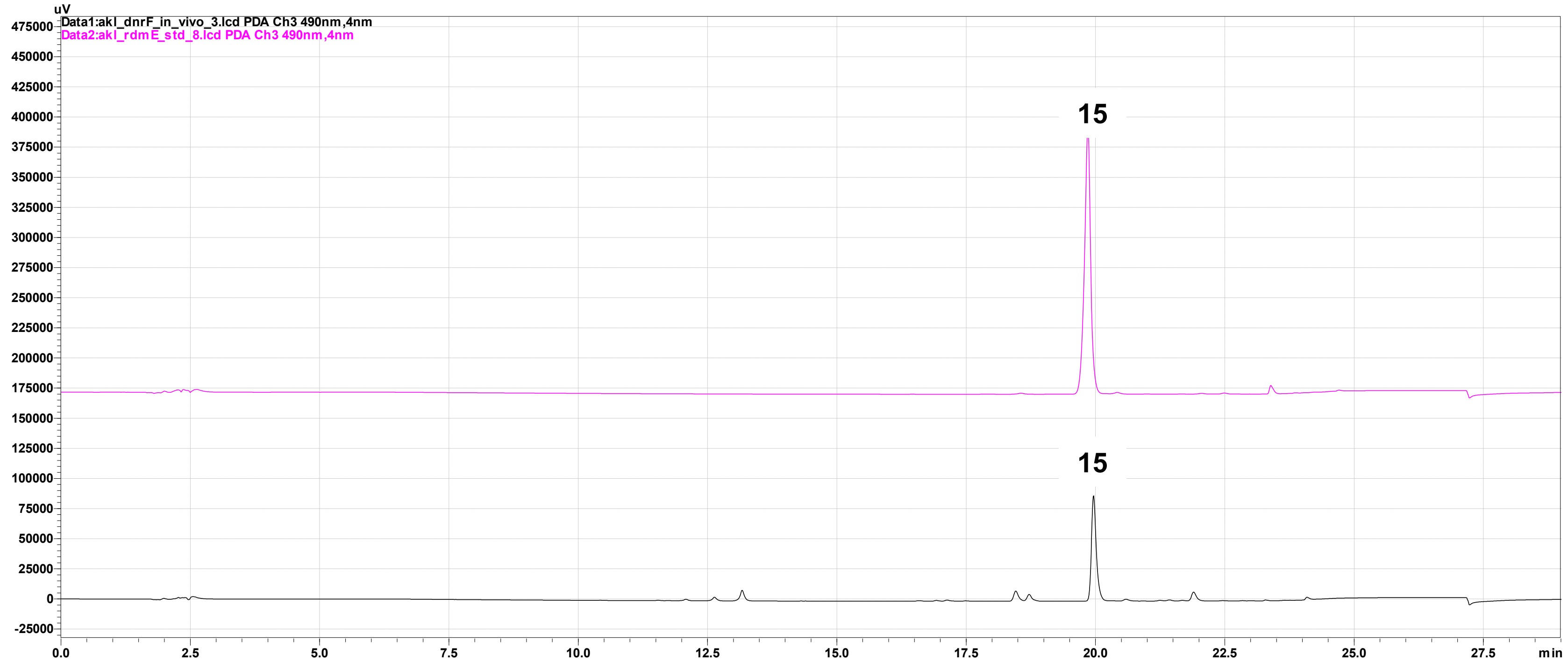

Figure S66. Alignment of epsilon-rhodomycinone (**15**) produced both *in vivo* and *in vitro*.

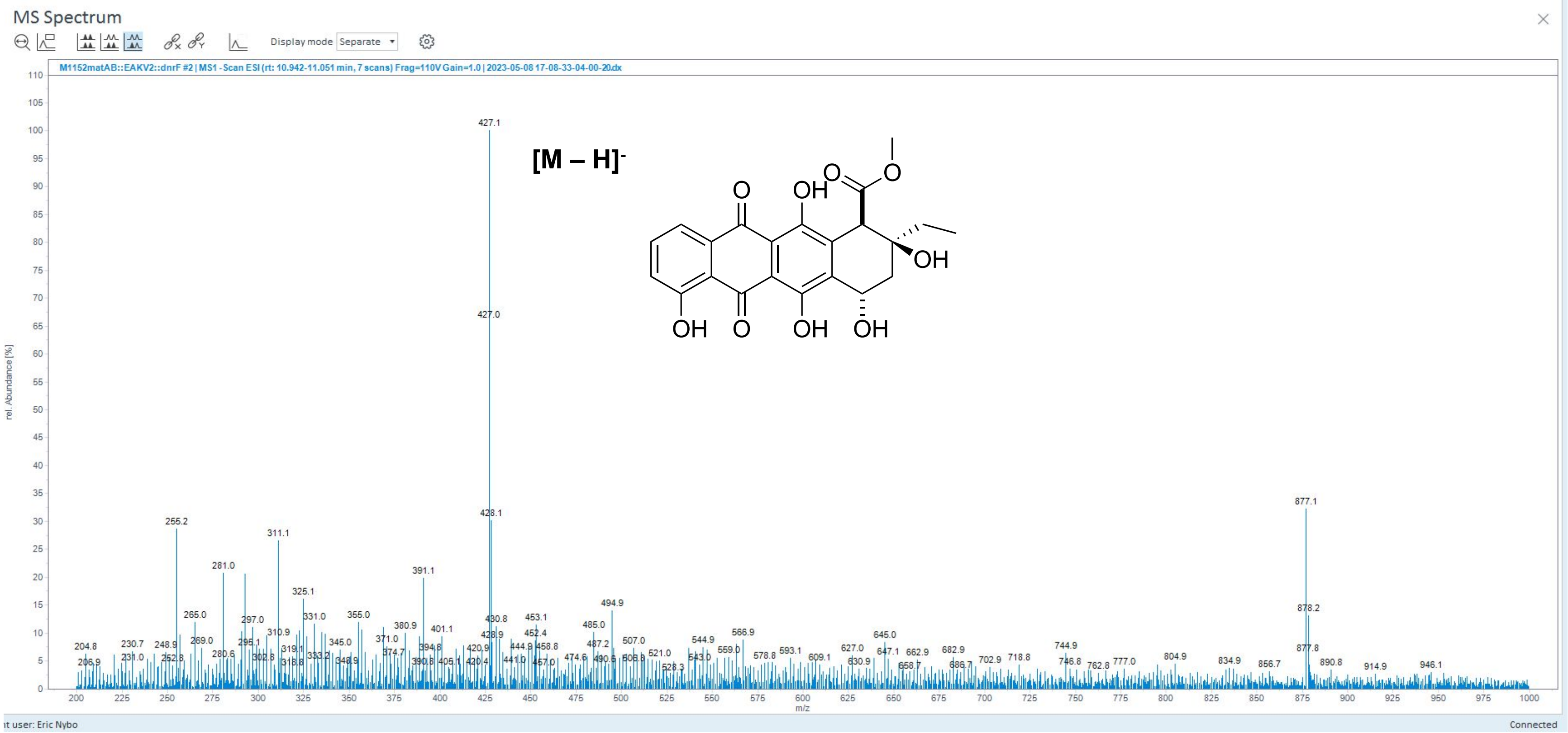

Figure S67. Mass spectrum of 11-hydroxy-9-*epi*-aklavinone (**16**) identified from strain co-expressing the 9-*epi*-aklavinone pathway and *dnrF.*

**Sample Name**	KS_FSU_E6	**Position**	P1-B7	**Instrument Name**	Instrument 1
**User Name**		**Inj Vol**	5	**InjPosition**	
**Sample Type**	Sample	**IRM Calibration Status**	Success	**Data Filename**	KS_FSU_E6.d
**ACQ Method**	Zheng_AQC ACC short_Neg.m	**Comment**		**Acquired Time**	11/13/2023 7:07:10 PM (UTC-05:00)

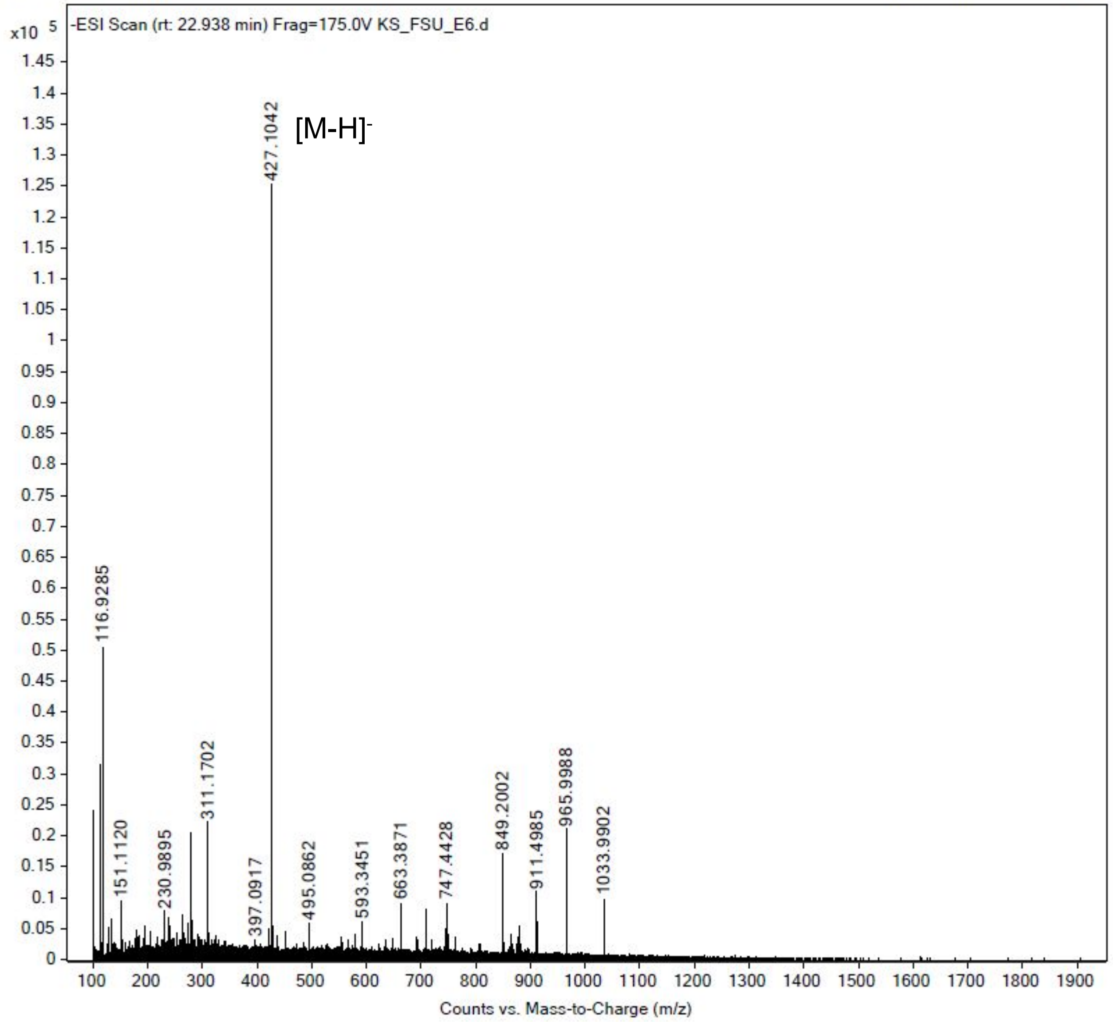

Figure S68. (-)-HRESI-MS spectrum of 11-hydroxy-9-*epi*-aklavinone (**16**).

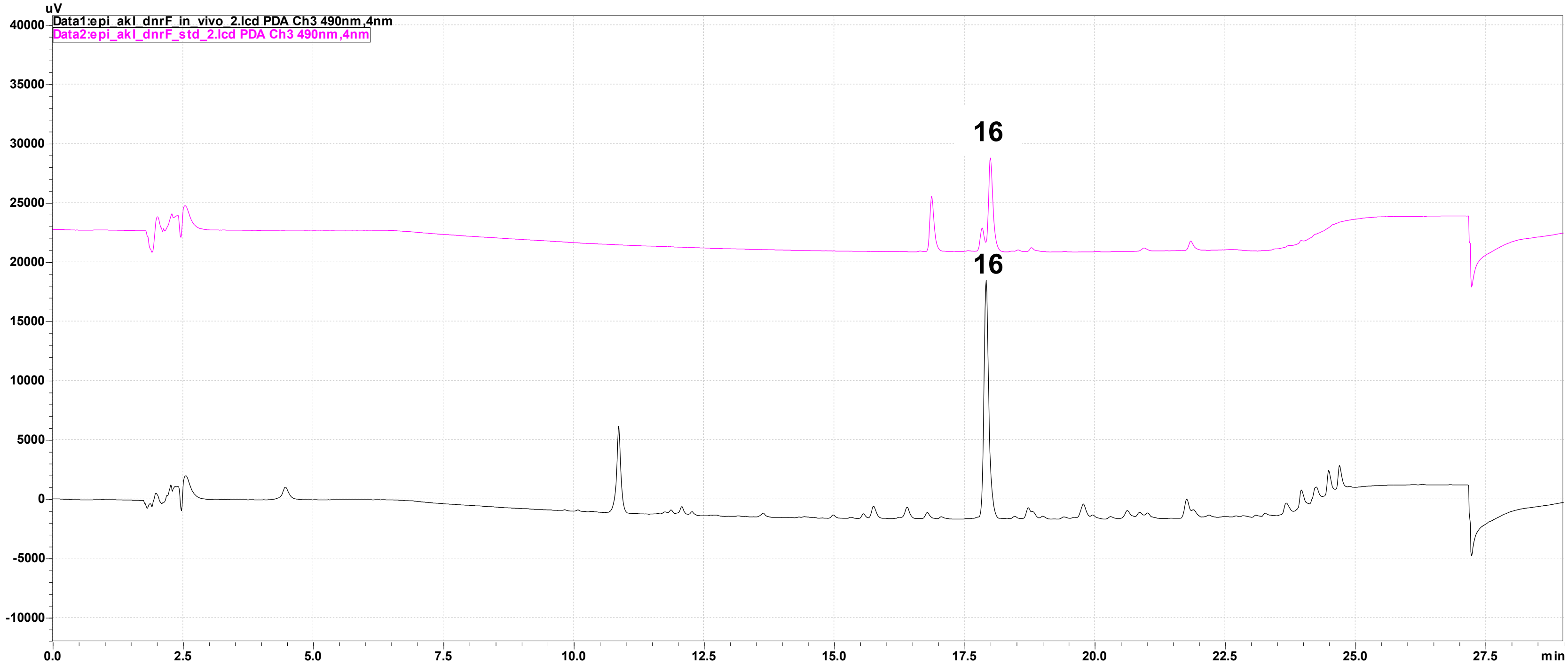

Figure S69. Alignment of 11-hydroxy-9-*epi*-aklavinone (**16**) produced both *in vivo* and *in vitro*.

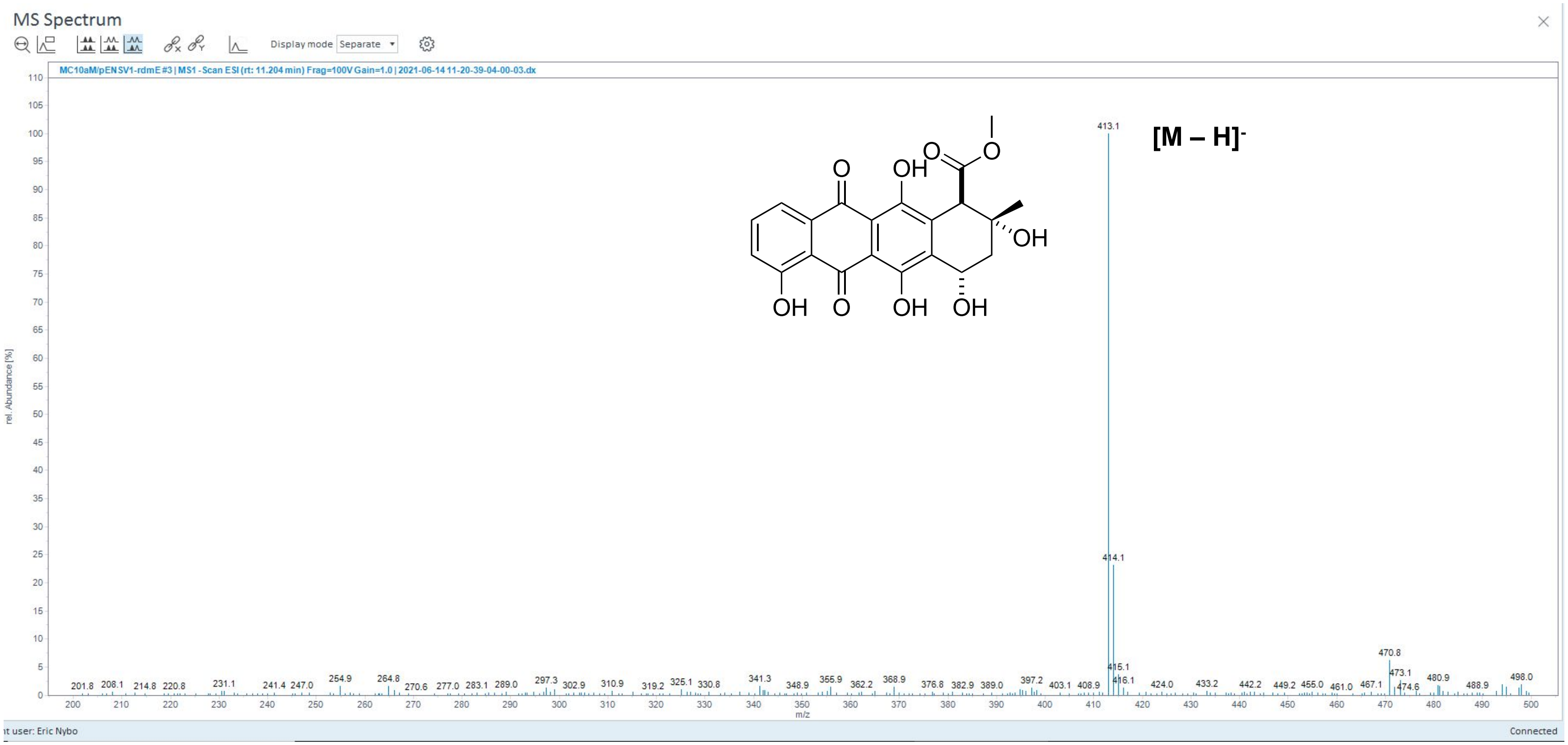

Figure S70. Mass spectrum of 11-hydroxy-auramycinone (**17**) identified from strain co-expressing the auramycinone pathway and *rdmE*.

**Sample Name**	KS_FSU_E11	**Position**	P1-C1	**Instrument Name**	Instrument 1
**User Name**		**Inj Vol**	5	**InjPosition**	
**Sample Type**	Sample	**IRM Calibration Status**	Success	**Data Filename**	KS_FSU_E11.d
**ACQ Method**	Zheng_AQC ACC short_Neg.m	**Comment**		**Acquired Time**	11/13/2023 9:32:12 PM (UTC-05:00)

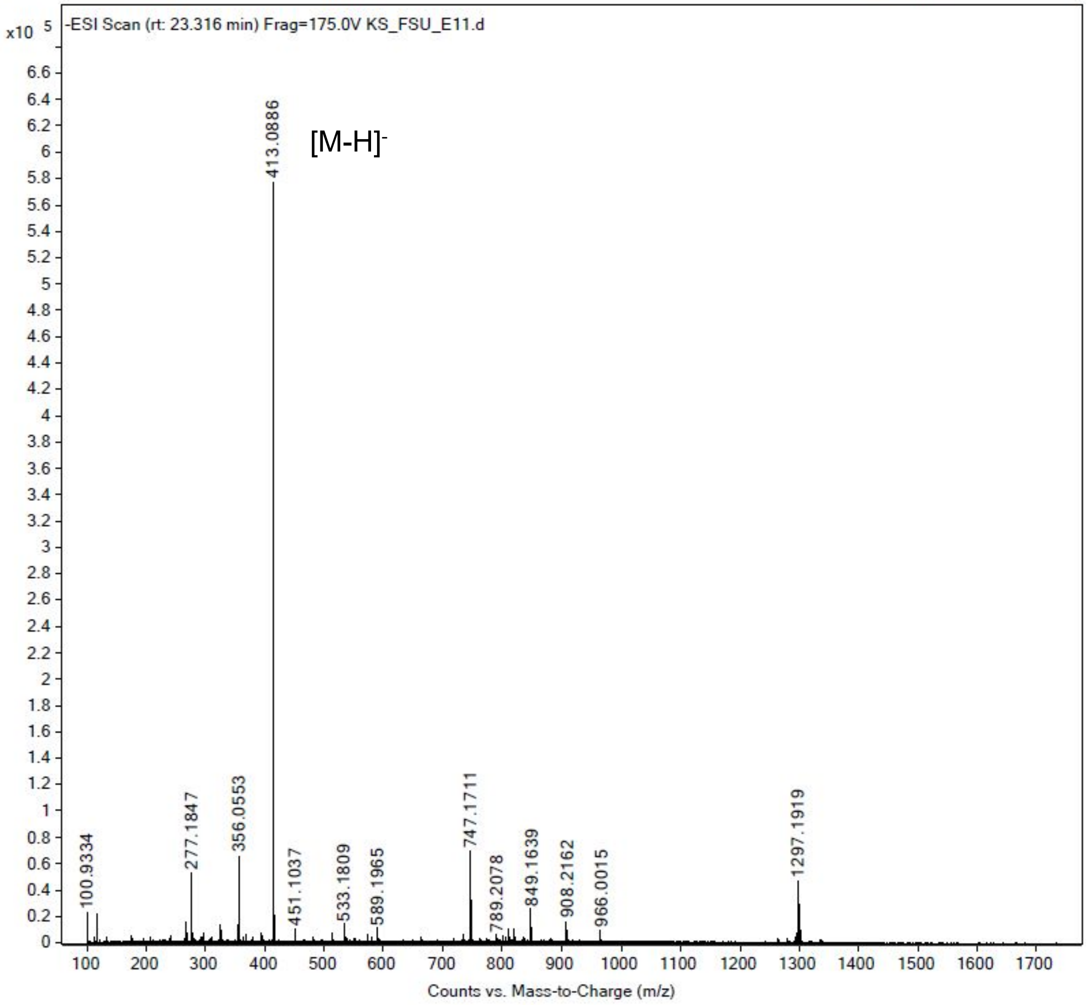

Figure S71. (-)-HRESI-MS spectrum of 11-hydroxy-auramycinone (**17**).

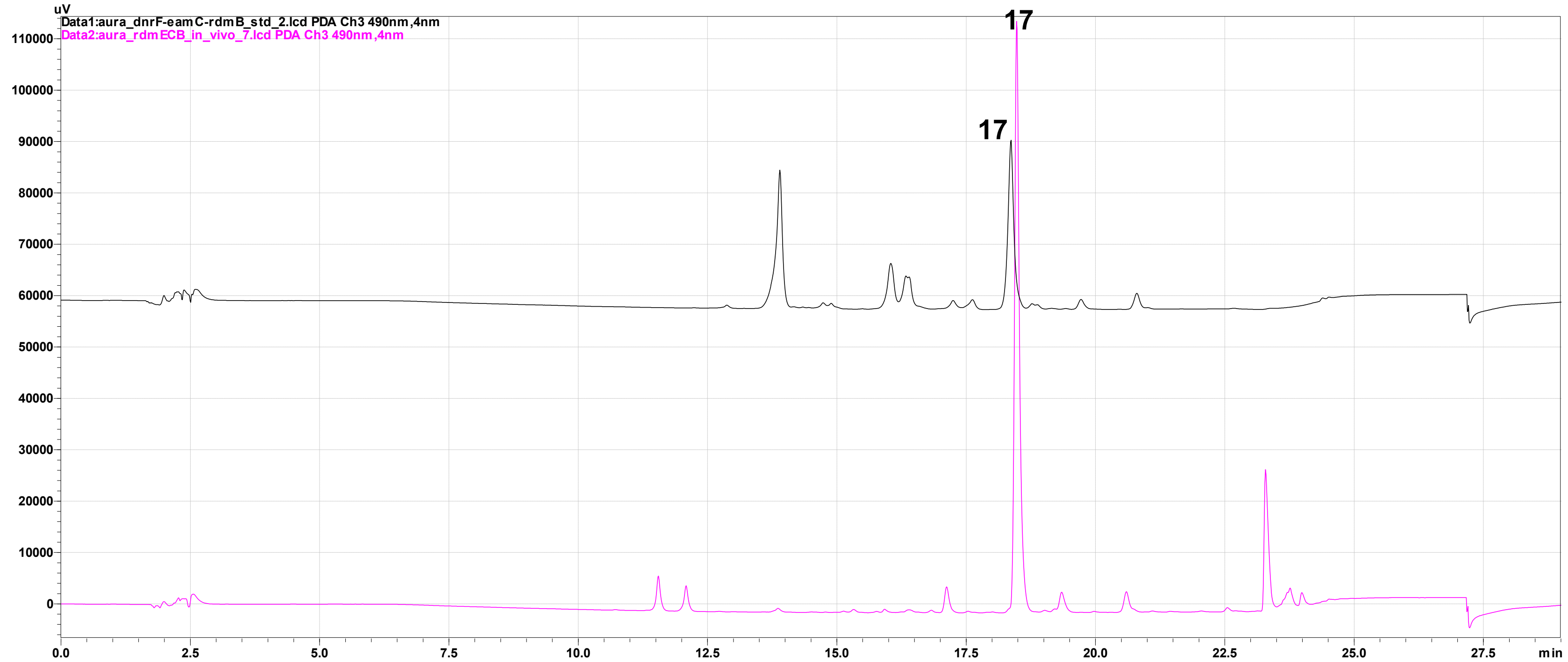

Figure S72. Alignment of 11-hydroxy-auramycinone (**17**) produced both *in vivo* and *in vitro.*

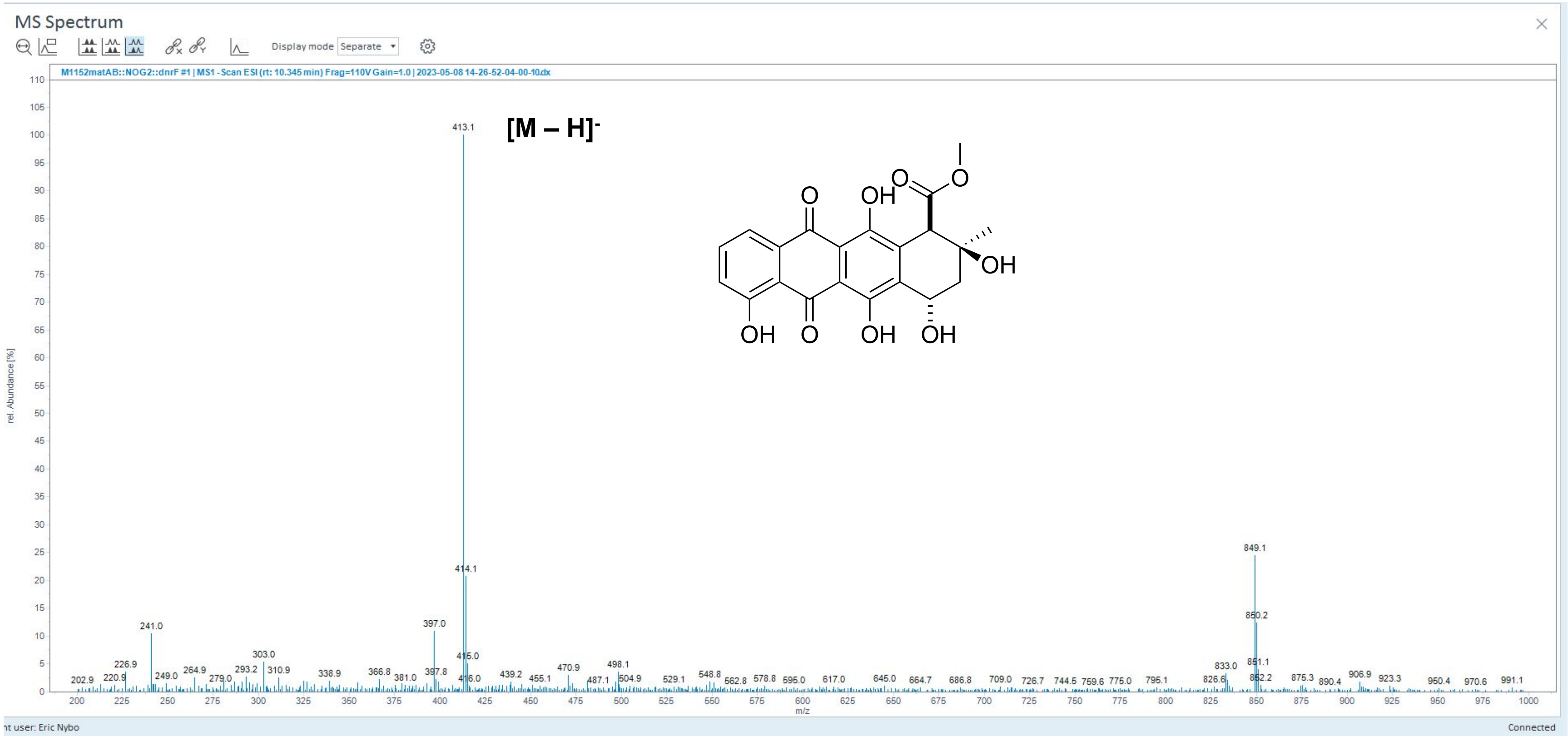

Figure S73. Mass spectrum of 11-hydroxy-nogalamycinone (**18**) identified from strain co-expressing the nogalamycinone pathway and *dnrF*.

**Sample Name**	KS_FSU_E16	**Position**	P1-C6	**Instrument Name**	Instrument 1
**User Name**		**Inj Vol**	5	**InjPosition**	
**Sample Type**	Sample	**IRM Calibration Status**	Success	**Data Filename**	KS_FSU_E16.d
**ACQ Method**	Zheng_AQC ACC short_Neg.m	**Comment**		**Acquired Time**	11/13/2023 11:57:16 PM (UTC-05:00)

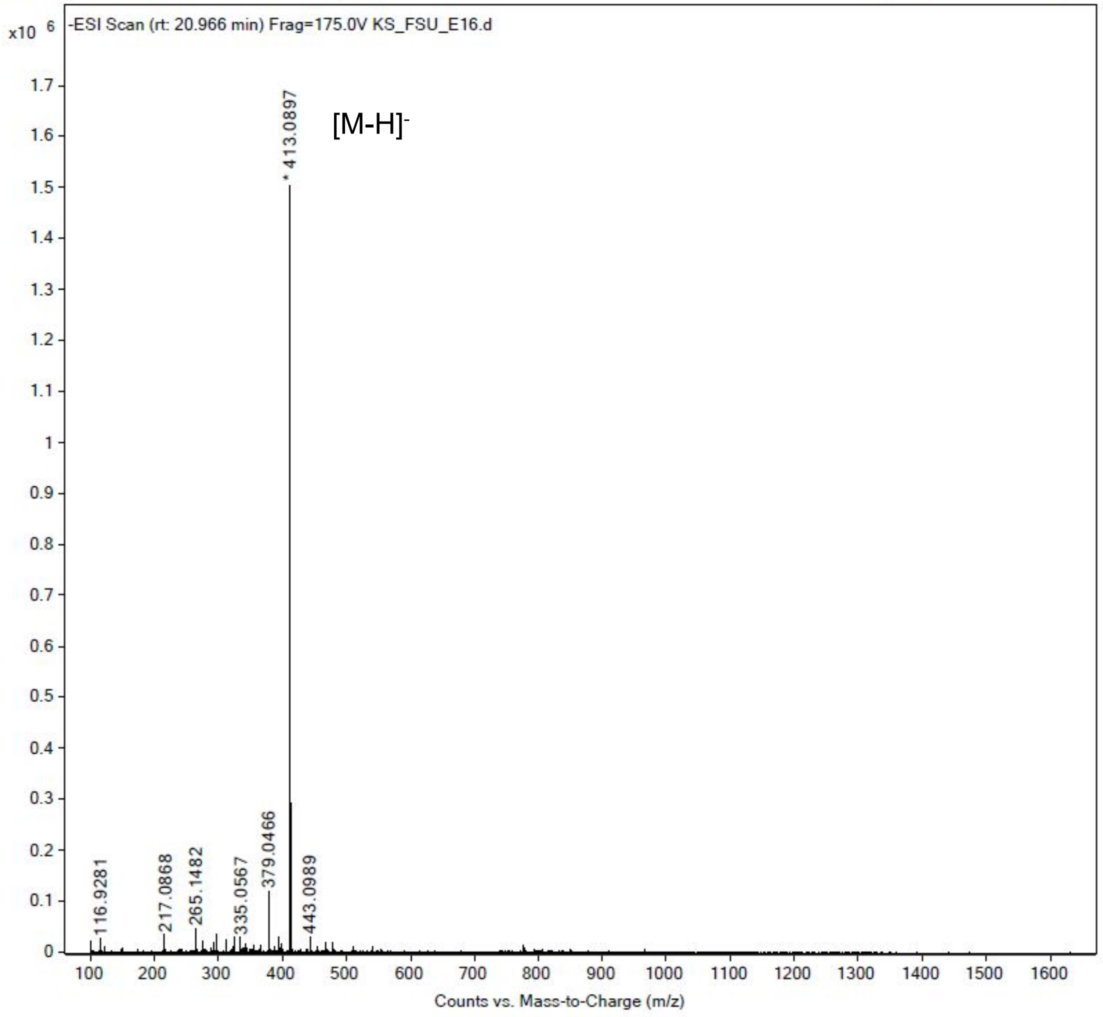

Figure S74. (-)-HRESI-MS spectrum of 11-hydroxy-nogalamycinone (**18**).

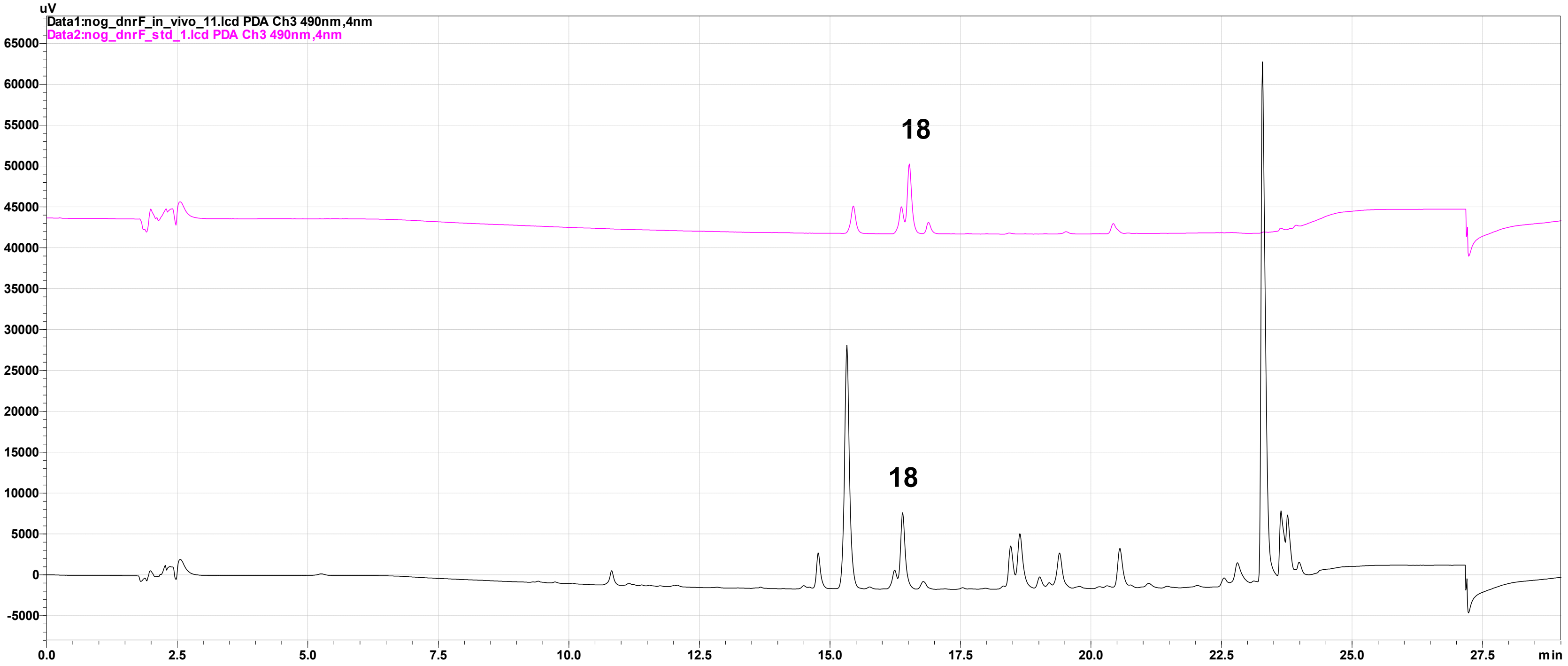

Figure S75. Alignment of 11-hydroxy-nogalamycinone (**18**) produced both *in vivo* and *in vitro*.

Print of window 80: MS Spectrum

MS Spectrum

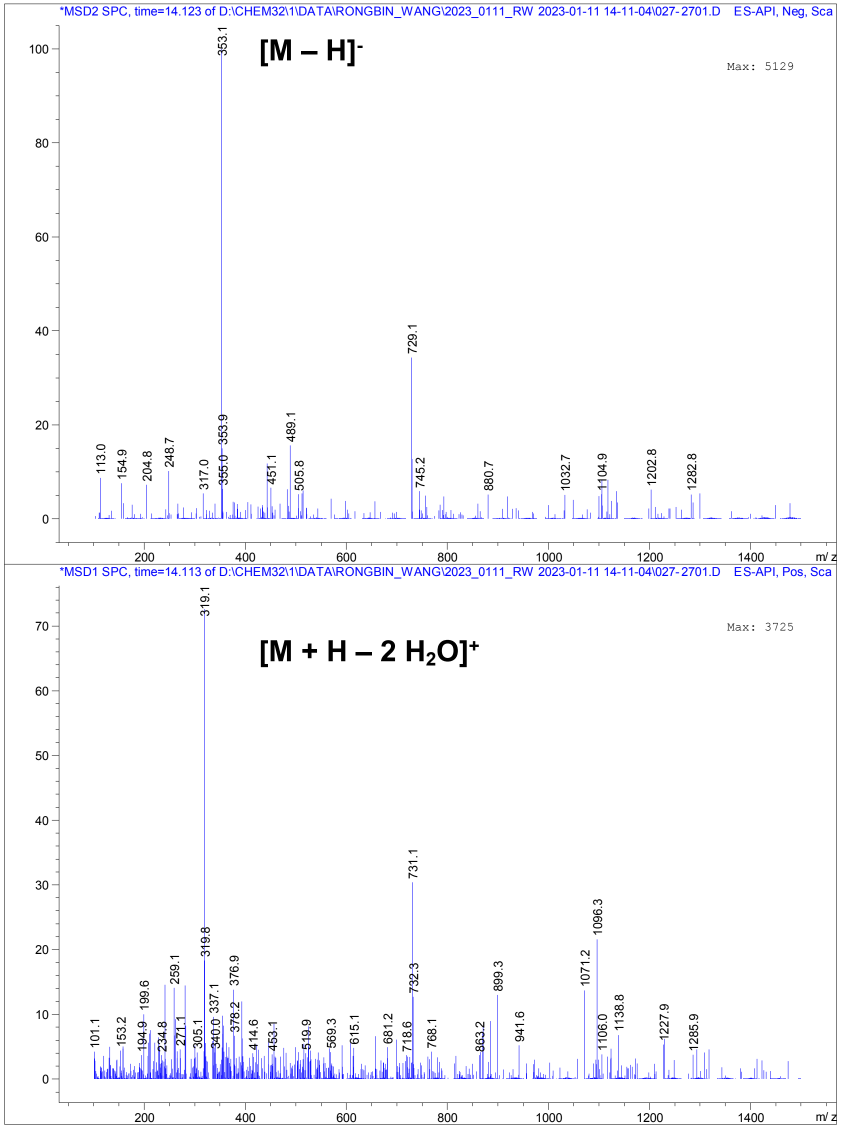

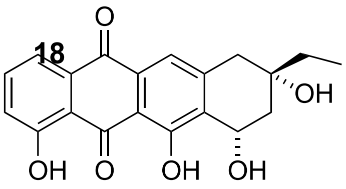

18

Agilent_LCMS 1/15/2023 11:45:01 PM SYSTEM Page 1 of 1

Figure S76. Mass spectrum of 10-decarboxy-aklavinone (**19**) produced in in vitro assay with purified aklavinone and EamC+K.

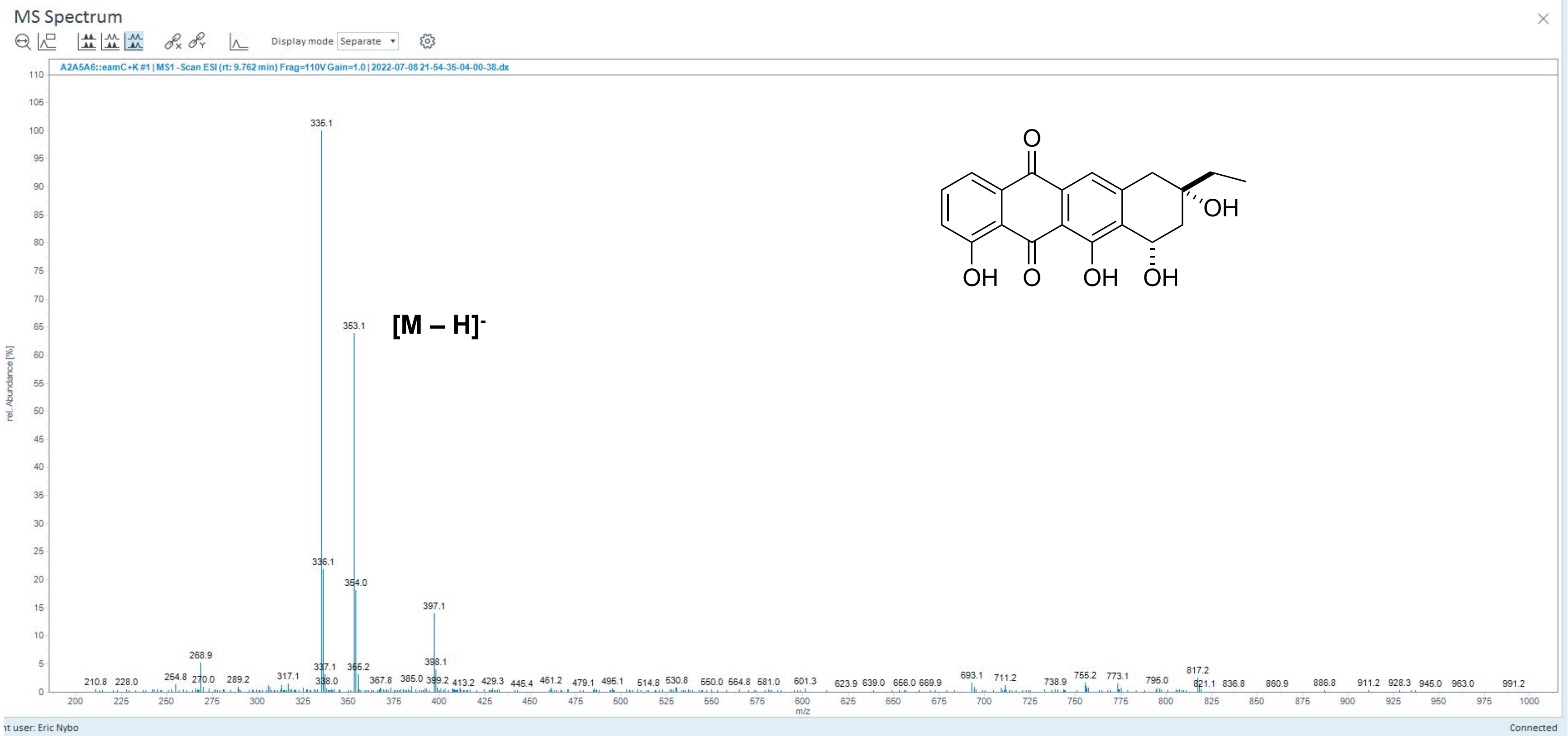

Figure S77. Mass spectrum of 10-decarboxy aklavinone (**19**) identified from strain co-expressing the aklavinone pathway and *eamC+K*.

**Sample Name**	KS_FSU_E2	**Position**	P1-B3	**Instrument Name**	Instrument 1
**User Name**		**Inj Vol**	5	**InjPosition**	
**Sample Type**	Sample	**IRM Calibration Status**	Success	**Data Filename**	KS_FSU_E2.d
**ACQ Method**	Zheng_AQC ACC short_Neg.m	**Comment**		**Acquired Time**	11/13/2023 5:11:08 PM (UTC-05:00)

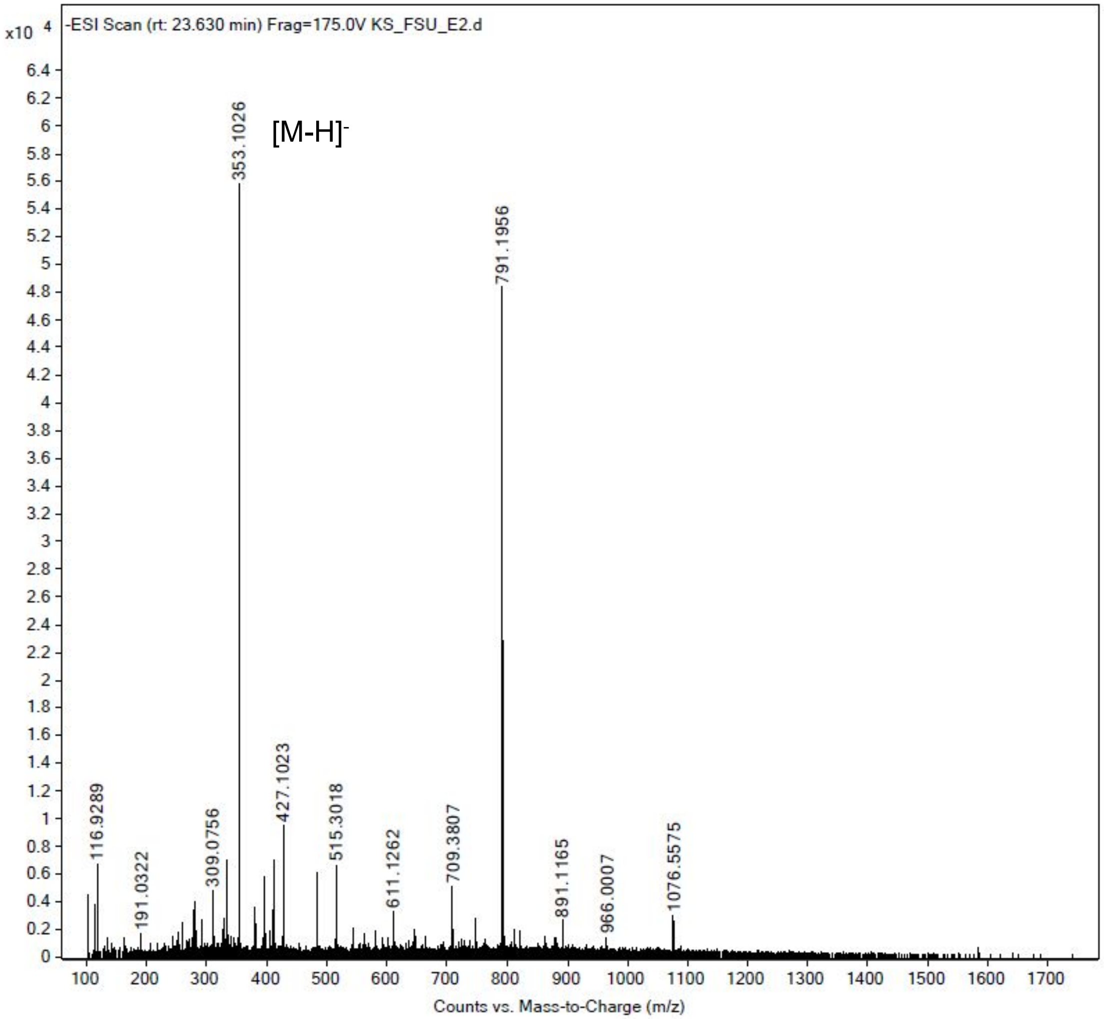

Figure S78. (-)-HRESI-MS spectrum of 10-decarboxy-aklavinone (**19**).

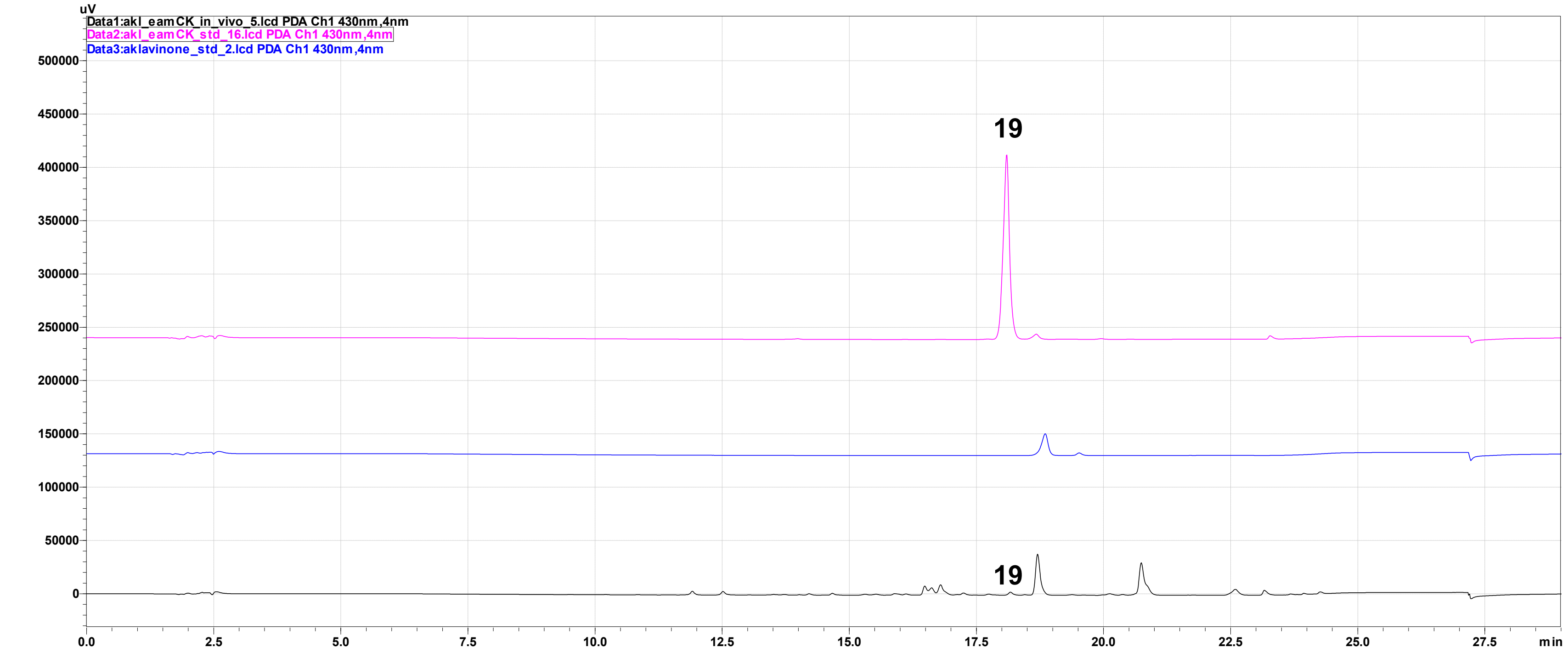

Figure S79. Alignment of 10-decarboxy-aklavinone (**19**) produced both *in vitro* and *in vivo*.

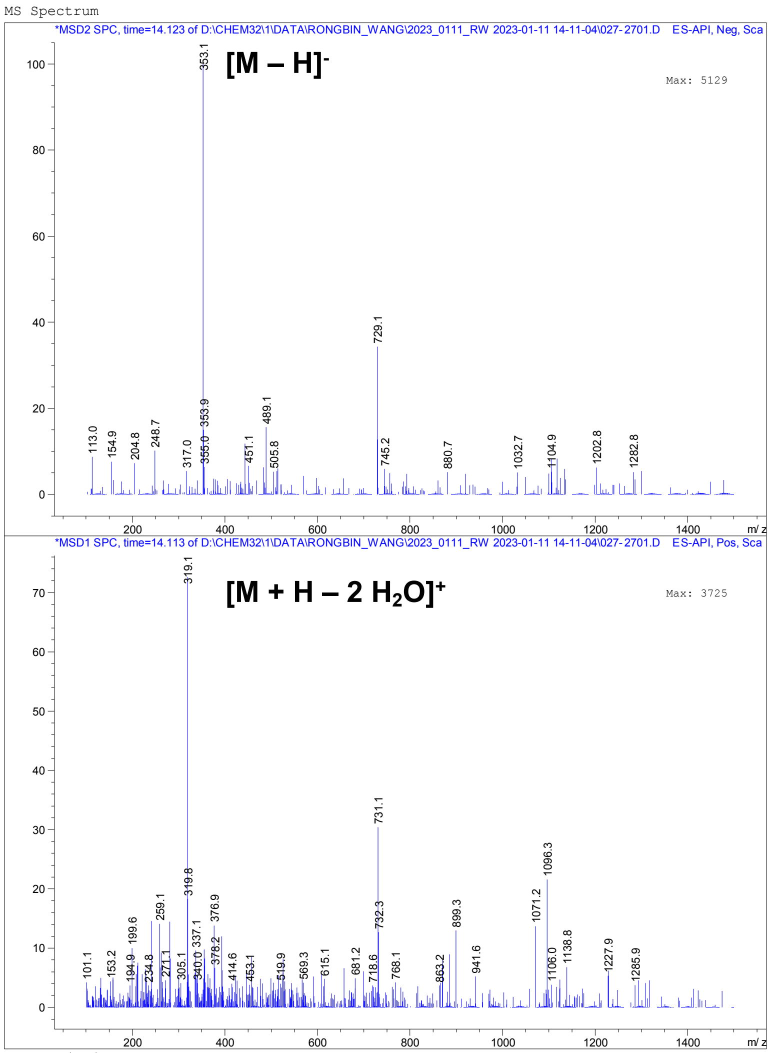

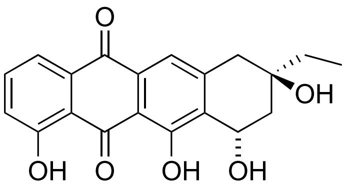

Figure S80. Mass spectrum of 10-decarboxy-9-*epi*-aklavinone (**20**) produced in *in vitro* assay with purified 9-*epi*-aklavinone and EamC+K.

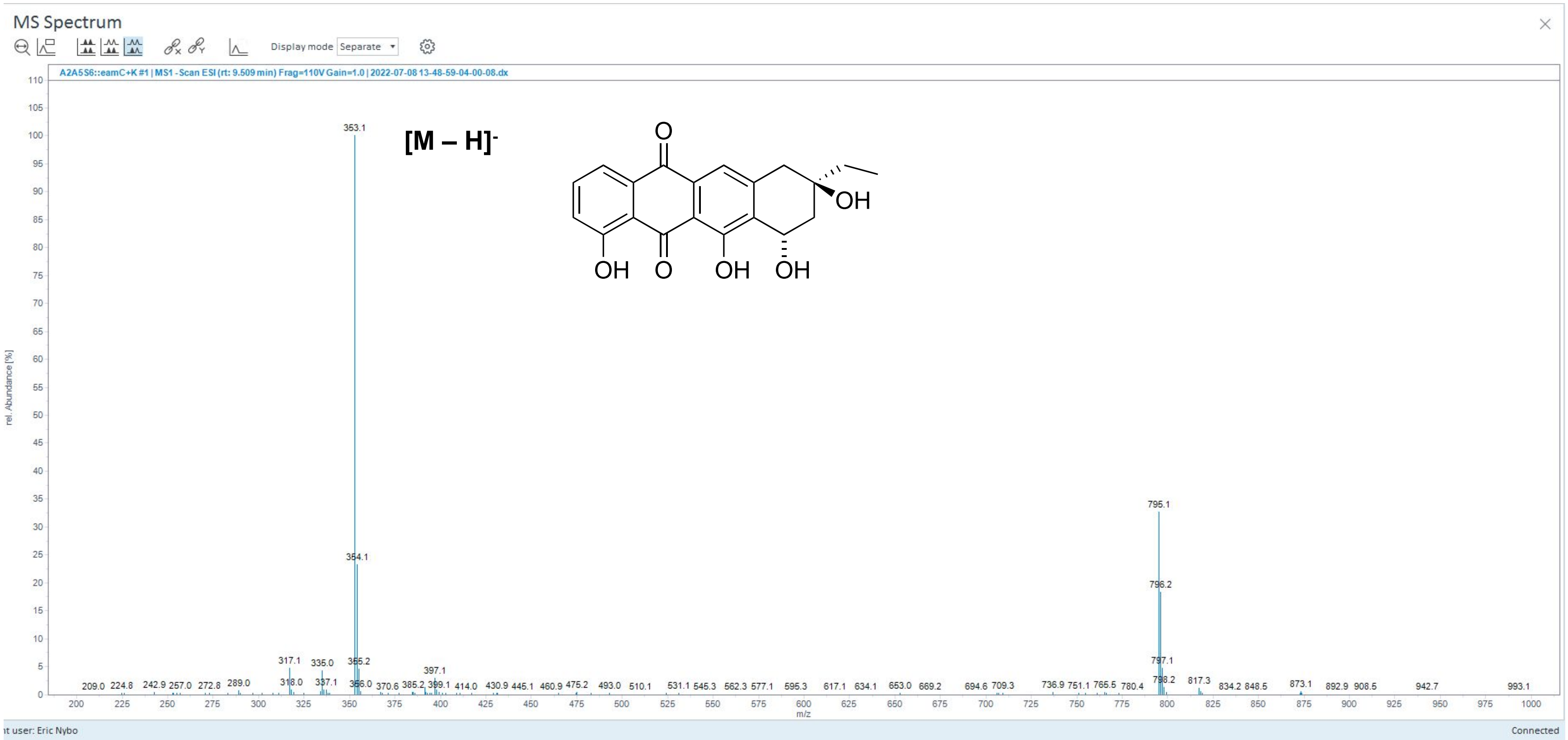

Figure S81. Mass spectrum of 10-decarboxy-9-*epi*-aklavinone (**20**) identified from strain co-expressing the 9-*epi*-aklavinone pathway and *eamC+K.*

**Sample Name**	KS_FSU_E7	**Position**	P1-B8	**Instrument Name**	Instrument 1
**User Name**		**Inj Vol**	5	**InjPosition**	
**Sample Type**	Sample	**IRM Calibration Status**	Success	**Data Filename**	KS_FSU_E7.d
**ACQ Method**	Zheng_AQC ACC short_Neg.m	**Comment**		**Acquired Time**	11/13/2023 7:36:09 PM (UTC-05:

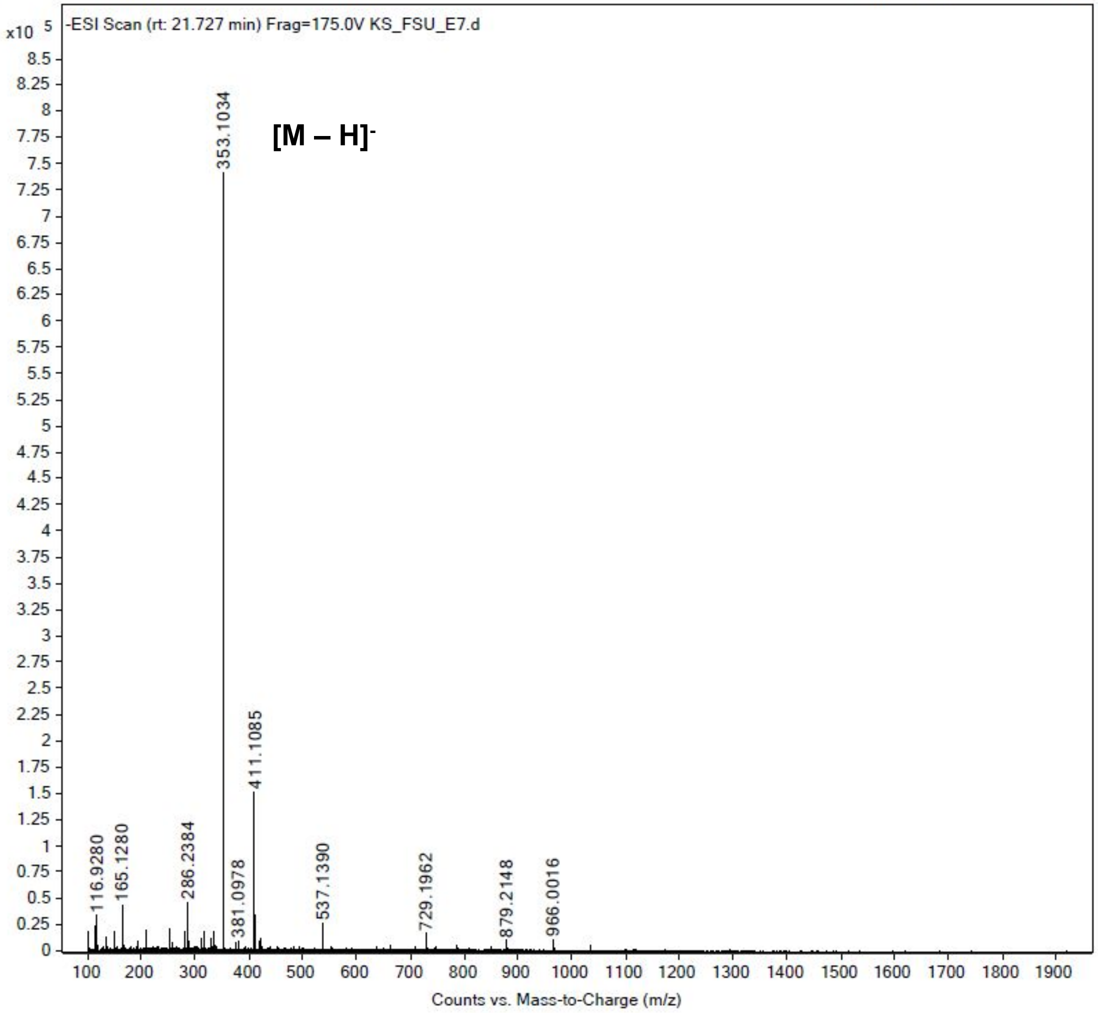

Figure S82. (-)-HRESI-MS spectrum of 10-decarboxy-9-*epi*-aklavinone (**20**).

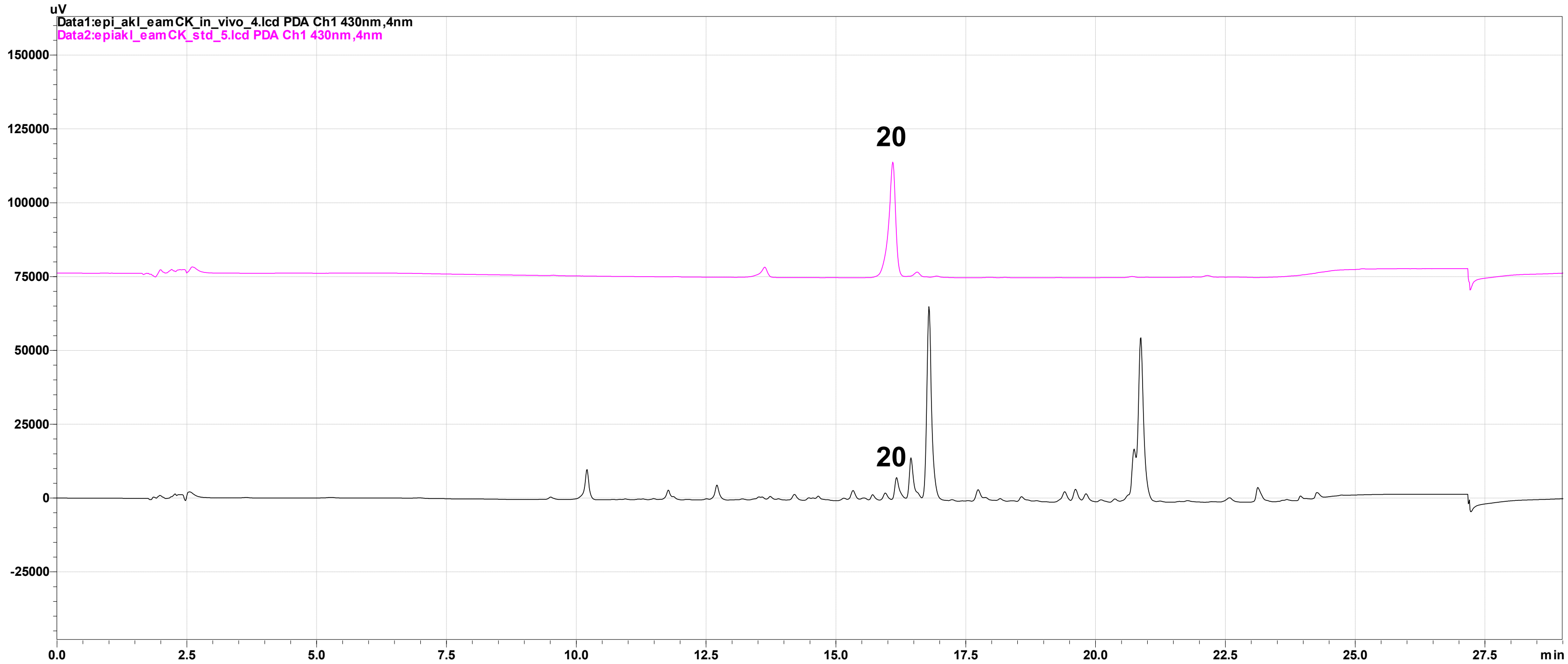

Figure S83. Alignment of 10-decarboxy-9-*epi*-aklavinone (**20**) produced both *in vitro* and *in vivo*.

Print of window 80: MS Spectrum

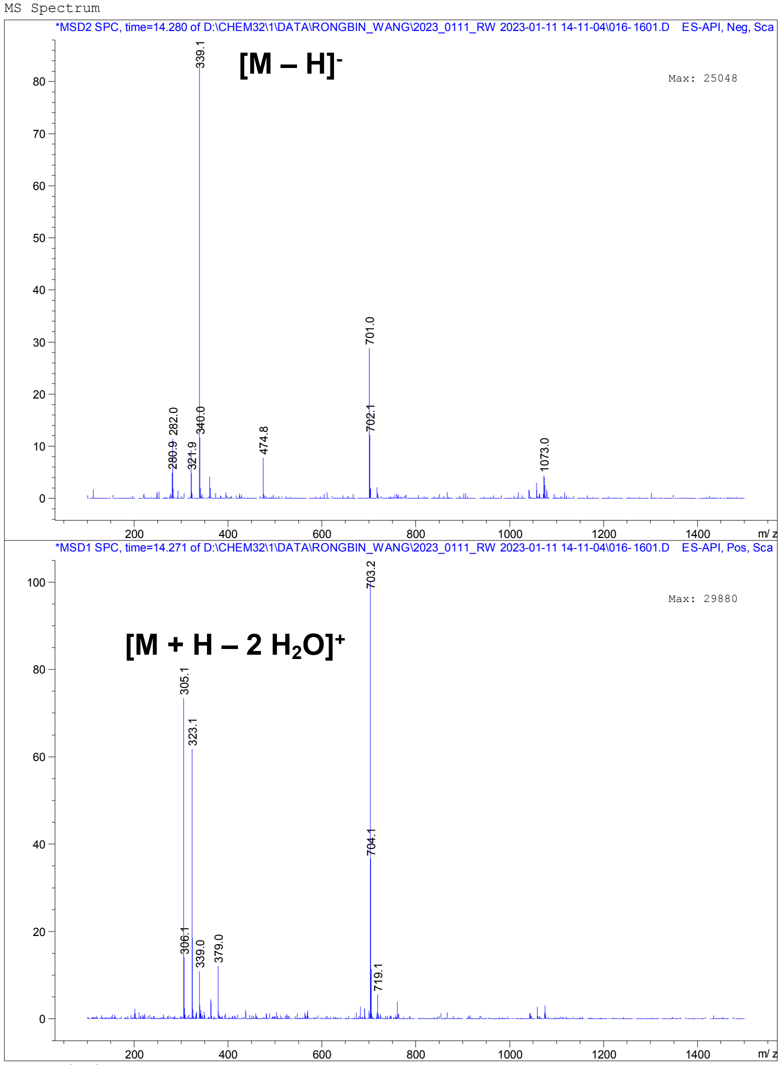

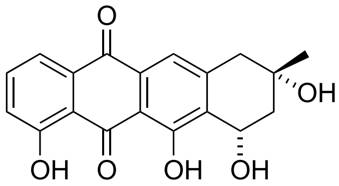

Agilent_LCMS 1/13/2023 3:33:50 PM SYSTEM Page 1 of 1

Figure S84. Mass spectrum of 10-decarboxy-auramycinone (**21**) produced in *in vitro* assay with purified auramycinone and EamC+K.

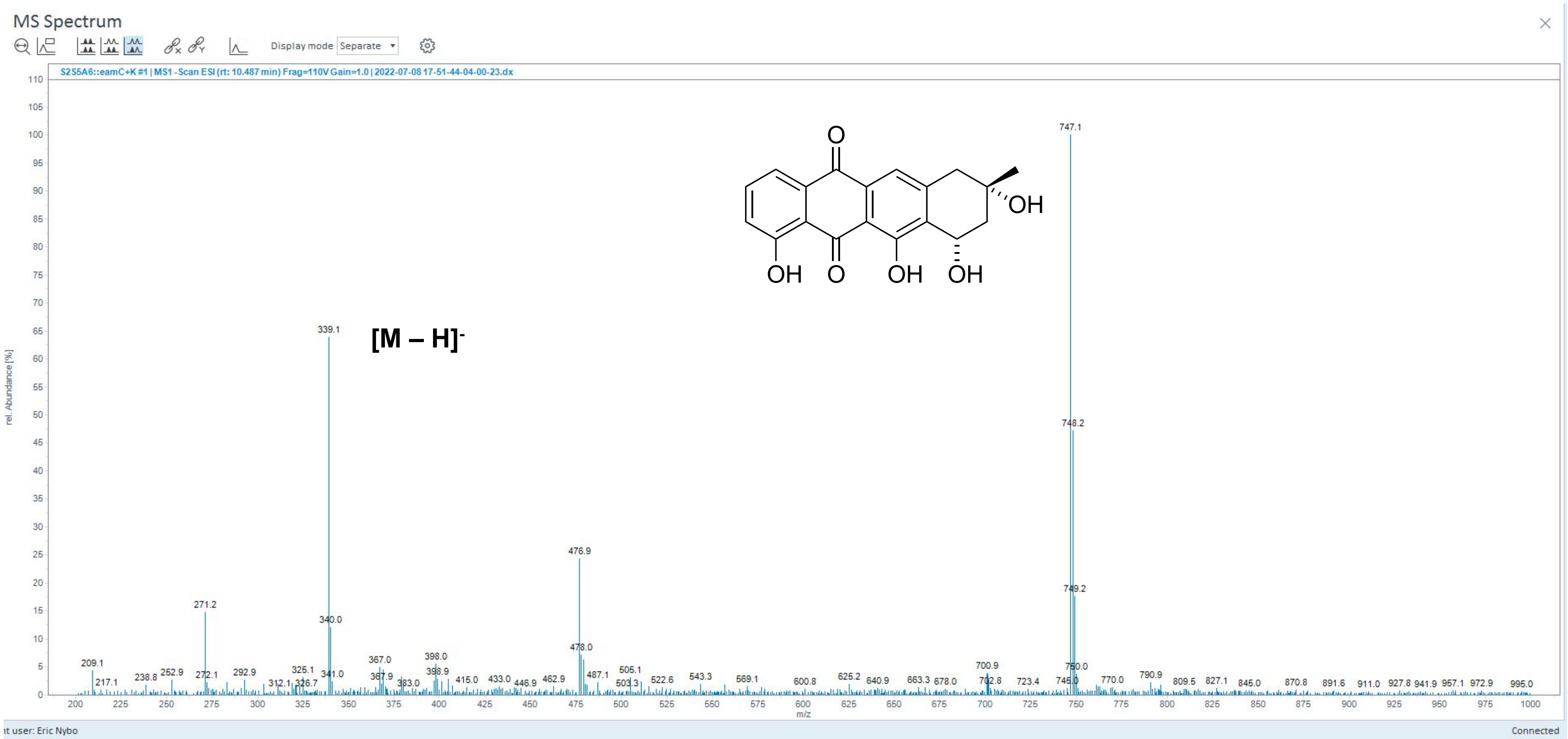

Figure S85. Mass spectrum of 10-decarboxy-auramycinone (**21**) identified from strain co-expressing the auramycinone pathway and *eamC+K.*

**Sample Name**	KS_FSU_E11	**Position**	P1-C1	**Instrument Name**	Instrument 1
**User Name**		**Inj Vol**	5	**InjPosition**	
**Sample Type**	Sample	**IRM Calibration Status**	Success	**Data Filename**	KS_FSU_E11.d
**ACQ Method**	Zheng_AQC ACC short_Neg.m	**Comment**		**Acquired Time**	11/13/2023 9:32:12 PM (UTC-05:00)

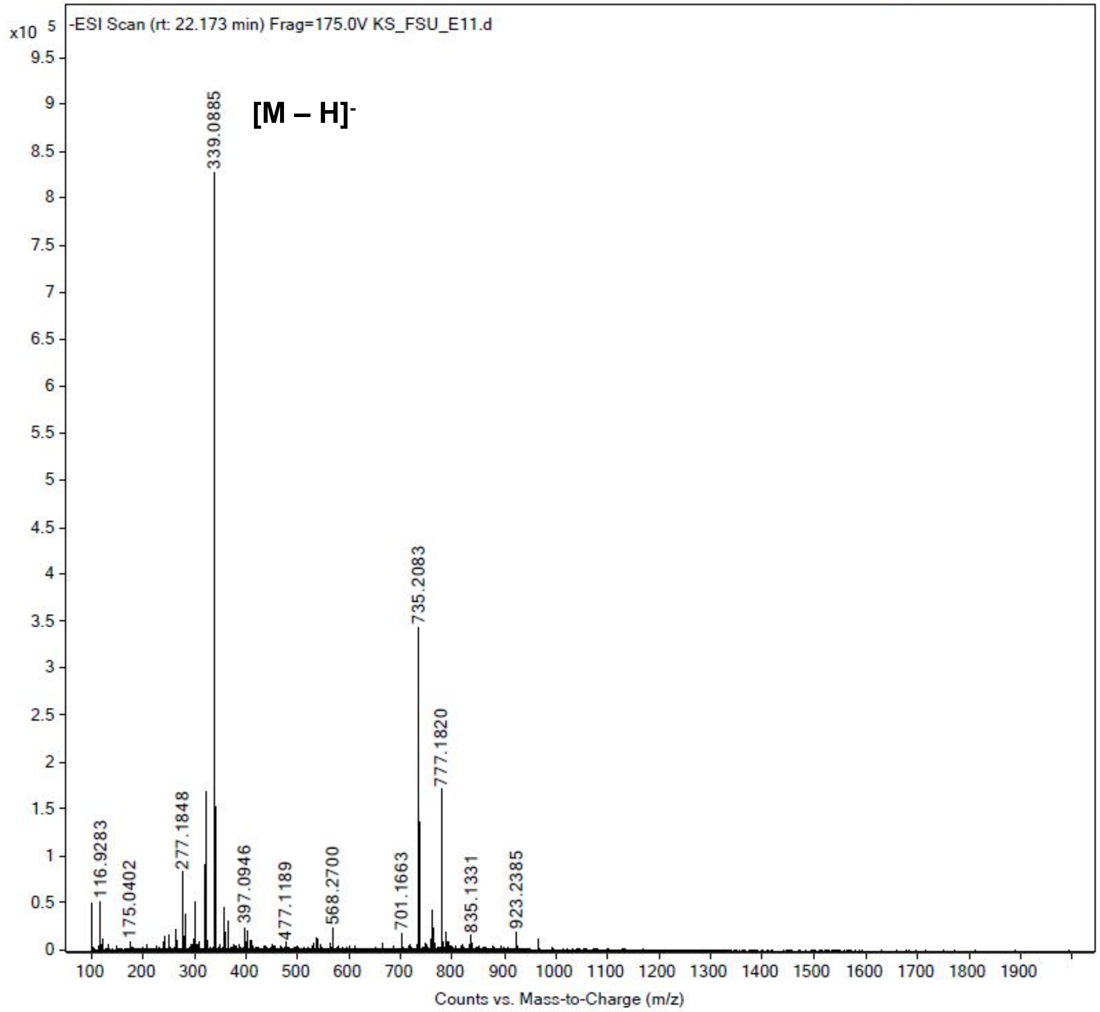

Figure S86. (-)-HRESI-MS spectrum of 10-decarboxy-auramycinone (**21**).

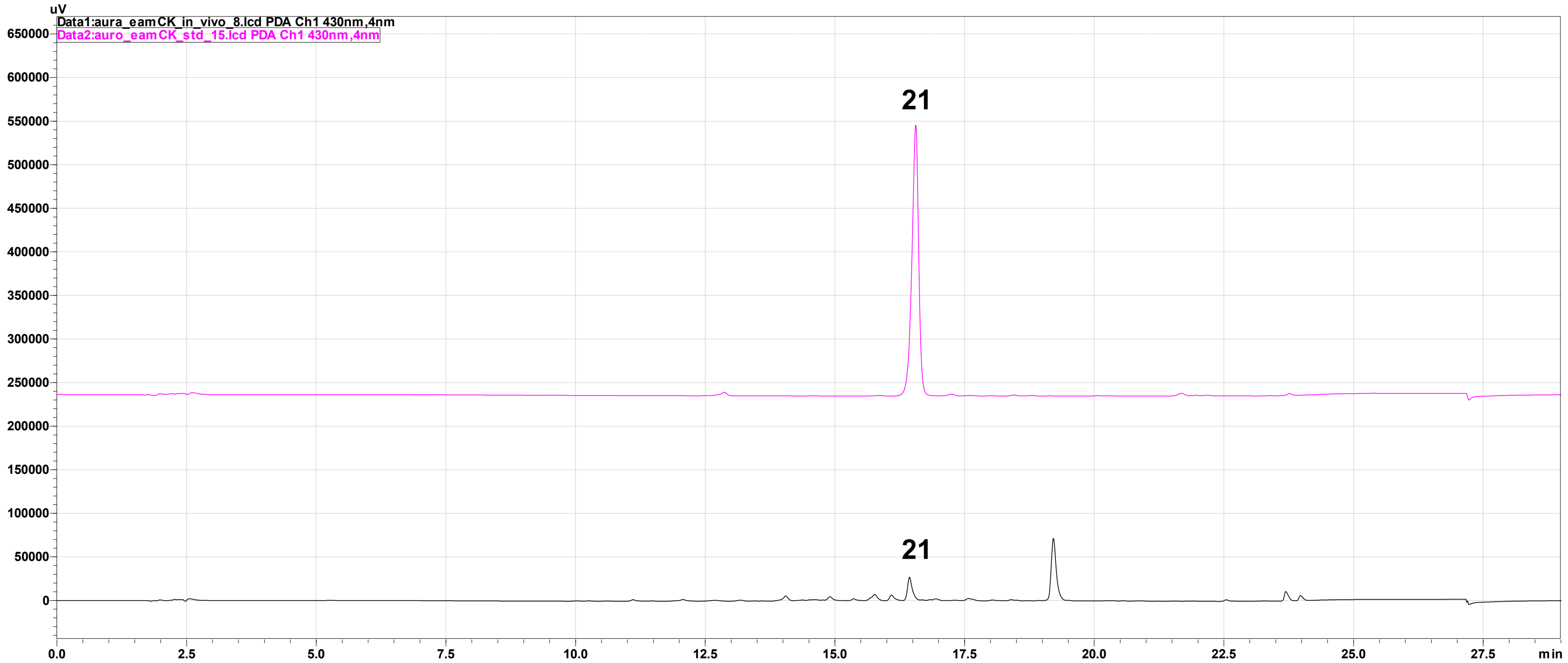

Figure S87. Alignment of 10-decarboxy-auramycinone (**21**) produced *in vitro* and *in vivo*.

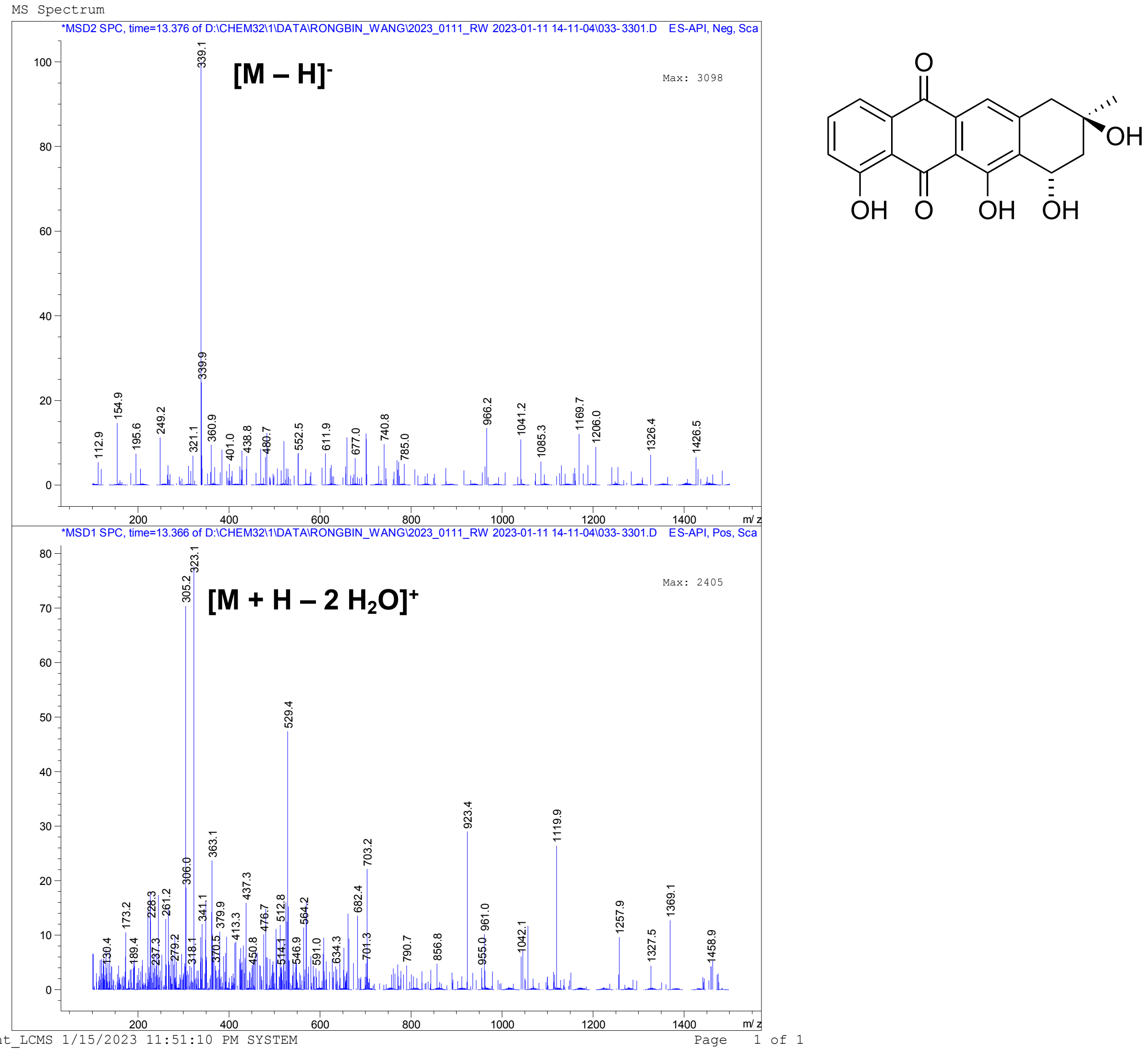

Figure S88. Mass spectrum of 10-decarboxy-nogalamycinone (**22**) produced in *in vitro* assay with purified nogalamycinone and EamC+K.

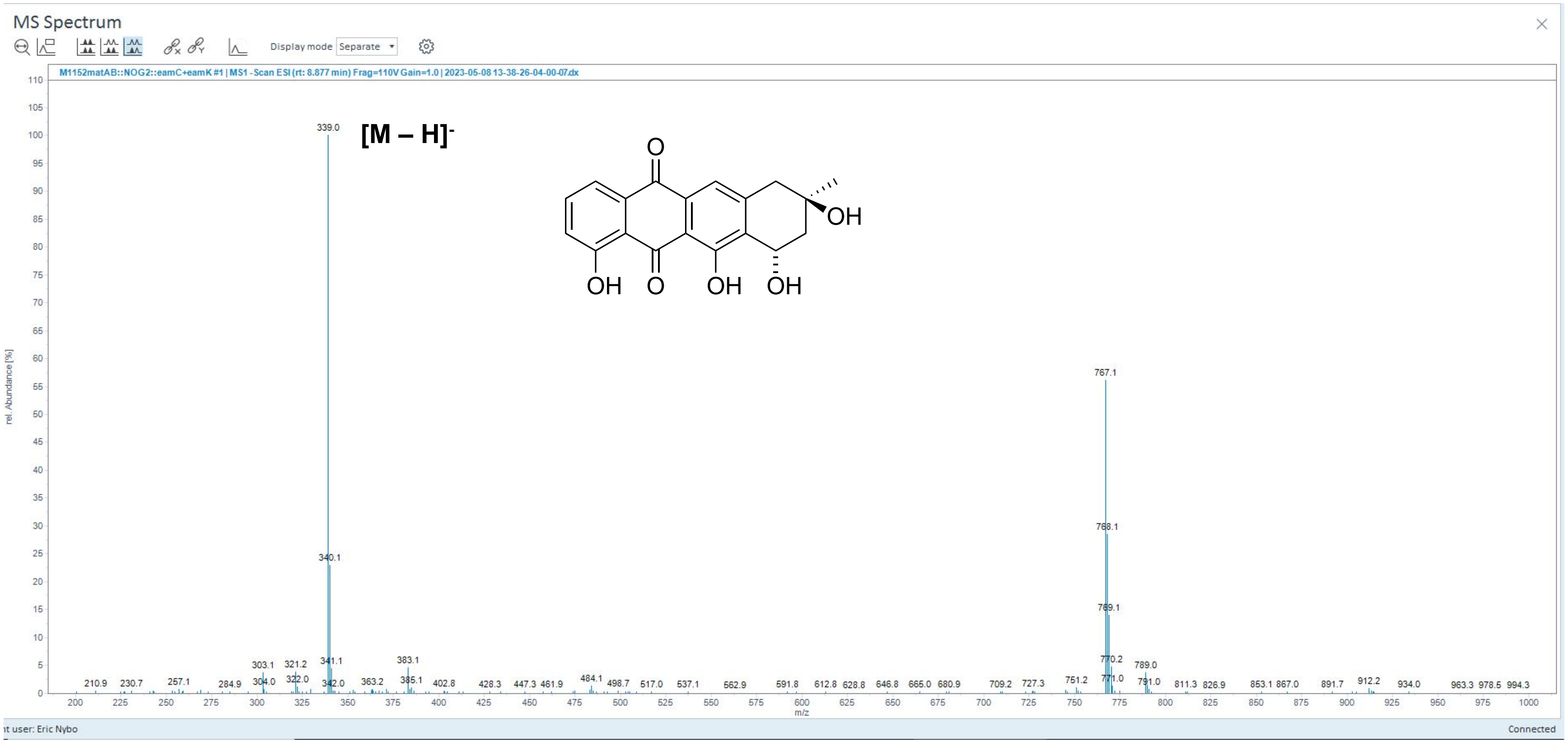

Figure S89. Mass spectrum of 10-decarboxy-nogalamycinone (**22**) identified from strain co-expressing the nogalamycinone pathway and *eamC+K*.

**Sample Name**	KS_FSU_E17	**Position**	P1-C7	**Instrument Name**	Instrument 1
**User Name**		**Inj Vol**	5	**InjPosition**	
**Sample Type**	Sample	**IRM Calibration Status**	Success	**Data Filename**	KS_FSU_E17.d
**ACQ Method**	Zheng_AQC ACC short_Neg.m	**Comment**		**Acquired Time**	11/14/2023 12:26:16 AM (UTC-05:00)

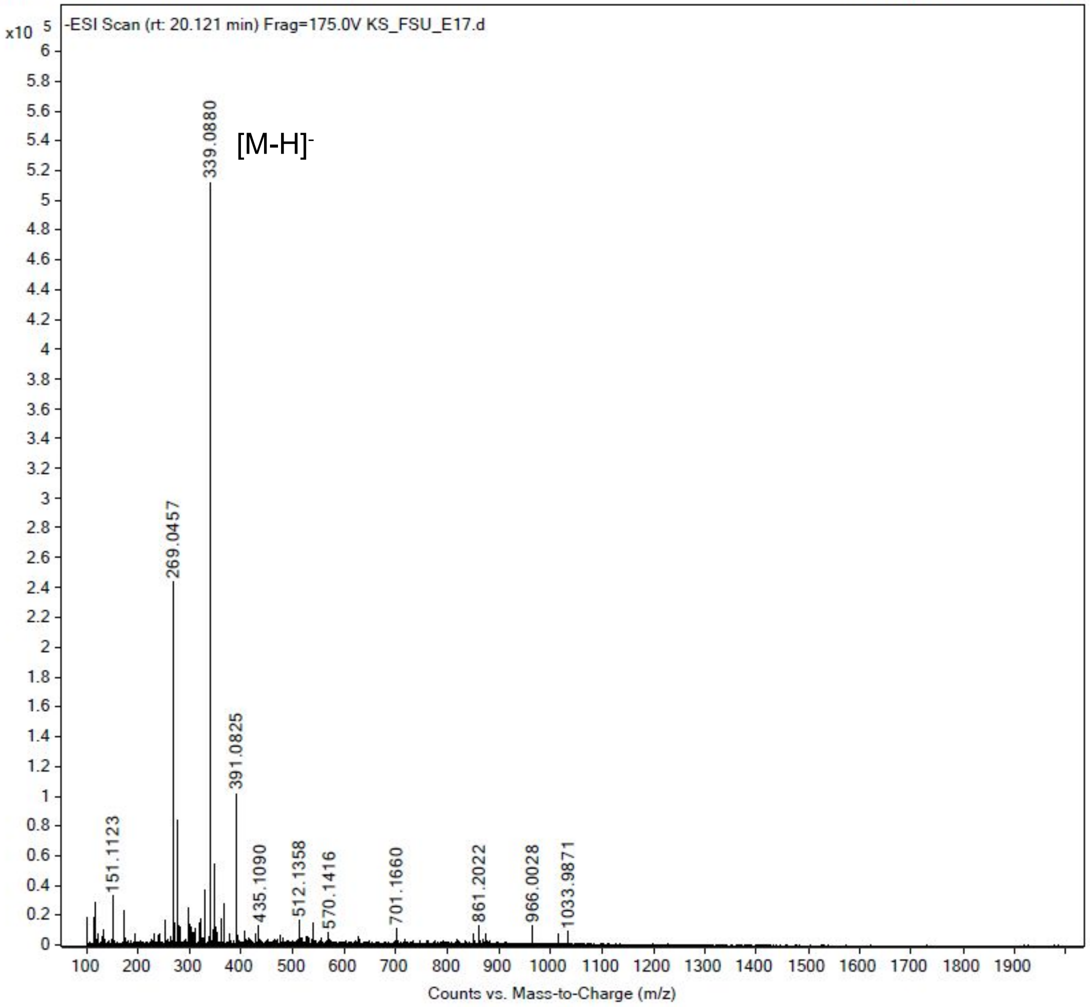

Figure S90. (-)-HRESI-MS spectrum of 10-decarboxy-nogalamycinone (**22**).

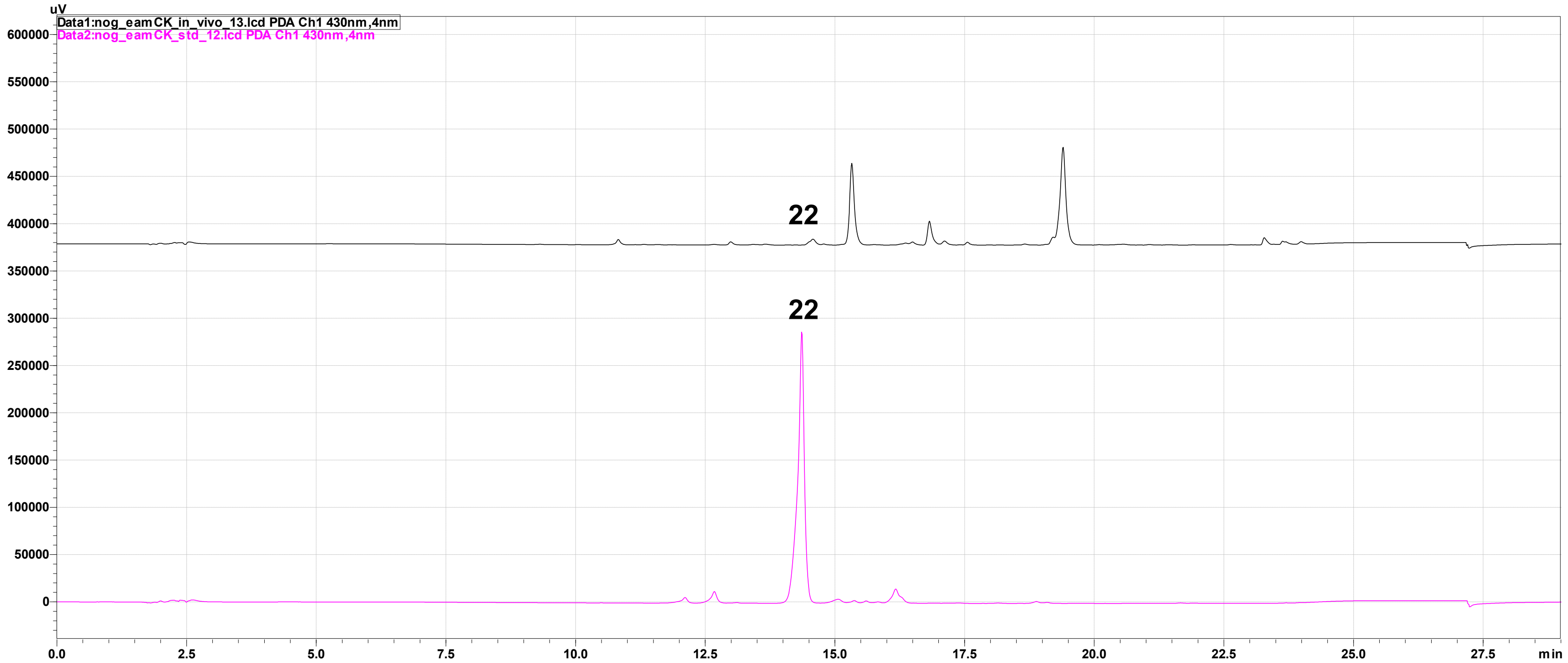

Figure S91. Alignment of 10-decarboxy nogalamycinone (**22**) produced both *in vitro* and *in vivo.*

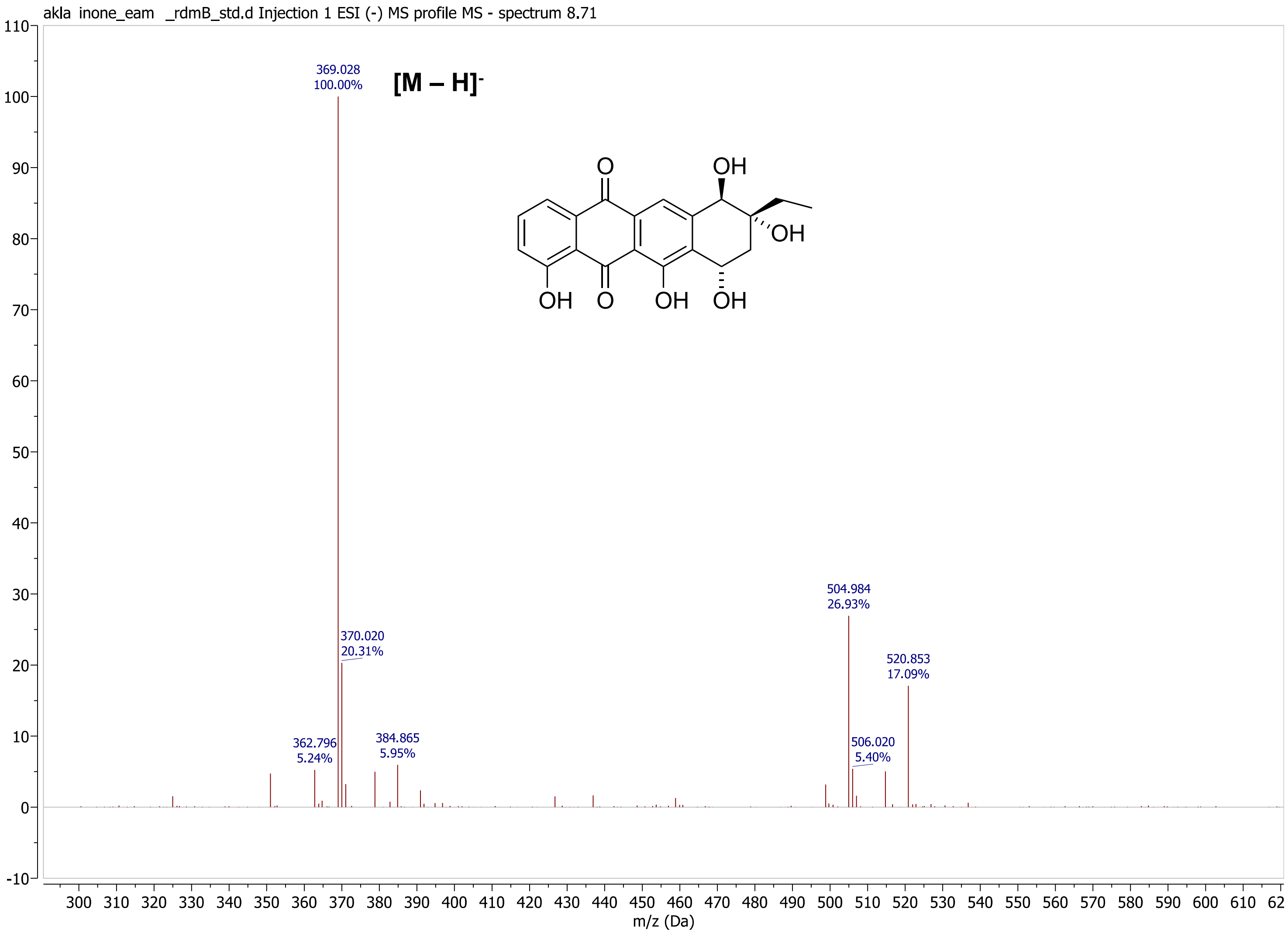

Figure S92. Mass spectrum of 10-hydroxy-aklavinone (**23**) produced in *in vitro* assay with purified aklavinone and EamC+RdmB.

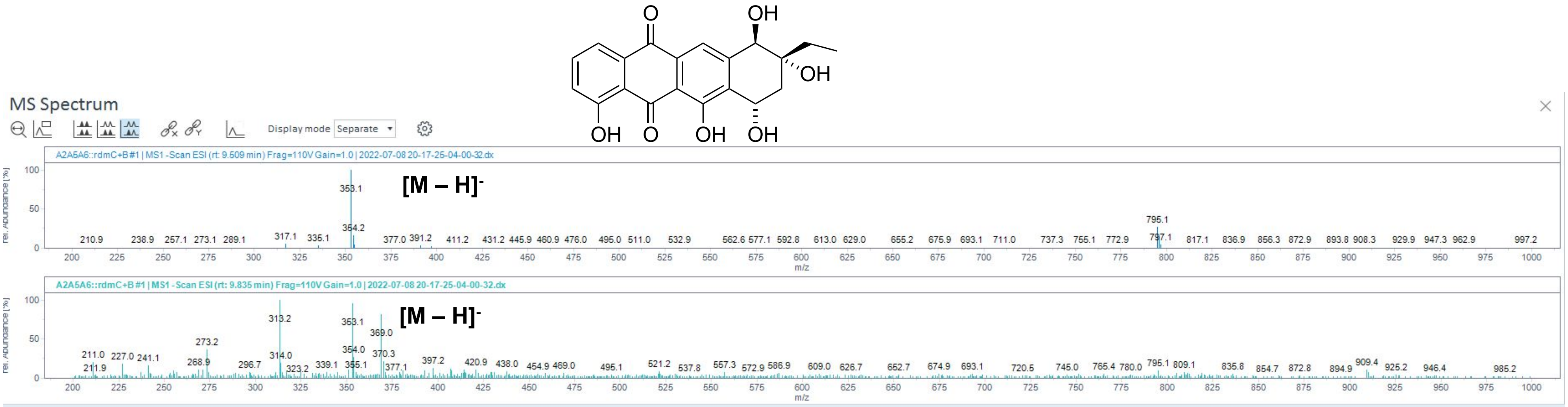

Figure S93. Mass spectrum of 10-hydroxy-aklavinone (**23**) identified from strain co-expressing the aklavinone pathway and *rdmC+B.*

**Sample Name**	KS_FSU_E2	**Position**	P1-B3	**Instrument Name**	Instrument 1
**User Name**		**Inj Vol**	5	**InjPosition**	
**Sample Type**	Sample	**IRM Calibration Status**	Success	**Data Filename**	KS_FSU_E2.d
**ACQ Method**	Zheng_AQC ACC short_Neg.m	**Comment**		**Acquired Time**	11/13/2023 5:11:08 PM (UTC-05:00)

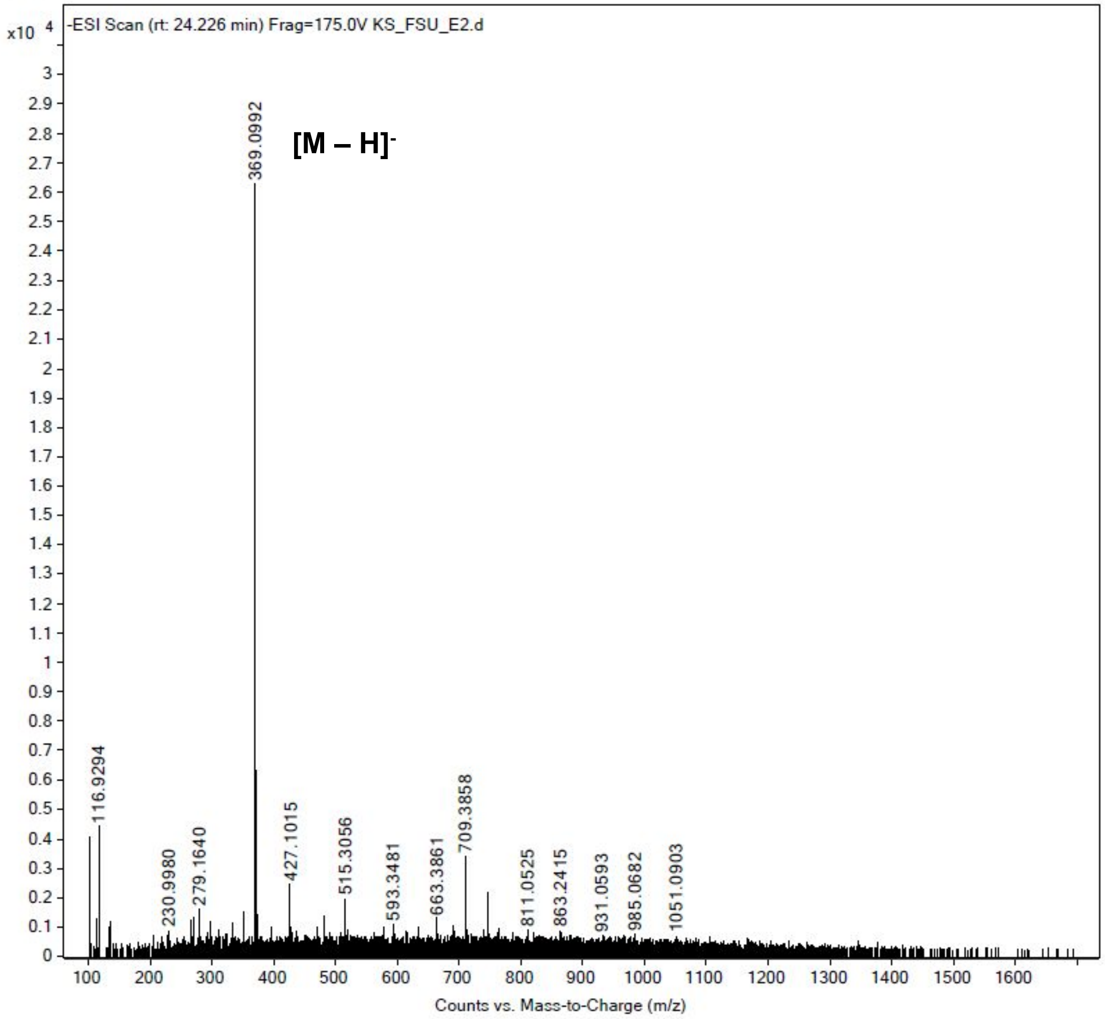

Figure S94. (-)-HRESI-MS spectrum of 10-hydroxy-aklavinone (**23**).

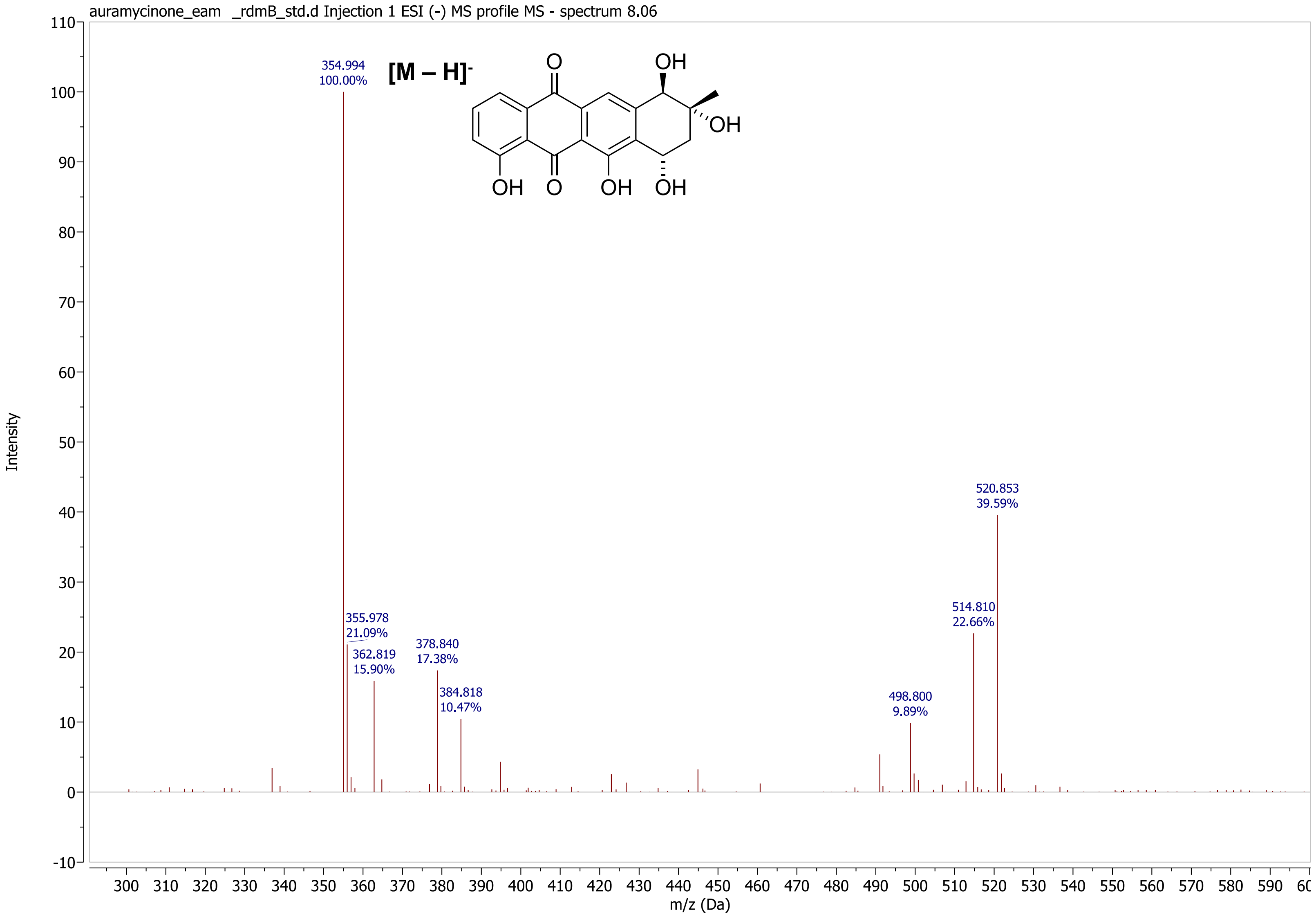

Figure S95. Mass spectrum of 10-hydroxy-auramycinone (**24**) produced in *in vitro* assay from purified auramycinone and EamC+RdmB.

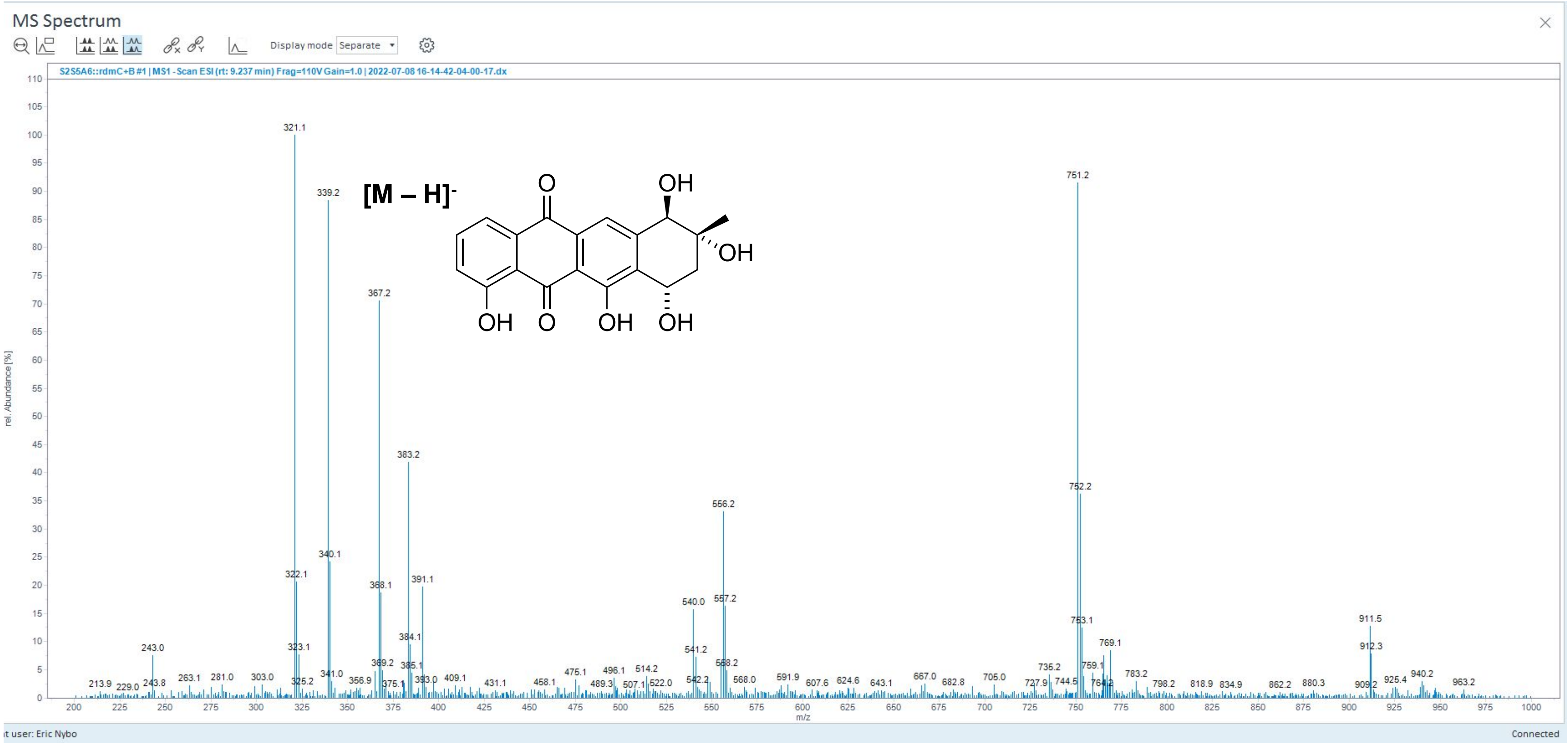

Figure S96. Mass spectrum of 10-hydroxy-auramycinone (**24**) identified from strain co-expressing the auramycinone pathway and *rdmC+B.*

**Sample Name**	KS_FSU_E11	**Position**	P1-C1	**Instrument Name**	Instrument 1
**User Name**		**Inj Vol**	5	**InjPosition**	
**Sample Type**	Sample	**IRM Calibration Status**	Success	**Data Filename**	KS_FSU_E11.d
**ACQ Method**	Zheng_AQC ACC short_Neg.m	**Comment**		**Acquired Time**	11/13/2023 9:32:12 PM (UTC-05:00)

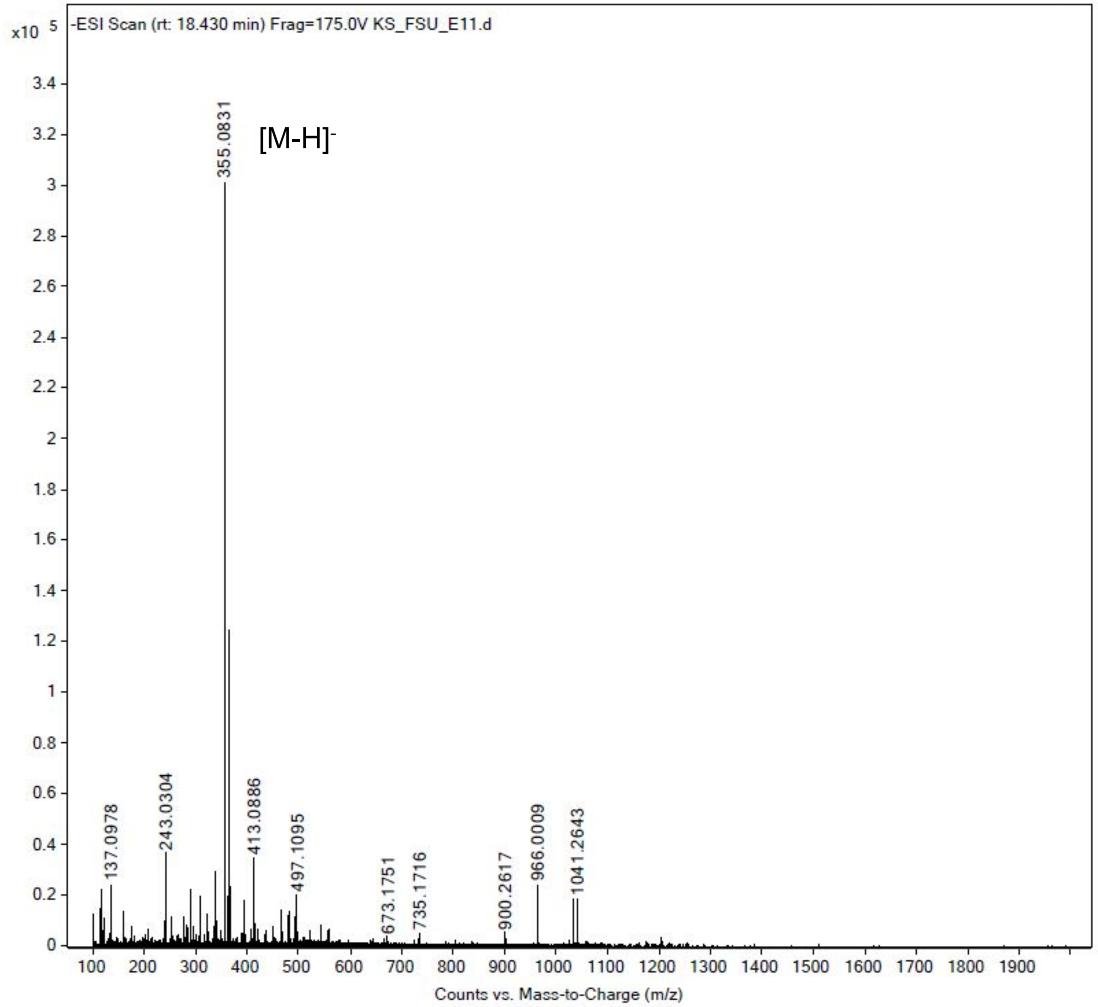

Figure S97. (-)-HRESI-MS spectrum of 10-hydroxy-auramycinone (**24**).

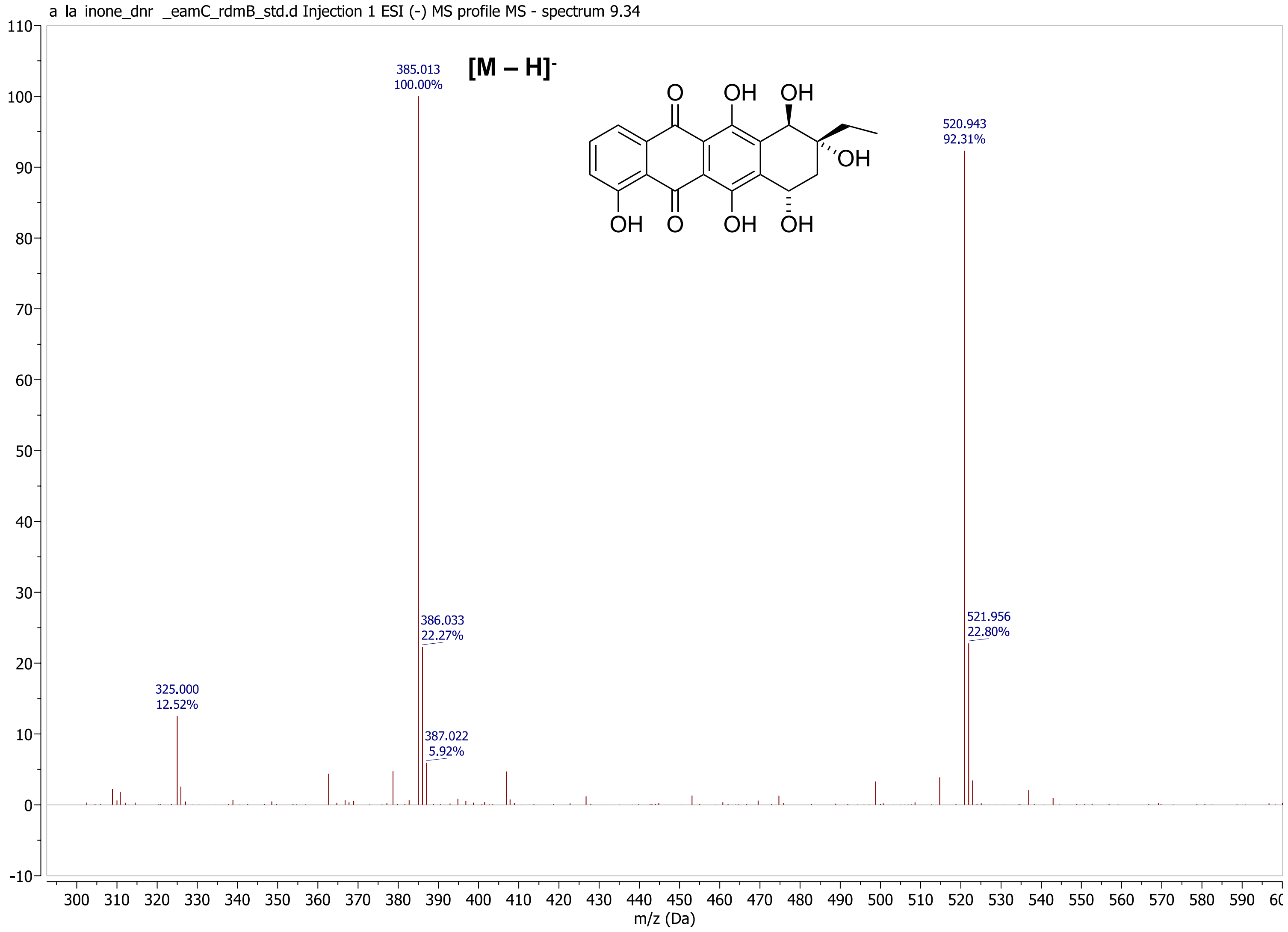

Figure S98. Mass spectrum of β-rhodomycinone (**25**) produced in *in vitro* assay with purified aklavinone and DnrF+EamC+RdmB.

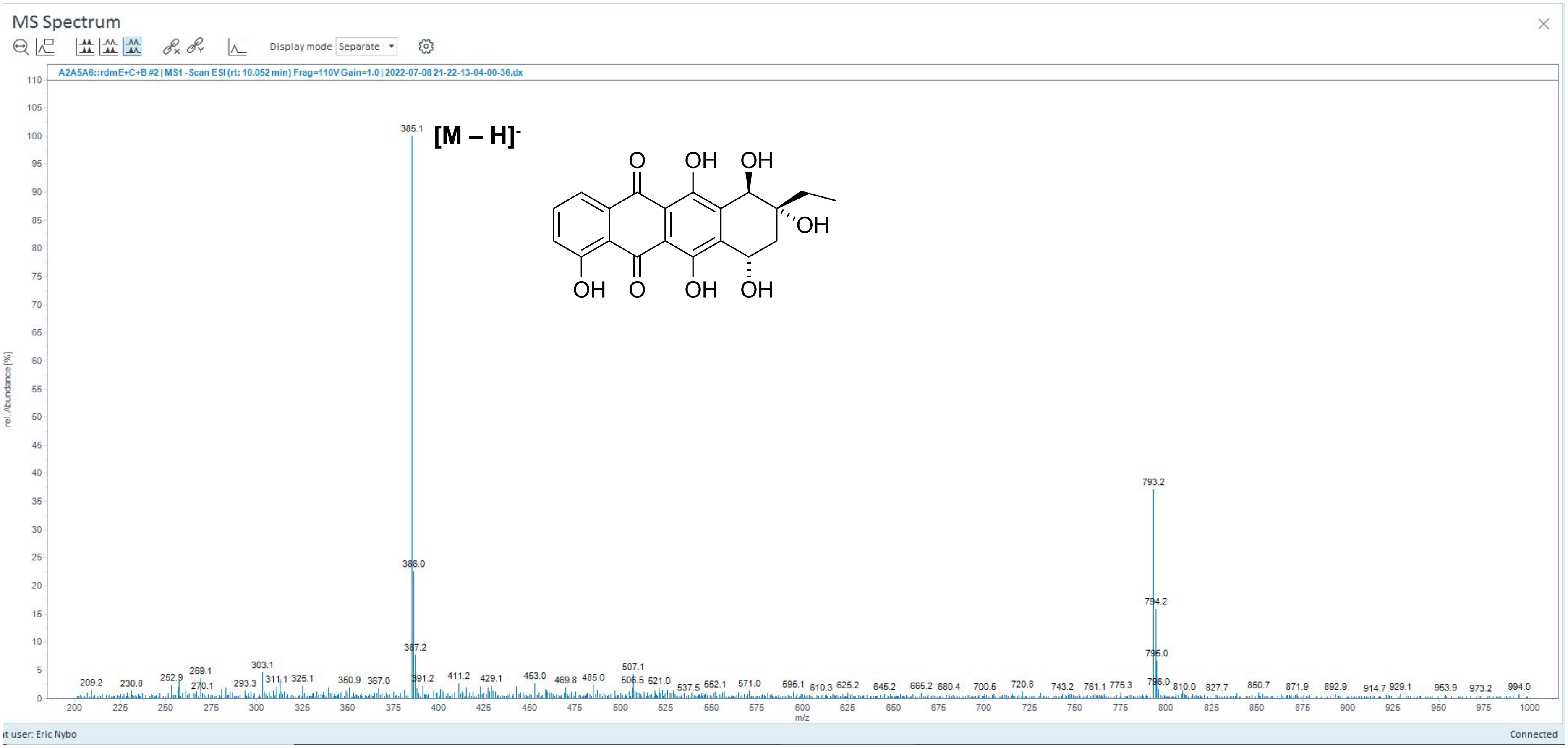

Figure S99. Mass spectrum of β-rhodomycinone (**25**) identified from strain co-expressing the aklavinone pathway and *rdmE+C+B.*

**Sample Name**	KS_FSU_E2	**Position**	P1-B3	**Instrument Name**	Instrument 1
**User Name**		**Inj Vol**	5	**InjPosition**	
**Sample Type**	Sample	**IRM Calibration Status**	Success	**Data Filename**	KS_FSU_E2.d
**ACQ Method**	Zheng_AQC ACC short_Neg.m	**Comment**		**Acquired Time**	11/13/2023 5:11:08 PM (UTC-05:00)

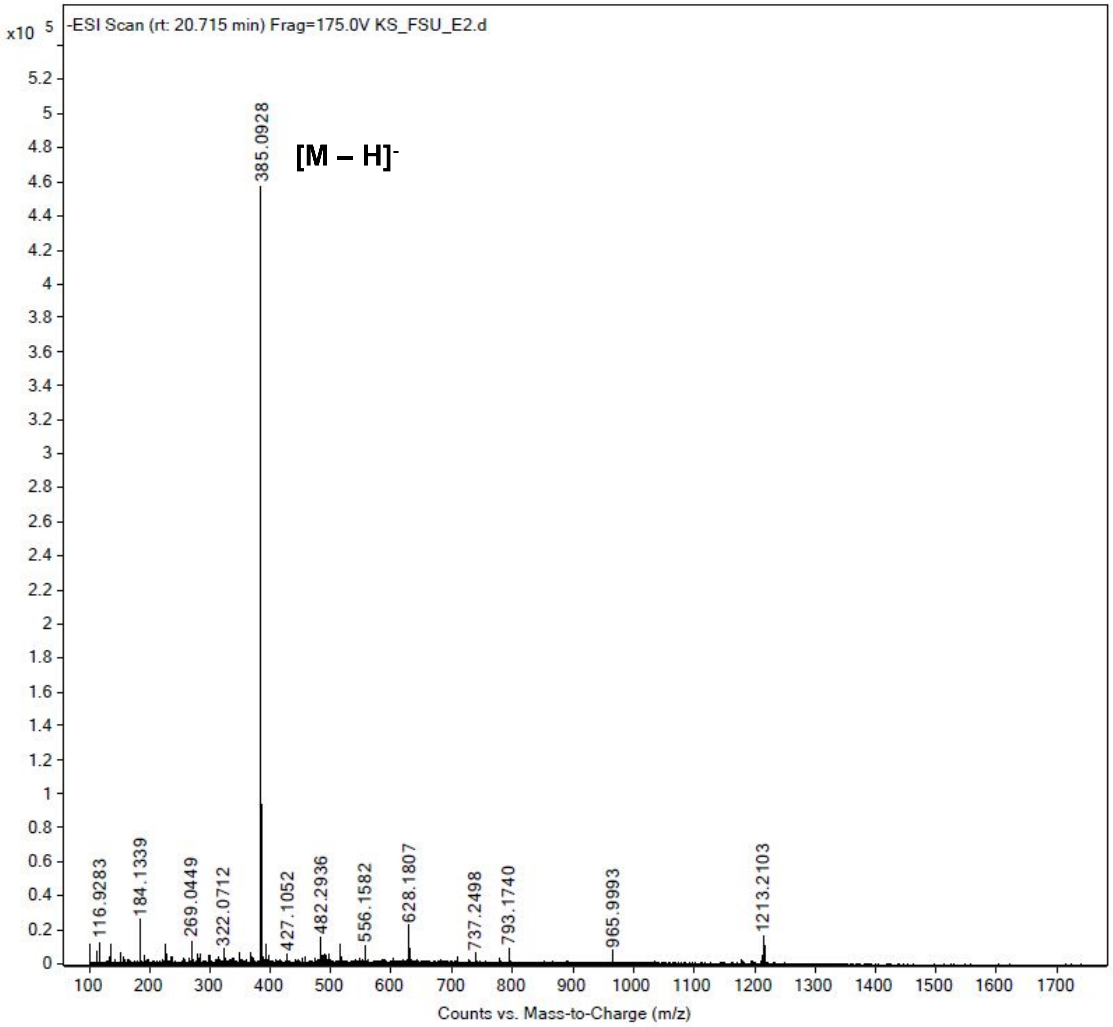

Figure S100. (-)-HRESI-MS spectrum of beta-rhodomycinone (**25**).

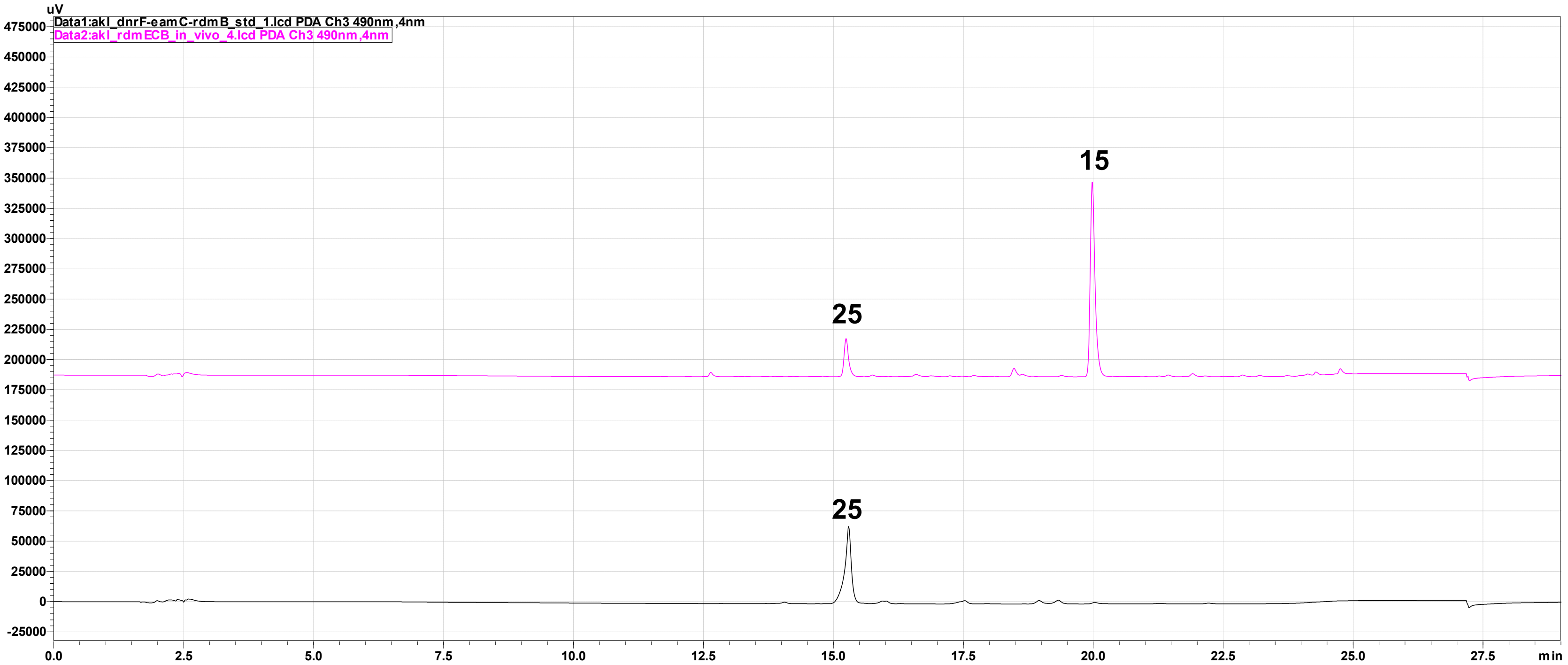

Figure S101. Alignment of β-rhodomycinone (**25**) produced both *in vitro* and *in vivo.*

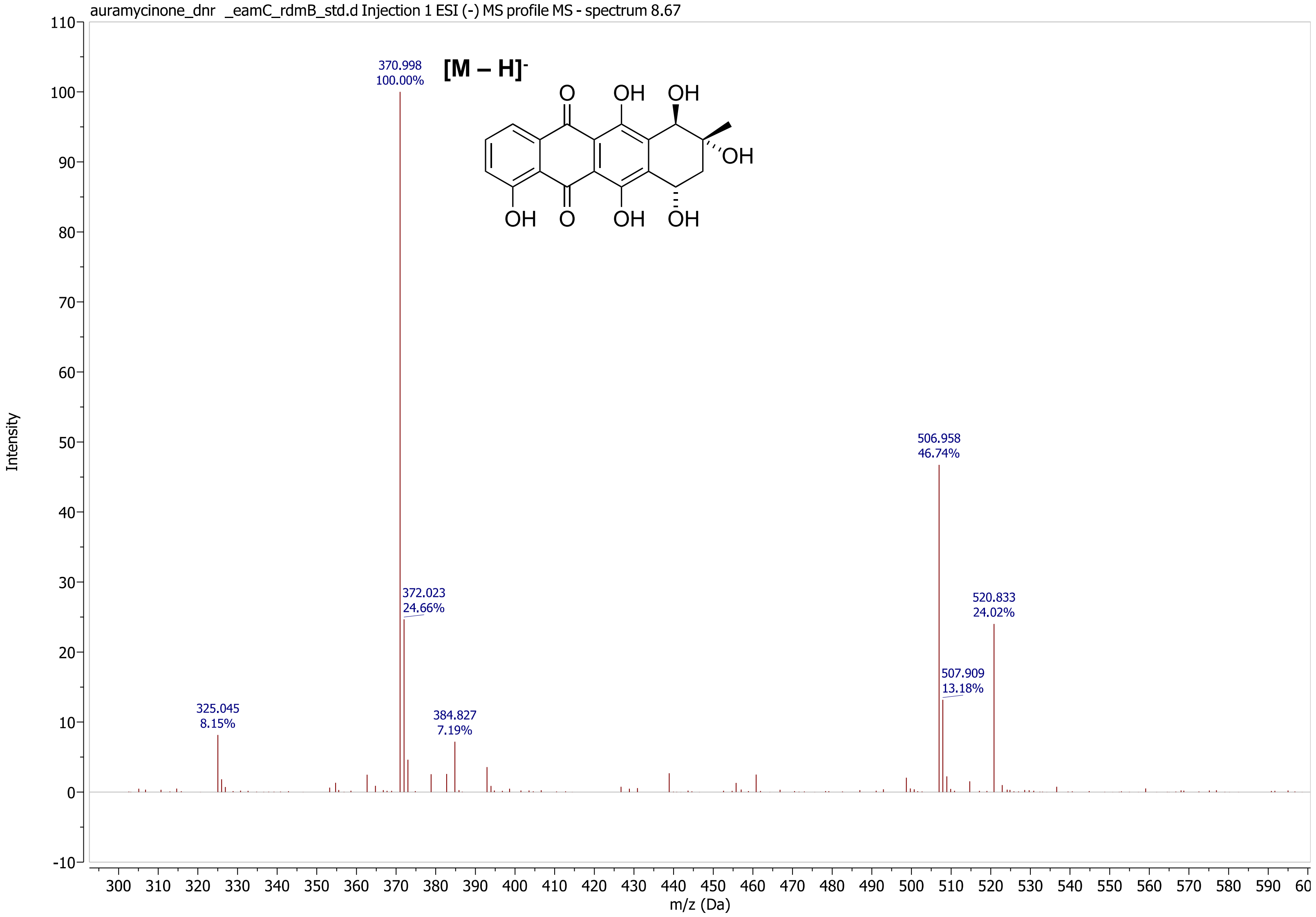

Figure S102. Mass spectrum of 10,11-dihydroxy-auramycinone (**26**) produced in *in vitro* assay with purified auramycinone and DnrF+EamC+RdmB.

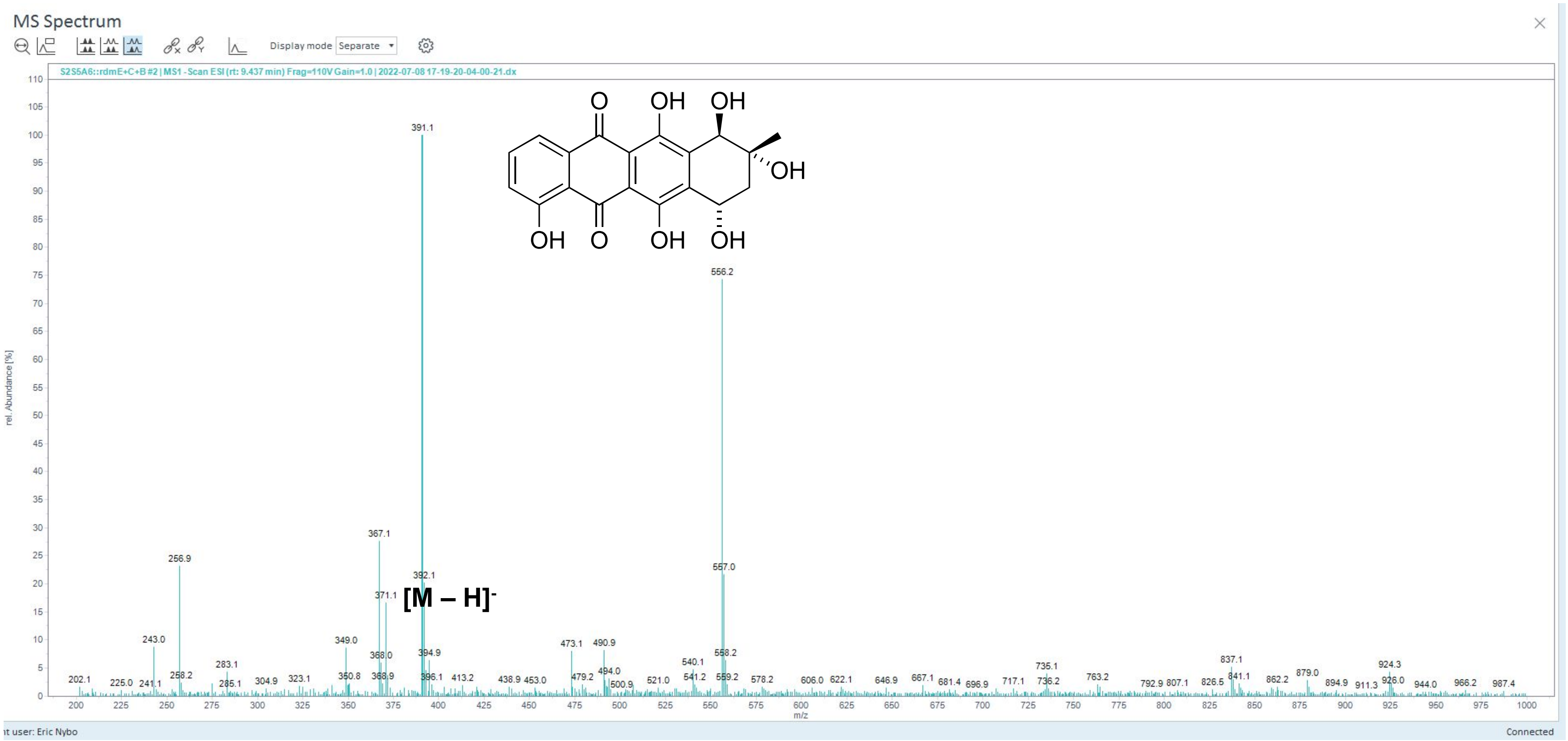

Figure S103. Mass spectrum of 10,11-dihydroxy-auramycinone (**26**) identified from strain co-expressing the auramycinone pathway and *rdmE+C+B.*

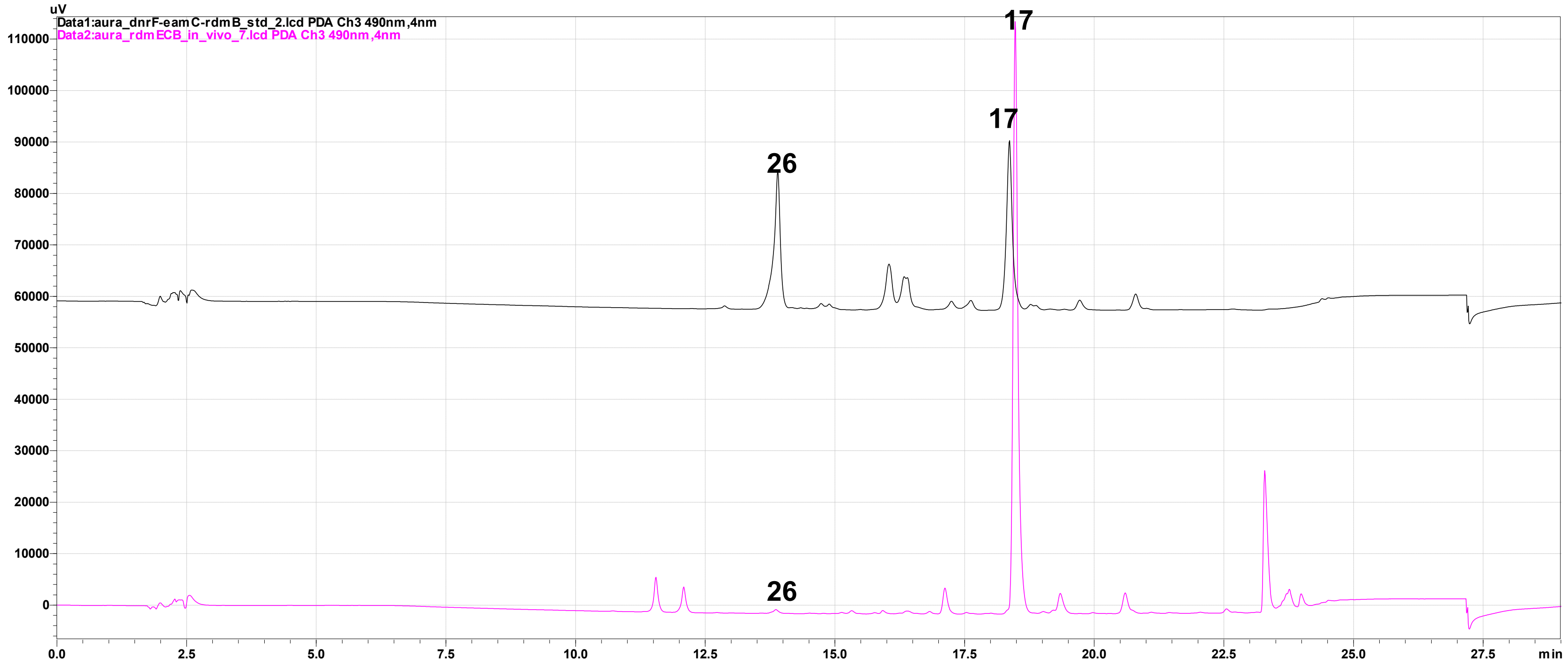

Figure S104. Alignment of 10,11-dihydroxy-auramycinone (**26**) produced both *in vitro* and *in vivo.*

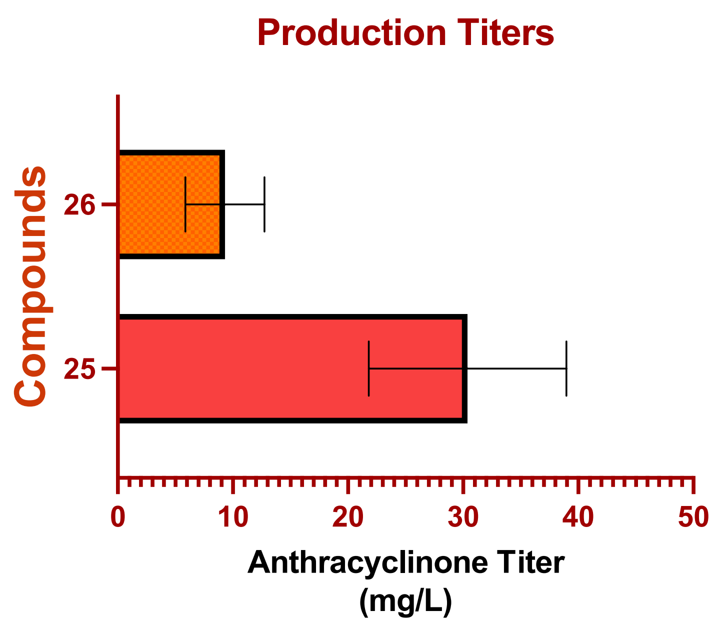

Figure S105. Production titers of compounds **25** and **26** from lines expressing the *rdmE+C+B* construct.

Print of window 80: MS Spectrum

MS Spectrum

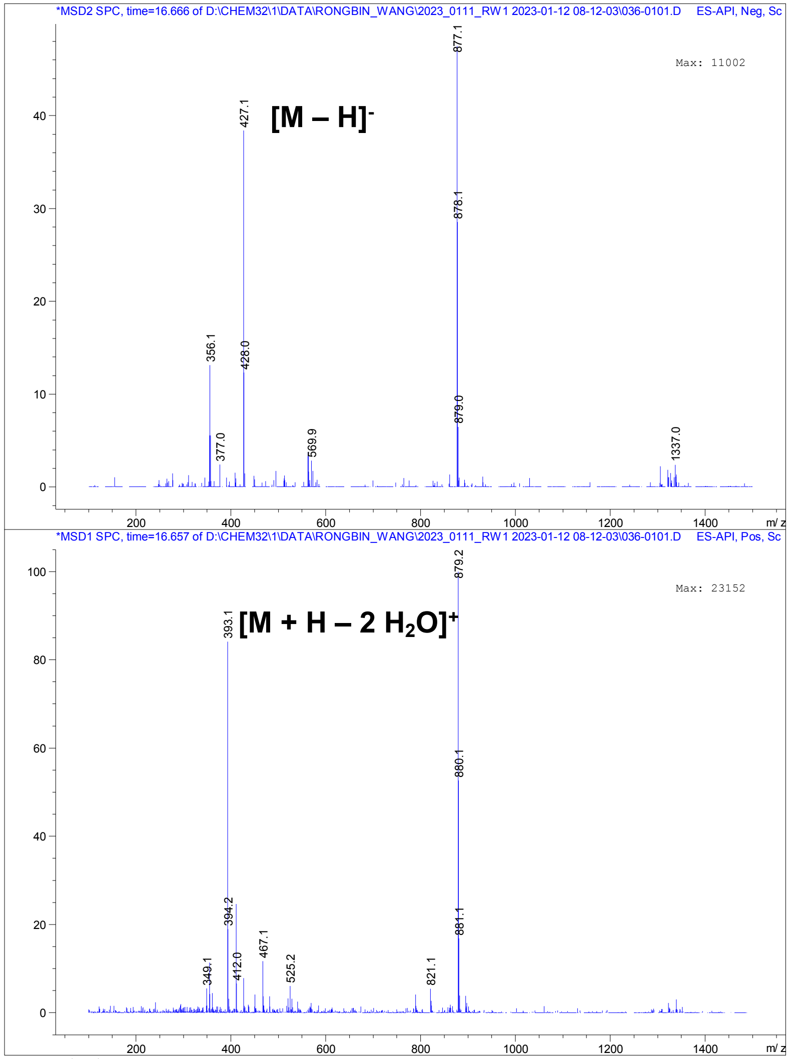

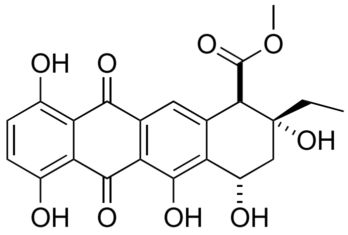

Agilent_LCMS 1/15/2023 11:52:43 PM SYSTEM Page 1 of 1

Figure S106. Mass spectrum of 1-hydroxy-aklavinone (**27**) produced in *in vitro* assay with purified aklavinone and KstA15+A16.

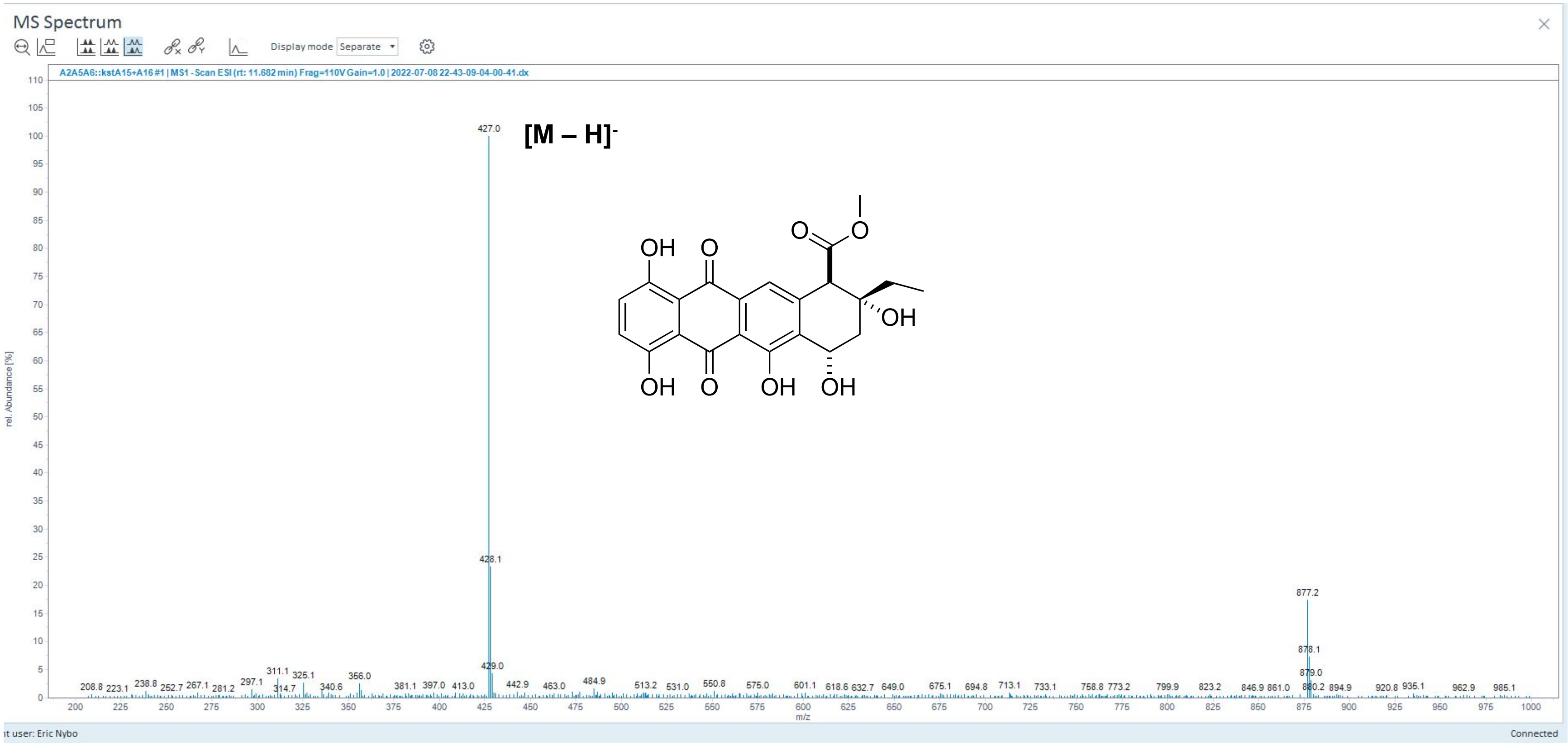

Figure S107. Mass spectrum of 1-hydroxy-aklavinone (**27**) identified from a strain co-expressing the aklavinone pathway and KstA15+A16.

**Sample Name**	KS_FSU_E5	**Position**	P1-B6	**Instrument Name**	Instrument 1
**User Name**		**Inj Vol**	5	**InjPosition**	
**Sample Type**	Sample	**IRM Calibration Status**	Success	**Data Filename**	KS_FSU_E5.d
**ACQ Method**	Zheng_AQC ACC short_Neg.m	**Comment**		**Acquired Time**	11/13/2023 6:38:08 PM (UTC-05:00)

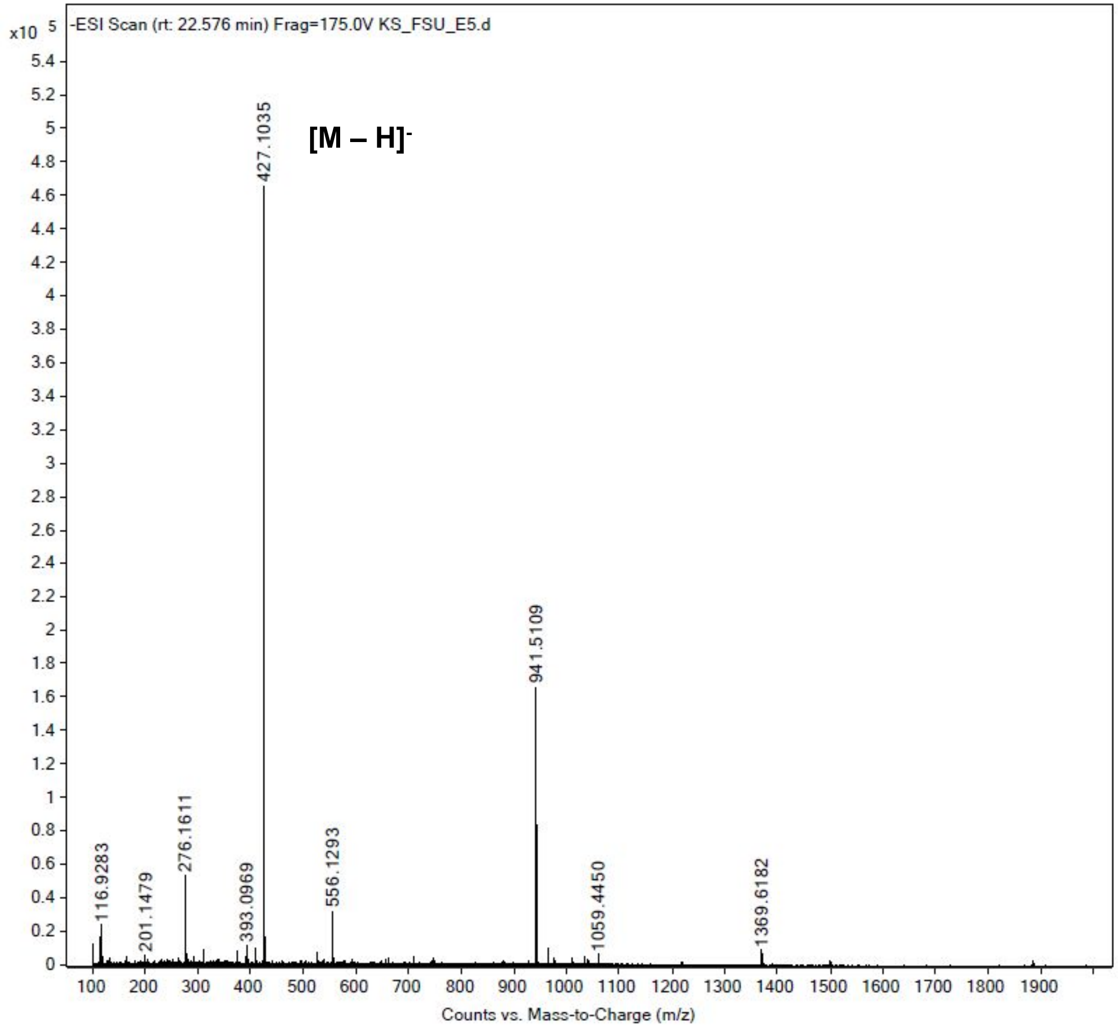

Figure S108. (-)-HRESI-MS spectrum of 1-hydroxy-aklavinone (**27**).

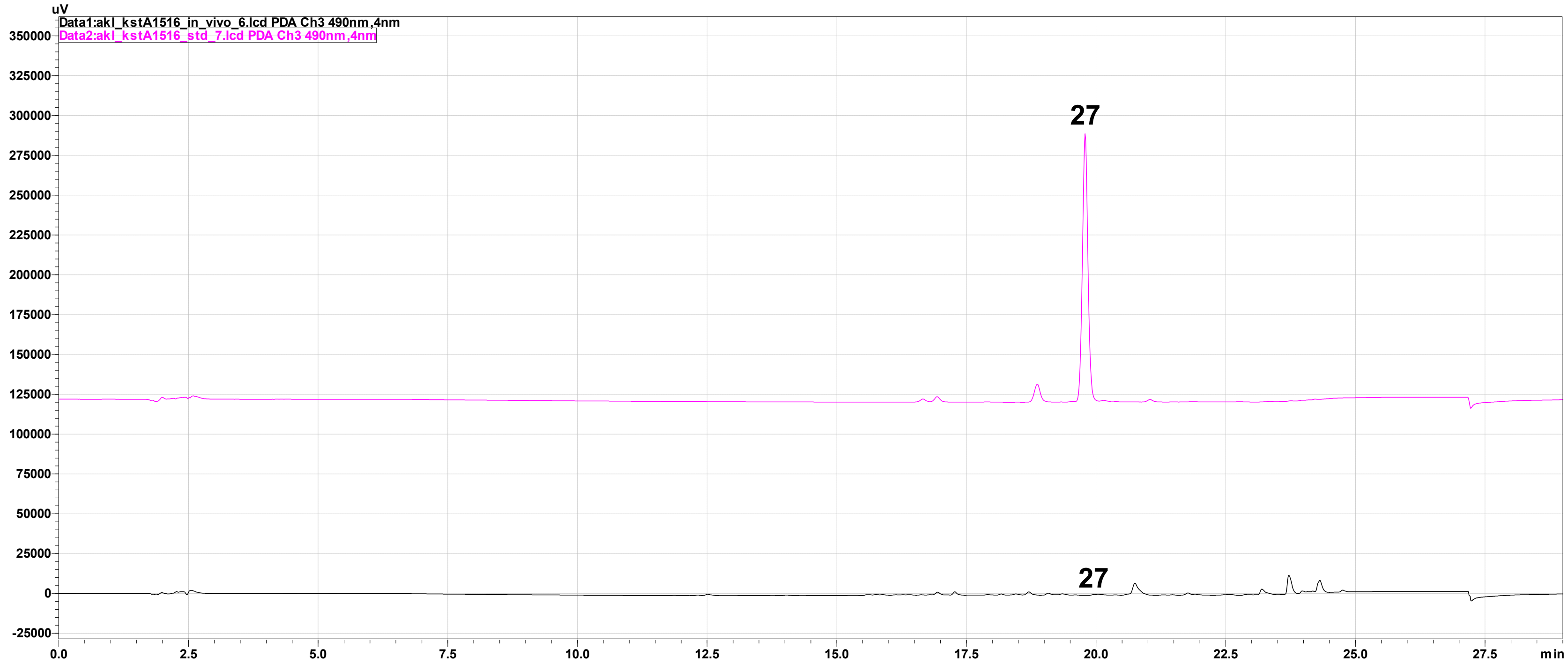

Figure S109. Alignment of 1-hydroxy-aklavinone (**27**) produced *in vitro* and *in vivo.*

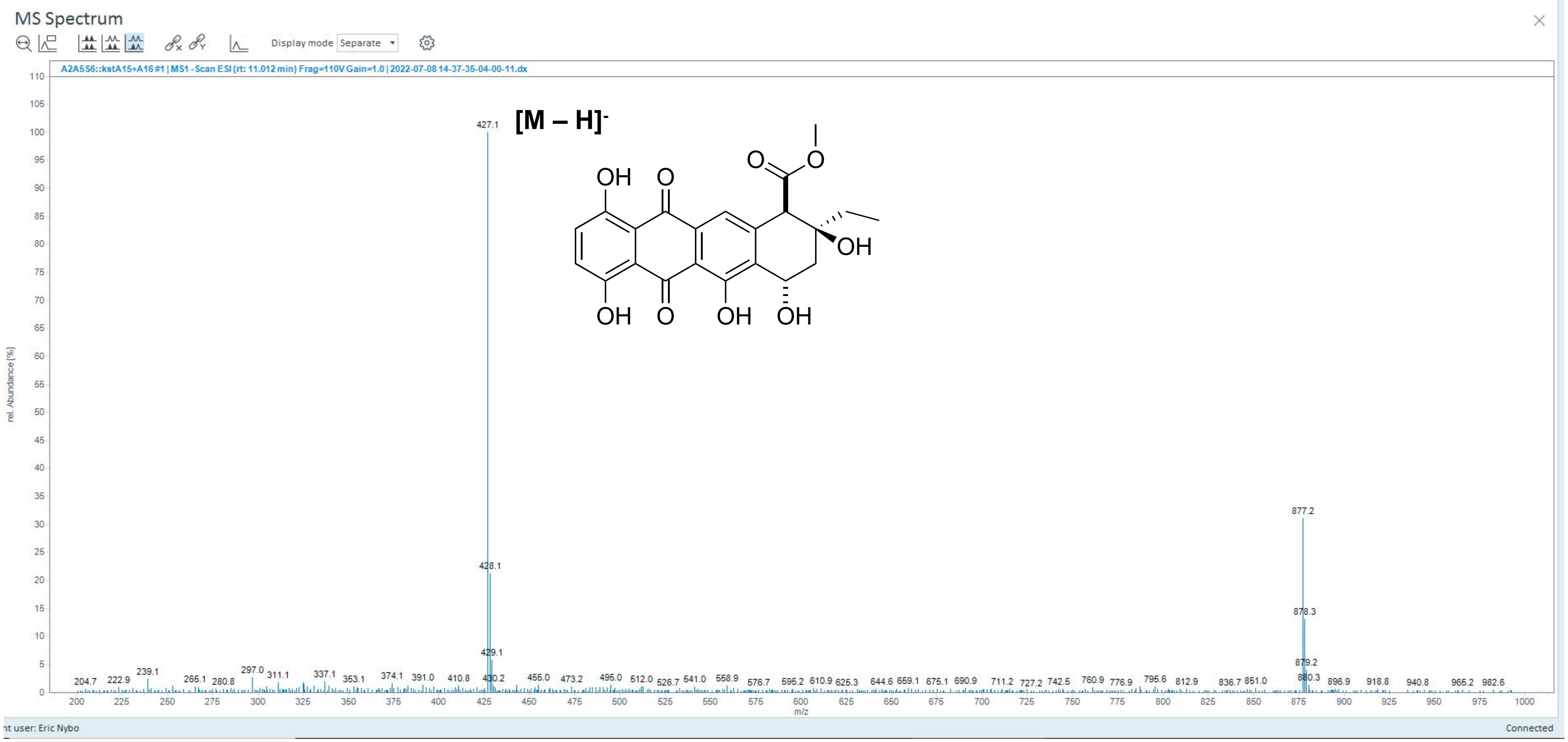

Figure S110. Mass spectrum of 1-hydroxy-9-*epi*-aklavinone (**28**) identified from strain co-expressing the 9-*epi*-aklavinone pathway with *kstA15+A16.*

**Sample Name**	KS_FSU_E10	**Position**	P1-B11	**Instrument Name**	Instrument 1
**User Name**		**Inj Vol**	5	**InjPosition**	
**Sample Type**	Sample	**IRM Calibration Status**	Success	**Data Filename**	KS_FSU_E10.d
**ACQ Method**	Zheng_AQC ACC short_Neg.m	**Comment**		**Acquired Time**	11/13/2023 9:03:12 PM (UTC-05:00

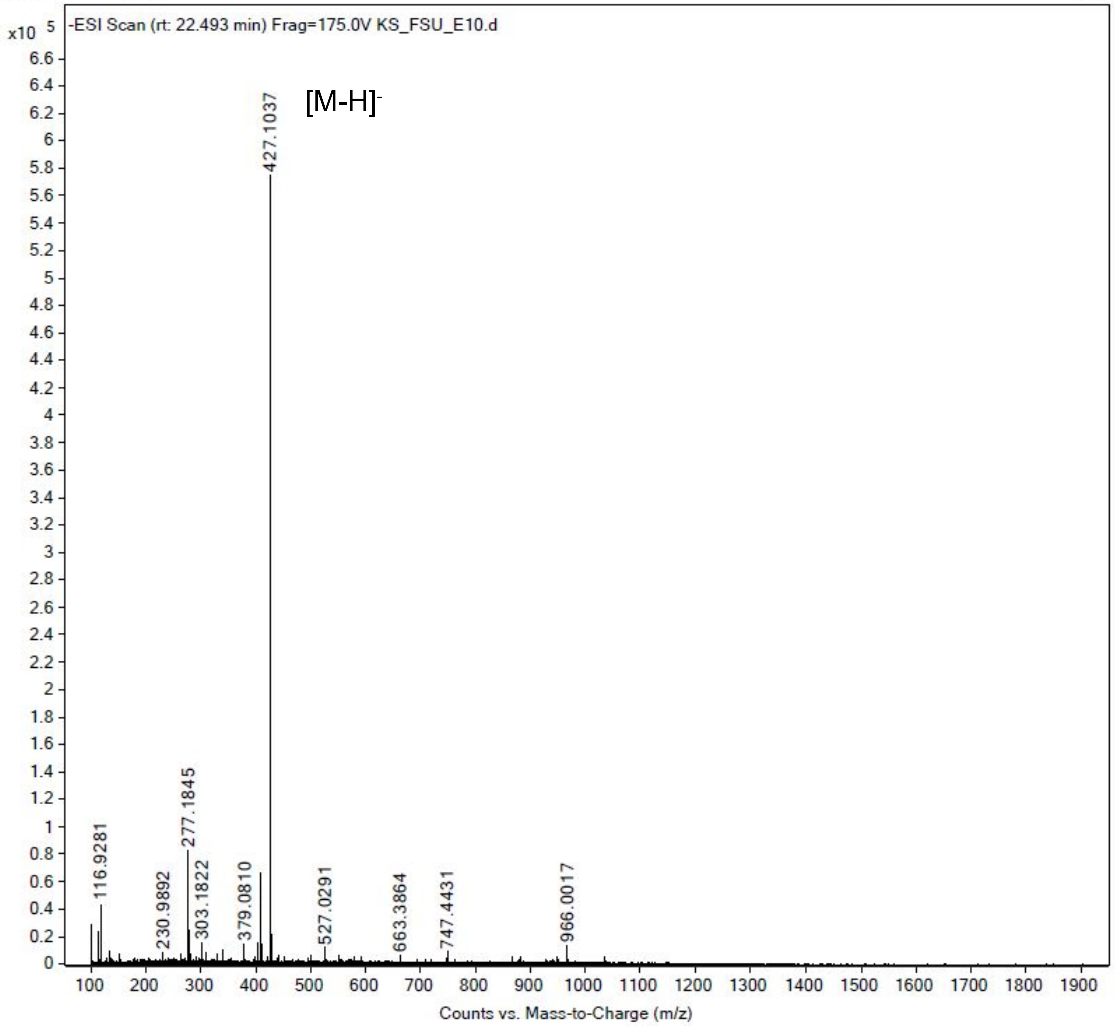

Figure S111. (-)-HRESI-MS spectrum of 1-hydroxy-9-*epi*-aklavinone (**28**).

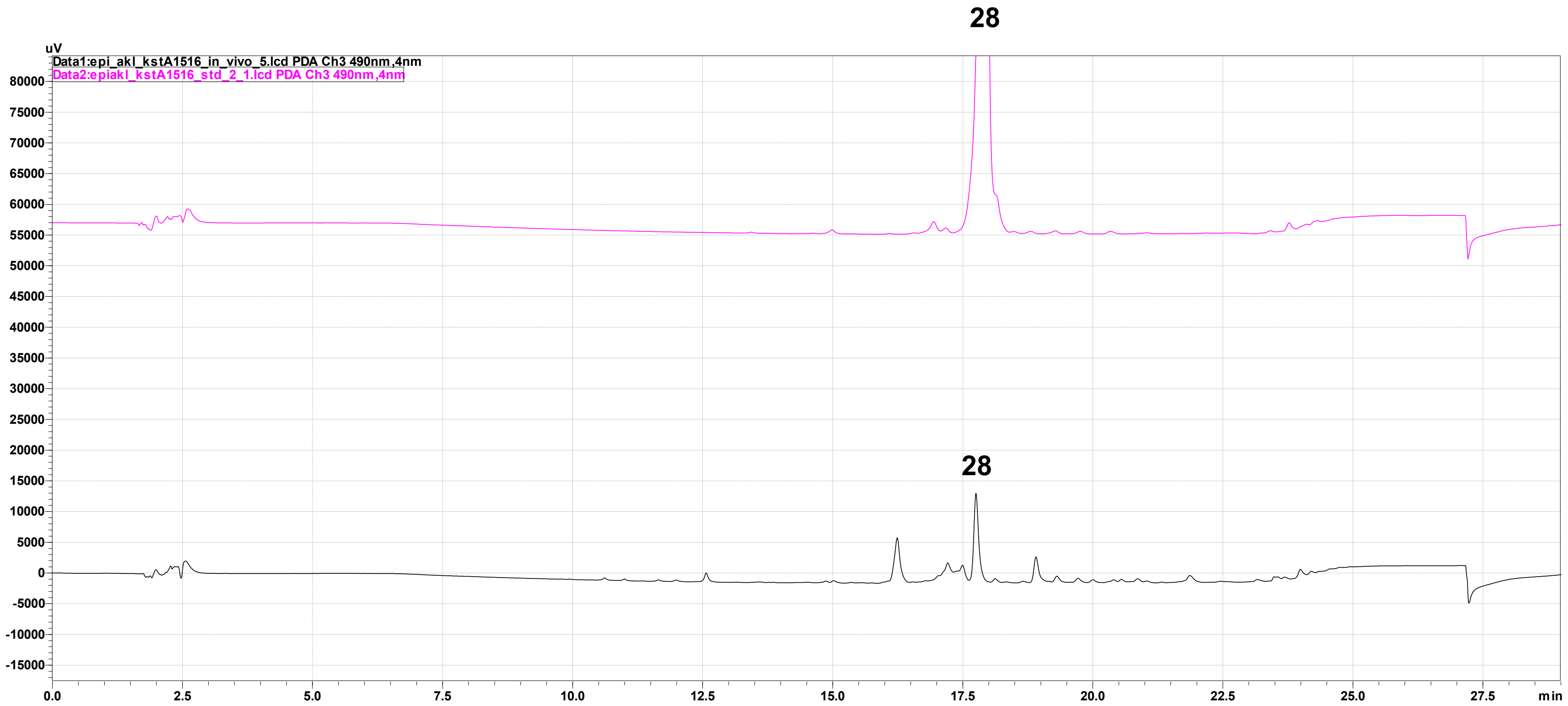

Figure S112. Alignment of 1-hydroxy-9-*epi*-aklavinone (**28**) produced *in vitro* and *in vivo*.

Print of window 80: MS Spectrum

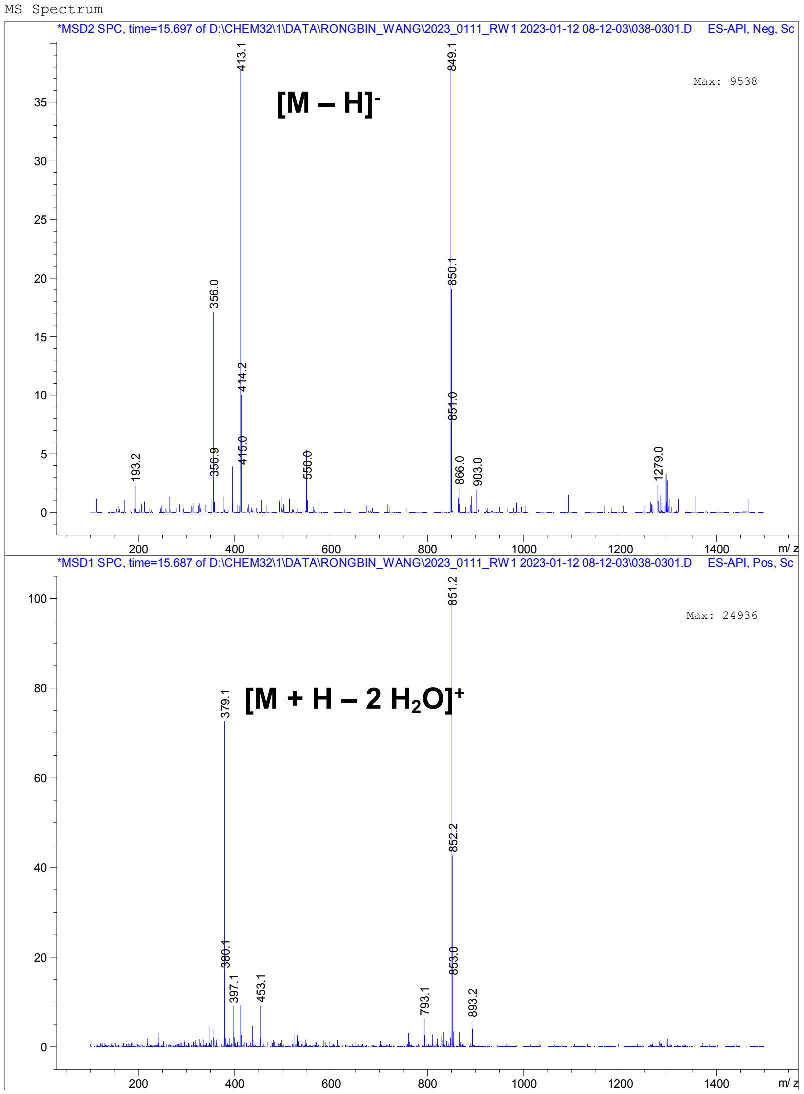

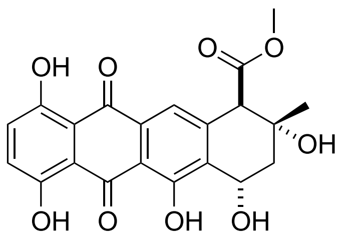

Agilent_LCMS 1/15/2023 11:54:00 PM SYSTEM Page 1 of 1

Figure S113. Mass spectrum of 1-hydroxy-auramycinone (**29**) produced in *in vitro* assay with purified auramycinone and KstA15+A16.

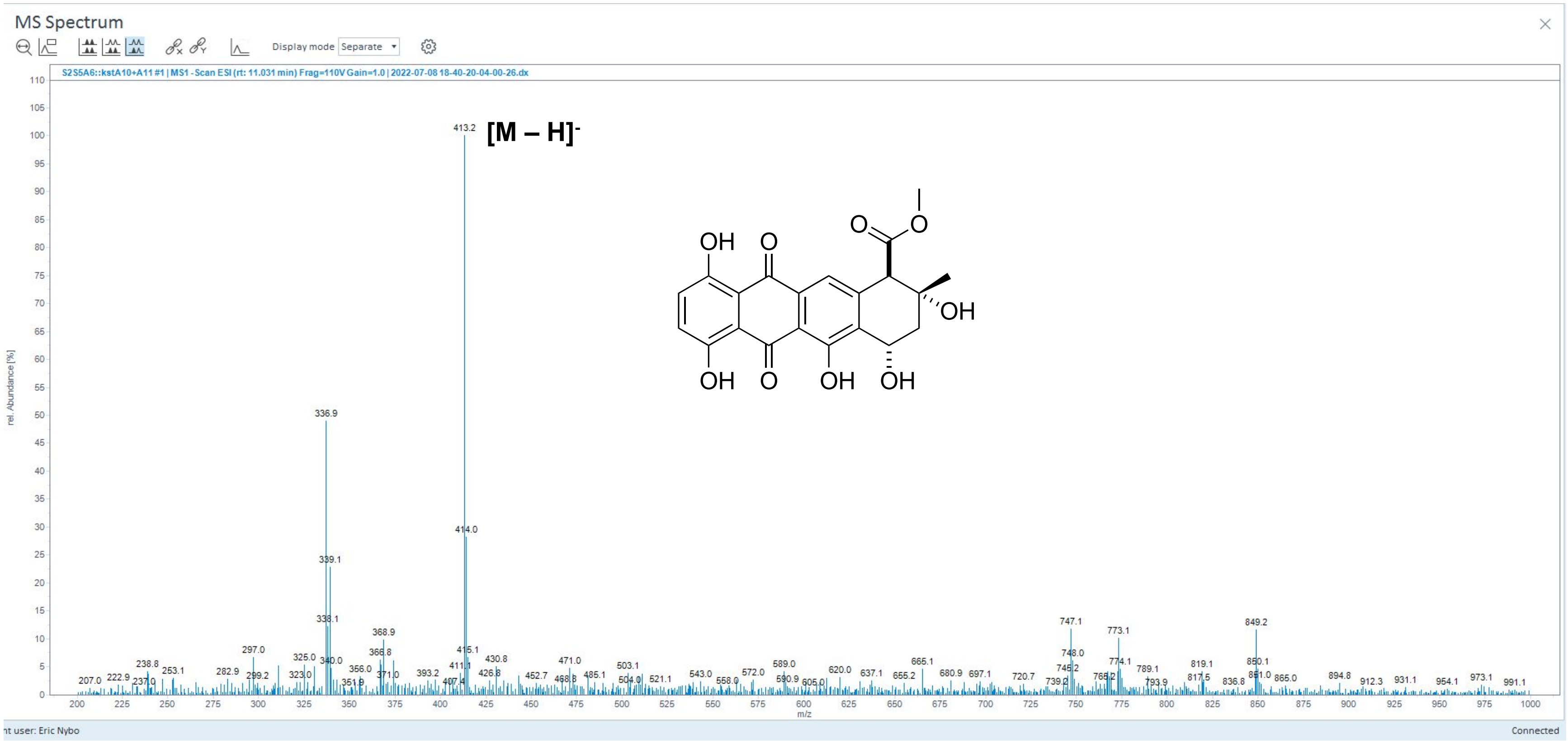

Figure S114. Mass spectrum of 1-hydroxy-auramycinone (**29**) identified from a strain co-expressing the auramycinone pathway and *kstA15+A16.*

**Sample Name**	KS_FSU_E13	**Position**	P1-C3	**Instrument Name**	Instrument 1
**User Name**		**Inj Vol**	5	**InjPosition**	
**Sample Type**	Sample	**IRM Calibration Status**	Success	**Data Filename**	KS_FSU_E13.d
**ACQ Method**	Zheng_AQC ACC short_Neg.m	**Comment**		**Acquired Time**	11/13/2023 10:30:13 PM (UTC-05:00)

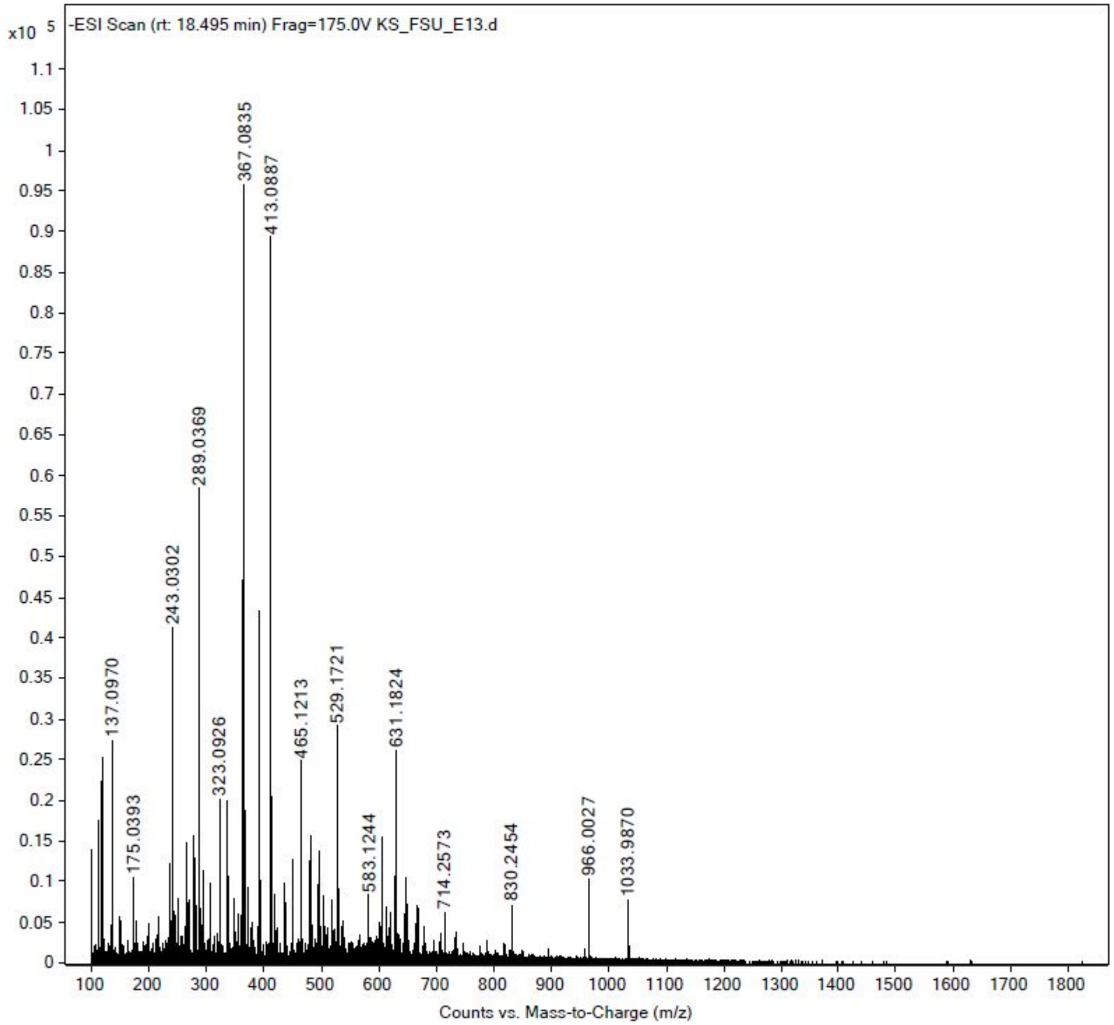

Figure S115. (-)-HRESI-MS spectrum of 1-hydroxy-auramycinone (**29**).

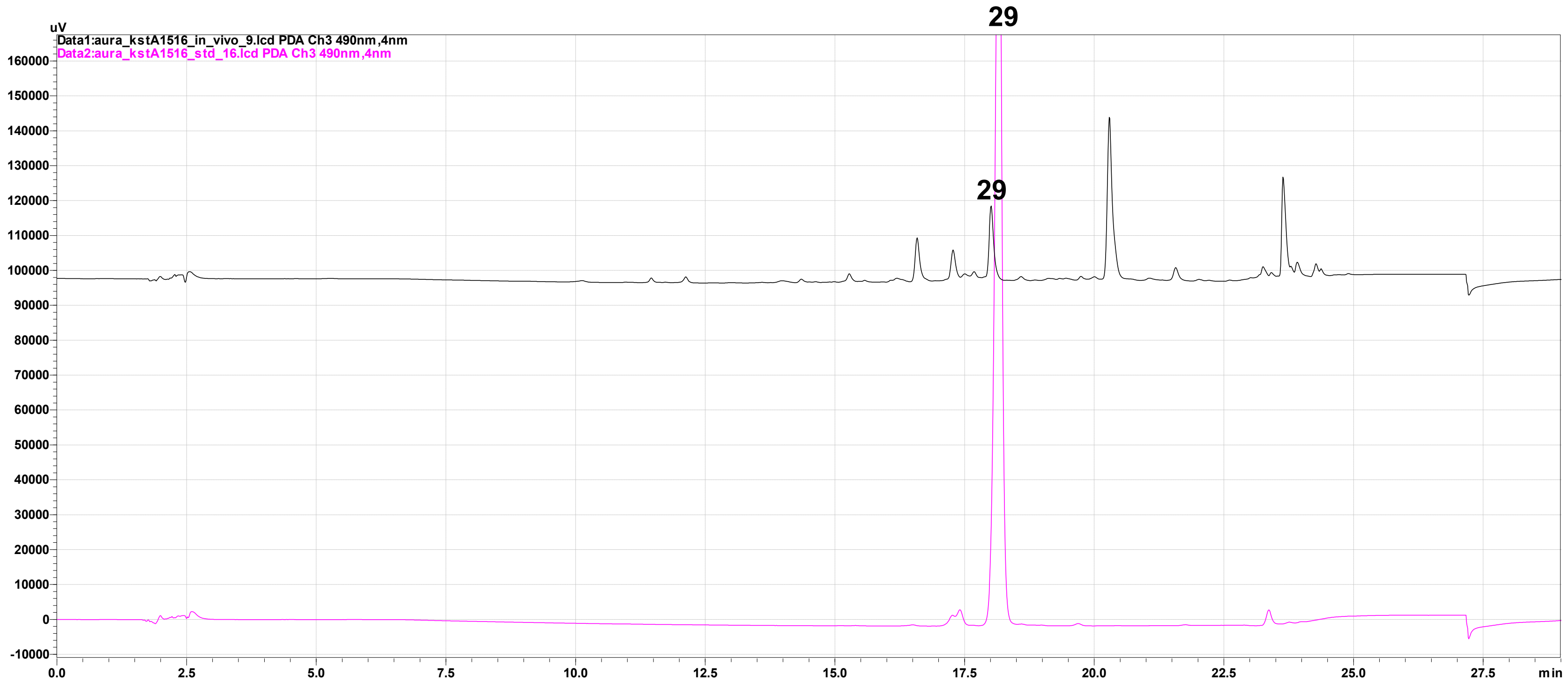

Figure S116. Alignment of 1-hydroxy-auramycinone (**29**) produced both *in vitro* and *in vivo.*

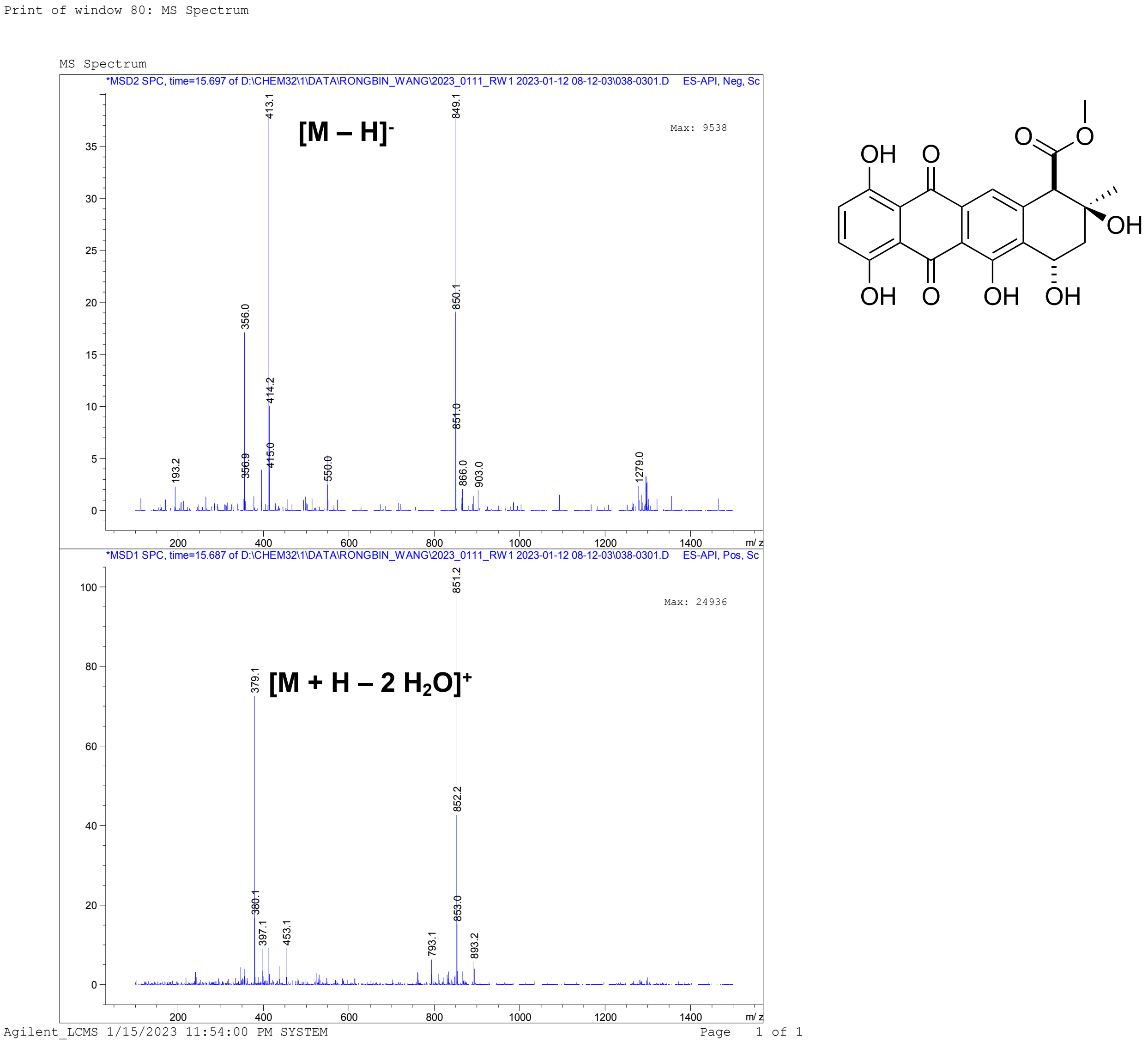

Figure S117. Mass spectrum of 1-hydroxy-nogalamycinone (**30**) produced in *in vitro* assay with purified nogalamycinone and KstA15+A16.

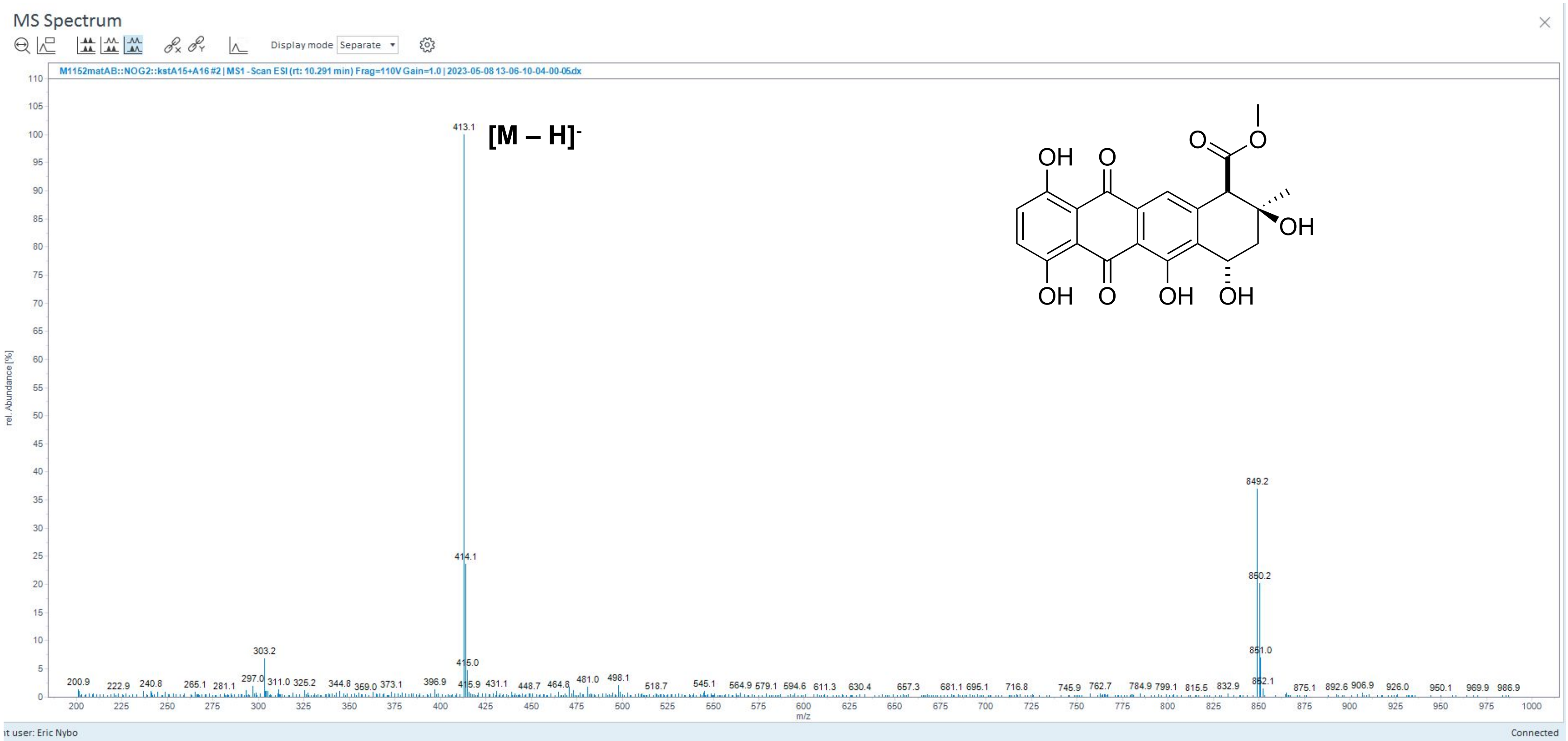

Figure S118. Mass spectrum of 1-hydroxy-nogalamycinone (**30**) identified from strain co-expressing the nogalamycinone pathway and *kstA15+A16.*

**Sample Name**	KS_FSU_E19	**Position**	P1-C9	**Instrument Name**	Instrument 1
**User Name**		**Inj Vol**	5	**InjPosition**	
**Sample Type**	Sample	**IRM Calibration Status**	Success	**Data Filename**	KS_FSU_E19.d
**ACQ Method**	Zheng_AQC ACC short_Neg.m	**Comment**		**Acquired Time**	11/14/2023 1:24:18 AM (UTC-05:00)

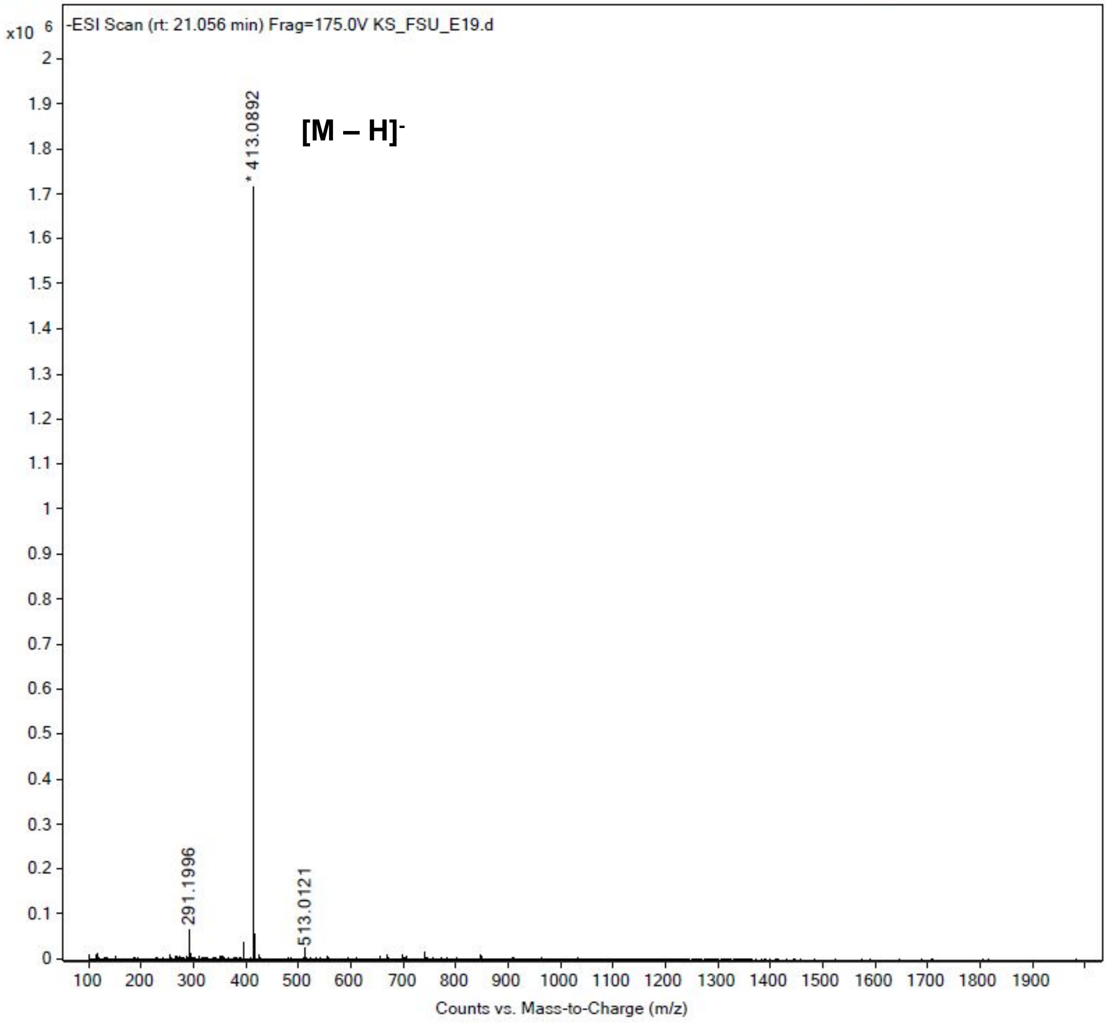

Figure S119. (-)-HRESI-MS spectrum of 1-hydroxy-nogalamycinone (**30**).

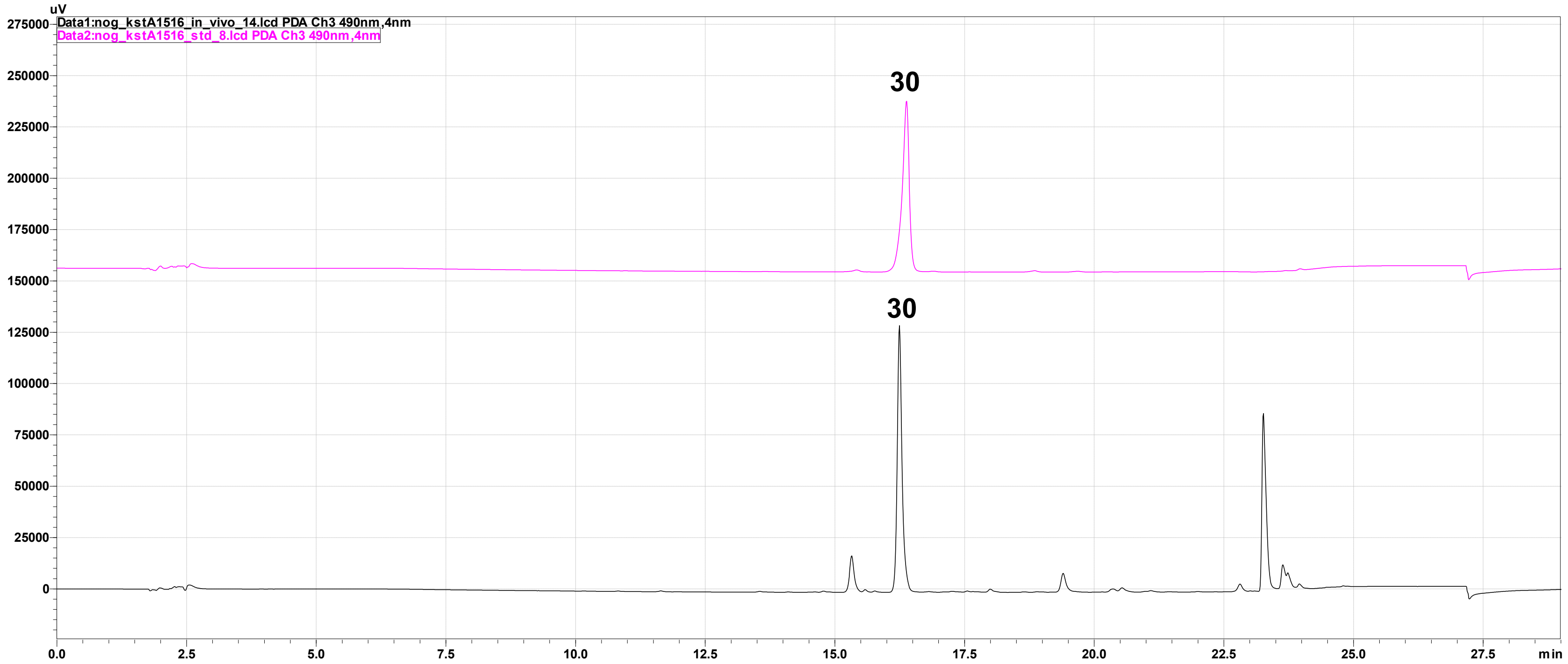

Figure S120. Alignment of 1-hydroxy-nogalamycinone (**30**) produced *in vitro* and *in vivo*.

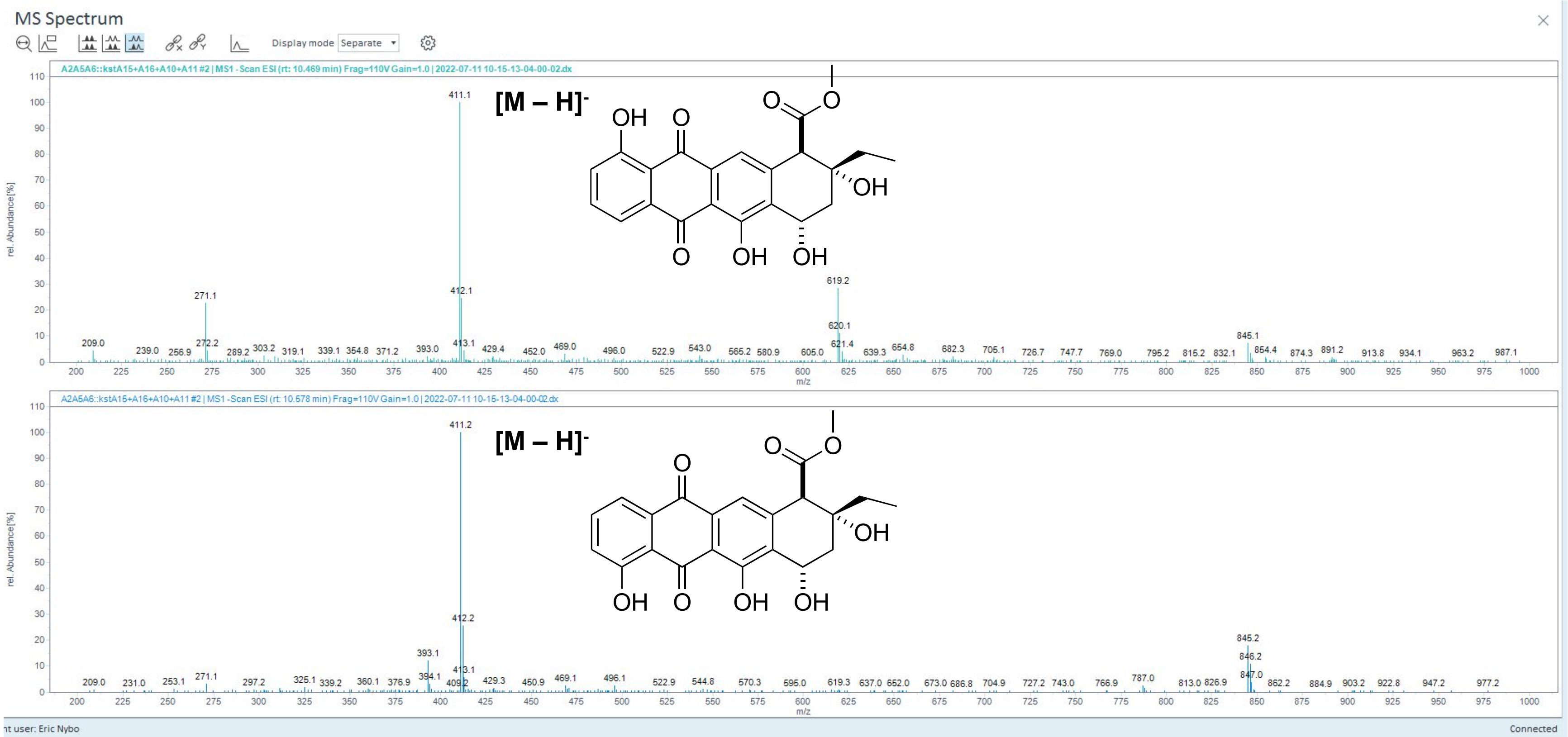

Figure S121. Mass spectrum of aklavinone (**1**) and *iso*-aklavinone (**31**) identified from a strain co-expressing the aklavinone pathway and *kstA15+A16+A10+A11.*

**Sample Name**	KS_FSU_E5	**Position**	P1-B6	**Instrument Name**	Instrument 1
**User Name**		**Inj Vol**	5	**InjPosition**	
**Sample Type**	Sample	**IRM Calibration Status**	Success	**Data Filename**	KS_FSU_E5.d
**ACQ Method**	Zheng_AQC ACC short_Neg.m	**Comment**		**Acquired Time**	11/13/2023 6:38:08 PM (UTC-05:00)

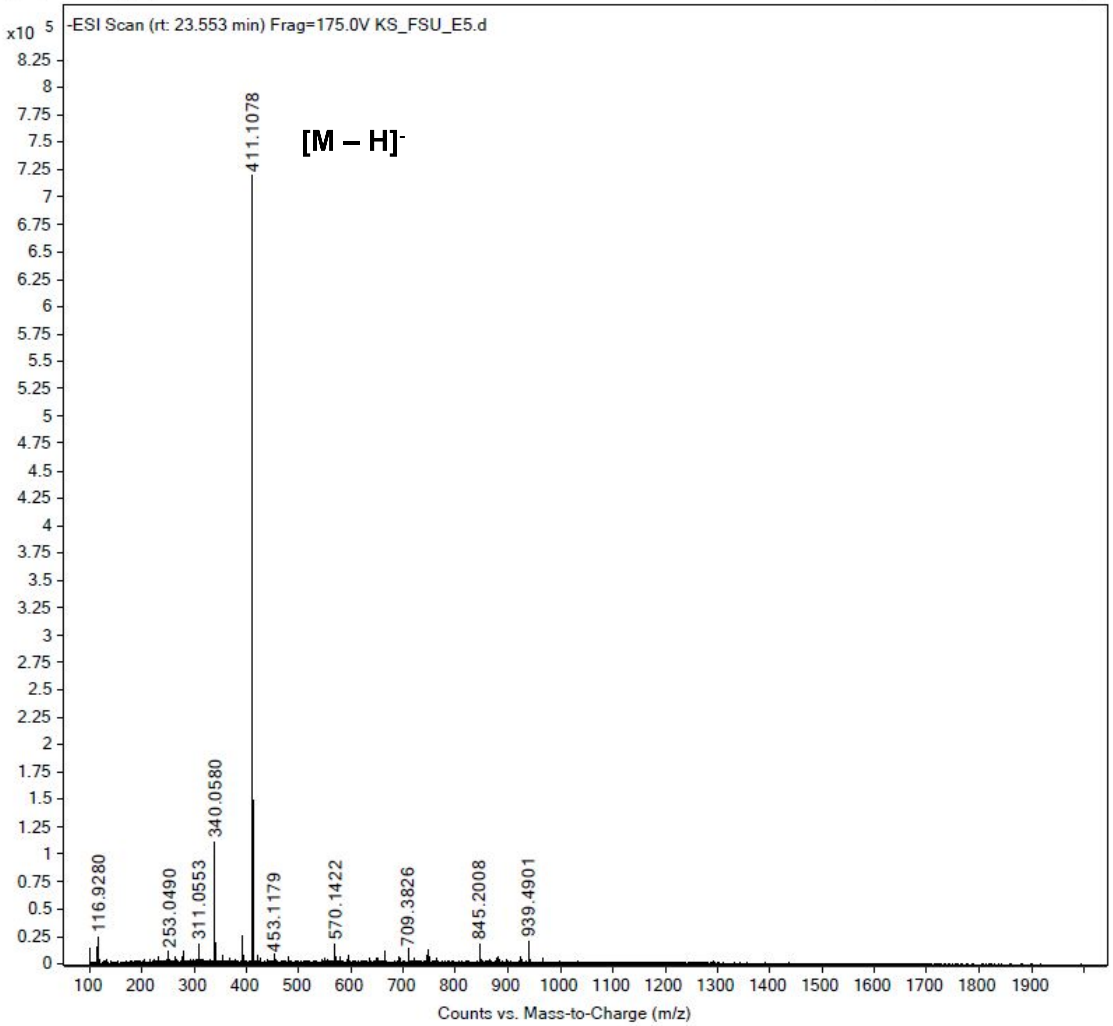

Figure S122. (-)-HRESI-MS spectrum of 1-iso-aklavinone (**31**).

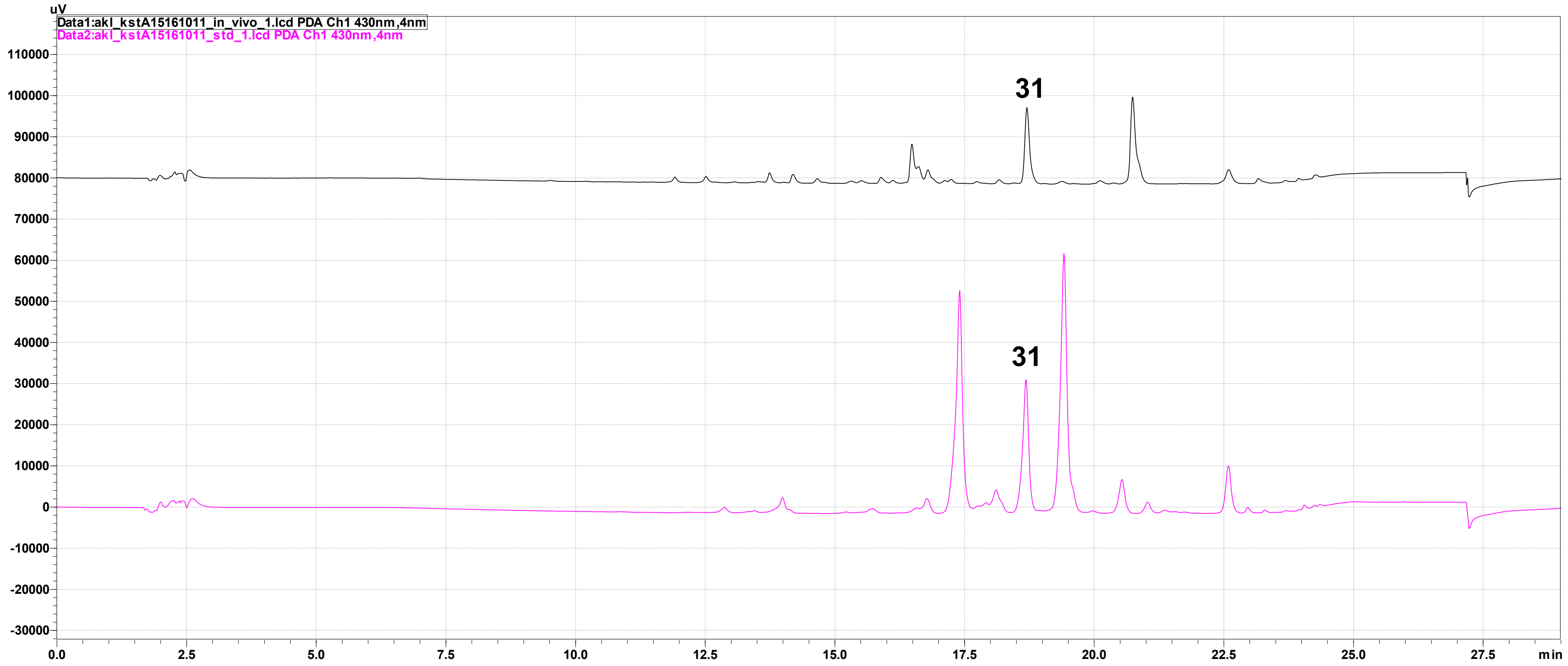

Figure S123. Alignment of *iso*-aklavinone (**31**) produced both *in vitro* and *in vivo.*

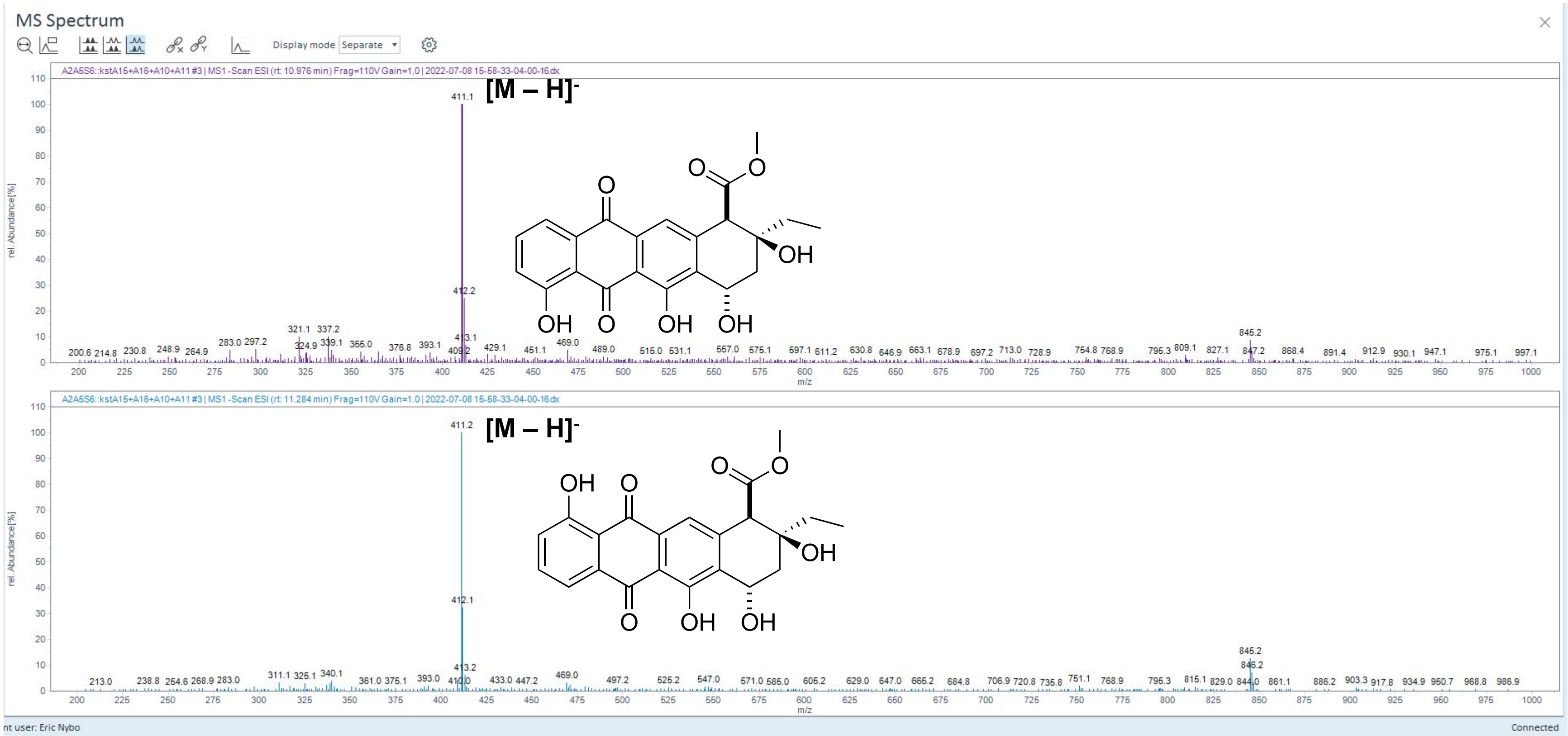

Figure S124. Mass spectrum of 9-*epi*-aklavinone (**2**) and *iso*-9-*epi*-aklavinone (**32**) produced from a strain co-expressing the 9-*epi*-aklavinone pathway and *kstA15+A16+A10+A11.*

**Sample Name**	KS_FSU_E10	**Position**	P1-B11	**Instrument Name**	Instrument 1
**User Name**		**Inj Vol**	5	**InjPosition**	
**Sample Type**	Sample	**IRM Calibration Status**	Success	**Data Filename**	KS_FSU_E10.d
**ACQ Method**	Zheng_AQC ACC short_Neg.m	**Comment**		**Acquired Time**	11/13/2023 9:03:12 PM (UTC-05:00)

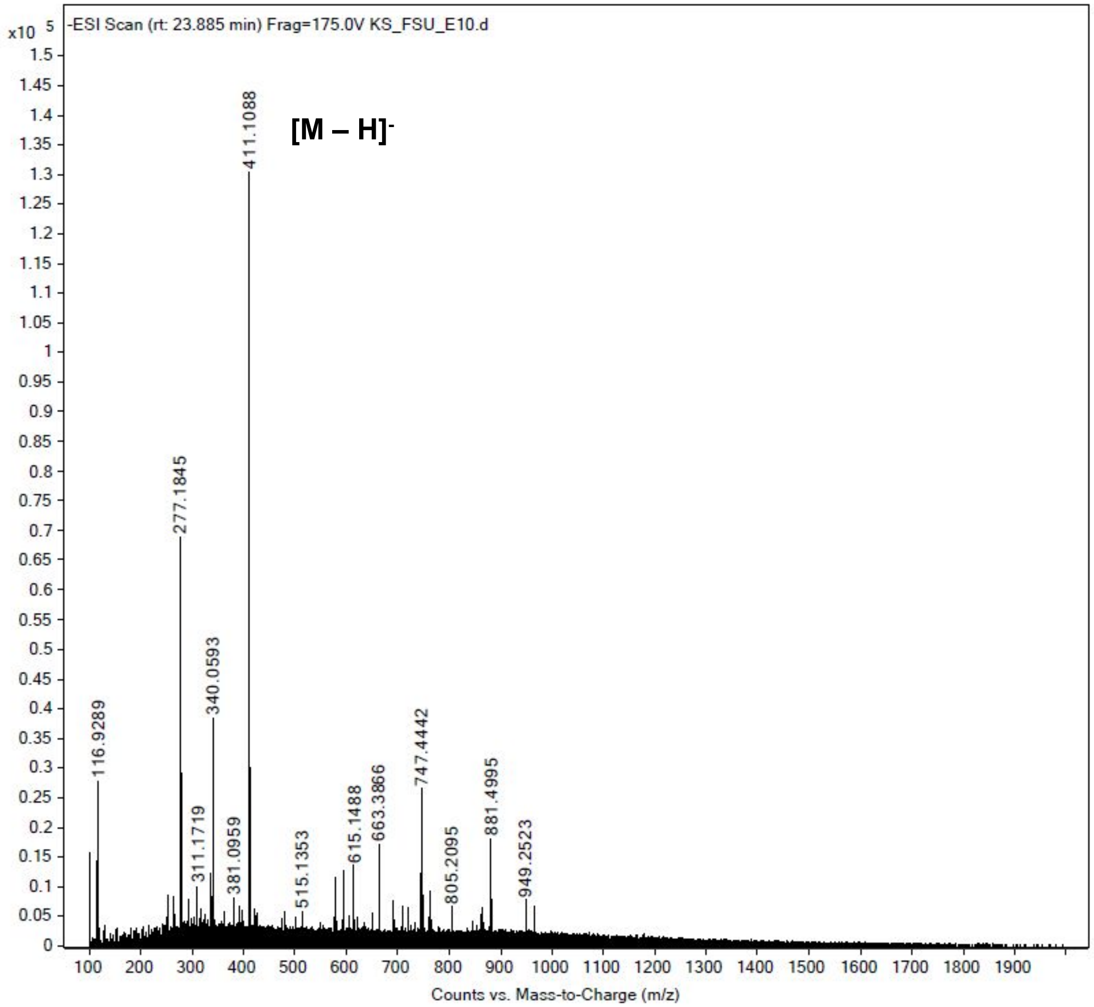

Figure S125. (-)-HRESI-MS spectrum of 1-*iso*-9-*epi*-aklavinone (**32**).

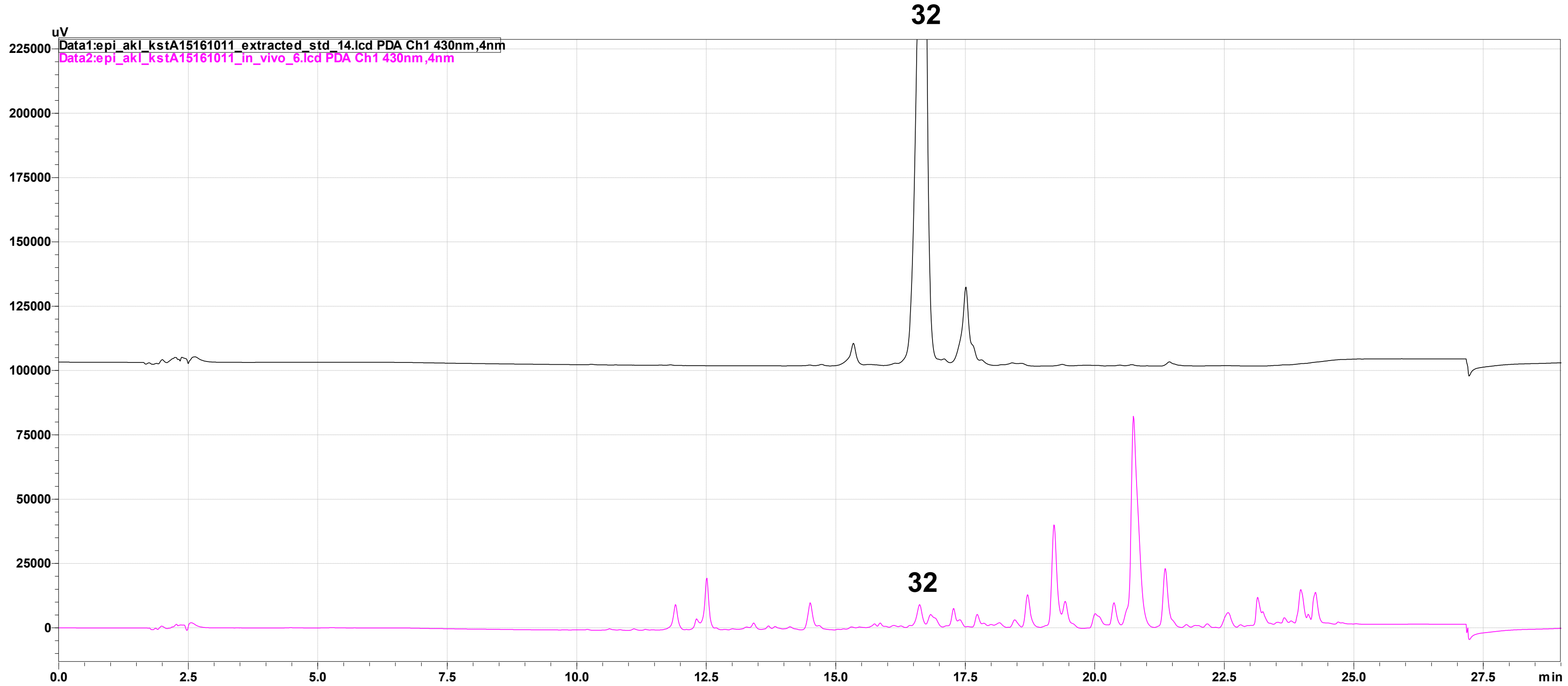

Figure S126. Alignment of *iso*-9-*epi*-aklavinone (**32**) produced both *in vitro* and *in vivo.*

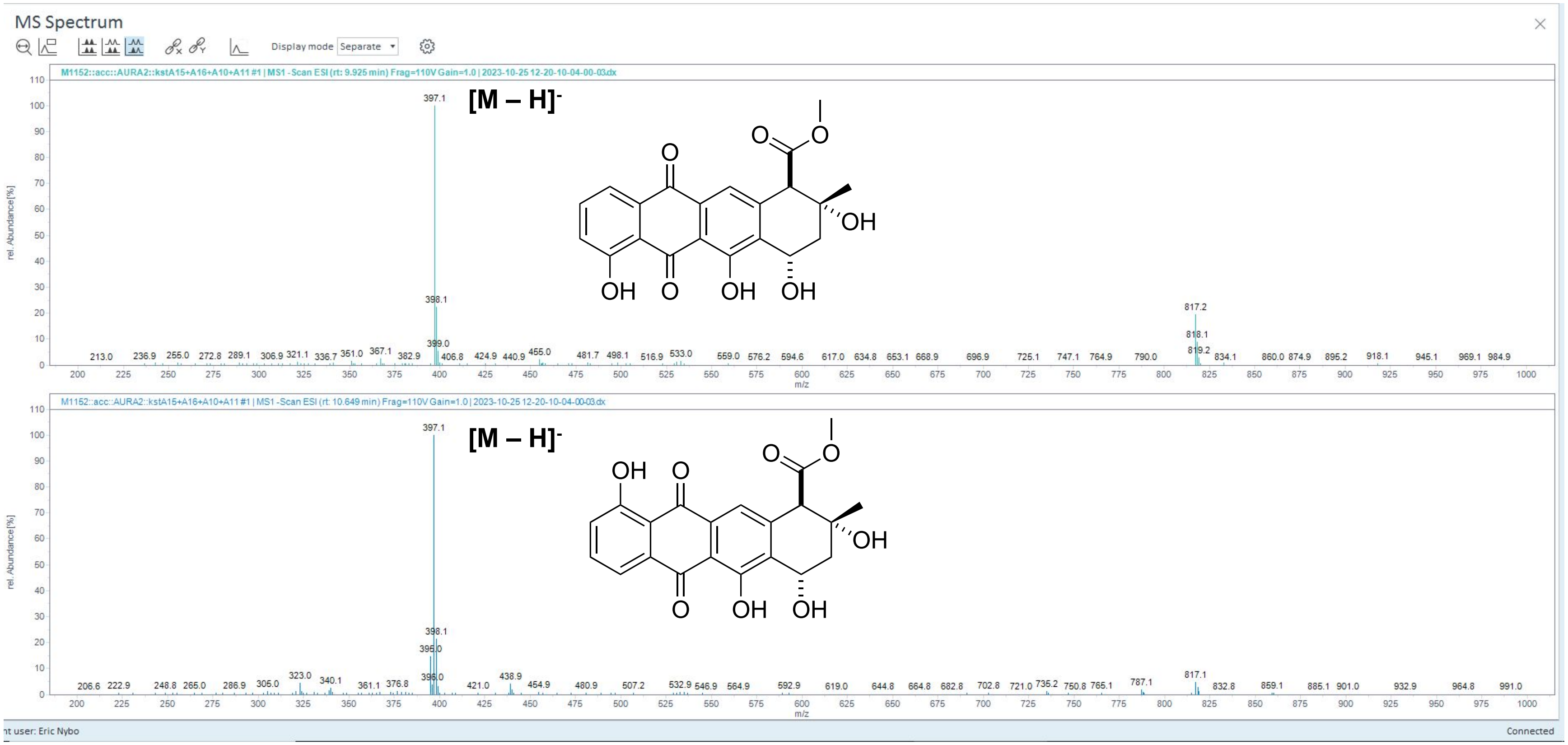

Figure S127. Mass spectrum of auramycinone (**3**) and *iso*-auramycinone (**33**) produced from a strain co-expressing the 9-*epi*-aklavinone pathway and *kstA15+A16+A10+A11.*

**Sample Name**	KS_FSU_E14	**Position**	P1-C4	**Instrument Name**	Instrument 1
**User Name**		**Inj Vol**	5	**InjPosition**	
**Sample Type**	Sample	**IRM Calibration Status**	Success	**Data Filename**	KS_FSU_E14.d
**ACQ Method**	Zheng_AQC ACC short_Neg.m	**Comment**		**Acquired Time**	11/13/2023 10:59:14 PM (UTC-05:00)

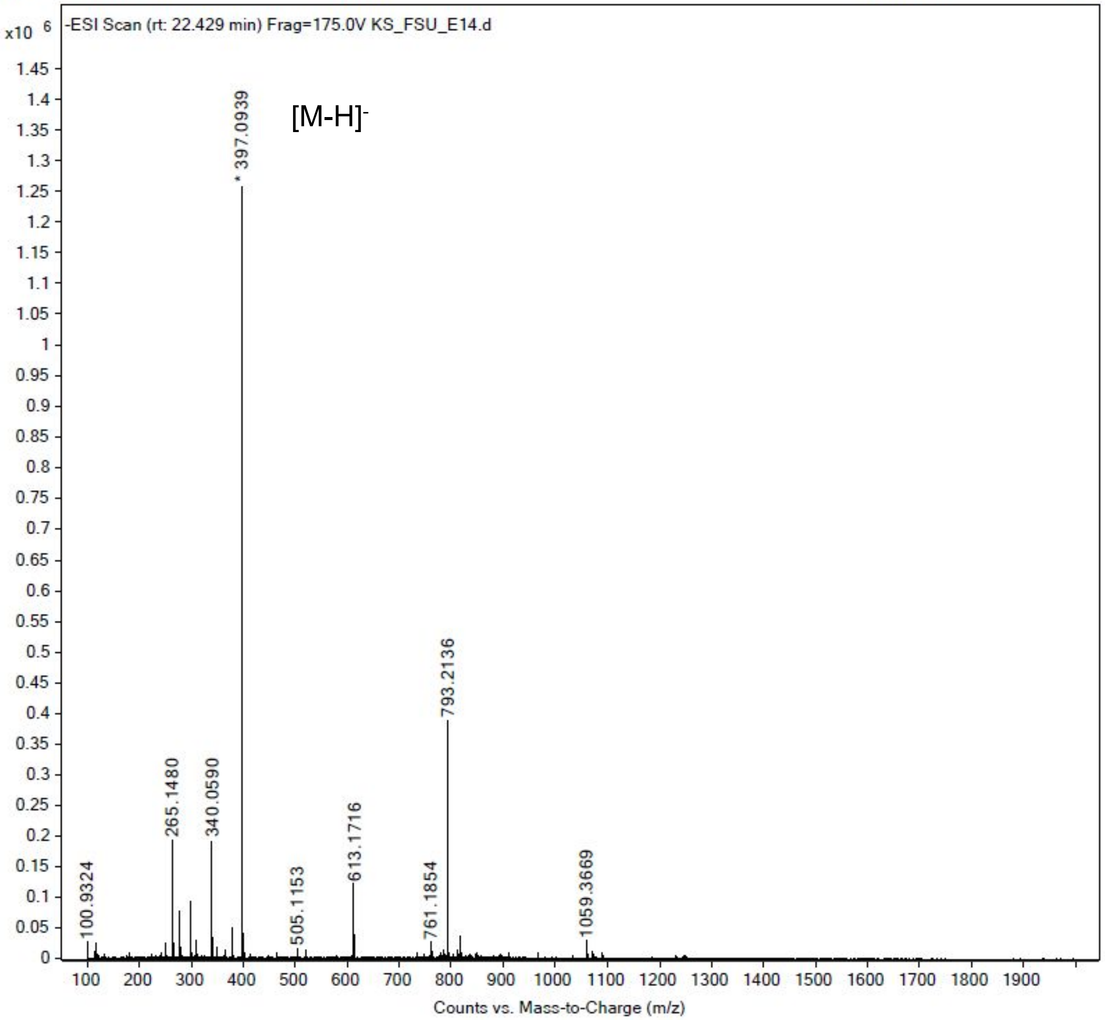

Figure S128. (-)-HRESI-MS spectrum of 1-iso-auramycinone (**33**).

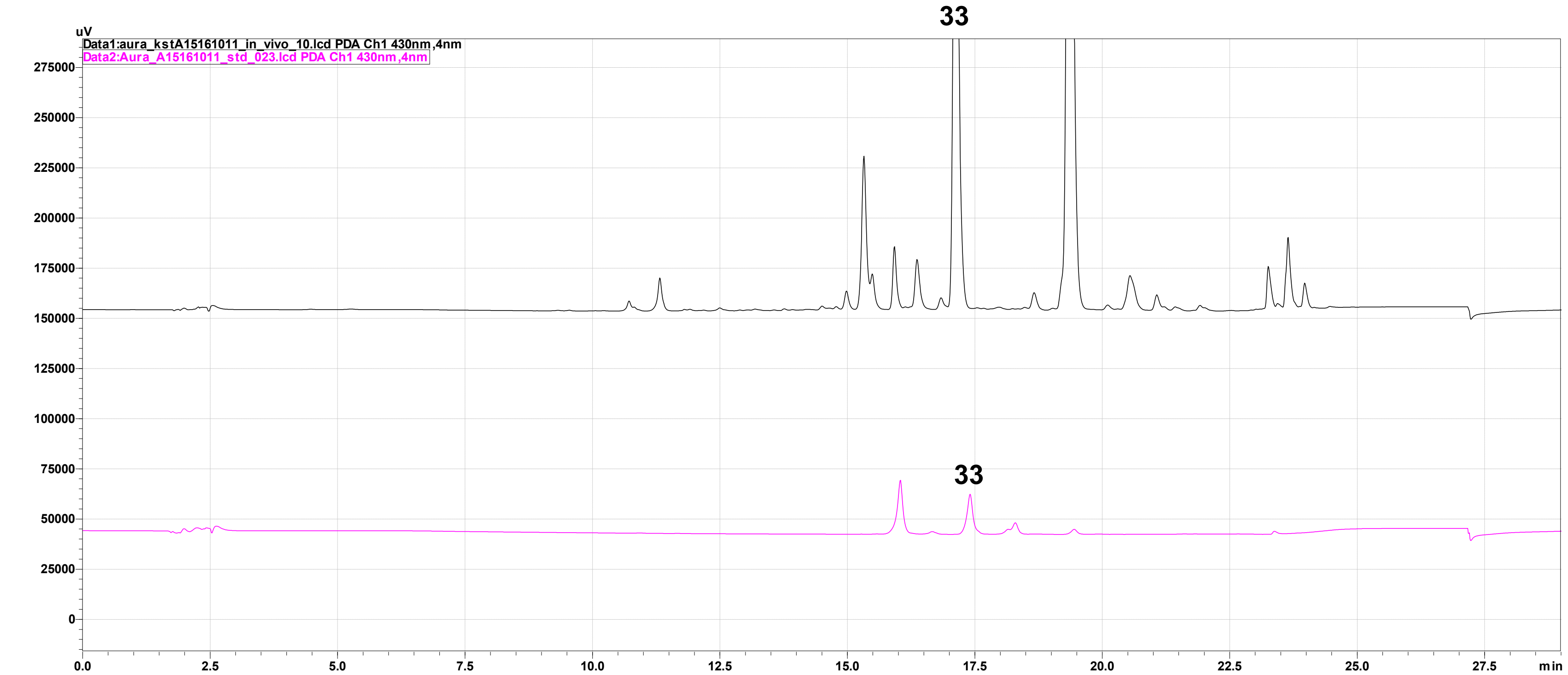

Figure S129. Alignment of *iso*-auramycinone (**33**) produced *in vitro* and *in vivo*.

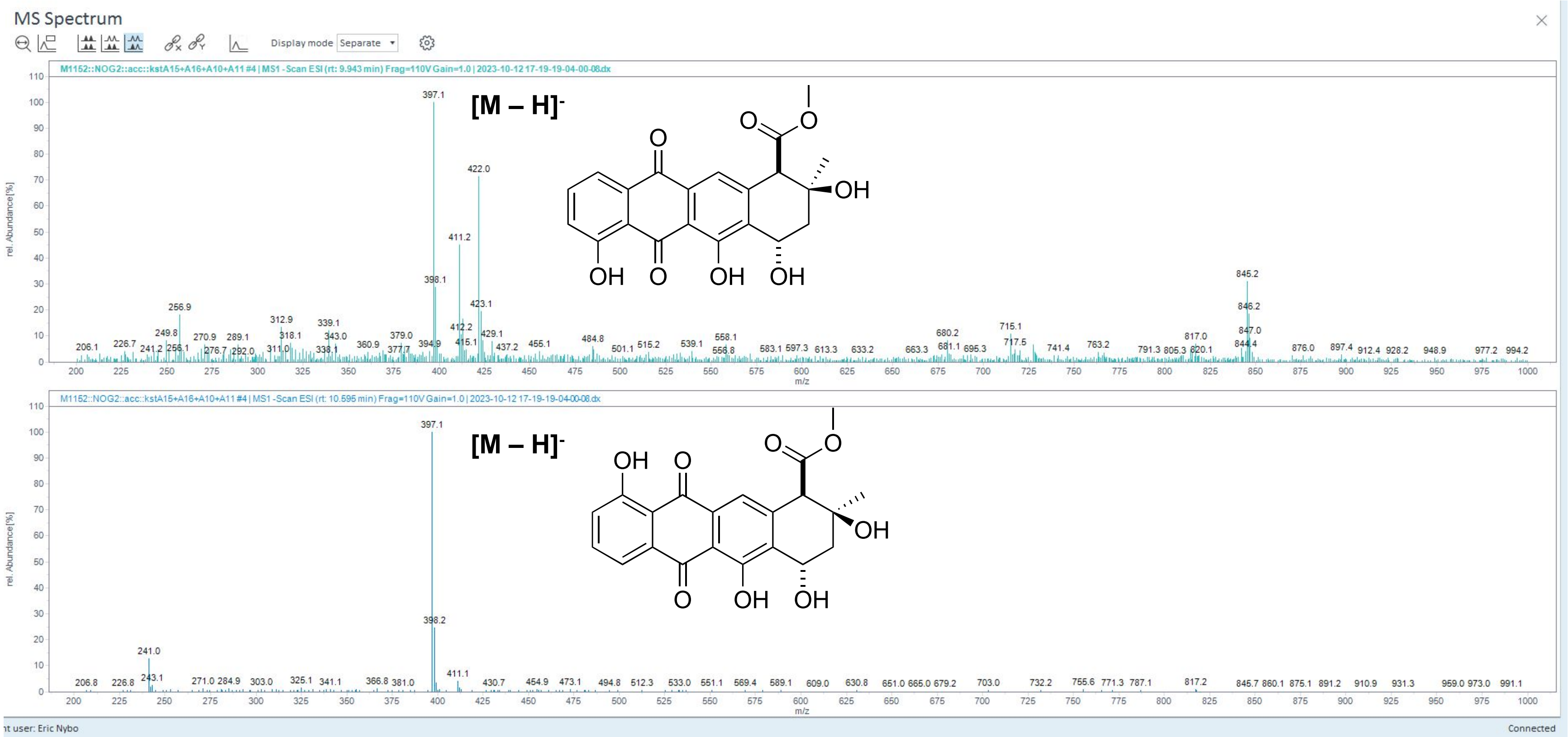

Figure S130. Mass spectrum of nogalamycinone (**4**) and *iso*-nogalamycinone (**34**) produced from a strain co-expressing the 9-*epi*-aklavinone pathway and *kstA15+A16+A10+A11.*

**Sample Name**	KS_FSU_E19	**Position**	P1-C9	**Instrument Name**	Instrument 1
**User Name**		**Inj Vol**	5	**InjPosition**	
**Sample Type**	Sample	**IRM Calibration Status**	Success	**Data Filename**	KS_FSU_E19.d
**ACQ Method**	Zheng_AQC ACC short_Neg.m	**Comment**		**Acquired Time**	11/14/2023 1:24:18 AM (UTC-05:00)

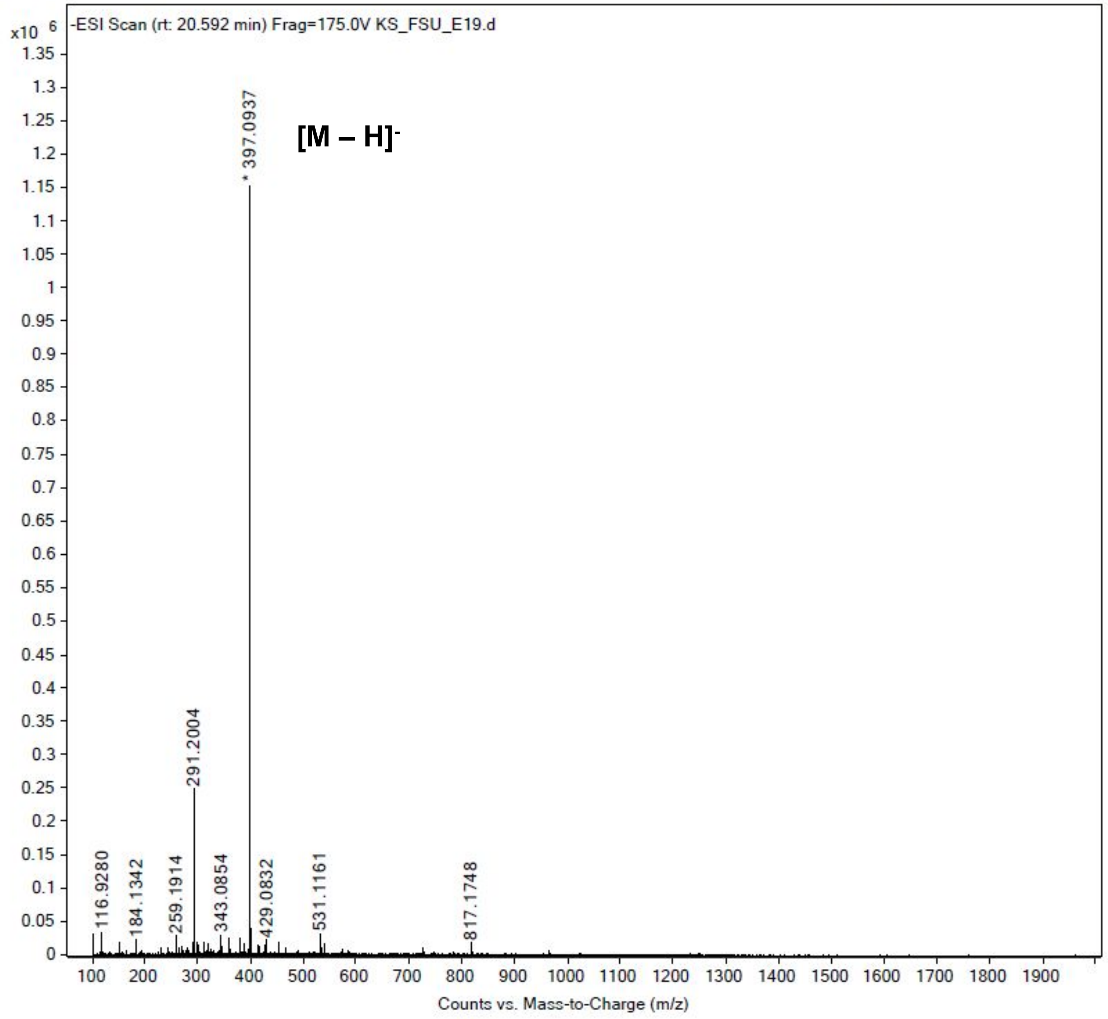

Figure S131. Figure (-)-HRESI-MS spectrum of 1-iso-nogalamycinone (**34**).

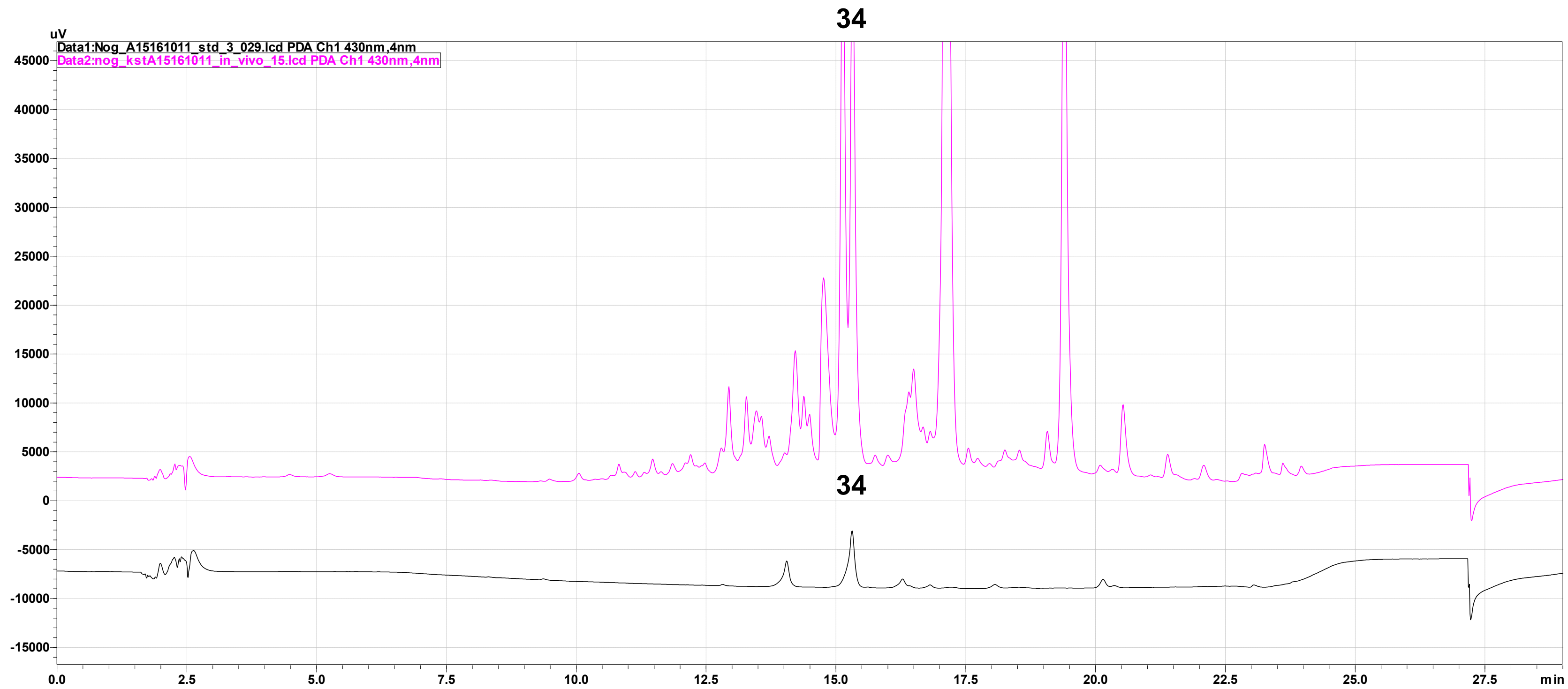

Figure S132. Alignment of *iso*-nogalamycinone (**34**) produced *in vitro* and *in vivo*.

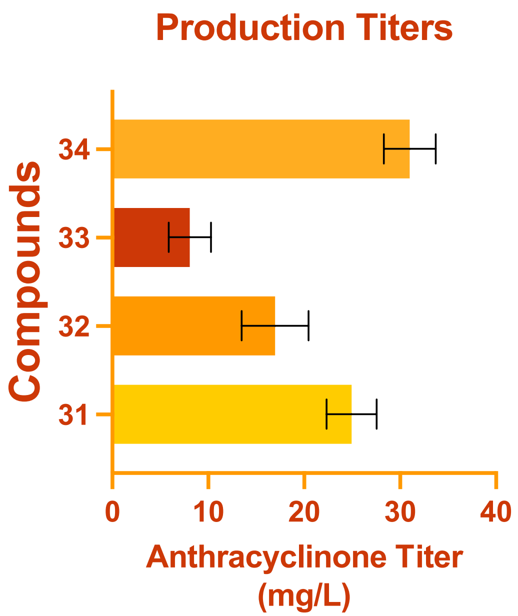

Figure S133. Production titers of **31-34** from strains expressing *kstA15+A16+A10+A11*.

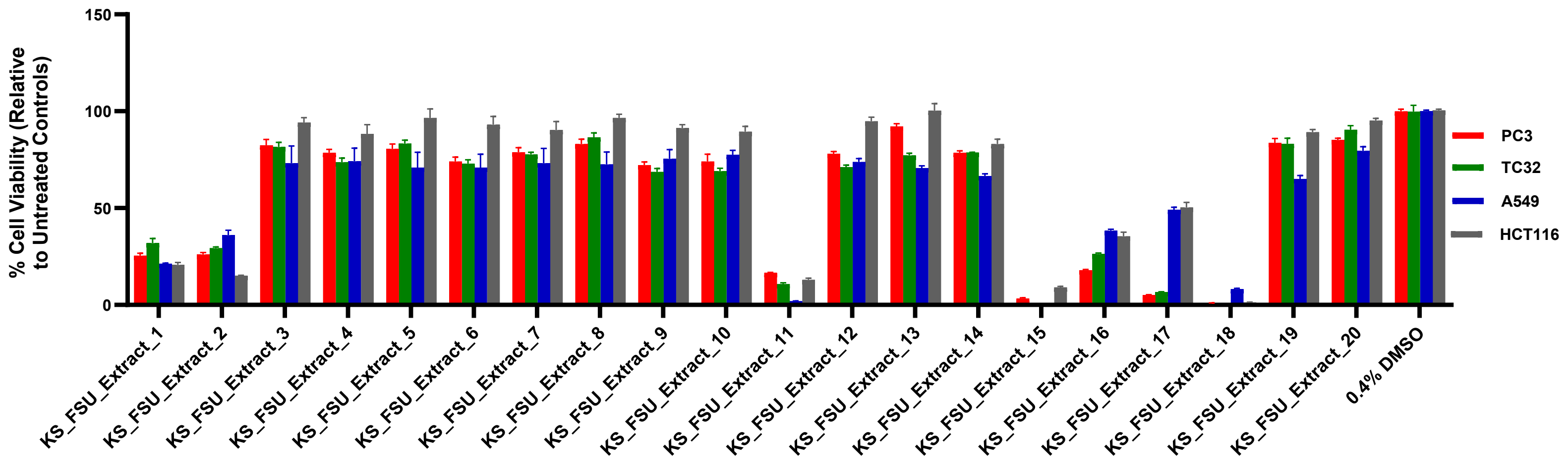

Figure S134. % Viability vs 100% of untreated control of the generated strains extracts at 40 μg/mL concentration.

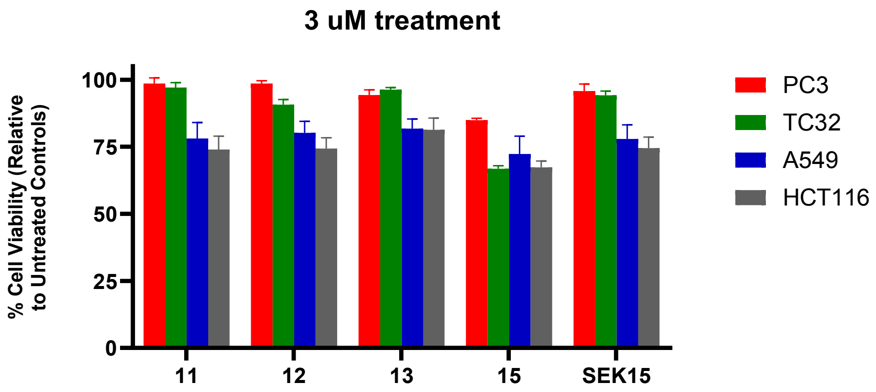

Figure S135. % Viability vs 100% of untreated control of compounds **11-13**, **15** and SEK15, at 3 μM concentration.

Table S6. Cytotoxic activities of the generated strain extracts in PC3, TC32, A549, and HCT116 cell lines.

Sample Name	Strain Extract	% viability vs 100% of untreated control (40 μg/mL)							
		**PC3**	SE	**TC32**	SE	**A549**	SE	**HCT116**	SE
AKV+*dnrF*	**KS_FSU_Extract_1**	25.43	0.74	31.93	1.34	21.27	0.17	20.70	0.70
AKV-*rdmECB*	**KS_FSU_Extract_2**	26.10	0.53	29.33	0.35	36.10	1.45	15.03	0.15
AKV-*eamCK*	**KS_FSU_Extract_3**	**82.43**	1.76	**81.57**	1.35	**73.13**	5.17	**94.23**	1.39
AKV-*kstA15+A16*	**KS_FSU_Extract_4**	78.57	0.98	73.63	**1.29**	74.13	**3.91**	88.33	**2.71**
AKV-*kstA15+A16+A10+A11*	**KS_FSU_Extract_5**	80.60	1.42	83.27	1.01	70.90	4.56	96.57	2.69
EAKV+*dnrF*	**KS_FSU_Extract_6**	74.00	1.33	72.97	1.13	70.93	3.94	93.07	2.46
EAKV-*rdmECB*	**KS_FSU_Extract_7**	78.80	1.40	77.67	0.68	73.20	4.39	90.33	2.49
EAKV-*eamCK*	**KS_FSU_Extract_8**	83.00	1.50	86.50	1.32	72.60	3.68	96.50	1.13
EAKV-*kstA15+A16*	**KS_FSU_Extract_9**	72.17	0.96	68.67	1.04	75.47	2.74	91.30	1.01
EAKV-*kstA15+A16+A10+A11*	**KS_FSU_Extract_10**	**74.07**	2.18	**69.07**	0.87	**77.60**	1.31	**89.47**	1.55
AURA-*rdmECB*	**KS_FSU_Extract_11**	16.60	0.15	10.83	**0.32**	1.93	**0.07**	12.93	**0.50**
AURA-*eamCK*	**KS_FSU_Extract_12**	78.03	0.66	71.20	0.55	73.80	0.98	94.87	1.22
AURA-*kstA15+A16*	**KS_FSU_Extract_13**	92.20	0.75	77.13	0.66	70.70	0.66	100.27	2.09
AURA-*kstA15+A16+A10+A11*	**KS_FSU_Extract_14**	78.60	0.53	78.63	0.13	66.50	0.65	83.00	1.46
NOG-dnrF	**KS_FSU_Extract_15**	3.30	0.21	-1.63	0.03	0.80	0.00	9.10	0.25
NOG-rdmECB	**KS_FSU_Extract_16**	17.90	0.21	26.27	0.29	38.37	0.44	35.40	1.23
NOG-eamCK	**KS_FSU_Extract_17**	**5.10**	0.15	**6.70**	0.06	**49.17**	0.73	**50.30**	1.56
NOG-kstA15+A16	**KS_FSU_Extract_18**	1.00	**0.06**	-2.43	**0.03**	8.20	**0.21**	1.30	**0.06**
NOG-kstA15+A16+A10+A11	**KS_FSU_Extract_19**	83.73	1.28	83.20	1.65	65.10	1.02	89.23	0.74
HEAKV	**KS_FSU_Extract_20**	85.20	0.52	90.47	1.20	79.53	1.21	95.17	0.69
	**0.4% DMSO**	99.93	0.66	99.87	1.83	100.03	0.33	100.40	0.35

% Viability values were obtained after 72 h incubation. Actinomycin D and H_2O_2 were used as positive control at 20 μM and 1 mM concentration, respectively (0% viable cells, n=3). SE= Standard Error

References

(1) Nguyen, J. T.; Riebschleger, K. K.; Brown, K. V.; Gorgijevska, N. M.; Nybo, S. E. A BioBricks Toolbox for Metabolic Engineering of the Tetracenomycin Pathway. *Biotechnol J* **2021**, 2100371. https://doi.org/10.1002/BIOT.202100371.

(2) Wang, R.; Nguyen, J.; Hecht, J.; Schwartz, N.; Brown, K.; Ponomareva, L.; Niemczura, M.; van Dissel, D.; van Wezel, G.; Thorson, J.; Metsä-Ketelä, M.; Shaaban, K.; Nybo, S. A BioBricks Metabolic Engineering Platform for the Biosynthesis of Anthracyclinones in <italic Toggle="yes">Streptomyces Coelicolor</Italic>. *ACS Synth Biol 11* (12), 4193–4209. https://doi.org/10.1021/acssynbio.2c00498.

(3) Aubry, C.; Pernodet, J. L.; Lautru, S. Modular and Integrative Vectors for Synthetic Biology Applications in Streptomyces Spp. *Appl Environ Microbiol* **2019**, *85* (16). https://doi.org/10.1128/AEM.00485-19.

(4) Lou, C.; Stanton, B.; Chen, Y. J.; Munsky, B.; Voigt, C. A. Ribozyme-Based Insulator Parts Buffer Synthetic Circuits from Genetic Context. *Nature Biotechnology 2012 30:11* **2012**, *30* (11), 1137–1142. https://doi.org/10.1038/nbt.2401.

(5) Yanisch-Perron, C.; Vieira, J.; Messing, J. *Improved Ml3 Phage Cloning Vectors and Host Strains: Nucleotide Sequences of the M13mp18 and PUC19 Vectors (Recombinant DNA; Molecular Cloning; Polycloning Sites; Progressive Deletions)*.

(6) Flett, F.; Mersinias, V.; Smith, C. P.; Flett ', F.; Mersinias, V.; Smith, C. P. High Efficiency Intergeneric Conjugal Transfer of Plasmid DNA from *Escherichia Coli* to Methyl DNA-Restricting Streptomycetes. *FEMS Microbiol Lett* **1997**, *155* (2), 223–229. https://doi.org/10.1016/S0378-1097(97)00392-3.

(7) Lindqvist, Y.; Koskiniemi, H.; Jansson, A.; Sandalova, T.; Schnell, R.; Liu, Z.; Mäntsälä, P.; Niemi, J.; Schneider, G. Structural Basis for Substrate Recognition and Specificity in Aklavinone-11-Hydroxylase from Rhodomycin Biosynthesis. **2009**, *393* (4), 966–977. https://doi.org/10.1016/j.jmb.2009.09.003.

(8) Grocholski, T.; Yamada, K.; Sinkkonen, J.; Tirkkonen, H.; Niemi, J.; Metsä-Ketelä, M. Evolutionary Trajectories for the Functional Diversification of Anthracycline Methyltransferases. *ACS Chem Biol* **2019**, *14* (5), 850–856. https://doi.org/10.1021/acschembio.9b00238.

(9) Dinis, P.; Tirkkonen, H.; Wandi, B. N.; Siitonen, V.; Niemi, J.; Grocholski, T.; Metsä-Ketelä, M. Evolution-Inspired Engineering of Anthracycline Methyltransferases. *PNAS Nexus* **2023**, *2* (2). https://doi.org/10.1093/PNASNEXUS/PGAD009.

(10) Siitonen, V.; Claesson, M.; Patrikainen, P.; Aromaa, M.; Mäntsälä, P.; Schneider, G.; Metsä-Ketelä, M. Identification of Late-Stage Glycosylation Steps in the Biosynthetic Pathway of the Anthracycline Nogalamycin. *ChemBioChem* **2012**, *13* (1), 120–128. https://doi.org/10.1002/cbic.201100637.
